# Effect of Health Risk Assessment and Counselling on Health Behaviour and Survival in Older People: A Pragmatic Randomised Trial

**DOI:** 10.1371/journal.pmed.1001889

**Published:** 2015-10-19

**Authors:** Andreas E. Stuck, André Moser, Ueli Morf, Urban Wirz, Joseph Wyser, Gerhard Gillmann, Stephan Born, Marcel Zwahlen, Steve Iliffe, Danielle Harari, Cameron Swift, John C. Beck, Matthias Egger

**Affiliations:** 1 Department of Geriatrics, Inselspital, University Hospital Bern and University of Bern, Bern, Switzerland; 2 Institute of Social and Preventive Medicine, University of Bern, Bern, Switzerland; 3 Institute of Primary Care, University of Bern, Bern, Switzerland; 4 Research Department of Primary Care and Population Health, University College London, London, United Kingdom; 5 Department of Ageing and Health, St. Thomas’ Hospital, London, United Kingdom; 6 Clinical Age Research Unit, King’s College London, London, United Kingdom; 7 University of California, Los Angeles, California, United States of America; 8 Langley Research Institute, Los Angeles, California, United States of America; Stanford University, UNITED STATES

## Abstract

**Background:**

Potentially avoidable risk factors continue to cause unnecessary disability and premature death in older people. Health risk assessment (HRA), a method successfully used in working-age populations, is a promising method for cost-effective health promotion and preventive care in older individuals, but the long-term effects of this approach are unknown. The objective of this study was to evaluate the effects of an innovative approach to HRA and counselling in older individuals for health behaviours, preventive care, and long-term survival.

**Methods and Findings:**

This study was a pragmatic, single-centre randomised controlled clinical trial in community-dwelling individuals aged 65 y or older registered with one of 19 primary care physician (PCP) practices in a mixed rural and urban area in Switzerland. From November 2000 to January 2002, 874 participants were randomly allocated to the intervention and 1,410 to usual care. The intervention consisted of HRA based on self-administered questionnaires and individualised computer-generated feedback reports, combined with nurse and PCP counselling over a 2-y period. Primary outcomes were health behaviours and preventive care use at 2 y and all-cause mortality at 8 y. At baseline, participants in the intervention group had a mean ± standard deviation of 6.9 ± 3.7 risk factors (including unfavourable health behaviours, health and functional impairments, and social risk factors) and 4.3 ± 1.8 deficits in recommended preventive care. At 2 y, favourable health behaviours and use of preventive care were more frequent in the intervention than in the control group (based on *z*-statistics from generalised estimating equation models). For example, 70% compared to 62% were physically active (odds ratio 1.43, 95% CI 1.16–1.77, *p* = 0.001), and 66% compared to 59% had influenza vaccinations in the past year (odds ratio 1.35, 95% CI 1.09–1.66, *p* = 0.005). At 8 y, based on an intention-to-treat analysis, the estimated proportion alive was 77.9% in the intervention and 72.8% in the control group, for an absolute mortality difference of 4.9% (95% CI 1.3%–8.5%, *p* = 0.009; based on *z*-test for risk difference). The hazard ratio of death comparing intervention with control was 0.79 (95% CI 0.66–0.94, *p* = 0.009; based on Wald test from Cox regression model), and the number needed to receive the intervention to prevent one death was 21 (95% CI 12–79). The main limitations of the study include the single-site study design, the use of a brief self-administered questionnaire for 2-y outcome data collection, the unavailability of other long-term outcome data (e.g., functional status, nursing home admissions), and the availability of long-term follow-up data on mortality for analysis only in 2014.

**Conclusions:**

This is the first trial to our knowledge demonstrating that a collaborative care model of HRA in community-dwelling older people not only results in better health behaviours and increased use of recommended preventive care interventions, but also improves survival. The intervention tested in our study may serve as a model of how to implement a relatively low-cost but effective programme of disease prevention and health promotion in older individuals.

**Trial Registration:**

International Standard Randomized Controlled Trial Number: ISRCTN 28458424

## Abstract 2

In a randomized trial, Andreas Stuck and colleagues assess the benefits of a collaborative care intervention to health behaviors and survival among elderly participants in Solothurn, Switzerland.

## Editors' Summary

**Background:**

The world’s population is getting older. In almost every country, the over–60 age group is growing faster than any other age group. In 2000, globally, there were about 605 million people aged 60 or more; by 2050, 2 billion people (many living in low- and middle-income countries) will be in this age group. But old age is not always a happy and healthy phase of life. Sadly, many older people find that their enjoyment of life is curtailed by chronic illnesses and increasing disability. Moreover, many older people die prematurely. In part, these adverse outcomes are linked to avoidable risk factors, particularly unhealthy lifestyles and failure to engage in preventative care. For example, older people commonly are physically inactive, smoke, drink too much alcohol, or do not have regular blood pressure checks or annual influenza vaccinations.

**Why Was This Study Done?:**

Programs that encourage a healthy lifestyle and the uptake of preventative care among older people are a health policy priority worldwide. But what is the best way to improve health and reduce premature death among older people? One promising approach is “health risk assessment.” In this multidimensional approach, which has been used successfully among working-age populations, older individuals complete a questionnaire to provide information about their risk factors for functional status decline and are subsequently given personalized feedback on how to promote health, maintain function, or prevent disease. Previous studies showed that this approach may improve short-term outcomes such as take-up of preventive care and health behaviors, but the long-term effects on health were unknown. Here, the researchers evaluate the effects of health risk assessment plus counseling on both short-term outcomes and on long-term survival among older people by undertaking a pragmatic randomized controlled trial in Solothurn, Switzerland. A randomized controlled trial compares the outcomes of individuals randomly chosen to receive or not receive an intervention; a pragmatic trial asks whether an intervention works under real-life conditions.

**What Did the Researchers Do and Find?:**

The researchers allocated 874 community-dwelling individuals aged 65 years or older living in a mixed rural and urban area in Switzerland to receive the intervention (the intervention group) and 1,410 individuals to receive usual care (the control group). The intervention consisted of health risk assessment based on self-administered questionnaires and individualized computer-generated feedback reports, combined with nurse and primary care physician counseling over a two-year period. At baseline, intervention group participants had about seven risk factors on average (including unfavorable health behaviors, health and functional impairments, and social risk factors) and 4–5 deficits in recommended preventative care. At two years, favorable health behaviors and use of preventative care were more frequent in the intervention group than in the control group, and these differences were statistically significant. For example, 70% of the intervention group were physically active compared to 62% of the control group, and 66% of the intervention group had had an influenza vaccination during the past 12 months compared to 59% of the control group. At eight years, 77.9% and 72.8% of the participants in the intervention and control groups, respectively, were still alive. Comparing the intervention group with the control group, the hazard ratio of death was 0.79. Finally, the researchers calculated that, to avert one death over eight years, 21 individuals would need to receive the intervention.

**What Do These Findings Mean?:**

These findings show that implementation of a collaborative care model of health risk assessment in community-dwelling older people resulted in better health behaviors, increased use of preventative care, and improved survival. Certain aspects of the trial design may limit the interpretation of these findings. For example, a self-administered questionnaire was used to collect the two-year health behavior outcome data, and some participants may have given socially desirable answers (for example, they may have understated their alcohol intake). Also, as the study was undertaken at a single site, these findings may not be generalizable. Moreover, the study was based on complete follow-up information on survival, but no long-term follow-up data were available for functional status outcome. Overall, however, these findings suggest that the use of health risk assessment combined with personal reinforcement of health risk assessment recommendations by specially trained counselors might be an effective and relatively low-cost way to promote good health among non-disabled older people. Moreover, the researchers suggest that it might be possible to adapt this model for use in low- and middle-income countries, where the challenge of a rapidly growing population of older people is greatest.

**Additional Information:**

This list of resources contains links that can be accessed when viewing the PDF on a device or via the online version of the article at http://dx.doi.org/10.1371/journal.pmed.1001889.

- The US National Institute on Aging provides information on health and aging (in English and Spanish)
- The UK National Health Service and Age UK (a not-for-profit organization) have produced a practical guide to healthy aging
- The World Health Organization provides information on many aspects of aging (in several languages); the WHO Study on Global Ageing and Adult Health is compiling longitudinal information on the health and well-being of adult populations and the aging process
- The United Nations Population Fund and HelpAge International publication Ageing in the Twenty-First Century is available
- HelpAge International is an international non-governmental organization that helps older people claim their rights, challenge discrimination, and overcome poverty, so that they can lead dignified, secure, and healthy lives
- More information on this trial, the Prevention in Older People–Assessment in Generalists’ Practices (PRO-AGE) trial, is available
- Wikipedia has a page on health risk assessment (note that Wikipedia is a free online encyclopedia that anyone can edit; available in several languages)

## Introduction

An increasing number of older individuals are affected by multiple risks and morbidities, leading to functional impairment, nursing home admissions, or premature death, with enormous social and economic costs to society [1]. These adverse outcomes might at least in part be avoidable. For example, recent studies demonstrate a continued high prevalence of unhealthy behaviours and preventive care deficits in older individuals despite evidence supporting the importance of healthy lifestyles and optimal preventive care in later life [2]. Also, early identification of, and intervention for, previously unknown health and functional deficits may contribute to better outcomes in older people [3]. The search for, and the implementation of, multimodal programmes for cost-effective disease prevention and health promotion has therefore become a top health policy priority worldwide.

It has been shown that multimodal interventions may substantially improve health status and reduce mortality for frail or disabled older individuals. For example, one randomised controlled trial found that chronically ill older adults who were offered a community-based nurse intervention had a 25% lower risk of death as compared to control group individuals with usual care [4]. However, for non-disabled older individuals, previous studies have revealed inconsistent findings. For example, a meta-analysis of trials of systematic health checks for general adult populations concluded that these interventions did not have favourable effects on mortality, perhaps since these programmes were organised in parallel to, and not aligned with, primary care [5]. Moreover, systematic analyses of multimodal preventive care home visit programmes found no consistent effects on mortality and other outcomes, although some studies found that these programmes significantly reduced or delayed nursing home admissions in older individuals [6].

Health risk assessment (HRA) has recently received attention as a method for multidimensional preventive care intervention among older individuals [7,8]. Originally developed for workforce health promotion, HRA is based on self-reports to guide risk factor interventions, with subsequent individualised feedback to participants on their health status and on how to promote health, maintain function, or prevent disease [9,10]. HRA is a potentially promising approach for use in older individuals, with scientific evidence for favourable effects on intermediate outcomes such as health behaviours and use of preventive care [7,8,11]. However, studies have found that HRA-based interventions were effective for intermediate outcomes only if older individuals received HRA combined with some form of personal reinforcement [7,8,11]. For example, this was confirmed by the findings of two recent randomised controlled trials funded by the European Union [12,13]. One trial conducted in London (UK) tested the effects of a single HRA. This HRA was combined with an electronic health record reminder system for use in the primary care practice setting, but it is not known to what extent these reminders were actually used for counselling [12]. At 1-y follow-up, this study found no or only minimal intervention effects on health behaviours and preventive care use among older individuals, which is consistent with the fact that personal reinforcement was likely minimal [12]. The other trial was conducted in Hamburg (Germany) [13]. It also offered an initial HRA in the primary care setting, and, in addition, older individuals in the intervention arm participated in a half-day group counselling session or, alternatively, received an initial home visit with individual counselling. This trial found mild to moderate favourable intervention effects on health behaviours and preventive care among older individuals, which is consistent with the fact that this intervention ensured some amount of reinforcement of HRA-based recommendations [13].

Although multiple earlier studies have addressed intermediate outcomes of HRA-based interventions, a 2011 systematic analysis found no controlled study with long-term health outcomes of the effect of HRA on mortality or functional status in older individuals [8], and, to our knowledge, no new study of this type has been published since then. We designed a randomised controlled study with a system to collect intermediate and long-term follow-up data, using an intention-to-treat approach. The purpose of this study is to confirm whether a HRA-based intervention with a reliable long-term system of reinforcement has favourable effects on health behaviours and preventive care use in community-dwelling older individuals, and to evaluate whether this intervention also results in favourable long-term outcomes.

## Methods

### Ethical Review

The study was approved by the Ethics Committee of the Canton of Solothurn (EKO–0023) and the Ethics Committee of the Canton of Bern (205/06).

### Study Design

The study methods and selected baseline findings of the present trial conducted in Solothurn have been previously published [14,15], and the detailed study protocol and analysis plan are available in S1 Text. The study was conducted at the offices of 19 primary care physicians (PCPs) serving two mixed rural and urban primary care catchment areas in the Canton of Solothurn in Switzerland. Recruitment began November 16, 2000, and ended January 8, 2002. The study received funding from the European Union as part of the PRO-AGE (Prevention in Older People–Assessment in Generalists’ Practices) study and from regional foundations. The PRO-AGE study consists of three trials of HRA conducted in Solothurn (the present trial), Hamburg, and London. The two trials conducted in Hamburg and London were designed as short-term trials, and the final results of these trials (including effects on preventive care use and health behaviours at 1-y follow-up) have been published [12,13].

### Study Participants

The PCPs generated lists of all patients aged 65 y or older whom they had seen at least once over the past 5 y. Patients with disability (defined as needing human assistance for performing basic activities of daily living) [16], cognitive impairment (equivalent to a Mini Mental State Examination score of 24 or less) [17], terminal disease, or inability to speak German were excluded. Remaining patients who gave written informed consent were sequentially listed for enrolment by the local study centre based in Solothurn, and were randomly allocated to the intervention and control groups by the study centre based at the University of Bern using a computer-generated allocation sequence. Individuals living in the same household were allocated to the same group. Participants allocated to the control group continued to receive usual care from their PCPs.

### Interventions

The Health Risk Assessment for Older Persons (HRA-O) questionnaire was developed based on a systematic literature review [14,18,19] and expert panel consensus. Experts selected risk factors for functional status decline based on four criteria: potential impact on functional impairment, strength of evidence, potential for risk reduction, and feasibility of assessment. For each risk factor, assessment questions were selected based on reliability, validity, feasibility, and previous use in large studies of older individuals. The risk factors included unfavourable health behaviours, health and functional impairments, and social risk factors (S1 Table). For health behaviours, questions on participants’ intention to change unfavourable behaviours were added [20]. In addition, the expert panel also selected 11 preventive care recommendations for inclusion in the questionnaire based on the 1996 guidelines of the US Preventive Services Task Force [21]. Field tests among community-dwelling older individuals in the US, the UK, Germany, and Switzerland demonstrated the acceptance and feasibility of the HRA-O questionnaire [12–14,22]. The UK English version was translated and regionally adapted to the German language (for UK English and German versions of the HRA-O questionnaire, see S3 and S4 Texts). For this trial, an intervention manual prepared for use in UK primary care practices was translated, regionally adapted, and modified for use by nurse counsellors and PCPs. This manual was used as training material and as a reference guide for the PCPs and nurse counsellors involved in the intervention (for UK English and German versions of the intervention manual, see S5 and S6 Texts). The role of the health professionals in the intervention is summarised in Table 1.

**Table 1:** Role of health professionals in the intervention.

Health Professional	Role
**PCPs**	Sent baseline and 1-y follow-up HRA-O questionnaire to participants, and received provider feedback reports, for use in clinical care^a^
	In case discussion with nurse counsellors, approved/modified plan for each participant’s preventive care goals, taking into account participant’s priorities
	Were encouraged to reinforce recommendations related to health behaviours and to implement preventive care measure changes during routine office visits, and to refer participants for specialist preventive care
**Nurse counsellors**	Received baseline and 1-y follow-up HRA-O provider feedback report on participants’ problems and risks, and visited participants at home to obtain additional information on problems and risks as needed
	Prepared a tentative plan for each participant’s preventive care goals for case discussion with geriatrician and subsequent approval by PCP
	Selected and prioritised preventive care goals for each participant based on baseline and yearly case discussions with geriatrician and PCP (main criteria: relevance of the risk factor for adverse outcomes, potential for successful risk factor modification, and participant’s self-reported readiness to change)
	Made phone calls (3 mo after baseline, and additionally if needed) and home visits (at baseline and every 6 mo, and additionally if needed) to discuss the individualised HRA-O participant feedback reports with participants and to motivate participants to adhere to recommendations
	Supported participants in implementing preventive care goals by empowering participants to address risks, reminding them of non-completed recommendations, and facilitating appropriate referrals to health and social care agencies
	Had weekly interactive training sessions
**Geriatricians**	Trained nurse counsellors with initial and subsequent monthly training sessions, based on intervention manual^b^
	Offered training to PCPs with initial and subsequent quarterly interactive group sessions, based on intervention manual^b^
	Were available for specialist advice for PCPs

^a^For HRA-O questionnaire, see S3 and S4 Texts.

^b^For intervention manual, see S5 and S6 Texts.

At baseline and 1-y follow-up, PCPs sent a HRA-O questionnaire to patients allocated to the intervention arm. Based on completed HRA-O questionnaires, individualised computer-generated participant and provider feedback reports were generated and returned to the PCPs and the participants. PCPs used the reports to motivate patients to reduce unhealthy behaviours in collaboration with the nurse counsellors, to implement preventive care interventions (e.g., influenza vaccination, blood pressure measurement), and to refer patients for specialty-based preventive care (e.g., breast cancer screening, ophthalmology referral). Over the 2-y intervention period, nurse counsellors visited participants at home (at baseline and every 6 mo, and additionally if needed) and contacted them by phone (at 3 mo, and additionally if needed) to evaluate risks and reinforce HRA-O-based recommendations. The nurse counsellors had one initial meeting and then meetings each year during the 2-y intervention period with the geriatricians to refine recommendations for each participant. The PCPs and nurse counsellors received training and support from project geriatricians.

### Study Assessments and Outcomes

Baseline data were obtained from practice registers, a brief pre-randomisation questionnaire including questions to calculate the Pra score (a previously validated overall risk score identifying older people at high risk for adverse health outcomes [23]), and the Swiss Federal Population Census 2000 through record linkage with the Swiss National Cohort [24,25]. At 1-y follow-up, a long self-administered questionnaire was sent to surviving participants for short-term outcome analysis, but due to a high rate of non-return of these questionnaires, these data could not be used for further analyses (further details in S1 Text). At 2 y, surviving participants were sent a short validated questionnaire to measure six health-related behaviours [26], dependency in basic activities of daily living, and self-perceived health status. Nonresponding participants were contacted by trained interviewers blinded to group allocation, and were interviewed face-to-face if possible. Participants’ adherence to preventive care recommendations usually performed in PCP practices were abstracted from PCP records by data extractors blinded to group allocation. Since PCPs saw patients only during routine clinical care and often not at the time of 2-y follow-up, an initial plan to collect 2-y measurement data in the practice setting could not be realised. For logistic reasons, 2-y follow-up data were not available for participants living in nursing homes at the 2-y follow-up time point. At 2 y, participating PCPs were sent a brief questionnaire on their perception of the intervention.

At 2 y, primary outcomes were adherence to six recommended health behaviours (physical activity, fruit/vegetable/fibre intake, fat intake, seat belt use, tobacco consumption, alcohol use) and use of six preventive care services (blood pressure measurement, cholesterol measurement, glucose measurement, influenza vaccination, pneumococcal vaccination, faecal occult blood testing). An initial plan to use composite variables (e.g., by calculating an overall adherence rate for summarising the information on adherence to each of the six recommended health behaviours) was dropped because the main study hypothesis was to test the effects on individual, and not on combined, items. Secondary outcomes were nursing home admissions, dependency in basic activities of daily living, and self-perceived health status. At 8 y, the primary outcome was all-cause mortality, and the secondary outcome was cause-specific mortality. Vital status at the end of 2008 was ascertained for all study participants, either through probabilistic linkage with the Swiss National Cohort [24] or, if linkage was unsuccessful, from municipal registers. The underlying cause of death was ascertained from the death certificate, based on the International Classification of Diseases–Tenth Revision (ICD–10).

### Sample Size and Statistical Analysis

The number of participants needed to demonstrate a 1.3-fold increase in the prevalence of positive health behaviours or preventive care use with 80% power at a significance level of 0.05 was 1,000 individuals in each group, assuming a control group prevalence of 20%, and a 20% dropout rate. For a 1:2 randomisation (intervention to control) ratio, the required numbers were 732 individuals in the intervention and 1,464 in the control group. We changed the randomisation ratio from 1:1 to 1:2 on March 27, 2001, when resource constraints mandated a reduction of the size of the intervention group. Enrolment was terminated on January 8, 2002, when the required sample size was reached.

Comparisons of the prevalence of healthy behaviours and adherence to preventive care at 2 y were based on modified (i.e., using imputation methods for handling missing data) intention-to-treat analyses based on all surviving participants. We used multiple imputation by chained equations assuming a missing-at-random situation [27]. Analyses were run on 25 imputation datasets, and the results were combined with Rubin’s rule [28]. In sensitivity analyses we used the complete case dataset, excluding individuals with missing data. Further, we conducted a sensitivity analysis to test the potential impact of attrition bias due to loss to follow-up of individuals at 2 y [29]. We used inverse-probability-of-attrition weighting to examine the influence of attrition bias on group allocation and 2-y outcomes [30]. Standard intention-to-treat analyses were used for mortality analysis. We used generalised estimating equation models with an underlying equicorrelation structure to compare health behaviour and preventive care outcomes [31]. Survival was analysed using Kaplan-Meier life table methods and Cox regression models, with time from the date of randomisation to the date of death or 31 December 2008, as the underlying timescale. Maximal individual observation time was restricted to 8 y of follow-up. The proportional-hazards assumption was tested by Schoenfeld’s test [32]. All analyses were unadjusted. A *p*-value of less than 0.05 in two-sided test statistics was considered to indicate statistical significance. The number needed to treat was calculated from absolute risk differences over the follow-up period [33,34]. Models accounted for the allocation of individuals living in the same household to the same group. The effect of the intervention in pre-specified subgroups at low and high risk for adverse health outcomes (high risk defined as a Pra score ≥ 0.286) was assessed by treatment–subgroup interactions. Analyses were done using Stata 12.1 (Stata Corp) or R 3.0.1 (R Foundation for Statistical Computing) software.

## Results

**Fig 1:**
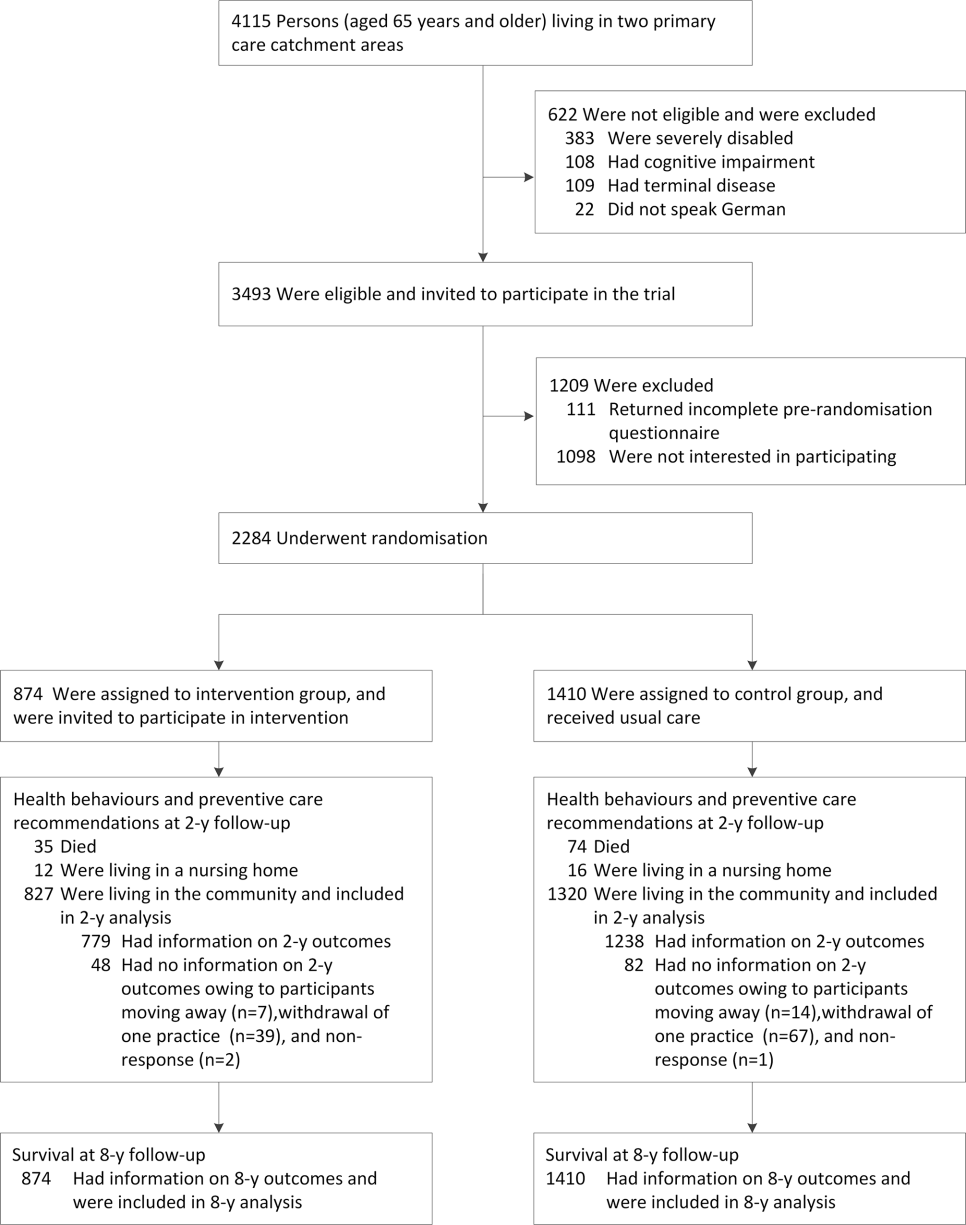
PRO-AGE Solothurn CONSORT diagram. The randomisation ratio (intervention to control group) was 1:1 in the first project phase (November 16, 2000, to March 27, 2001), and 1:2 in the second project phase (March 28, 2001, to January 8, 2002), resulting in a ratio overall of 1:1.6.

A total of 4,115 patients aged 65 y and older were assessed for eligibility, 3,493 were eligible, and 2,284 were included in the study and underwent randomisation (Fig 1).

In all, 874 participants were allocated to the intervention group, and 1,410 to the control group. There were no significant differences (*p* < 0.05; based on *z*-statistics from generalised estimating equation models) between the intervention and control groups in any of the baseline characteristics listed in Table 2.

**Table 2:** Baseline characteristics of study participants.

Characteristic	Intervention Group, *n =* 874	Control Group, *n =* 1,410
**Age at randomisation (years)**	74.5 ± 5.8	74.5 ± 6.1
**Gender: female**	497 (56.9)	796 (56.5)
**Hospital use in past year: ≥1 admissions** ^**a**^	174 (19.9)	261 (18.5)
**Doctor visits in past year: ≥7 visits** ^**a**^	210 (24.0)	343 (24.3)
**Self-perceived health** ^**a**^		
Excellent	22 (2.5)	33 (2.3)
Very good	133 (15.2)	189 (13.4)
Good	545 (62.4)	839 (59.5)
Fair	168 (19.2)	338 (24.0)
Poor	6 (0.7)	11 (0.8)
**Self-reported diabetes** ^**a**^	91 (10.4)	169 (12.0)
**Self-reported coronary heart disease** ^**a**^	189 (21.6)	325 (23.0)
**No informal caregiver available if needed** ^**a**^ ^**,**^ ^**b**^	86 (9.8)	163 (11.6)
**Pra score** ^**c**^	0.29 ± 0.10	0.29 ± 0.11
**Highest completed education** ^**d**^		
Compulsory education or less (≤9 y)	388 (44.4)	606 (43.0)
Secondary-level education (10–12 y)	399 (45.7)	643 (45.6)
Tertiary-level education (>12 y)	68 (7.8)	126 (8.9)
Unknown	19 (2.2)	35 (2.5)
**Living arrangement** ^**d**^		
Living alone	261 (29.9)	404 (28.7)
Not living alone	600 (68.6)	977 (69.3)
Unknown	13 (1.5)	29 (2.1)
**Marital status** ^**d**^		
Single	37 (4.2)	73 (5.2)
Married	548 (62.7)	875 (62.1)
Widowed	258 (29.5)	399 (28.3)
Divorced	18 (2.1)	34 (2.4)
Unknown	13 (1.5)	29 (2.1)
**Religious affiliation** ^**d**^		
Protestant	461 (52.7)	735 (52.1)
Catholic	364 (41.6)	571 (40.5)
No religious affiliation	14 (1.6)	34 (2.4)
Other/unknown	35 (4.0)	70 (5.0)
**Socio-economic status: Swiss neighbourhood index** ^**e**^	61.2 ± 7.3	60.8 ± 7.4

Data are mean ± standard deviation (SD) or *n* (percent).

^a^Based on self-reported information from pre-randomisation baseline questionnaire.

^b^Said “no” to the following question: “Is there a friend, relative, or neighbour who would take care of you for a few days if necessary?”

^c^The Pra score is calculated from the person’s age, gender, hospital admissions, doctor visits, health status, diabetes status, heart disease status, and caregiver availability [23].

^d^Based on linkage with data from Swiss Federal Population Census 2000.

^e^Higher scores denote higher levels of socio-economic status [25].

### Uptake of the Intervention

At baseline, 748 (85.6%) of the 874 participants allocated to the intervention group returned the HRA-O questionnaire. It revealed a mean ± SD of 6.9 ± 3.7 risk factors for functional status decline for these participants (Table 3).

**Table 3:** Prevalence rates of risk factors for functional status decline among study participants in the intervention group at baseline (*n =* 748).

Risk Factor Domain	Risk Factor	*n* (Percent) or Mean ± SD
**Accident prevention**	Does not always wear a seat belt	90 (12.0)
**Activities of daily living**	Difficulty/need for human assistance in ≥2 IADL items	135 (18.0)
	Changed kind of mobility activity (preclinical mobility disability)	366 (48.9)
	Decreased frequency of mobility activity (preclinical mobility disability)	262 (35.0)
**Alcohol use**	Possible misuse of alcohol	85 (11.4)
**Falls**	Repeated (≥1) falls in past 12 mo	50 (6.7)
	Self-reported limitation of activities due to fear of falling	167 (22.3)
**Health status**	Self-perceived health status “moderate” or “poor”	116 (15.5)
**Hearing**	Impaired hearing	178 (23.8)
**Incontinence**	Urinary incontinence on >5 d in past 12 mo	144 (19.3)
**Medication use**	Use of ≥4 medications	200 (26.7)
	Total number of medications used	2.6 ± 2.2
	Use of long-acting benzodiazepine or amitriptyline	54 (7.2)
	Self-reported medication side effects	64 (8.6)
	Possible adverse reaction to prescribed medication	33 (4.4)
**Medical history**	Presence of ≥3 chronic conditions	279 (37.3)
	Number of chronic conditions	2.1 ± 1.6
**Memory**	Memory problems	46 (6.1)
**Mood**	Depressive mood	105 (14.0)
**Nutrition**	Body mass index < 20 kg/m^2^	14 (1.9)
	Body mass index ≥ 27 kg/m^2^	375 (50.1)
	Body mass index (kg/m^2^)	27.2 ± 4.5
	Loss of weight (≥5 kg in past 6 mo)	35 (4.7)
	Consumption of >2 high-fat food items per day	354 (47.3)
	Consumption of <5 fruit/fibre items per day	489 (65.4)
**Oral health**	Oral health problem	188 (25.1)
**Pain**	Presence of moderate to severe pain	166 (22.2)
**Physical activity**	Moderate or strenuous physical activity on <5 d/wk	524 (70.1)
**Social factors**	Low level of emotional support	64 (8.6)
	High risk of social isolation	66 (8.8)
	Marginal family ties	45 (6.0)
	Marginal friendship ties	126 (16.8)
	No participation in social groups or organisations	149 (19.9)
**Tobacco use**	Current tobacco use	86 (11.5)
**Vision**	Problem in ≥1 vision sub-domains	93 (12.4)

Based on self-report data from 748 participants of the intervention group on the baseline HRA-O questionnaire. Participant nonresponse was categorised as absence of risk (this was for participants who completed some of the questionnaire but missed parts). Participant nonresponse ranged between 17 and 184 for the risk factors listed in the table). For detailed definitions and references of instruments, see S1 Table.

IADL, instrumental activities of daily living.

For example, 167 (22.3%) participants reported fear of falling [35], 262 (35.0%) reported that they had reduced the frequency of mobility activities (e.g., walking, climbing stairs) in the past year [36], and 354 (47.3%) reported high intake of fatty foods. Only a small minority of participants reported an intention to change adverse health behaviour; for example, only six (1.6%) of the 354 participants reporting high intake of fatty foods reported plans to reduce their fat intake in the near future (S2 Table). In addition, the questionnaire revealed a mean ± SD of 4.3 ± 1.8 deficits per participant among the 11 recommended preventive care recommendations, with ≥1 deficits in 731 participants (S3 Table). Overall, 586 (80.2%) of the 731 participants with ≥1 deficits did not realise that they had deficits in preventive care (S4 Table).

Among the 874 participants in the intervention group, 514 (58.8%) received the intervention for the entire 2-y period, with a mean of 5.3 nurse counsellor visits and 2.0 telephone contacts. Ninety-four (10.8%) participants declined nurse counselling, but received the PCP component of the intervention for the 2-y period. In addition, 126 (14.4%) participants did not receive the intervention because they did not return the baseline HRA-O questionnaire. The remaining 140 (16.0%) participants received the full intervention (including nurse counselling), but the intervention was terminated prior to the 2-y follow-up time point due to death (*n =* 21), nursing home admission (*n =* 6), withdrawal of one PCP practice from the project (*n =* 25), or participant request (*n =* 88).

Of the 19 PCPs, 18 participated in the intervention for the entire 2-y time period, and one PCP withdrew from the project in the second year for personal reasons. Sixteen PCPs responded to questions on their perception of the preventive care intervention at the end of the 2-y follow-up (S5 Table). Most of them did not feel resource constraints limited them in offering the recommended preventive care services to their patients. All 16 PCPs considered the evidence for recommending yearly influenza vaccinations to older individuals as strong, but some PCPs considered the evidence as weak for recommending other preventive care measures (e.g., ten of the 16 PCPs considered the evidence for recommending colon cancer screening as weak). PCPs and nurse counsellors did not report any harm resulting from the intervention.

### Outcomes at 2-y Follow-Up

Overall, 827 participants in the intervention group and 1,320 in the control group survived and were living in the community at 2-y follow-up and were included in the 2-y follow-up analyses, which included imputation of missing data (see Fig 1 and S6 Table for information on missing data). Table 4 summarises primary outcomes at 2-y follow-up.

**Table 4:** Primary outcomes at 2-y follow-up: health behaviours and adherence to preventive care recommendations.

Outcome	Intervention Group, *n* (Percent)	Control Group, *n* (Percent)	Odds Ratio (95% CI)	*p*-Value
**Health behaviours**				
Medium to high level of physical activity (daily average ≥ 30 min)	580 (70.1)	820 (62.1)	1.43 (1.16–1.77)	0.001
Medium to high level of fruit/vegetable/fibre intake (≥2 portions per day)	386 (46.7)	511 (38.7)	1.40 (1.15–1.70)	0.001
Low level of fat intake (<2 portions of high-fat items per day)	249 (30.1)	332 (25.2)	1.35 (1.08–1.68)	0.008
Always use of seat belt	734 (88.8)	1,117 (84.6)	1.42 (1.06–1.92)	0.02
No tobacco consumption	742 (89.7)	1,180 (89.4)	1.03 (0.75–1.42)	0.86
No or little alcohol use (≤1 alcoholic drink per day)	773 (93.5)	1,186 (89.8)	1.64 (1.15–2.33)	0.006
**Preventive care recommendations**				
Blood pressure measurement in past 1 y	759 (91.8)	1,168 (88.5)	1.45 (1.06–2.00)	0.02
Cholesterol measurement (individuals aged <75 y) in past 5 y	435 (90.2)^a^	676 (86.2)^a^	1.48 (1.02–2.13)	0.04
Glucose measurement in past 3 y	670 (81.0)	1,014 (76.8)	1.29 (1.03–1.62)	0.03
Influenza vaccination in past 1 y	544 (65.8)	781 (59.2)	1.35 (1.09–1.66)	0.005
Pneumococcal vaccination (ever)	259 (31.3)	266 (20.2)	1.90 (1.52–2.37)	<0.001
Faecal occult blood test in past 1 y (individuals aged <80 y)	191 (28.1)^b^	234 (21.5)^b^	1.45 (1.15–1.85)	0.002

Modified intention-to-treat analysis based on all participants surviving in the community, with multiple imputation for missing values (intervention group, *n =* 827; control group, *n =* 1,320). Odds ratios and *p*-values based on *z*-statistics from generalised estimating equation models. For analysis with complete case dataset alone (i.e., dataset without imputed data), see S7 Table. Control group is reference group.

^a^Denominator includes individuals aged <75 y only: intervention group, *n =* 482; control group, *n =* 784 (individuals aged ≥75 y were excluded since the recommendation for cholesterol measurement was given to individuals aged <75 y only).

^b^Denominator includes individuals aged <80 y only: intervention group, *n =* 680; control group, *n =* 1,089 (individuals aged ≥80 y were excluded since the recommendation for faecal occult blood testing was given to individuals aged <80 y only).

Health behaviours related to physical activity, diet, seat belt use, and alcohol consumption in the intervention group were better than in the control group (Table 4). For example, in the intervention group 70.1% of individuals reported being physically active on average at least 30 min per day compared to 62.1% in the control group. Adherence to the preventive care recommendations was also greater in the intervention group than in the control group (Table 4). Complete case analyses yielded similar results (S7 Table). Also, the results of the sensitivity analyses with inverse-probability-of-attrition weighting for investigating attrition bias were similar to complete case and multiple imputation results (S7 Text).

There were no statistically significant differences between the intervention and control groups for self-reported dependency in basic activities of daily living (S8 Table) or for nursing home admissions (S9 Table) at 2-y follow-up.

### Outcomes at 8-y Follow-Up

Vital status at the end of 2008 could be ascertained for all study participants, either through linkage with the Swiss National Cohort (for 2,242 patients, 98.2%) or, if linkage was unsuccessful, from municipal registers (42 patients, 1.8%). Length of follow-up ranged from 6.8 y to 8.2 y; the median length of follow-up was 7.7 y in both groups. We compared the mortality data from record linkage at 2 y with the data from medical record abstraction at 2-y follow-up. In 2,080 participants the information was available from both sources, and the accuracy was >99%.

**Fig 2:**
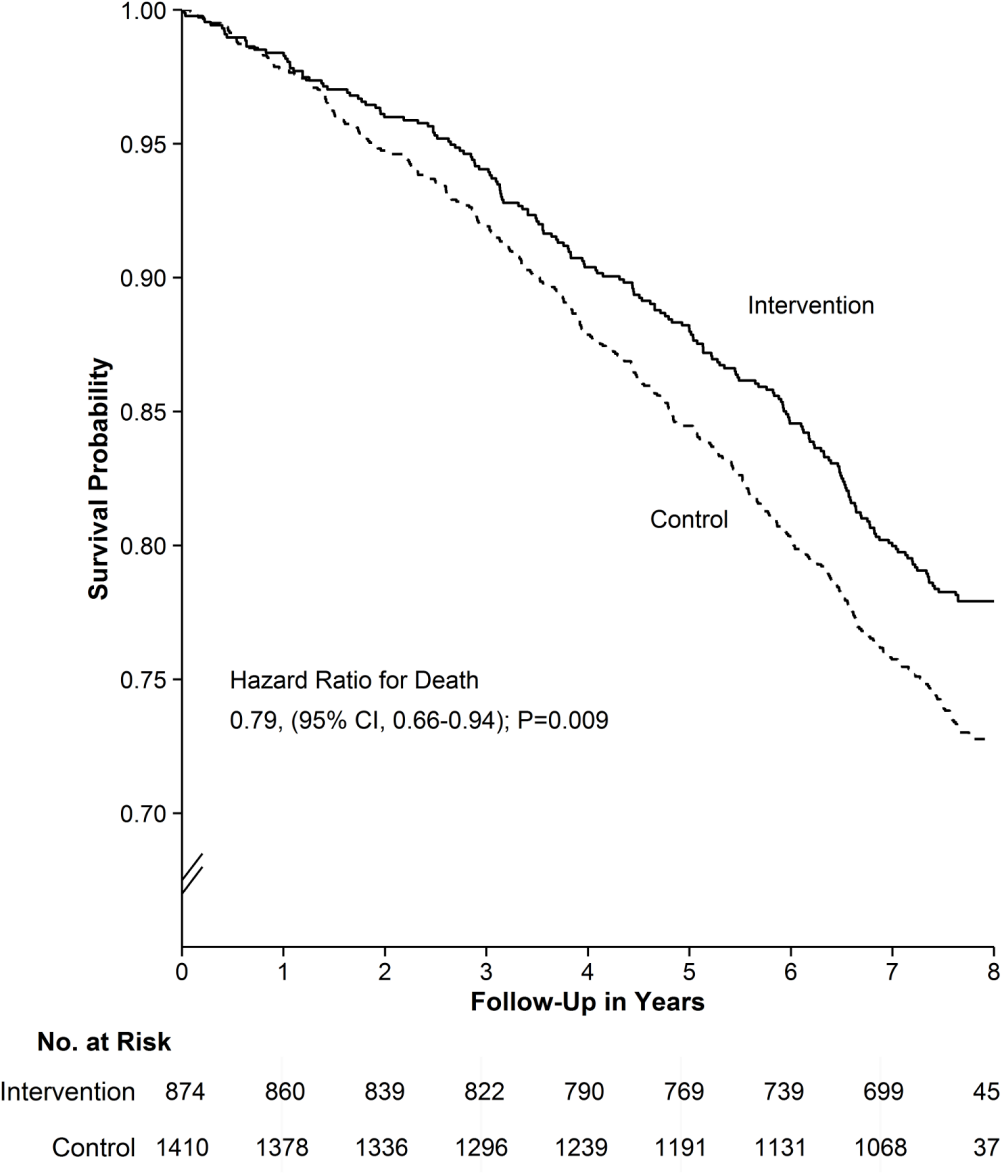
Probability of survival. The primary outcome at 8-y follow-up was all-cause mortality. Based on Kaplan-Meier estimates of survival.

Over the 8-y follow-up, the mortality rate was 3.16 (95% CI 2.74–3.63) per 100 person-years in the intervention group, as compared to 3.97 (95% CI 3.59–4.39) in the control group; the hazard ratio was 0.79 (95% CI 0.66–0.94, *p* = 0.009; based on Wald test from Cox regression model) (Fig 2). Sensitivity analyses with adjustment for two key baseline variables (self-perceived health and access to informal caregiver support) yielded similar results (S10 Table).

The estimated proportion alive at 8 y was 77.9% (95% CI 75.2%–80.7%) in the intervention and 72.8% (95% CI 70.4%–75.2%) in the control group, for an absolute mortality difference of 4.9% (95% CI 1.3%–8.5%, *p =* 0.009; based on *z*-test for risk difference). The number needed to treat was 21 (95% CI 12–79) (i.e., 21 individuals needed to receive the intervention to prevent one death over 8 y). Table 5 lists the detailed intervention effects for the two most frequent causes of death (i.e., circulatory system diseases and neoplasm). Deaths due to other types of disorders were classified as “other and unknown cause of death” because the numbers were too low for separate analyses. The combined mortality rate for diseases of the circulatory system was lower for the intervention group than for the control group (*p* = 0.03; based on Wald test from Cox regression model). There were no other statistically significant differences in cause-specific mortality rates (Table 5).

**Table 5:** Secondary outcome at 8-y follow-up: mortality rates for main causes and sub-causes of death.

Cause of Death (ICD–10 Codes)	Intervention Group, *n =* 874		Control Group, *n =* 1,410		Hazard Ratio^a^ (95% CI)	*p-*Value
	Number of Individuals Who Died	Death Rate per 100 Person-Years (95% CI)	Number of Individuals Who Died	Death Rate per 100 Person-Years (95% CI)		
**Circulatory system disease (category I)**	**81**	**1.32 (1.07–1.65)**	**171**	**1.79 (1.54–2.07)**	**0.74 (0.57–0.97)**	**0.03**
Ischemic heart disease (I20–I25)	35	0.57 (0.41–0.80)	77	0.80 (0.64–1.01)	0.71 (0.47–1.06)	0.10
Hypertensive disease (I10–I15)	12	0.20 (0.11–0.35)	21	0.22 (0.14–0.34)	0.89 (0.44–1.80)	0.74
Stroke (I64)	9	0.15 (0.08–0.28)	16	0.17 (0.10–0.27)	0.87 (0.39–1.97)	0.74
**Neoplasm (category C)**	**58**	**0.95 (0.73–1.23)**	**103**	**1.08 (0.89–1.30)**	**0.88 (0.64–1.21)**	**0.42**
Respiratory (C30–C39)	12	0.20 (0.11–0.35)	22	0.23 (0.15–0.35)	0.86 (0.43–1.73)	0.67
Digestive (C15–C26)	16	0.26 (0.16–0.43)	29	0.30 (0.21–0.44)	0.87 (0.47–1.59)	0.64
Gynaecological (C50–C58)	6	0.10 (0.04–0.22)	14	0.15 (0.09–0.25)	0.67 (0.25–1.74)	0.40
**Other and unknown (other categories/unknown)**	**54**	**0.88 (0.68–1.15)**	**106**	**1.11 (0.92–1.34)**	**0.79 (0.57–1.11)**	**0.17**

^a^Hazard ratios are based on Cox proportional-hazards models. Control group is reference group.

In an additional analysis, we compared the survival proportion observed in the present study with that of the general Swiss population of the same age for the same time period. As expected—because individuals with disabilities, terminal disease, and dementia were excluded from the present study population—survival in the general population (69.0%, 95% CI 68.9%–69.1%) was somewhat lower than survival in the control group (72.8%) (S1 Fig).

In addition, we conducted an a priori planned subgroup analysis according to the baseline Pra risk score [25] of study participants (high risk for adverse health outcomes defined as Pra score ≥ 0.286). In the low-risk subgroup, yearly mortality rates were low (intervention group, 1.98%; control group, 2.23%), with a hazard ratio for death of 0.89 (95% CI 0.67–1.18, *p* = 0.42; based on Wald test from Cox regression model). The yearly mortality rates were high among participants in the high-risk subgroup (intervention group, 4.99%; control group, 6.67%), with a hazard ratio for death of 0.74 (95% CI 0.59–0.92, *p* = 0.007; based on Wald test from Cox regression model). A Cox regression analysis including a treatment–subgroup interaction term revealed that there was no statistically significant interaction between group assignment (intervention versus control) and the two pre-specified subgroups (low and high baseline risk) (*p* = 0.32), demonstrating that the relative survival effects of the intervention did not differ between the low- and high-risk subgroups.

### Cost of the Intervention

The cost of providing the full intervention over the 2-y period, based on 2014 costs for personnel and overhead in Switzerland, was US$1,017 per participant. The majority of the costs were related to the time and expenses of the involved health professionals. Only a small amount (US$56) was spent on generating and administering the HRA-O questionnaires and feedback reports (S11 Table).

## Discussion

In this study we evaluated the long-term effects of a collaborative model of care based on HRA in older individuals as compared to usual care. After 8 y, mortality was significantly lower in individuals receiving the intervention than in individuals in the control group. The early detection and successful modification of risk factors for functional status decline identified with the HRA-based intervention and the improvement in recommended preventive care use likely explained this reduction in mortality. In fact, 2-y follow-up confirmed that the intervention group had more favourable health behaviours and used preventive care services more frequently than individuals in the control group. In addition, it is likely the intervention also had other favourable effects contributing to the survival effect, such as early interventions for health and functional impairments uncovered with the HRA system, or improved management of chronic conditions (e.g., hypertension, diabetes) with the nurse counselling integrated into the process of primary care.

A main strength of this study is the randomised controlled design with an intention-to-treat analysis and fully available long-term survival data on all study participants. Also, the study was conducted in a “real world” setting, with a study population consisting of older individuals registered in PCP practices, and not of a selected group of individuals highly motivated to receive preventive care. It is unlikely that the study overestimates the survival effect of the intervention; on the contrary, it may underestimate the effect for several reasons. First, PCPs received training and gained experience in preventive care, which likely resulted in improved care for individuals in the control group (possible contamination effect). Second, a proportion (14.4%) of participants allocated to the intervention group did not complete the HRA-O questionnaire at baseline, and were therefore not offered the intervention as planned during the 2-y follow-up period. With the intention-to-treat design, the present study might therefore underestimate treatment effects for individuals adhering to the intervention. Finally, an intervention continued over the 8 y of the survival follow-up period likely would have had stronger effects than the intervention limited to a 2-y period, as tested in this study.

An important question is whether the finding of an approximate 20% reduction in mortality is plausible and consistent with previous findings in the literature. There is no previous research on long-term outcomes of HRA for comparison. However, multiple studies have attempted to evaluate the potential effect of risk factor modification on reduction of all-cause mortality. A recent meta-analysis of influenza vaccination studies concluded that even after adjustment for potential bias, the odds ratio for all-cause mortality was 0.60 (i.e., an approximately 40% reduction of mortality) for vaccinated compared to non-vaccinated individuals in years when the vaccine matched the circulating virus [37]. A pooled analysis of population-based cohort studies demonstrated that physical activity is related to a 20% to 37% reduction in mortality among adults, with a dose–response association [38]. A systematic analysis of prospective studies on the combined effects of health lifestyle behaviours showed an estimated 66% reduction in all-cause mortality if four healthy risk factors were compared with four unhealthy risk factors [39]. A study of cardiovascular risk factors found that the adjusted hazard ratio for all-cause mortality was 0.49 (95% CI 0.33–0.74) for participants with six or more versus one or fewer favourable cardiovascular health metrics [40]. Overall, these recent analyses, although mostly based on non-randomised prospective studies, demonstrate that a 20% reduction in mortality, as observed in our study, is in the expected range for an intervention modifying health behaviours and preventive care use.

The present study has several limitations. It was conducted at one single site. However, extensive preparatory work and field tests in the US, Germany, and the UK confirmed that the intervention used in our trial is well accepted and feasible for use in other regions [12–14,22]. A further limitation is the fact that the intervention phase of this study took place more than a decade ago (between 2000 and 2004): publication of long-term outcome data was possible only after long-term outcome data became fully available in 2014. However, the study findings are relevant today since most risk factors and key recommendations have remained unchanged since 2004. An additional limitation is the use of a brief self-report questionnaire for measuring health behaviour outcomes at 2-y follow-up. This approach contributed to a high response rate, but it may overestimate prevalence rates of favourable health behaviours and does not measure effects on the multiple other risk factors for functional status decline that were measured with the baseline HRA-O questionnaire. Also, the fact that we did not collect extensive baseline information among control group individuals limits our ability to analyse in detail intervention effects on HRA-O-based risk factors. In addition, the use of self-report information for the 2-y follow-up outcomes may lead to socially desirable answers and therefore overestimate the prevalence of favourable outcomes. However, since outcome assessment was blinded for group allocation, it is unlikely that this resulted in a bias between the intervention and control groups. Another limitation is the lack of information on specifically which changes in risk behaviours and clinical preventive care use made the biggest contribution to reduced mortality in this multifactorial trial. A further limitation is the validity of cause of death information, which relies on information coded by different attending physicians.

Our study did not evaluate long-term effects on functional status, quality of life, or actual cost-effectiveness, and did not disentangle which components of the complex intervention tested in this trial were most efficacious. Future studies should address these issues and, in addition, examine the generalisability of the benefits observed in this study to other settings and refine the HRA-O-based intervention to further increase its efficiency and effectiveness. For example, practice-based instead of home-based counselling, use of other forms of reinforcement such as Internet or mobile communication, use of behaviour change techniques (e.g., pedometer step-count and accelerometer) as part of counselling [41], or repetitive group sessions might be effective alternatives or add-ons to the preventive care home visits by nurse counsellors.

### Conclusion

Many previous studies have revealed the importance of multimodal interventions and coordination of care in disabled or demented older individuals. In contrast, the HRA-based approach tested in the present study was designed for the approximate 80% of the older population without pre-existing disability. The findings of this trial have important implications for policy and practice. Several countries have introduced multimodal preventive care programmes available to healthy older individuals, and are challenged to decide whether, and if so how, these programmes should be continued. For example, the US introduced the Welcome to Medicare and Annual Wellness Visit programs for Medicare beneficiaries [42]. The favourable results of our study support that implementation should be based on a multidimensional HRA system with adequate personalised reinforcement.

For practice implementation, a key factor for success is ensuring personal reinforcement of HRA-based recommendations by specially trained counsellors who take into account individuals’ personal preferences. To ensure synergies with primary care, regionally adapted approaches for integrating HRA into primary care need to be developed. This integration is facilitated by the use of HRA as a comprehensive self-administered tool for initial assessment, the availability of automatically generated, regionally adapted feedback reports, and delegation of health counselling to specially trained health professionals. Our study may also serve as a model for low- and middle-income countries, given the importance of the demographic challenge of rapidly growing populations of older individuals in these countries [43]. Regionally adapted HRA-O approaches might reach large groups of older individuals at relatively low cost.

## Supporting Information

S1 Fig Comparison of 8-y survival in study population with survival in general Swiss population aged 65 y and older in the same time period. The data for the general population is based on the Swiss National Cohort; mean age of the population was 74.8 ± 6.9 (SD) y, with 58.1% women in the census in 2000. Analysis is based on Kaplan-Meier estimates of survival.
(TIFF)

S1 Table Definitions of risk factors and sources of instruments included in the HRA-O questionnaire. (PDF)

S2 Table Intention to change health behaviours among study participants in the intervention group at baseline. (PDF)

S3 Table Prevalence rates of deficits in recommended preventive care use among study participants in the intervention group at baseline. (PDF)

S4 Table Reasons for not having used recommended preventive care among study participants in the intervention group at baseline. (PDF)

S5 Table Survey among primary care physicians after completion of the intervention. (PDF)

S6 Table Missing values of primary outcomes at 2-y follow-up. (PDF)

S7 Table Primary outcomes at 2-y follow-up: sensitivity analysis based on complete case dataset (without imputed data). (PDF)

S8 Table Secondary outcomes at 2-y follow-up: self-reported information. (PDF)

S9 Table Secondary outcomes at 2-y follow-up: individuals permanently admitted to nursing homes. (PDF)

S10 Table Survival analyses: sensitivity analyses with adjustment for selected individual baseline variables. (PDF)

S11 Table Estimation of costs for providing the intervention. (PDF)

S1 Text PRO-AGE Solothurn study protocol and statistical analysis plan. (PDF)

S2 Text CONSORT 2010 checklist. (PDF)

S3 Text HRA-O questionnaire (UK English version). (PDF)

S4 Text HRA-O questionnaire (German language version). (PDF)

S5 Text PRO-AGE intervention manual (English version). (PDF)

S6 Text PRO-AGE Solothurn intervention manual (German language version). (PDF)

S7 Text Sensitivity analysis of 2-y outcome results accounting for attrition. (PDF)

## Abbreviations

HRA: health risk assessment
HRA-O: Health Risk Assessment for Older Persons
ICD–10: International Classification of Diseases–Tenth Revision
PCP: primary care physician
PRO-AGE: Prevention in Older People–Assessment in Generalists’ Practices
SD: standard deviation
