# Supplementary figures and images for: Effect of Health Risk Assessment and Counselling on Health Behaviour and Survival in Older People: A Pragmatic Randomised Trial
Source: PLoS Med. 2015 Oct 19;12(10):e1001889. doi: 10.1371/journal.pmed.1001889 (PMC4610679; doi:10.1371/journal.pmed.1001889)

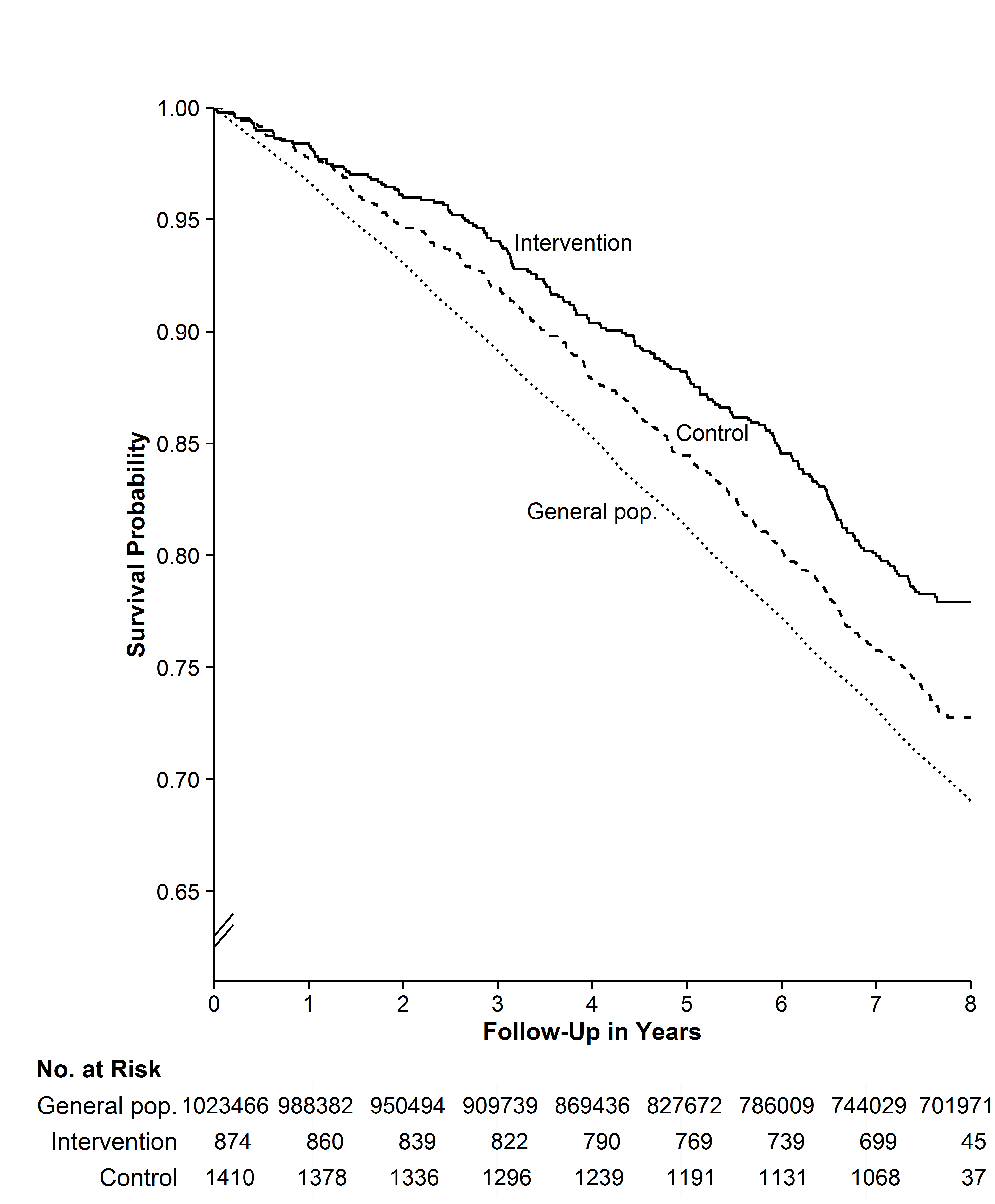

Supplement: S1 Fig — The data for the general population is based on the Swiss National Cohort; mean age of the population was 74.8 ± 6.9 (SD) y, with 58.1% women in the census in 2000. Analysis is based on Kaplan-Meier estimates of survival. (TIFF) [file pmed.1001889.s001.tiff]
