# Supplementary material for: Effect of Health Risk Assessment and Counselling on Health Behaviour and Survival in Older People: A Pragmatic Randomised Trial
Source: PLoS Med. 2015 Oct 19;12(10):e1001889. doi: 10.1371/journal.pmed.1001889 (PMC4610679; doi:10.1371/journal.pmed.1001889)
Supplement: S1 Table — (PDF) [file pmed.1001889.s002.pdf]

Table S1. Definitions of Risk Factors and Sources of Instruments Included in the HRA-O Questionnaire.[a]

Risk Factor Domains (Alphabetical Order)	Instruments Used for Risk Assessment[d]	Definition of Risk
1. Accident prevention	Use of seatbelt [National Center for Chronic Disease Prevention and Health Promotion, 1993]	Does not always wear a seat belt
2. Activities of daily living	Instrumental Activities of Daily Living (IADL) [Lawton and Brody, 1969]	Difficulty/ need for human assistance in ≥ 2 items of instrumental activities of daily living (IADL)
	Item of Preclinical Mobility Disability [Fried et al., 2000]	Changed kind of mobility-related activity in past 12 months
	Item of Preclinical Mobility Disability [Fried et al., 2000]	Decreased frequency of mobility-related activity in past 12 months
2. Alcohol use	Alcohol Use Disorders Identification Test [Babor et al., 1992]	Drinking more than age- and gender-specific limits of quantity and frequency of alcohol[b]
3. Falls	Study of Osteoporotic Fractures Research Group Survey [Kelsey et al., 1992]	Repeated (≥1) falls in past 12 months
	Fear of falling [Tinetti et al., 1988]	Self-reported limitation of activities due to fear of falling
5. Health status	Self-perceived health status [Human Population Laboratory, 1965]	Self-perceived health status “fair” or “poor”
6. Hearing	Hearing Handicap Inventory for the Elderly [Lichtenstein et al., 1988]	Impaired hearing
7. Incontinence	Urinary incontinence (Medical, Epidemiological and Social Aspects of Aging Project Questionnaire) [Diokno et al., 1986]	Urinary incontinence on >5 days in past 12 months
8. Medication use	Use of medications [Breslow et al., 1997]	Use of ≥ 4 medications
	Inappropriate medication use [Beers, 1997]	Use of long-acting benzodiazepine or amitriptyline
	Medication compliance [Breslow et al., 1997]	Self-reported medication side effects
	Questionnaire on Drug-Related Symptoms in Elderly Outpatients [Wasson et al., 1992]	Possible prescribed medication adverse reaction
9. Medical history	Chronic Conditions [Human Population Laboratory, 1965]	Presence of ≥ 3 chronic condition(s)
10. Memory	Memory Self Report [Riege, 1982]	Memory problems
11. Mood	5-item Mental Health Inventory Screening Test [Stewart et al., 1988]	Depressive mood

Table S1-continued. Definitions of Risk Factors and Sources of Instruments Included in the HRA-O Questionnaire.[a]

Risk Factor Domains (Alphabetical Order)	Instruments Used for Risk Assessment[§]	Definition of Risk
12. Nutrition	Self-reported height and weight	Body mass index <20 kg/m^2
	Self-reported height and weight	Body mass index ≥ 27 kg/m^2
	Self-reported weight loss of ≥ 5 kg in past 6 months	Loss of weight (≥ 5kg in past 6 months)
	CRISP (Cholesterol Reduction in Seniors Program) Fat Food Screening Questionnaire [Stoy et al., 1995]	Consumption of >2 high fat food items per day
	CRISP (Cholesterol Reduction in Seniors Program) Plant Food Screening Questionnaire [Stoy et al., 1995]	Consumption of <5 fruit/ fiber items per day
13. Oral health	Geriatric Oral Health Assessment Index [Atchison and Dolan, 1990]	Oral health problem
14. Pain	Geriatric Pain Measure [Ferrell et al., 2000]	Presence of moderate to severe pain
15. Physical activity[c]	PASE (Physical Activity Scale for the Elderly) [Washburn et al., 1993]	Moderate or strenuous physical activity <5 times/ week
16. Social factors	Medical Outcomes Study Social Support Survey [Sherbourne and Stewart, 1991]	Low level of emotional support
	Lubben Social Network Scale [Lubben et al., 1988]	High risk of social isolation
	Subscale Lubben Social Network Scale [Lubben et al., 1988]	Marginal family ties
	Subscale Lubben Social Network Scale [Lubben et al., 1988]	Marginal friendship ties
	Single-item question [Berkman and Syme, 1979]	No participation in social groups or organizations
17. Tobacco use	Tobacco Use Questionnaire [Breslow et al., 1997]	Current tobacco use
18. Vision	Visual Functioning Questionnaire [Mangione et al., 1998]	Problem in ≥ 1 vision sub-domains

[a] HRA-O denotes Health Risk Appraisal for Older Persons (for full description of HRA-O questionnaire and sample reports see Study Protocol (supplementary material).

[b] Risk Possible misuse of alcohol was defined as drinking more than age- and gender-specific limits of quantity and frequency of alcohol (men < 70 years >14 drinks per week, men >70 years >11 drinks per week, women <70 years >11 drinks per week, women >70 years >8 drinks per week), or as meeting the criteria of binge drinking (>4 drinks at one occasion monthly or more frequently).

[c] Based on participant self-reported number of days with moderate or strenuous level of physical activity.

Table S1-continued. Definitions of Risk Factors and Sources of Instruments Included in the HRA-O Questionnaire.[a]

[d] References (in alphabetical order):

Atchison KA, Dolan TA. Development of the Geriatric Oral Health Assessment Index. *J Dent Education.* 1990;54(11):680-687.

Babor TF, de la Fuente JR, Saunders J, Grant M: AUDIT – The Alcohol Use Disorders Identification Test: guidelines for use in primary health care. Geneva: World Health Organization; 1992.http://whqlibdoc.who.int/hq/1992/who_psa_92.4.pdf (Accessed March 16, 2015).

Beers MH. Explicit criteria for determining potentially inappropriate medication use by the elderly. *Arch Intern Med.* 1997;157(14):1531-1536.

Berkman LF, Syme SL. Social networks, host resistance, and mortality: A nine-year follow-up study of Alameda County residents. *Am J Epidemiol*. 1979;109(2):186-204.

Breslow L, Beck JC, Morgenstern H, Fielding JE, Moore AA, Carmel M, Higa J. Development of a health risk appraisal for the elderly (HRA-E). *Am J Health Promot*. 1997;11(5):337-343.

Diokno AC, Brock BM, Brown MB, Herzog AR. Prevalence of urinary incontinence and other urological symptoms in the noninstitutionalized elderly. *J Urol*. 1986;136(5):1022-1025.

Ferrell BA, Stein WM, Beck JC. The Geriatric Pain Measure: Validity, reliability and factor analysis. *J Am Geriatr Soc*. 2000;48(12):1669-1673.

Fried LP, Bandeen-Roche K, Chaves PH, Johnson BA. Preclinical mobility disability predicts incident mobility disability in older women. *J Gerontol.* 2000;55(1):M43-M52.

Human Population Laboratory. Health and Ways of Living, Human Population Laboratory (HPL) 1965 Men's Form.

Kelsey JL, Browner WS, Seeley DG, Nevitt MC, Cummings SR. Risk factors for fractures of the distal forearm and proximal humerus. *Am J Epidemiol*. 1992;135(5):477-489.

Lawton MP, Brody EM. Assessment of older people: Self-maintaining instrumental activities of daily living. *Gerontologist*. 1969;9(3):179-186.

Lichtenstein MJ, Bess FH, Logan SA. Validation of screening tools for identifying hearing-impaired elderly in primary care. *JAMA*. 1988;259(19):2875-2878.

Lubben JE. Assessing social networks among elderly populations. *Fam Comm Health*. 1988;11(3):42-52.

Mangione CM, Lee PP, Pitts J, Gutierrez P, Berry S, Hays RD. Psychometric properties of the National Eye Institute Visual Function Questionnaire (NEI-VFQ). *Arch Ophthalmol*. 1998;116(11):1496-1504.

National Center for Chronic Disease Prevention and Health Promotion. Behavioral Risk Factor Survey. Department of Health and Human Services, 1993.

Riege WH. Self-report and tests of memory aging. *Clin Gerontol*. 1982;1(2):23-36.

Sherbourne CD, Stewart AL. The MOS social support survey. *Soc Sci Med*. 1991;32(6):705-714.

Stewart AL, Hays RD, Ware JE. The MOS short-form general health survey. Reliability and validity in a patient population. *Med Care*. 1988;26(7):724-732.

Stoy DB, Curtis RC, Dameworth KS, Dowdy AA, Hegland J, Levin JA, Sousoulas BG. The successful recruitment of elderly black subjects in a clinical trial: the CRISP experience. Cholesterol Reduction in Seniors Program. *J Natl Med Assoc.* 1995;87(4):280-287.

Tinetti ME, Speechley M, Ginter SF. Risk factors for falls among elderly persons living in the community. *N Engl J Med*. 1988; 319(26):1701-1707.

Washburn RA, Smith KW, Jette AM, Janney CA. The Physical Activity Scale for the Elderly (PASE): development and evaluation. *J Clin Epidemiol*. 1993;46(2):153-162.

Wasson J, Nierenberg D, Landgraf J, Whaley F, Malenka, D. Johnson D, Keller A, Dartmouth Primary Care COOP The effect of a patient questionnaire on drug-related symptoms in elderly outpatients. *Ann Rev Geriatr Gerontol*. 1992(12):109-125.
