# Supplementary material for: Effect of Health Risk Assessment and Counselling on Health Behaviour and Survival in Older People: A Pragmatic Randomised Trial
Source: PLoS Med. 2015 Oct 19;12(10):e1001889. doi: 10.1371/journal.pmed.1001889 (PMC4610679; doi:10.1371/journal.pmed.1001889)
Supplement: S2 Table — (PDF) [file pmed.1001889.s003.pdf]

Table S2. Intention to Change Health Behaviour among Study Participants of the Intervention Group at Baseline.[a]

Definition of Subgroup at Risk	Description of Question	Answer Categories	No./ Total	%
Persons with low level of physical activity (<5 times/ week moderate or strenuous physical activity according to PASE questionnaire)[b] (n=524)	Intention to increase physical activity	Plans to take steps in next month	7/524	1.3
		Plans to take steps in the next 6 months	1/524	0.2
		No plans within next 6 months	516/524	98.5
	Reasons for not increasing physical activity	Already frequent and regular exercise	331/524	63.2
		Pain with physical activity	88/524	16.8
		Illness limiting physical activity	69/524	13.2
		A physical limitation	35/524	6.7
Consumption of >2 high fat food items per day according to CRISP fat food questionnaire[b] (n=354)	Intention to decrease high fat intake	Plans to take steps in next month	3/354	0.8
		Plans to take steps in the next 6 months	3/354	0.8
		No plans within next 6 months	348/354	98.3
	Reasons for not decreasing high fat intake	Is already minimizing fat intake	239/354	67.5
		Does not think it is important to eat less fat	48/354	13.6
		Likes the taste of high-fat foods	33/354	9.3
		Has trouble to shop/ prepare low-fat foods	27/354	7.6
Consumption of < 5 fruit/ fibre items per day according to CRISP plant food questionnaire[b] (n=489)	Intention to increase fruit/fibre intake.	Plans to take steps in next month	1/489	0.2
		Plans to take steps in the next 6 months	1/489	0.2
		No plans within next 6 months	487/489	99.6
	Reasons for not increasing low fruit/fiber intake	Already eats plenty of fruits/ vegetables	458/489	93.7
Current tobacco use (n=86)	Intention to change current tobacco	Plans to quit smoking in next month	12/86	14.0
		Plans to quit smoking in next 6 months	10/86	11.6
		No plans within next 6 months	64/86	74.4

[a] HRA-O denotes Health Risk Appraisal for Older Persons. Results based on self-report answers.
[b] For references, see Table S1.
