# Supplementary material for: Effect of Health Risk Assessment and Counselling on Health Behaviour and Survival in Older People: A Pragmatic Randomised Trial
Source: PLoS Med. 2015 Oct 19;12(10):e1001889. doi: 10.1371/journal.pmed.1001889 (PMC4610679; doi:10.1371/journal.pmed.1001889)
Supplement: S3 Table — (PDF) [file pmed.1001889.s004.pdf]

Table S3. Prevalence Rates of Deficits in Recommended Preventive Care Use among Study Participants of the Intervention Group at Baseline (n=748).[a]

Setting	Definition	No. (%).
Preventive care usually performed in PCP setting	No blood glucose measurement in past 3 years	172 (30.0)
	No blood pressure measurement in past year	35 (4.7)
	No cholesterol measurement in past 5 years and age <75 yr	99 (13.2)
	No faecal occult blood measurement in past year and age <80 yr	395 (52.8)
	No influenza vaccination in past year	395 (52.8)
	No pneumococcal vaccination (ever)	639 (85.4)
Preventive care usually performed in specialist setting	No cervical smear in past 3 years (women)	244 (32.6)
	No dental check in past year	306 (40.9)
	No hearing check-up in past year	473 (63.2)
	Women without mammography in past 2 years and age <70 yr	72 (9.6)
	No vision check-up in past year	280 (37.4)

[a] Based on the 748 of the 874 participants allocated to the intervention group who returned the baseline HRA -O (Health Risk Appraisal for Older Persons) questionnaire. PCP denotes primary care physician. The denominator includes participants with incomplete or missing self-report on individual preventive care items (number of participants with missing information is between 17 and 48).
