# Supplementary material for: Effect of Health Risk Assessment and Counselling on Health Behaviour and Survival in Older People: A Pragmatic Randomised Trial
Source: PLoS Med. 2015 Oct 19;12(10):e1001889. doi: 10.1371/journal.pmed.1001889 (PMC4610679; doi:10.1371/journal.pmed.1001889)
Supplement: S4 Table — (PDF) [file pmed.1001889.s005.pdf]

Table S4. Reasons for Not Having Used Recommended Preventive Care Among Study Participants of the Intervention Group at Baseline. (N=731).[a]

Main Self-Reported Reason	Answer Categories	No. (%)
Does not see a need	Does not see a need/ does not think it is important / never recommended by PCP/ never thought about it	586 (80.2)
Financial barrier	Financial reason (cost, insurance)	13 (1.8)
Time constraint	Lack of time	1 (0.1)
Does not give a specific reason	No specific reason indicated/ no answer	131 (17.9)

[a] Based on self-report information of participants allocated to the intervention group. PCP denotes primary care physician. The denominator only includes persons with ≥ 1 deficits in preventive care at baseline.
