# Supplementary material for: Effect of Health Risk Assessment and Counselling on Health Behaviour and Survival in Older People: A Pragmatic Randomised Trial
Source: PLoS Med. 2015 Oct 19;12(10):e1001889. doi: 10.1371/journal.pmed.1001889 (PMC4610679; doi:10.1371/journal.pmed.1001889)
Supplement: S5 Table — (PDF) [file pmed.1001889.s006.pdf]

Table S5. Survey among Primary Care Physicians (PCPs) after Completion of the Intervention (N=16).[a]

A. PCP-Perceived Strength of Evidence[b] for Supporting Preventive Care Recommendations

**Recommendation**	**PCP-Perceived Strength of Evidence**	
	very strong/ relatively strong	*relatively weak/ very weak*
Yearly blood pressure measurement, No. (%)	14 (87.5)	2 (12.4)
5-yearly cholesterol measurement, No. (%)	10 (62.5)	6 (37.5)
3-yearly blood glucose measurement, No. (%)	13 (81.3)	3 (18.7)
Yearly influenza vaccination, No. (%)	16 (100.0)	0 (0.0)
Pneumococcal vaccination (once) , No. (%)	9 (56.3)	7 (43.8)
Yearly faecal occult blood test, No. (%)	6 (37.5)	10 (62.5)

B. PCP-Perceived Impact of Resource Constraints[c] Making it Difficult to Implement Preventive Care Recommendations.

**Recommendation**	**PCP-Perceived Impact of Resource Constraints**	
	significant/ some constraints	*no constraints*
Yearly blood pressure measurement, No. (%)	0 (0.0)	16 (100.0)
5-yearly cholesterol measurement, No. (%)	2 (12.5)	14 (87.5)
3-yearly blood glucose measurement, No. (%)	0 (0.0)	16 (100.0)
Yearly influenza vaccination, No. (%)	1 (6.3)	15 (93.7)
Pneumococcal vaccination (once) , No. (%)	4 (25.0)	12 (75.0)
Yearly faecal occult blood test, No. (%)	1 (6.3)	15 (93.7)

[a] Sixteen of the 19 PCPs (primary care physicians) included in this study agreed to complete an anonymized self-report questionnaire after completion of the intervention at two-year follow-up.

[b] For each of the recommendations the survey question was: “Please rate the strength of evidence for supporting the recommendation by circling the most appropriate description.”

[c] For each of the recommendations the survey question was: “Which of the following preventive measures are difficult to provide in routine clinical practice because of limited resources? Circle the answer that best describes the impact of resource constraints in your practice.”
