# Supplementary material for: Effect of Health Risk Assessment and Counselling on Health Behaviour and Survival in Older People: A Pragmatic Randomised Trial
Source: PLoS Med. 2015 Oct 19;12(10):e1001889. doi: 10.1371/journal.pmed.1001889 (PMC4610679; doi:10.1371/journal.pmed.1001889)
Supplement: S6 Table — (PDF) [file pmed.1001889.s007.pdf]

Supporting Table S6. Missing Values of Primary Outcomes at 2-Year Follow-up.

Outcome	Intervention Group	Control Group
	No./ Total (%)[a]	
**Health behaviours**		
Medium to high level of physical activity (≥ 30 minutes per day)[d]	108 (13.1)	159 (12.1)
Medium to high level of fruit/ vegetable/ fiber intake (≥ 2 portions per day)	100 (12.1)	146 (11.1)
Low level of fat intake (< 2 portions of high fat items per day)	93 (11.3)	135 (10.2)
Use of seat belt (always use of seat belt)	98 (11.9)	126 (9.6)
No tobacco consumption	97 (11.7)	131 (9.9)
No or little alcohol use (≤ 1 alcoholic drink per day)		
**Adherence with selected preventive care recommendations**		
Blood pressure measurement in past y	61 (7.4)	110 (8.3)
Cholesterol measurement (persons aged <75 y) in past 5 y [b]	38 (7.9)	62 (7.9)
Glucose measurement in past 3 y	61 (7.4)	123 (9.3)
Influenza vaccination in past y	76 (9.2)	126 (9.6)
Pneumococcal vaccination (ever)	95 (11.5)	169 (12.8)
Faecal occult blood test in past y (persons aged <80 y) [c]	74 (10.9)	113 (10.4)

[a] Calculated from the difference of all available participants surviving in the community (intervention group, n=827; control group n=1320) and the complete case denominator of Table S6.
[b] Denominator includes persons aged <75 years only: intervention group, n=482; control group, n=784.
[c] Denominator includes persons aged <80 years only: intervention group, n=680; control group, n=1089.
