# Supplementary material for: Effect of Health Risk Assessment and Counselling on Health Behaviour and Survival in Older People: A Pragmatic Randomised Trial
Source: PLoS Med. 2015 Oct 19;12(10):e1001889. doi: 10.1371/journal.pmed.1001889 (PMC4610679; doi:10.1371/journal.pmed.1001889)
Supplement: S7 Table — (PDF) [file pmed.1001889.s008.pdf]

Table S7. Primary Outcomes at 2-Year Follow-up: Sensitivity Analysis Based on Complete Case Dataset (Without Imputed Data).[a]

Outcome	Intervention Group	Control Group	Odds Ratio (95% CI)[c]	*P* Value
	No./ Total (%)[b]			
**Health behaviours**				
Medium to high level of physical activity (≥ 30 minutes per day)[d]	519/719 (72.2)	731/1161 (63.0)	1.53 (1.24–1.90)	<0.001
Medium to high level of fruit/ vegetable/ fiber intake (≥ 2 portions per day)	346/727 (47.6)	456/1174 (38.8)	1.46 (1.19–1.78)	<0.001
Low level of fat intake (< 2 portions of high fat items per day)	225/734 (30.7)	297/1185 (25.1)	1.40 (1.12–1.74)	0.003
Use of seat belt (always use of seat belt)	650/729 (89.2)	1011/1194 (84.7)	1.48 (1.10–1.98)	0.009
No tobacco consumption	660/730 (90.4)	1066/1189 (89.7)	1.07 (0.78–1.48)	0.66
No or little alcohol use (≤ 1 alcoholic drink per day)	685/733 (93.5)	1072/1191 (90.0)	1.61 (1.13–2.30)	0.008
**Adherence with selected preventive care recommendations[e]**				
Blood pressure measurement in past y	705/766 (92.0)	1069/1210 (88.3)	1.52 (1.11–2.10)	0.009
Cholesterol measurement (persons aged <75 y) in past 5 y	400/444 (90.1)	624/722 (86.4)	1.43 (0.98–2.07)	0.06
Glucose measurement in past 3 y	622/766 (81.2)	925/1197 (77.3)	1.28 (1.02–1.61)	0.04
Influenza vaccination in past y	496/751 (66.1)	707/1194 (59.2)	1.36 (1.11–1.68)	0.003
Pneumococcal vaccination (ever)	225/732 (30.7)	221/1151 (19.2)	2.00 (1.59–2.51)	<0.001
Faecal occult blood test in past y (persons aged <80 y)	167/606 (27.6)	205/976 (21.0)	1.45 (1.14–1.85)	0.003

[a] CI denotes confidence interval.
[b] Total is the number of persons with available data per outcome. One reason for the variable denominators is the variable definition of the target participant group for cholesterol measurement and faecal occult blood test. For cholesterol measurement, the target group was persons aged <75 yr, and for fecal occult blood testing, the target group was persons aged <80 yr, respectively. The other reason for the variable denominators is different numbers of missing data per outcome. An example: The denominator for the physical activity outcome in the intervention group is 719. As indicated in the flow diagram (Fig 1), 779 of 827 surviving persons in the intervention group answered the 2-yr follow-up questionnaire. Among the 779 person, 60 did not respond to the physical activity question, leaving 719 persons with complete data on physical activity at the 2-yr follow-up.
[c] Control group is reference group.
[d] Based on participant self-reported answers to average daily duration of moderate or strenuous level of physical activity.
[e] Based on abstraction of primary care physicians' patient charts.
