# Supplementary material for: Effect of Health Risk Assessment and Counselling on Health Behaviour and Survival in Older People: A Pragmatic Randomised Trial
Source: PLoS Med. 2015 Oct 19;12(10):e1001889. doi: 10.1371/journal.pmed.1001889 (PMC4610679; doi:10.1371/journal.pmed.1001889)
Supplement: S8 Table — (PDF) [file pmed.1001889.s009.pdf]

Table S8. Secondary Outcomes at 2-Year Follow-up: Self-Reported Information.[a]

Outcome	Intervention Group	Control Group	Odds Ratio (95% CI)	*P* Value
**Main Analysis with Imputed Data**[b]				
**Self-perceived health**			n.a.	0.04[c]
Excellent, No. / Total (%)	14/827 (1.7)	12/1320 (0.9)		
Very good, No. / Total (%)	121/827 (14.6)	159/1320 (12.0)		
Good, No. / Total (%)	548/827 (66.3)	856/1320 (64.8)		
Fair, No. / Total (%)	136/827 (16.4)	267/1320 (20.2)		
Poor, No. / Total (%)	8/827 (1.0)	26/1320 (2.0)		
**Self-reported basic activities of daily living**				
Need for human assistance, No. / Total (%)	38/827 (4.6)	62/1320 (4.7)	0.97 (0.61–1.54)	0.91
**Sensitivity Analysis with Complete Case Dataset (No Imputed Data)**				
**Self-perceived health**			n.a.	0.04‡
Excellent, No. / Total (%)	13/764 (1.7)	12/1215 (1.0)		
Very good, No. / Total (%)	114/764 (14.9)	148/1215 (12.2)		
Good, No. / Total (%)	507/764 (66.4)	790/1215 (65.0)		
Fair, No. / Total (%)	123/764 (16.1)	242/1215 (19.9)		
Poor, No. / Total (%)	7/764 (0.9)	23/1215 (1.9)		
**Self-reported basic activities of daily living**				
Need for human assistance, No/ Total (%)	32/763 (4.2)	53/1212 (4.4)	0.94 (0.60-1.49)	0.81

[a] CI denotes confidence interval; n.a. not applicable. Odds Ratio based on logistic general estimation equation (GEE) model adjusted for cluster household. Control group is reference group.
[b] Missing information was imputed for the analyses, using a multiple imputation technique.
[c] *P* Value from overall test using logistic regression adjusted for cluster household.
