# Supplementary material for: Effect of Health Risk Assessment and Counselling on Health Behaviour and Survival in Older People: A Pragmatic Randomised Trial
Source: PLoS Med. 2015 Oct 19;12(10):e1001889. doi: 10.1371/journal.pmed.1001889 (PMC4610679; doi:10.1371/journal.pmed.1001889)
Supplement: S9 Table — (PDF) [file pmed.1001889.s010.pdf]

Table S9. Secondary Outcomes at 2-Year Follow-up: Persons Permanently Admitted to Nursing Home.[a]

Outcome	Intervention Group (n=874)	Control Group (n=1410)	Odds Ratio (95% CI)[b]	*P* Value[c]
**Nursing home admission**				
Nursing home admission, No./ Total (%)	12/831 (1.4)	26/1338 (1.9)	0.74 (0.37-1.48)	0.39

[a] This Table does not include imputed data. CI denotes confidence interval. Information was missing for persons of practice withdrawn from the project (see Fig 1, for numbers of persons withdrawn). The denominator also includes persons who died within the two-year follow-up period; for these persons we recorded whether they were permanently admitted to a nursing home prior to death.
[b] Odds ratio based on logistic general estimation equation (GEE) model adjusted for cluster household. Control group is reference group.
[c] P Value from overall test using logistic regression adjusted for cluster household with intervention as outcome.
