# Supplementary material for: Effect of Health Risk Assessment and Counselling on Health Behaviour and Survival in Older People: A Pragmatic Randomised Trial
Source: PLoS Med. 2015 Oct 19;12(10):e1001889. doi: 10.1371/journal.pmed.1001889 (PMC4610679; doi:10.1371/journal.pmed.1001889)
Supplement: S10 Table — (PDF) [file pmed.1001889.s011.pdf]

Table S10 Survival Analyeses: Sensitivity Analyses with Adjustment for Selected Individual Base-Line Variables

Table S10A. Survival Analysis Adjusted for Availability of Caregiver at Base-Line.

**Parameter**	**Level**	**Hazard Ratio[a] (95%CI)**	***P* value**
Group allocation	Control	Reference	
	Intervention	0.79 (0.67, 0.94)	0.009
Availability of caregiver if needed	Yes	Reference	
	No	1.18 (0.92, 1.51)	0.20

[a]Hazard ratios from Cox regression model adjusted for household cluster. CI denotes confidence interval.

Table S10B. Survival Analysis Adjusted for Base-Line Self-Perceived Health Status.

**Parameter**	**Level**	**Hazard Ratio* (95%CI)**	***P* value**
Group allocation	Control	Reference	
	Intervention	0.83 (0.70, 0.98)	0.03
Self-perceived health	Excellent	Reference	<0.001[b]
	Very good	1.07 (0.49, 2.35)	
	Good	1.75 (0.84, 3.67)	
	Fair	3.98 (1.89, 8.35)	
	Poor	8.18 (3.09, 21.67)	

[a]Hazard ratios from Cox regression model adjusted for household cluster. CI denotes confidence interval.
[b]p-value from Wald test of composite hypothesis that all self-perceived health levels are equal to zero on log hazard scale
