# Supplementary material for: Effect of Health Risk Assessment and Counselling on Health Behaviour and Survival in Older People: A Pragmatic Randomised Trial
Source: PLoS Med. 2015 Oct 19;12(10):e1001889. doi: 10.1371/journal.pmed.1001889 (PMC4610679; doi:10.1371/journal.pmed.1001889)
Supplement: S11 Table — (PDF) [file pmed.1001889.s012.pdf]

Table S11. Estimation of Costs for Providing the Intervention.[a]

Cost Element	Basis for Calculation	Year 1: Mean Time per Participant (Minutes)	Year 1: Cost per Participant (CHF)	Year 2: Mean Time per Participant (Minutes)	Year 2: Cost per Participant (CHF)
**1. HRA-O questionnaire and reports**	**Administrative intervention costs related to use of the HRA-O system**				
Selecting patients from practice register and generating an address list of patients to be invited for the intervention	Reimbursement given to primary care physicians	n.a.	CHF 2.65	n.a.	CHF 0.00
Mailing of personal invitation with brief questionnaire to participants, and data entry of completed brief questionnaires	Amount charged by service provider	n.a.	CHF 5.90	0.0	CHF 0.00
Mailing of HRA-O questionnaire to participants, return mailing of completed HRA-O questionnaires to service provider, and data entry of completed HRA-O questionnaires	Amount charged by service provider	n.a.	CHF 15.90	n.a.	CHF 15.90
Generating individualized computer-generated HRA-O participant and provider reports, mailing participant reports to participants, and mailing of provider reports to primary care physicians and nurse counsellors	Amount charged by service provider	n.a.	CHF 7.64	n.a.	CHF 7.64
**Subtotal for HRA-O questionnaire and reports**			**CHF 32.09**		**CHF 23.54**
**2. Health professionals**[b]	**Costs for health professionals (nurse counsellors, PCPs, and geriatricians)**				
A. Nurse counsellors					
Home visits	Time of nurse counsellor per participant used for conducting home visits based on intervention records of participants who received the full intervention	105.2 minutes	CHF 97.31	128.1 minutes	CHF 118.49
Travel time	Time of nurse counsellor per participant used for travel (calculated from number of home visits per participant multiplied by estimated average travel time of 15 minutes per visit)	33.9 minutes	CHF 31.36	45.0 minutes	CHF 41.63

Table S11-continued. Estimation of Costs for Providing the Intervention.[a]

Telephone contacts	Time of nurse counsellor per participant used for telephone contacts based on intervention records of participants who received the full intervention	18.6 minutes	CHF 17.21	1.2 minutes	CHF 1.11
Case reviews with geriatricians and primary care physicians	Time of nurse counsellor per participant for conducting case reviews	20.0 minutes	CHF 18.50	20.0 minutes	CHF 18.50
Weekly training sessions with senior nurse counselor	Time of nurse counsellor per participant for participating in weekly training (estimate is 40 one-hour training sessions per year, case load 200 participants per nurse counsellor)	12.0 minutes	CHF 11.10	12.0 minutes	CHF 11.10
Administrative time	Calculated as 60% of time used for counselling and conducting case reviews (includes preparation and documentation)	86.3 minutes	CHF 79.81	89.6 minutes	CHF 82.88
Overhead	25% of nurse counsellor salary costs	n.a.	CHF 63.83	n.a.	CHF 68.43
Travel expenses	Includes travel expenses (number of home visits per participant multiplied by CHF 16.50, based on an average travel distance of 15 km per home visits and part of per diem reimbursement for meals)	n.a.	CHF 37.52	n.a.	CHF 49.50
Cost for support by senior nurse counselor	Cost for senior nurse counsellor for weekly one-hour training (40 one-hour training sessions per year, case load 200 participants per nurse counsellor; cost of senior health counsellor time CHF 60.95/hour plus 25% overhead)	n.a.	CHF 15.24	n.a.	CHF 15.24
Initial one-week training	Total cost per participant for time of nurse counsellor preparing and attending the initial one-week training, and for providing this training (calculated for a two-year employment period, case load of 200 participants)	n.a.	CHF 17.28	n.a.	CHF 17.28

Table S11-continued. Estimation of Costs for Providing the Intervention.[a]

B. PCPs[c]					
Case reviews with nurse counsellors	Time of PCP per participant per study year (estimate)	5.0 minutes	CHF 14.95	5.0 minutes	CHF 14.95
Participation in initial and quarterly training session with geriatrician	Time of PCP back-calculated per participant for participating in initial (2 hour) and quarterly (1/2 hour) training session (case load of 50 participants per PCP)	4.8 minutes	CHF 14.35	2.4 minutes	CHF 7.18
C. Geriatricians					
Case reviews with nurse counsellors	Time of geriatrician per participant per study year (estimate)	15.0 minutes	CHF 25.98	15.0 minutes	CHF 25.98
Training sessions with PCPs and specialist advice to PCPs	Time of geriatrician per participant per study year (estimate)	5.0 minutes	CHF 8.66	5.0 minutes	CHF 8.66
Overhead	25% of geriatrician salary costs	n.a.	CHF 8.66	n.a.	CHF 8.66
**Subtotal for health professionals**		**n.a.**	**CHF 461.76**	**n.a.**	**CHF 489.59**
**3. Total**		**n.a.**	**CHF 493.85**	**n.a.**	**CHF 513.13**

Conversion to USD (Conversion Rate, Mar 16, 2015: 1 CHF [Swiss Franc] = 1.01 USD [U.S. Dollar]).

	**Year 1: Cost per Participant**	**Year 2: Cost per Participant**	**Year 1 + 2: Cost per Participant**
**Subtotal for HRA-O questionnaire and reports**	**USD 32.41**	**USD 23.78**	**USD 56.19**
**Subtotal for health professionals**	**USD 466.38**	**USD 494.49**	**USD 960.87**
**Total**	**USD 498.79**	**USD 518.27**	**USD 1017.06**

[a] HRA-O denotes Health Risk Appraisal for Older Persons, PCP denotes primary care physician, n.a. denotes not applicable.

[b] The following salary costs per one hour health professional time were used: nurse counsellor CHF 55.50; geriatrician CHF 103.90. Costs for conducting the research part of the project, and licensing fees for commercial use of instruments and software are not included in the calculation.

[c] PCP time does not take into account time used for counselling or for implementing the intervention as part of routine clinical care. For cost of PCPs, the reimbursement covered by basic health insurance (2014) for a 5 minute primary care physician consultation was used in this calculation (costs are hypothetical, PCPs did not receive additional reimbursement for implementing the intervention; PCP time for case discussions and training sessions were recognized as part of compulsory continuing medical education
