# Supplementary material for: Effect of Health Risk Assessment and Counselling on Health Behaviour and Survival in Older People: A Pragmatic Randomised Trial
Source: PLoS Med. 2015 Oct 19;12(10):e1001889. doi: 10.1371/journal.pmed.1001889 (PMC4610679; doi:10.1371/journal.pmed.1001889)
Supplement: S1 Text — (PDF) [file pmed.1001889.s013.pdf]

Supporting Information S1 Text
Study Protocol and Statistical Analysis Plan

This document contains the following items:
1. Original protocol, final protocol, summary of changes.
2. Original statistical analysis plan, final statistical analysis plan, summary of changes

for: A Collaborative Care Model of Health Risk Assessment and Counselling in Older Persons: A Randomised Clinical Trial


| | **Page** |
|---|---|
| **Study protocol (PRO-AGE Solothurn Study Plan)** | |
| Original study protocol | 2 |
| Document of changes of study protocol | 21 |
| Final study protocol | 24 |
| | |
| **Statistical analysis plan** | |
| Original statistical analysis plan | 44 |
| Document of changes of statistical analysis plan | 50 |
| Final statistical analysis plan | 53 |

# PRO-AGE Solothurn Study plan
## (Prevention in older persons – assessment in generalists' practices) Version Sept 29, 2000

1. Goal ........ 2
2. Background ........ 3
3. Main Hypotheses of Randomised Study ........ 4
4. Outcome measures ........ 5
5. Sample size calculation ........ 6
6. Overall design of the randomised study (Figure 1) ........ 8
7. Plan for inclusion/ exclusion of subjects for randomised study ........ 10
8. Pre-randomisation questionnaire ........ 11
9. Contamination ........ 12
10. Intervention design (Figure 2) ........ 13
11. Methods to measure the intervention ........ 15
12. Methods to measure the satisfaction of providers ........ 16
13. Data confidentiality ........ 17
14. Statistical analysis ........ 18
15. References ........ 19

# 1. Goal

The ultimate goal of the project is to maintain function and in the mid to long term prevent the onset of disability and to minimise unnecessary service utilisation among persons 65 years of age and older. Health risk appraisal (HRA), i.e. identifying modifiable risk factors for disease and disability, and seeking to minimise their adverse impact, is being widely applied for working age adults. Needed now is a comprehensive HRA for elderly persons (HRA-E) designed to reduce functional impairment, the major health problem among the rapidly growing elder population.

## 2. Background

Verbrugge and Jette (1994) hypothesise that the onset of disability is related to a multiplicity of underlying biological, psychological, social, functional, and environmental risk factors. Based on this model, we hypothesise that effective prevention of disability in older persons should be based on early modification of relevant risk factors. Many studies have shown (e.g., Stewart et al. 1993) that older individuals are often willing to participate in interventions designed to improve their health behaviours or reduce risk behaviours, and, furthermore, that these interventions may result in improvements in their health, functioning and perceptions of well-being. Several studies support the efficacy of preventive interventions to not only change health and safety practices of the elderly, for example by increasing exercise (Buchner et al., 1992), reducing hypertension (Applegate et al., 1994), or improving cardiovascular fitness (Stewart et al., 1993), but also to achieve improvements in health, functional status, and perceived quality of life (Applegate et al., 1994). For example, Wagner et al. (1994) demonstrated that a multicomponent intervention consisting of nurse assessment and follow-up in-home sessions targeting risk factors resulted in significantly lower decline of functional status and significantly lower incidence of falls than subjects who received usual care.

In December 1992 a group of University faculty (United States) began developing a health risk appraisal instrument for the elderly (HRA-E). Their work was supported by the John A. Hartford Foundation, The California Wellness Foundation, and the Health Care Financing Organisation. The products of their work to date include (Breslow et al., 1997): (1) a questionnaire for comprehensive health risk assessment of older persons; (2) software for processing computer-generated, personalised reports to participants and their physicians from the questionnaires, and (3) favourable preliminary findings from testing the questionnaire and software in older subjects. The development of the HRA-E was based on a systematic literature review, a priori selection criteria for selection of domains and items, focus group meetings, and on testing of several intermediate versions. Extensive field testing showed favourable data on acceptance and self-reported behaviour change in a sample of approximately 2000 older Americans

# 3. Main Hypotheses of Randomised Study

**<u>We expect:</u>**

A. The intervention will reduce overall and specific health risk
B. The intervention will improve the older persons' self efficacy
C. The participation rate among older persons will be high (over 70 percent)
D. The intervention will result in a high participation rate.

# 4. Outcome measures

**Health habits**

- Minimal or very limited exercise
- Eating a high fat diet
- Eating a low fibre diet
- Not wearing seat belts
- Drinking alcohol excessively
- Smoking

**Preventive practices**

- Blood pressure measurement
- Vision examination
- Hearing examination
- Dental examination
- Cholesterol measurement
- Blood glucose
- Faecal occult blood test
- Influenza vaccination
- Pneumococcal vaccination
- Mammogram
- Pap smear

**Secondary outcomes**

- BADL
- IADL
- AADL
- preclinical functional status impairment
- self-perceived health status
- self-perceived cognitive function
- depressive mood
- vision (based on Vision Function Questionnaire)
- hearing (based on Hearing Handicap Inventory)
- Self efficacy with regard to own health
- Self efficacy with regard to interaction with physician
- Mortality
- Living location

## 5. Sample size calculation

The sample size calculations are based on expected frequencies of adverse health care behaviour and the expected impact of the intervention on the health behaviour. Table 1 presents the observed yield of the HRA-E in an older American population and the self-reported improvement in health behaviour:

Power estimates are shown in Table 2, by the frequency of the outcome event (1-50%) and the minimum effect to be detected (relative risk 1.1 to 3.0). The planned sample size of 800 subjects per group will yield good power (80% and better) for detecting small-to-moderate effects on both rare and common outcome events, e.g. a relative risk of 1.2 (i.e., a 20 percent increase in favourable health care behaviour) with an outcome frequency of 25%.

**Table 1:** Adverse health habits in 1482 older Americans (aged 55 years and older) and % of subjects improving this behaviour ( 4-month telephone follow-up interview)

| | Number of persons receiving message in HRA-E (N=1482) | % of persons receiving message who improved |
|---|---|---|
| **Adverse health habit** | | |
| Minimal or very limited exercise | 280 | 27 |
| Eating a high fat diet | 902 | 44 |
| Not wearing seatbelts | 75 | 16 |
| Drinking alcohol excessively | 480 | 21 |
| Having depressed mood | 209 | 15 |
| Smoking | 280 | 21 |
| **Tardy preventive behaviour** | | |
| Blood pressure measurement | 51 | 21 |
| Vision examination | 248 | 54 |
| Hearing examination | 774 | 19 |
| Dental examination | 203 | 70 |
| Cholesterol measurement | 105 | 65 |
| Colonoscopy | 377 | 25 |
| Influenza vaccination | 440 | 53 |
| Pneumococcal vaccination | 690 | 24 |
| Mammogram | 52 | 67 |
| Breast examination | 32 | 68 |
| Pap smear | 90 | 52 |

**Table 2:** Estimated power (%) for comparing two groups, by expected frequency of the outcome event and the minimum effect (relative risk) to be detected:

| Outcome frequency (%) | Minimum Relative Risk | | | | | | | | | |
|---|---|---|---|---|---|---|---|---|---|---|
| | 1.1 | 1.2 | 1.3 | 1.4 | 1.5 | 1.6 | 1.7 | 2.0 | 2.5 | 3.0 |
| 1 | 4 | 7 | 11 | 15 | 20 | 25 | 30 | 45 | 66 | 79 |
| 2 | 6 | 11 | 17 | 26 | 35 | 44 | 53 | 74 | 92 | 97 |
| 3 | 7 | 14 | 24 | 36 | 49 | 60 | 70 | 89 | 99 | 99+ |
| 4 | 8 | 17 | 31 | 46 | 61 | 73 | 83 | 96 | 99+ | 99+ |
| 5 | 9 | 21 | 37 | 55 | 71 | 83 | 99 | 99 | 99+ | 99+ |
| 10 | 14 | 38 | 66 | 86 | 95 | 99 | 99+ | 99+ | 99+ | 99+ |
| 15 | 19 | 55 | 85 | 97 | 99+ | 99+ | 99+ | 99+ | 99+ | 99+ |
| 20 | 26 | 70 | 95 | 99+ | 99+ | 99+ | 99+ | 99+ | 99+ | 99+ |
| 25 | 32 | 82 | 99 | 99+ | 99+ | 99+ | 99+ | 99+ | 99+ | 99+ |
| 30 | 40 | 90 | 99+ | 99+ | 99+ | 99+ | 99+ | 99+ | 99+ | 99+ |
| 35 | 48 | 96 | 99+ | 99+ | 99+ | 99+ | 99+ | 99+ | 99+ | 99+ |
| 40 | 57 | 98 | 99+ | 99+ | 99+ | 99+ | 99+ | 99+ | 99+ | 99+ |
| 50 | 74 | 99+ | 99+ | 99+ | 99+ | 99+ | 99+ | 99+ | 99+ | 99+ |

(all power estimates are based on the comparison of two proportions for groups of equal size (N=800), assuming alpha=0.05 (two-sided).

# 6. Overall design of the randomised study (Figure 1)

**<u>Step 1:</u> Select approximately 12 participating general practitioners:**
The required number of physicians must be selected with the goal of recruiting 2400 older persons. For determining the required number of physicians, the following factors must be taken into account:

- number of older persons in practice
- percentage of eligible persons among older persons
- percentage of refusal to participate among eligible older persons

The goal is to achieve a sample of approximately 2400 older persons. An average general practitioner cares for approximately 300 older persons. Assuming that 200 of the 300 persons participate in the program, approximately 12 general practitioners will be needed at each site.

**<u>Step 2:</u> Each practice generates a list of eligible older persons:**
Each practice produces a list of all persons aged 65 years or older, living at home, and speaking the German language. Each practice will have a final list with addresses, gender, and age of eligible persons.

**<u>Step 3:</u> Physicians circles**
Three large practice circles will be identified. One circle will be allocated (by random, notification by DMC Bern) to “No physician training” (group C), and two groups will be assigned to “physician training” (group AB). Physician interaction and patient crossover would have resulted in unacceptable contamination in group B.
Physicians allocated to “Physician training” will get an initial training session (approximately one day) and ongoing training throughout the project (e.g., monthly training end reinforcement visits by project geriatrician to physician per month, including academic detailing and case discussion). Physicians allocated to “Physician training at project end” will get no additional training at the start of the project (they will get the intervention manual at the end of the project) and the older persons of these physicians will constitute a second control group (group C).

**<u>Step 4:</u> Randomisation of older persons**
Within each physician practice of group A/B (trained physicians), older persons will be randomised to the intervention group (group A and to the control group (group B). For doing the randomisation, a special data management system will be implemented. Eligible person of group A/B can be entered by the regional centre into the database, and the data management system will inform the regional centre about the result of random allocation. (A sealing system ensuring blind and irreversible randomisation will be implemented). Random allocation will be by household.
The older persons of the physicians assigned to “No physician training” will NOT be randomised and will all receive usual care (group C).

**Figure 1: Design of randomized of study (each site)**

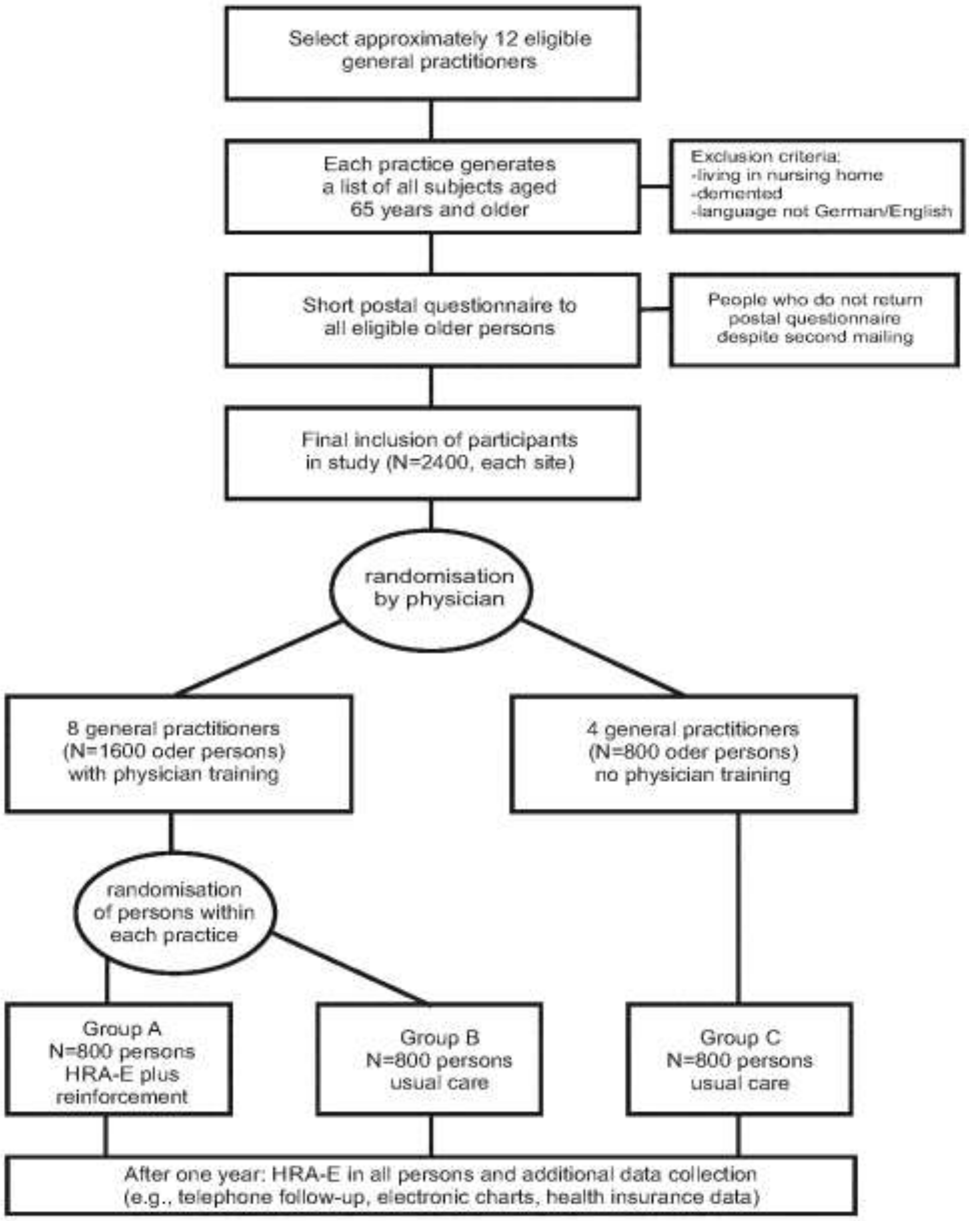

# 7. Plan for inclusion/ exclusion of subjects for randomised study

1. Physician practice generates list of all persons of his/her practice aged 65 and older of their practice. This list is printed. There is a system to make sure that this list is complete (possible assistance of physician if printed cards only, double check of plausibility if it is computer-generated extract).
2. The practice excludes the following persons based on **administrative criteria**:
   - persons living in a nursing home or other form of assisted living (persons live in an environment where they get personal care of supervision, e.g. supervision of medication intake)
   – persons who died ("should not be on the list, but are on the list")
   – persons who have a different primary care provider (changed to a different practice, are recorded because they have been seen for emergency care only, persons who have moved away)
3. The remaining list is checked based on the following **clinical criteria**, and the following persons are excluded:
   – persons who do not speak the regional language (L)
   – persons with terminal disease (T)
   – persons who are severely disabled or live in a nursing home (N)
   – persons with dementia or probable dementia (D) (corresponds to a Mini-Mental Status lower than 24)
4. All remaining persons get an invitation letter to participate in the project (letter from general practitioner, with cover letter for invitation, project description, questionnaire, and if needed an informed consent form) and are asked to return **the pre-randomisation questionnaire** (and if needed, the consent form) (see below). The returned pre-randomisation questionnaires will be checked, and persons will be excluded if:
   – the questionnaire is not filled out completely
   – according to the questionnaire, the persons need assistance for bathing
5. Persons, who do not return the invitation letter, get telephone calls (by the practice, or by the project), in an attempt to motivate these persons to participate. If persons **refuse** to participate despite the reminder call, they are asked for the reason for not returning the questionnaire.
   The reasons will be listed, and categorised if possible along the following categories:
   - persons who do not want to participate because they think they are too sick for participating in a preventive program;
   - persons who do not want to participate because they think they too healthy or they do not need a preventive program;
   – persons who have no interest in participating and do not offer a specific reason against such a program.
6. Based on this, a **flow chart** with the exact numbers for each of the steps and substeps will be generated. If possible, from the data protection perspective, age, gender, and self-perceived health status of refusers and of excluded persons will be collected (important for addressing the generalisability of the study).

# 8. Pre-randomisation questionnaire

We used the following criteria for defining the pre-randomisation questionnaire:

- The questionnaire must be self-administered.
- The questionnaire should be short (not more than one page)
- The questionnaire should not lead to contamination (therefore, no questions with regard to preventive care or health behaviour)
- The questionnaire should be validated and give a quantitative assessment of risk status.

In an extensive literature search, only the instrument developed by Boult Ch. et al. met all criteria. The PRA instrument classifies persons into high risk (PRA: Probability of the Risk for Hospital Admission) based on a multiple logistic regression analysis approach. The instrument has been extensively validated in the United States for the prediction of hospital admission, and has been shown to predict other health risks as well (including the risk for nursing home admission and the risk for mortality). It is widely used by organisations across the United States.

We added a question on need for help in basic activities of daily living (Katz et al.).

The pre-randomisation questionnaire serves the following purposes:

- to exclude non-eligible persons (the following criteria will be used:
  - persons not answering the first three PRA questions or
  – persons not answering the question on need for help
  – persons who state that they need help in the basic activities of daily living
  (- for additional exclusion criteria, see section on eligibility)
- to compare groups at baseline:
  - randomisation between groups A versus B can be checked
  - group C can be compared with groups A and B (randomisation on physician and not patient level)
  – for the analytic plan: these risk factors can be used as adjusters for the outcome analyses (i.e., possible differences between groups A,B,C can be corrected for)
  – possible differences in health status between sites can be taken into account
- to conduct post-randomisation stratified analyses:
  There is an a priori hypothesis that the intervention effect (longer term) depends on the PRA risk at baseline. The hypothesis is that favourable effects are stronger among persons at low risk (as compared to persons at low risk). Due to the fact, that the distribution of PRA is unknown in European populations, the stratification cut-off will be determined after randomisation.

# 9. Contamination

Contamination might occur under the current study design in different ways:

- Trained physicians might give better care to patients in group B;
- Trained nurses might give better care to patients in group B;
- Cross-treatment of patients in group C by trained physicians or trained nurses might result in better care;
- An organisational change (improving the system for preventive care).

There are two approaches to deal with contamination:

**Methods to minimise contamination**

Each site will implement ways to minimise contamination, e.g.:

- no relevant cross-treatment of patients of group C by trained physicians
- nurse/ health educator does not offer intervention to subjects in group B/C
- no training of physicians of group C until project end
- identify and minimise other possible sources of contamination

**Methods to measure contamination**

Contamination is not completely avoidable, and its extent may vary according to site. Each site will define methods for measuring the occurrence of contamination in a quantitative way.

## 10. Intervention design (Figure 2)

The core of the intervention process is:

A. Use of the European version of the HRA-E system (same content, same logic), including questionnaire, participant report, and physician summary;
B. Integration of the intervention in primary care system with (1) HRA-E is offered to older persons by primary care physician, (2) physicians are trained based on a training manual (guidelines generic, special comments regional);
C. There is a system of health education with ongoing reinforcement of the recommendations over the one-year follow-up period. Health education is based on the principle of the stages of change (transtheoretical model) and the promotion of self-efficacy.
D. The primary care physician will be supported by specially trained health educators. In Switzerland specially trained health nurses who collaborate with a geriatrician will assume this role.

**Step 1:** The physicians of the persons of group A will get additional training in using and reinforcing the HRA-E. For this purpose, the learning objectives and the exact content of the training program for the participating primary care practitioners will be determined. Both individual and group-based training will be used. During the ongoing project, there will be follow-up training sessions (about once per month) to reinforce the training and to discuss the experience with the ongoing intervention. The programs will be adapted to the environment of each centre.
**Step 2:** After randomisation, persons of group A will get a questionnaire from their primary care physician (he/she will be assisted by the research staff of the project). The older person will be invited to fill out and return the questionnaire with an attached response envelope. The physician will write a cover letter explaining that this is part of his/her care approach, and that this method will be tested scientifically, but that each person has the choice of participating (non-participation would not result in any disadvantage for the patient).
**Step 3:** The older person returns the questionnaire to the participant by mail. Older people who do not return the questionnaire will get written or telephone reminders. In addition, a field worker is available for older persons who need further help filling out questionnaires.
**Step 4:** The filled out questionnaires are brought to the study centre of each site, the questionnaire will be entered in the computer, the feed-back report will be printed, the summary for the physician will be printed, the material will be checked (beta-testing), the feed-back report will be mailed to the older person, and the summary report will be mailed to the physician.
**Step 5:** During the one year intervention follow-up period, the primary care physician reinforces the recommendations given in the report. Specific plans will be made for reinforcing the interventions. Focus group meetings and questionnaires have shown that physicians would be willing to collaborate with specially trained health nurses, who might assist them in educating the older patients. In developing the method for reinforcement, low cost approaches and clear guidelines will be emphasised (e.g. follow-up telephone call by health educator).

**Figure 2: Basic design of the intervention**

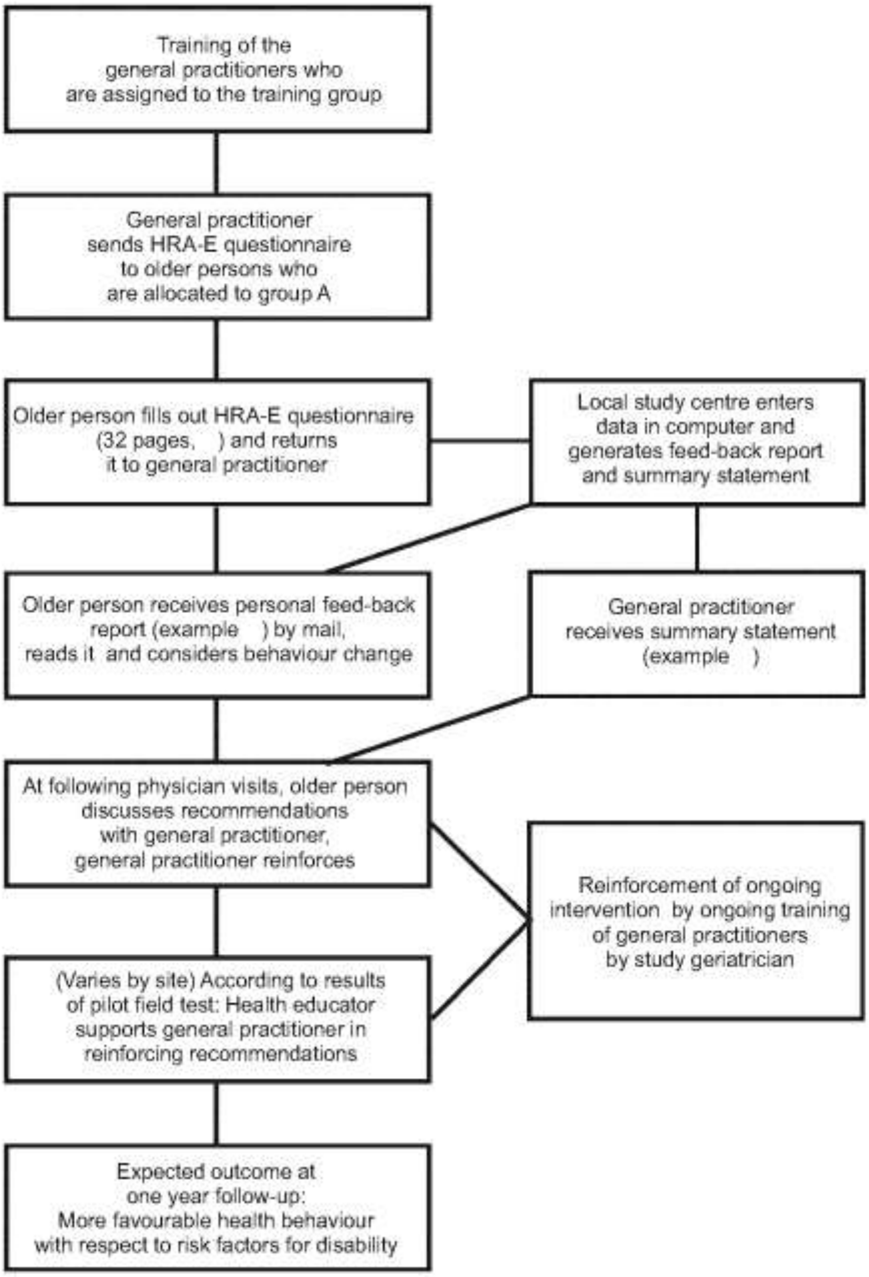

## 11. Methods to measure the intervention

Methods to measure the intensity of the intervention

- first, each site operationalises the method of intervention (available time of health educators, professional background of health educator, organisation of work for general practitioner, required material for implementing the intervention follow-up)
- quantitative description of the intervention once the method has been defined

## 12. Methods to measure the satisfaction of providers

Systematic evaluation of the satisfaction of the providers (physicians, nurses) at the end of the project.

## 13. Data confidentiality

All data will be anonymised for data analysis. The name of the older person, the name of the physician, and the name of the health educator will not be identifiable in the analytic data file. The data management system will be programmed in a way to ensure this principle.

# 14. Statistical analysis

The statistical work will include several steps during the ongoing project:

**Step 1: Creation of common database**

The development and the maintenance of a high quality database for the expected number of several thousand observations is key for the project. In a first step, codebooks and databases for all quantitative information will be developed. In addition, a system for the management of the data (importing the data, merging of different databases, controlling the quality of the data, calculating secondary variables for statistical analysis) will be organised. This will be possible in a relatively short period of time, because participant 2 has already created a basic data management system for the HRA-E in the course of the ongoing pilot field tests in Switzerland.

**Step 2: Randomisation**

The statistical centre will be responsible for organising an independent centralised system of random allocation. The information from persons included in the study will be anonymised and the anonymised lists will be transferred to the statistical centre. The centre will then inform the regional centre about the result of random allocation and use this information for the intention-to-treat analysis.

**Step 3: Descriptive analysis of base-line results of participating subjects**

The information collected with the short postal questionnaires will be analysed and the centre will check the equivalence of groups A, B, and C. In addition, the prevalence data will be compared with other statistics to ensure generalisability of the study.

**Step 4: Descriptive analysis of data from initial health risk appraisal**

The information collected with the base-line HRA-E in subjects assigned to the intervention group will be analysed and the information from the three sites compared. In addition, prospective data collection will be used to measure the quantity and type of reinforcing interventions for subjects in the intervention group.

**Step 5: Analysis of outcome results**

The final analysis will be conducted according to the priori analytic plan. For the randomised studies, the following main comparisons will be made:

(a) Comparison group A (one year follow-up) with group B (one year follow-up): Intervention effect but possible contamination bias: (bias by spurious reduction in the difference in clinical outcomes between experimental and control groups because control patients accidentally receive part of the intervention). The physicians responsible for persons in group B get additional training, therefore, although persons in group B do not receive the HRA-E intervention, they might benefit from the physician training.

(b) Comparison of group A (one year follow-up) with group C (one year follow-up): Intervention effect (no contamination, however based on randomisation by physician (N=12) and not by older persons).

(c) Comparison of group A (base-line) with group C (one year follow-up) Secular change (plus a minor change due to the fact that people in group C are one year older at the time of examination).

Furthermore, the relationship between intervention process (e.g., intensity of reinforcement), health care system, and characteristic of the older person (e.g., age, risk status, socio-economic status) with outcomes will be analysed.

# 15. References


1. Applegate WB, Pressel S, Wittes J et al. Impact of treatment of isolated systolic hypertension on behavioral variables. Arch Intern Med 1994; 154:2154-60.
2. Boult C, Dowd B, McCaffrey D, Boult L, Hernandez R, Krulewitch H: Screening elders for risk of hospital admission. J Am Geriatr Soc 1993, 41:811-17.
3. Breslow L, Beck JC, Morgenstern H et al. Development of a Health Risk Appraisal for the elderly (HRA-E). Am J Health Promot 1997; 11:337-43.
4. Buchner DM, Beresford SA, Larson EB, LaCroix AZ, Wagner EH. Effects of physical activity on health status in older adults. II. Intervention studies. Ann Rev Public Health 1992; 13:469-88.
5. Katz S, Ford AB, Moskowitz RW, Jackson BA, Jaffe MW: Studies of illness in the aged. The index of ADL: A standardized measure of biological and psychosocial function. JAMA 1963, 185:914-919.
6. Stewart AL, King AC, Haskell WL. Endurance exercise and health related quality of life in 50-65 year-old adults. 1993; 33:782-9.
7. Verbrugge LM, Jette AM. The disablement process. Soc Sci Med 1994; 38:1-14.
8. Wagner EH, LaCroix AZ, Grothaus L et al. Preventing disability and falls in older adults: a population-based randomized trial. Am J Publ Health 1994; 84:800-6.

# Change Protocol:
# Document of Changes

# PRO-AGE Solothurn Study Plan
## (Prevention in older persons – assessment in generalists' practices) Version Sept 29, 2000

| Date | Chapter | Description of and Rationale for Change | Effects on other documents |
|---|---|---|---|
| Sept 29, 2000 | 10 | Health Risk Appraisal questionnaire:<br>Based on prior pilot studies, the U.K. English version of the base-line Health Risk Appraisal questionnaire was finalized. | Older Persons Health Profile Questionnaire [Health Risk Appraisal for Older Persons questionnaire] |
| Nov 20, 2000 | 10, Appendix 1 | Health Risk Appraisal questionnaire:<br>Based on prior pilot studies, the German version, for use in Switzerland, of the base-line Health Risk Appraisal questionnaire was finalized. | German Version for Use in Switzerland: Older Persons Health Profile Questionnaire [Health Risk Appraisal for Older Persons questionnaire] |
| Nov 20, 2000 | 10, Appendix 2 | Feed-back report for older participants:<br>Based on prior pilot studies, the German version, for use in Switzerland, of the specifications for the participant report were finalized and the software program for generating the feed-back reports was finalized. | Sample participant report (Your Older Persons Health Profile Report) |
| Nov 20, 2000 | 10, Appendix 3 | Feed-back report for physicians:<br>Based on prior pilot studies, the German version, for use in Switzerland, of the specifications for the provider report were finalized and the software program for generating the feed-back reports was finalized. | Sample provider report |
| Jan 26, 2001 | 10 | Intervention manual:<br>The UK English version of the intervention manual, defining main intervention principles, was finalized. | Intervention Manual (UK English) |
| March 9, 2001 | 5,6 | Change of randomization ratio:<br>Recruitment went well, and a number of more than 1000 participants in the intervention group had to be expected, if recruitment continued in the same speed. However, due to budgetary restraints it was not possible to offer the intervention to such a high number of participants (high costs of the intervention). For this reason, the PRO-AGE Solothurn trial steering committee (Andreas Stuck, Christoph E. Minder, Stephan Born, and John C. Beck) reconsidered the randomization ratio, and agreed on the following amendment of the study protocol, based on an updated power calculation: change of randomization ratio of participants (intervention to control group) from 1:1 to 1:2. | no |
| Sept 16, 2001 | 9 | Prolongation of intervention by one year:<br>PRO-AGE Solothurn attempted to get additional funding for extending the follow-up from a one-year follow-up to a longer follow-up for the randomized controlled study (intervention and control group). The PRO-AGE Solothurn trial steering committee (Andreas Stuck, Christoph E. Minder, Stephan Born, and John C. Beck) decided to extend the intervention follow-up of the intervention group by an additional year (total duration of intervention 2 years. | no |
| Sept 16, 2001 | 6 | Termination of follow-up in group C:<br>Due to lack of additional funding, the PRO-AGE Solothurn trial steering committee | no |

| | | | |
|---|---|---|---|
| | | (Andreas Stuck, Christoph E. Minder, Stephan Born, and John C. Beck) decided to terminate follow-up in group C (concurrent comparison group) at the one-year follow-up. | |
| Sept 20, 2001 | 10, Appendix 4 | The final e German version: for use in Switzerland, PRO-AGE Intervention Manual defining main intervention principles for Switzerland, was finalized. | Intervention Manual |
| Sept 23, 2002 | 3,4 | Plan for two-year follow-up:<br>Based on available funding, the PRO-AGE Solothurn trial steering committee (Andreas Stuck, Christoph E. Minder, Stephan Born, and John C. Beck) made the following decisions:<br>1. Termination of the intervention in the intervention group after completion of the second study year.<br>2. Use of a short self-administered questionnaire and brief chart abstraction as methods for follow-up (this decision was made, because a method was searched to maximize return rates of two-year outcome data). | no |
| March 29, 2003 | 4,6,8,9,14 | Plan for one-year follow-up:<br>The interim analysis revealed that 32.5% of surviving participants (intervention and control group) did not return the one-year follow-up questionnaire. The PRO-AGE Solothurn trial steering committee (Andreas Stuck, Christoph E. Minder, Stephan Born, and John C. Beck) decided:<br>1. For comparisons of intervention versus control groups, one-year follow-up data cannot be used for outcome analysis due to this high missing data rate. Instead, information on two-year outcomes should be used for outcome analysis according to the plan for the two-year follow-up.<br>2. The planned analyses related to the concurrent control group C for measuring potential contamination effects is not possible, and has to be omitted. | Statistical analysis plan: Initial Version |
| Sept 16, 2011 | 3,4,16 | Plan for data linkage:<br>For long-term follow-up, the members of the PRO-AGE Solothurn trial steering committee were Andreas Stuck, André Moser, Stephan Born, John C. Beck, and Matthias Egger. This committee searched for the availability of a potential source of information for obtaining long-term follow-up outcomes. This group identified the “Swiss National Cohort database” (SNC database) as a new potential source of information that had not been available before.<br>The PRO-AGE Solothurn trial steering committee decided to explore whether a permission could be obtained for linkage of the PRO-AGE Solothurn trial database with the SNC database. | no |

# PRO-AGE Solothurn Study Plan
## (Prevention in older persons – assessment in generalists' practices) Version Sept 29, 2000

## *(Updates according to change protocol)*


1. Goal ..... 2
2. Background ..... 3
3. Main Hypotheses of Randomised Study ..... 4
4. Outcome measures ..... 5
5. Sample size calculation ..... 6
6. Overall design of the randomised study (Figure 1) ..... 8
7. Plan for inclusion/ exclusion of subjects for randomised study ..... 10
8. Pre-randomisation questionnaire ..... 11
9. Contamination ..... 12
10. Intervention design (Figure 2) ..... 13
11. Methods to measure the intervention ..... 15
12. Methods to measure the satisfaction of providers ..... 16
13. Data confidentiality ..... 17
14. Statistical analysis ..... 18
15. References ..... 19
*16. Addendum: data linkage for eight-year outcomes* ..... 20

# 1. Goal

The ultimate goal of the project is to maintain function and in the mid to long term prevent the onset of disability and to minimise unnecessary service utilisation among persons 65 years of age and older. Health risk appraisal (HRA), i.e. identifying modifiable risk factors for disease and disability, and seeking to minimise their adverse impact, is being widely applied for working age adults. Needed now is a comprehensive HRA for elderly persons (HRA-E) designed to reduce functional impairment, the major health problem among the rapidly growing elder population.

## 2. Background

Verbrugge and Jette (1994) hypothesise that the onset of disability is related to a multiplicity of underlying biological, psychological, social, functional, and environmental risk factors. Based on this model, we hypothesise that effective prevention of disability in older persons should be based on early modification of relevant risk factors. Many studies have shown (e.g., Stewart et al. 1993) that older individuals are often willing to participate in interventions designed to improve their health behaviours or reduce risk behaviours, and, furthermore, that these interventions may result in improvements in their health, functioning and perceptions of well-being. Several studies support the efficacy of preventive interventions to not only change health and safety practices of the elderly, for example by increasing exercise (Buchner et al., 1992), reducing hypertension (Applegate et al., 1994), or improving cardiovascular fitness (Stewart et al., 1993), but also to achieve improvements in health, functional status, and perceived quality of life (Applegate et al., 1994). For example, Wagner et al. (1994) demonstrated that a multicomponent intervention consisting of nurse assessment and follow-up in-home sessions targeting risk factors resulted in significantly lower decline of functional status and significantly lower incidence of falls than subjects who received usual care.

In December 1992 a group of University faculty (United States) began developing a health risk appraisal instrument for the elderly (HRA-E). Their work was supported by the John A. Hartford Foundation, The California Wellness Foundation, and the Health Care Financing Organisation. The products of their work to date include (Breslow et al., 1997): (1) a questionnaire for comprehensive health risk assessment of older persons; (2) software for processing computer-generated, personalised reports to participants and their physicians from the questionnaires, and (3) favourable preliminary findings from testing the questionnaire and software in older subjects. The development of the HRA-E was based on a systematic literature review, a priori selection criteria for selection of domains and items, focus group meetings, and on testing of several intermediate versions. Extensive field testing showed favourable data on acceptance and self-reported behaviour change in a sample of approximately 2000 older Americans

# 3. Main Hypotheses of Randomised Study

**We expect:**

A. The intervention will reduce overall and specific health risk
B. The intervention will improve the older persons' self efficacy
C. The participation rate among older persons will be high (over 70 percent)
D. The intervention will result in a high participation rate.

***Amendment, based on decision of steering committee Sept 23, 2002***
*We expect, specifically at two-year follow-up:*
*The intervention will increase recommended preventive care use.*
*The intervention will increase adherence with recommended health behaviour.*

***Amendment, based on decision of steering committee Sept 16, 2011***
*We expect, specifically at eight-year follow-up:*
*The intervention will increase survival.*

# 4. Outcome measures

***Amendment, based on decision of steering committee March 29, 2003***
*Outcomes at one-year follow-up*
*Data not usable due to high missing data rate.*

***Amendment, based on decision of steering committee Sept 23, 2002***
*Primary outcomes at two-year follow-up*
*Preventive care use (review of primary care medical records)*
- *blood pressure measurement in previous year*
- *cholesterol measurement in previous 5 years (age < 75 years)*
- *fasting blood glucose measurement in previous three years*
- *colon cancer screen in previous year (age < 80 years)*
- *influenza immunisation in previous year*
- *pneumococcal immunisation (ever)*

*Health behaviour (self-report)*
- *>= 30 minutes/day moderate/strenuous activity*
- *low fat consumption (daily or several times per week)*
- *high fibre / fruit consumption (daily or several times per week)*
- *At most 1 alcoholic drink per day*
- *No current tobacco use*
- *Use of safety belts*

*Secondary outcomes at two-year follow-up*
- *Survival and nursing home admission*
- *Self-perceived health*
- *Need for help in basic activities of daily living*

***Amendment, based on decision of steering committee Sept 16, 2011***
*Primary outcomes at eight-year follow-up*
- *Survival (Kaplan-Meier estimates)*

*Secondary outcomes at eight-year follow-up*
- *Cause of death*

# 5. Sample size calculation

The sample size calculations are based on expected frequencies of adverse health care behaviour and the expected impact of the intervention on the health behaviour. Table 1 presents the observed yield of the HRA-E in an older American population and the self-reported improvement in health behaviour:

Power estimates are shown in Table 2, by the frequency of the outcome event (1-50%) and the minimum effect to be detected (relative risk 1.1 to 3.0). The planned sample size of 800 subjects per group will yield good power (80% and better) for detecting small-to-moderate effects on both rare and common outcome events, e.g. a relative risk of 1.2 (i.e., a 20 percent increase in favourable health care behaviour) with an outcome frequency of 25%.

**Table 1:** Adverse health habits in 1482 older Americans (aged 55 years and older) and % of subjects improving this behaviour ( 4-month telephone follow-up interview)

| | Number of persons receiving message in HRA-E (N=1482) | % of persons receiving message who improved |
|---|---|---|
| **Adverse health habit** | | |
| Minimal or very limited exercise | 280 | 27 |
| Eating a high fat diet | 902 | 44 |
| Not wearing seatbelts | 75 | 16 |
| Drinking alcohol excessively | 480 | 21 |
| Having depressed mood | 209 | 15 |
| Smoking | 280 | 21 |
| **Tardy preventive behaviour** | | |
| Blood pressure measurement | 51 | 21 |
| Vision examination | 248 | 54 |
| Hearing examination | 774 | 19 |
| Dental examination | 203 | 70 |
| Cholesterol measurement | 105 | 65 |
| Colonoscopy | 377 | 25 |
| Influenza vaccination | 440 | 53 |
| Pneumococcal vaccination | 690 | 24 |
| Mammogram | 52 | 67 |
| Breast examination | 32 | 68 |
| Pap smear | 90 | 52 |

***Amendment, based on revised calculations, March 9, 2001***

***Table 2:*** *Estimated power (%) for comparing two groups, by expected frequency of the outcome event and the minimum effect (relative risk) to be detected (assuming equal sample size of 800 per group).*

| Outcome frequency (%) among controls | Minimum Relative Risk | | | | | | | | | |
|---|---|---|---|---|---|---|---|---|---|---|
| | 1.1 | 1.2 | 1.3 | 1.4 | 1.5 | 1.6 | 1.7 | 2 | 2.5 | 3 |
| 1 | 3% | 3% | 5% | 7% | 10% | 13% | 17% | 30% | 55% | 76% |
| 2 | 3% | 6% | 9% | 14% | 20% | 27% | 35% | 59% | 88% | 98% |
| 3 | 4% | 8% | 13% | 21% | 30% | 41% | 52% | 79% | 98% | 100% |
| 4 | 5% | 10% | 18% | 28% | 41% | 54% | 66% | 90% | 100% | 100% |
| 5 | 5% | 12% | 22% | 35% | 50% | 65% | 77% | 96% | 100% | 100% |
| 10 | 8% | 22% | 44% | 66% | 84% | 94% | 98% | 100% | 100% | 100% |
| 15 | 11% | 34% | 64% | 86% | 97% | 99% | 100% | 100% | 100% | 100% |
| 20 | 15% | 46% | **80%** | 96% | 100% | 100% | 100% | 100% | 100% | 100% |
| 25 | 19% | 59% | 90% | 99% | 100% | 100% | 100% | 100% | 100% | 100% |
| 30 | 24% | 71% | 96% | 100% | 100% | 100% | 100% | 100% | 100% | 100% |
| 35 | 29% | 81% | 99% | 100% | 100% | 100% | 100% | 100% | 100% | 100% |
| 40 | 35% | 89% | 100% | 100% | 100% | 100% | 100% | 100% | 100% | 100% |
| 50 | 50% | 98% | 100% | 100% | 100% | 100% | 100% | 100% | 100% | 100% |

*Power estimates are shown in the Table, by the frequency of the outcome event (1-50%) and the minimum effect to be detected (relative risk 1.1 to 3.0). The planned sample size of 800 subjects per group will yield good power (80% and better) for detecting small-to-moderate effects on both rare and common outcome events, e.g. a relative risk of 1.3 (i.e., a 30 percent increase in favourable health care behaviour) with an outcome frequency of 20% among controls.*
*We have to take into account a drop-out rate of 20%; the initial sample size is: n1=1000, n2=1000.*

***Table3:*** *Estimated sample sizes with different ratios intervention to control group:*

| *ratio n1/n2=1:1* | *ratio n1/n2=1:2* | *ratio n1/n2=1:1.5* |
|---|---|---|
| *p1 = 0.20*<br>*p2 = 0.26*<br>*n1 = 800*<br>*n2 = 800*<br>*-> power = 0.7979* | *p1 = 0.20*<br>*p2 = 0.26*<br>*n1 = 610*<br>*n2 = 1220*<br>*-> power = 0.8* | *p1 = 0.20*<br>*p2 = 0.26*<br>*n1 = 675*<br>*n2 = 1013*<br>*-> power = 0.8* |

*With drop-out rate of 20%; the initial sample size is:*
*1:2: n1 = 732, n2 = 1464*
*1:1.5: n1 = 810, n2 = 1216*

# 6. Overall design of the randomised study (Figure 1)

__Step 1:__ **Select approximately 12 participating general practitioners:**
The required number of physicians must be selected with the goal of recruiting 2400 older persons. For determining the required number of physicians, the following factors must be taken into account:

- number of older persons in practice
- percentage of eligible persons among older persons
- percentage of refusal to participate among eligible older persons

The goal is to achieve a sample of approximately 2400 older persons. An average general practitioner cares for approximately 300 older persons. Assuming that 200 of the 300 persons participate in the program, approximately 12 general practitioners will be needed at each site.

__Step 2:__ **Each practice generates a list of eligible older persons:**
Each practice produces a list of all persons aged 65 years or older, living at home, and speaking the German language. Each practice will have a final list with addresses, gender, and age of eligible persons.

__Step 3:__ **Physician circles**
Three large practice circles will be identified. One circle will be allocated (by random, notification by DMC Bern) to “No physician training” (group C), and two groups will be assigned to “physician training” (group AB). Physician interaction and patient crossover would have resulted in unacceptable contamination in group B.
Physicians allocated to “Physician training” will get an initial training session (approximately one day) and ongoing training throughout the project (e.g., monthly training end reinforcement visits by project geriatrician to physician per month, including academic detailing and case discussion). Physicians allocated to “Physician training at project end” will get no additional training at the start of the project (they will get the intervention manual at the end of the project) and the older persons of these physicians will constitute a second control group (group C).
***__Amendment, based on decision of steering committee Sept 16, 2001__***
*Project end for group C is at one-year follow-up. All physicians of group C get intervention manual after completion of first year of the study.*

__Step 4:__ **Randomisation of older persons**
Within each physician practice of group A/B (trained physicians), older persons will be randomised to the intervention group (group A and to the control group (group B). For doing the randomisation, a special data management system will be implemented. Eligible person of group A/B can be entered by the regional centre into the database, and the data management system will inform the regional centre about the result of random allocation. (A sealing system ensuring blind and irreversible randomisation will be implemented). Random allocation will be by household.
The older persons of the physicians assigned to “No physician training” will NOT be randomised and will all receive usual care (group C).
***__Amendment, based on decision of steering committee March 9, 2001__***
*Randomization ratio: intervention group (group A) to control group (group B):*
*Nov 16, 200 to March 27, 2001 ratio of 1:1*
*March 28 to end of study: ratio of 1:2*

***Amendment, based on decision of steering committee March 29, 2003***
*Comments to group C in this Figure are deleted, due to non-use of group C in final analysis.*

***Figure 1: Design of randomized study (Solothurn site)***

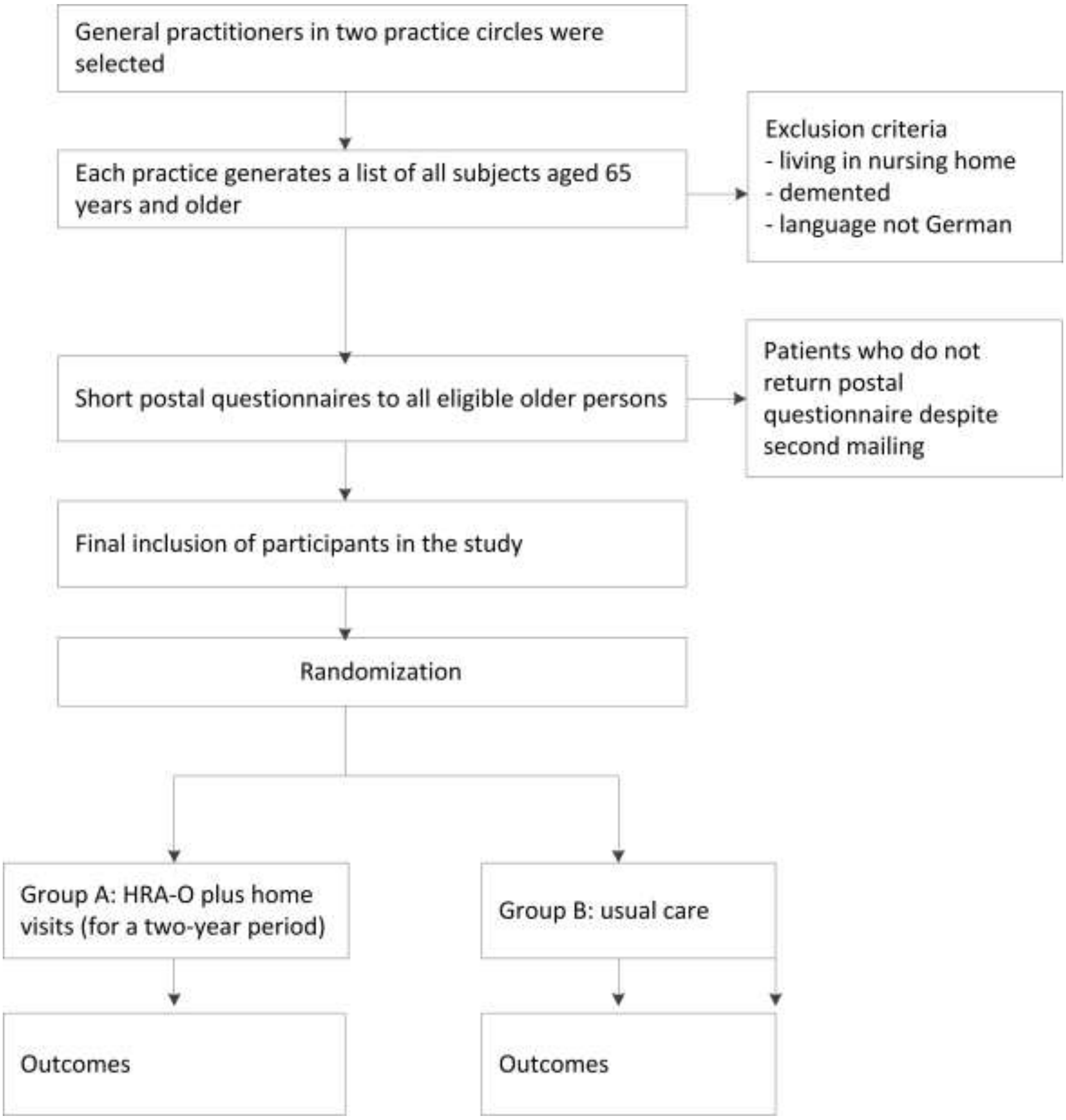

# 7. Plan for inclusion/ exclusion of subjects for randomised study

1. Physician practice generates list of all persons of his/her practice aged 65 and older of their practice. This list is printed. There is a system to make sure that this list is complete (possible assistance of physician if printed cards only, double check of plausibility if it is computer-generated extract).
2. The practice excludes the following persons based on **administrative criteria**:
   - persons living in a nursing home or other form of assisted living (persons live in an environment where they get personal care of supervision, e.g. supervision of medication intake)
   – persons who died ("should not be on the list, but are on the list")
   – persons who have a different primary care provider (changed to a different practice, are recorded because they have been seen for emergency care only, persons who have moved away)
3. The remaining list is checked based on the following **clinical criteria**, and the following persons are excluded:
   – persons who do not speak the regional language (L)
   – persons with terminal disease (T)
   – persons who are severely disabled or live in a nursing home (N)
   – persons with dementia or probable dementia (D) (corresponds to a Mini-Mental Status lower than 24)
4. All remaining persons get an invitation letter to participate in the project (letter from general practitioner, with cover letter for invitation, project description, questionnaire, and if needed an informed consent form) and are asked to return **the pre-randomisation questionnaire** (and if needed, the consent form) (see below). The returned pre-randomisation questionnaires will be checked, and persons will be excluded if:
   – the questionnaire is not filled out completely
   – according to the questionnaire, the persons need assistance for bathing
5. Persons, who do not return the invitation letter, get telephone calls (by the practice, or by the project), in an attempt to motivate these persons to participate. If persons **refuse** to participate despite the reminder call, they are asked for the reason for not returning the questionnaire.
   The reasons will be listed, and categorised if possible along the following categories:
   - persons who do not want to participate because they think they are too sick for participating in a preventive program;
   - persons who do not want to participate because they think they too healthy or they do not need a preventive program;
   – persons who have no interest in participating and do not offer a specific reason against such a program.
6. Based on this, a **flow chart** with the exact numbers for each of the steps and substeps will be generated. If possible, from the data protection perspective, age, gender, and self-perceived health status of refusers and of excluded persons will be collected (important for addressing the generalisability of the study).

# 8. Pre-randomisation questionnaire

We used the following criteria for defining the pre-randomisation questionnaire:

- The questionnaire must be self-administered.
- The questionnaire should be short (not more than one page)
- The questionnaire should not lead to contamination (therefore, no questions with regard to preventive care or health behaviour)
- The questionnaire should be validated and give a quantitative assessment of risk status.

In an extensive literature search, only the instrument developed by Boult Ch. et al. met all criteria. The PRA instrument classifies persons into high risk (PRA: Probability of the Risk for Hospital Admission) based on a multiple logistic regression analysis approach. The instrument has been extensively validated in the United States for the prediction of hospital admission, and has been shown to predict other health risks as well (including the risk for nursing home admission and the risk for mortality). It is widely used by organisations across the United States.

We added a question on need for help in basic activities of daily living (Katz et al.).

The pre-randomisation questionnaire serves the following purposes:

- to exclude non-eligible persons (the following criteria will be used:
  - persons not answering the first three PRA questions or
  – persons not answering the question on need for help
  – persons who state that they need help in the basic activities of daily living
  (- for additional exclusion criteria, see section on eligibility)
- to compare groups at baseline:
  - randomisation between groups A versus B can be checked
  – for the analytic plan: these risk factors can be used as adjusters for the outcome analyses (i.e., possible differences between groups A,B can be corrected for)
  – possible differences in health status between sites can be taken into account
- to conduct post-randomisation stratified analyses:
  There is an a priori hypothesis that the intervention effect (longer term) depends on the PRA risk at baseline. The hypothesis is that favourable effects are stronger among persons at low risk (as compared to persons at low risk). Due to the fact, that the distribution of PRA is unknown in European populations, the stratification cut-off will be determined after randomisation.

***<u>Amendment, based on decision of steering committee March 29, 2003</u>***
*Comments to group C in this chapter deleted, due to non-use of group C in final analysis.*

# 9. Contamination

Contamination might occur under the current study design in different ways:

- Trained physicians might give better care to patients in group B;
- An organisational change (improving the system for preventive care).

***Amendment, based on decision of steering committee March 29, 2003***

*Comments to group C in this chapter deleted, due to non-use of group C in final analysis.*

# 10. Intervention design (Figure 2)

The core of the intervention process is:

A. Use of the European version of the HRA-E system (same content, same logic), including questionnaire, participant report, and physician summary;
B. Integration of the intervention in primary care system with (1) HRA-E is offered to older persons by primary care physician, (2) physicians are trained based on a training manual (guidelines generic, special comments regional);
C. There is a system of health education with ongoing reinforcement of the recommendations over the one-year follow-up period. Health education is based on the principle of the stages of change (transtheoretical model) and the promotion of self-efficacy.
D. The primary care physician will be supported by specially trained health educators. In Switzerland specially trained health nurses who collaborate with a geriatrician will assume this role.

**Step 1:** The physicians of the persons of group A will get additional training in using and reinforcing the HRA-E. For this purpose, the learning objectives and the exact content of the training program for the participating primary care practitioners will be determined. Both individual and group-based training will be used. During the ongoing project, there will be follow-up training sessions (about once per month) to reinforce the training and to discuss the experience with the ongoing intervention. The programs will be adapted to the environment of each centre.
**Step 2:** After randomisation, persons of group A will get a questionnaire from their primary care physician (he/she will be assisted by the research staff of the project). The older person will be invited to fill out and return the questionnaire with an attached response envelope. The physician will write a cover letter explaining that this is part of his/her care approach, and that this method will be tested scientifically, but that each person has the choice of participating (non-participation would not result in any disadvantage for the patient).
**Step 3:** The older person returns the questionnaire to the participant by mail. Older people who do not return the questionnaire will get written or telephone reminders. In addition, a field worker is available for older persons who need further help filling out questionnaires.
**Step 4:** The filled out questionnaires are brought to the study centre of each site, the questionnaire will be entered in the computer, the feed-back report will be printed, the summary for the physician will be printed, the material will be checked (beta-testing), the feed-back report will be mailed to the older person, and the summary report will be mailed to the physician.
**Step 5:** During the one year intervention follow-up period, the primary care physician reinforces the recommendations given in the report. Specific plans will be made for reinforcing the interventions. Focus group meetings and questionnaires have shown that physicians would be willing to collaborate with specially trained health nurses, who might assist them in educating the older patients. In developing the method for reinforcement, low cost approaches and clear guidelines will be emphasised (e.g. follow-up telephone call by health educator).

***Amendment, based on decision of steering committee Sept 16, 2001***
*The intervention follow-up period is two years.*

***Amendment, based on decision of steering committee Sept 29, 2000***
*Approval of final U.K. English version of questionnaire for step 2.*

***Amendment, based on decision of steering committee Nov 20, 2000***
*Approval of final German version of questionnaire for step 2 and feed-back reports for step 4 (see Appendix 1 to 3, this Protocol).*

***Amendment, based on decision of steering committee Jan 26, 2001***
*Approval of final U.K. English version of Intervention Manual.*

***Amendment, based on decision of steering committee Sept 20, 2001***
*Approval of final German version of Intervention Manual (see Appendix 4, this Protocol).*

**Figure 2: Basic design of the intervention**

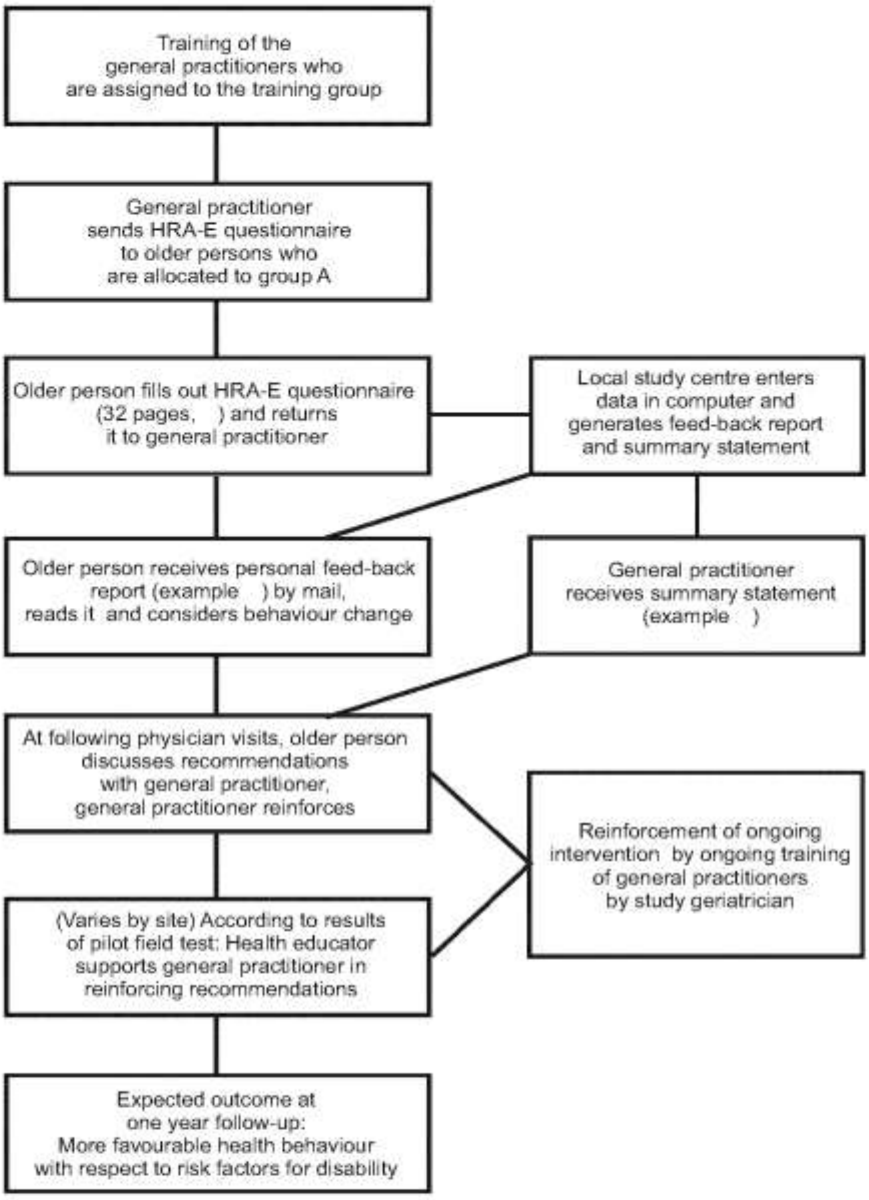

# 11. Methods to measure the intervention

Methods to measure the intensity of the intervention
first, each site operationalises the method of intervention (available time of health educators, professional background of health educator, organisation of work for general practitioner, required material for implementing the intervention follow-up)

- quantitative description of the intervention once the method has been defined

## 12. Methods to measure the satisfaction of providers

Systematic evaluation of the satisfaction of the providers (physicians, nurses) at the end of the project.

## 13. Data confidentiality

All data will be anonymised for data analysis. The name of the older person, the name of the physician, and the name of the health educator will not be identifiable in the analytic data file. The data management system will be programmed in a way to ensure this principle.

# 14. Statistical analysis

The statistical work will include several steps during the ongoing project:

**Step 1: Creation of common database**

The development and the maintenance of a high quality database for the expected number of several thousand observations is key for the project. In a first step, codebooks and databases for all quantitative information will be developed. In addition, a system for the management of the data (importing the data, merging of different databases, controlling the quality of the data, calculating secondary variables for statistical analysis) will be organised. This will be possible in a relatively short period of time, because participant 2 has already created a basic data management system for the HRA-E in the course of the ongoing pilot field tests in Switzerland.

**Step 2: Randomisation**

The statistical centre will be responsible for organising an independent centralised system of random allocation. The information from persons included in the study will be anonymised and the anonymised lists will be transferred to the statistical centre. The centre will then inform the regional centre about the result of random allocation and use this information for the intention-to-treat analysis.

**Step 3: Descriptive analysis of base-line results of participating subjects**

The information collected with the short postal questionnaires will be analysed and the centre will check the equivalence of groups A and B. In addition, the prevalence data will be compared with other statistics to ensure generalisability of the study.

**Step 4: Descriptive analysis of data from initial health risk appraisal**

The information collected with the base-line HRA-E in subjects assigned to the intervention group will be analysed and the information from the three sites compared. In addition, prospective data collection will be used to measure the quantity and type of reinforcing interventions for subjects in the intervention group.

**Step 5: Analysis of outcome results**

The final analysis will be conducted according to the priori analytic plan.

***Amendment, based on decision of steering committee March 29, 2003***

*Comments related to one-year follow-up analyses (A versus B) and comments to group C in this chapter deleted, due to non-usability of one-year follow-up data for analysis.*

# 15. References


1. Applegate WB, Pressel S, Wittes J et al. Impact of treatment of isolated systolic hypertension on behavioral variables. Arch Intern Med 1994; 154:2154-60.
2. Boult C, Dowd B, McCaffrey D, Boult L, Hernandez R, Krulewitch H: Screening elders for risk of hospital admission. J Am Geriatr Soc 1993, 41:811-17.
3. Breslow L, Beck JC, Morgenstern H et al. Development of a Health Risk Appraisal for the elderly (HRA-E). Am J Health Promot 1997; 11:337-43.
4. Buchner DM, Beresford SA, Larson EB, LaCroix AZ, Wagner EH. Effects of physical activity on health status in older adults. II. Intervention studies. Ann Rev Public Health 1992; 13:469-88.
5. Katz S, Ford AB, Moskowitz RW, Jackson BA, Jaffe MW: Studies of illness in the aged. The index of ADL: A standardized measure of biological and psychosocial function. JAMA 1963, 185:914-919.
6. Stewart AL, King AC, Haskell WL. Endurance exercise and health related quality of life in 50-65 year-old adults. 1993; 33:782-9.
7. Verbrugge LM, Jette AM. The disablement process. Soc Sci Med 1994; 38:1-14.
8. Wagner EH, LaCroix AZ, Grothaus L et al. Preventing disability and falls in older adults: a population-based randomized trial. Am J Publ Health 1994; 84:800-6.

# *16. Addendum: data linkage for eight-year outcomes*

***Amendment, this section added based on decision of steering committee Sept 16, 2011***

*The Swiss National Cohort database (SNC) is a potential source of information. The SNC is a longitudinal study of all Swiss residents included in the national censuses (Swiss National Census) of 1990 and 2000 (N=7.3 million) linked with official death and emigration records until Dec 31, 2008 [Spoerri A, Zwahlen M, Egger M, Bopp M. The Swiss National Cohort: a unique database for national and international researchers. Int J Public Health 2010;55:239–42. doi:10.1007/s00038-010-0160-5.].*

*With the SNC, the following data can be added for analysis:*
*Baseline data from Swiss National Census*

*Education*
*Living arrangement*
*Marital status*
*Religious affiliation*
*Socio-economic status*

*Outcome data from death records:*

- *Mortality: Survival information until Dec 31, 2008*
- *Nursing home admission: Not included*
- *Functional status: Not included.*

*SNC records will be linked to the database of the present study using deterministic and probabilistic methods [Fellegi IP, Sunter AB. A Theory for Record Linkage. J Am Stat Assoc 1969;64:1183. doi:10.2307/2286061; Newcombe HB. Handbook of record linkage. Methods for health and statistical studies, administration, and business. Oxford [Oxfordshire], New York: Oxford University Press, 1988]. Linkage will be done by an independent research group. Deterministic linkage will be based on main linkage variables (sex, date of birth, community of residence). Cases that cannot be linked with the main linkage variables, will be linked by a second deterministic linkage using additional variables (community of birth or country of birth) and probabilistic linkage using auxiliary variables (community of birth, country of birth, canton of birth, education or mother tongue). Alternative records will be excluded by linkage outcome weights. The linkage will be anonymous (e.g. no names or street addresses will be used in the linkage procedure). The final data set will consist of uniquely linked records, duplicates and non-linkable cases.*

*For non-linkable cases (linkage between PRO-AGE database and SNC database not possible) and duplicates (for one case in the PRO-AGE database, there are two or more matching cases in the SNC database), we will try to obtain missing information from municipal authorities.*

# Analytic plan PRO-AGE Solothurn
# (March 29, 2003)

## 1. Main hypotheses

For two-year follow-up, we hypothesise that the intervention:

- results in a higher **uptake of preventive care** (colon cancer screening, breast cancer screening, influenza vaccination, pneumococcal vaccination, blood pressure measurement, glucose measurement, cholesterol measurement)

- results in favourable changes in **health behaviour** (higher level of physical activity, reduced fat intake, higher fruit/fibre intake, reduction of hazardous alcohol use, increase in seat belt use, reduction of smoking),

# 2. Main analyses: Comparison A follow-up with B follow-up

Comparison A follow-up (A_2) with B follow-up (B_1) unadjusted, taking into account the household as a cluster (because randomisation was by household) (definition of outcome variables: see table below).

## 2.1. Subgroup analysis: Stratified analyses according to Pra risk status

Study Subhypothesis:
The intervention effects will be more favourable among subjects at low risk (based on Pra)
The Pra instrument classifies persons into high risk (Pra: Probability of the Risk for Hospital Admission) (>0.28).

Analyses are unadjusted with household as cluster.
Variables:
PRA028 = 0 low risk for hospital admission
PRA028 = 1 high risk for hospital admission

## 2.2. Analysis for effect in subgroups of persons who received site specific reinforcement

Intervention effects in persons randomised to group A who acccepted the preventive home visits versus other persons in group A

## 3. Statistical methods

Statistical analyses will take into account any cluster effect from
a) randomisation by household and
b) GP practice

PRO-AGE data arise in a clustered way. In addition to person, sources of variation are physician practice and household.

Households need to be taken into account as clusters in any case. First, members of the same household tend to have more similar health behaviour than any two persons picked at random from the population. Second, household determines randomisation; in fact, households were the units randomised, not individual patients. Thus, conceptually, households are our unit of intervention/observation.

This can be done efficiently using the GEE-methodology (Generalized Estimating Equations), which allows to characterize patterns of subjects responses of a given cluster.

# 4. Descriptive analyses

## 4.1. Descriptive analysis of intervention process

Determine the quantity of home visits that occurred during the two project years in SOLOTHURN.

## 4.2. Descriptive analysis of feed-back from physicians

Describe responses of physicians to questionnaire.

## 4.3. Descriptive analysis of feed-back from older persons

Describe feed-back of older person to questionnaire (based on base-line questionnaires, group A only).

# 5. Definition of outcome variables (two-year follow-up)

## 5.1. Primary outcomes

| | Variable Name | Variable Label | Value Label |
|---|---|---|---|
| **Primary outcomes** | | | |
| Preventive care use | | | |
| | _BLUT01 | blood pressure measurement in previous year | 1 = no 2 = yes |
| | _CHOLE01LE75 | cholesterol measurement in previous 5years (n = persons < 75 years) | 1 = no 2 = yes |
| | _GLUC01 | fasting blood glucose measurement in previous 12 months | 1 = no 2 = yes |
| | _HAEMO01_LE80 | colon cancer screen in previous year (n = persons < 80 years) | 1 = no 2 = yes |
| | _GRIPP01 | influenza immunisation in previous year | 1 = no 2 = yes |
| | _PNEUM01 | pneumococcal immunisation (ever) | 1 = no 2 = yes |
| | COMPPREV | composite score for uptake of preventive care [#1] | 0 ... 100 |
| Health behaviour | | | |
| Physical activity | YKRPAKTIV | >= 30 minutes/day moderate/strenuous activity | 0 = no 1 = yes |
| Nutrition (fat) | YFETTREICH | low fat consumption (daily or several times per week) | 0 = no 1 = yes |
| Nutrition (fibre, fruit) | YFRUITFIBER | high fibre / fruit consumption (daily or several times per week) | 0 = no 1 = yes |
| Alcohol use | YALKOHOHL | At most 1 alcoholic drink per day | 0 = no 1 = yes |
| Tobacco use | YRAUCHEN | No current tobacco use | 0 = no 1 = yes |
| Injury prevention | YSICHERHE | Use of safety belts | 0 = no 1 = yes |
| | COMPBEHA | composite score for health behaviour [#2] | 0 ... 100 |

[#1] Definition COMPPREV
Percentage of the 6 preventive care recommendations, which were done
COMPREV = 100 * (number of favourable items(*) / number of items(*) answered and relevant(#))
if >= 2 responses missing then COMPPREV = missing (MR20%)
Range 0 ... 100
(*) items are
_BLUT01
_CHOLE01LE75
_GLUC01
_HAEMO01_LE80
_GRIPP01
_PNEUM01

(#) for persons with [75 <= AGE < 80] PC_Q5(cholesterol measurement) is not relevant
(#) for persons with [AGE  80 +] PC_Q5(cholesterol measurement) and PC_Q8(colon cancer screen) are not relevant

[#2] Definition COMPBEHA
Percentage of the 6 health behaviour recommendations, which were done
COMPBEHA = 100 * (number of favourable items(*) / number of items(*) answered and relevant(#))
if >= 2 responses missing then COMPBEHA = missing (MR20%)
Range 0 ... 100
(*) items are

- YKRPAKTIV (mod/stren sport or recreational activities less than 3+ days per week)
- YFETTREICH (high fat consumption)
- YFRUITFIBER (low fibre/fruit consumption)
- YRAUCHEN (tobacco use)
- YSICHERHE (non-use safety belts)
- YALKOHOHL (hazardous alcohol use)

## 5.2. Secondary outcomes

| | Variable Name | Variable Label | Value Label |
|---|---|---|---|
| **Secondary outcomes** | | | |
| Health-related problems | | | |
| Self-perceived health status | YGESUNDHE | good / very good self-perceived health | 0 = no 1 = yes |
| Functional status | YHILFEATL | No difficulty or need of assistance in >=1 ADL | 0 = no 1 = yes |
| | Systolic_GE160 | Systolic blood pressure >= 160 mm Hg | 0 = no 1 = yes |
| | CHOLHIGH_LE75 | Cholesterin >= 7 mmol/l (denom. = persons aged < 75 years) | 0 = no 1 = yes |
| | GLUCOHIGH | Glucose > 6.7 mmol/l | 0 = no 1 = yes |
| Survival and nursing home admission | | | |
| | MORT1224 | Survival at 2-year follow-up | 0 = no 1 = yes |
| | NRSH1224 | Nursing home admission at 2-year follow-up | 0 = no 1 = yes |

# Change Protocol:
# Document of Changes

# Statistical Analysis Plan

## Analytic plan PRO-AGE Solothurn
## (March 29, 2003)

| **Date** | **Chapter** (chapter No. of updated plan) | **Description of and Rationale for Change** | **Effects on other documents** |
|---|---|---|---|
| Sept 16, 2011 | 1 | Plan for eight-year follow-up:<br>For long-term follow-up, the members of the PRO-AGE Solothurn trial steering committee were Andreas Stuck, André Moser, Stephan Born, John C. Beck, and Matthias Egger. This committee searched for the availability of a potential source of information for obtaining long-term follow-up outcomes. This group identified the "Swiss National Cohort database" (SNC database) as a new potential source of information that had not been available before.<br>The PRO-AGE Solothurn trial steering committee decided to explore whether a permission could be obtained for linkage of the PRO-AGE Solothurn trial database with the SNC database. The steering committee agreed on a preliminary plan for data linkage<br>The following hypothesis was added:<br>For eight-year follow-up, we hypothesize that the intervention will increase survival | Study protocol |
| March 16, 2013 | 2.1. | Subgroup Analysis according to Pra risk status:<br>Exact cut-off should be used (according to Pacala et al.1997): Pra >0.2860 then PRA028=1 | no |
| March 16, 2013 | 2.2. | Analysis for effect in subgroups of persons who received site specific reinforcement:<br>This analysis is dropped because results are subject to bias (persons who accepted the preventive home may differ from the remaining persons in the intervention group). | no |
| March 16, 2013 | 5.1. | Use of composite variables for two-year outcomes:<br>The composite variables as defined in the initial version of the statistical plan do not add clinically meaningful information. Therefore, these variables are deleted and not used for analysis. | no |
| March 16, 2013 | 5.2. | Analysis of chart abstraction data revealed, that values for blood pressure, cholesterol, and glucose at two-year follow-up were not available from primary care practices in >30% of cases:<br>Blood pressure not available in 45.6% among surviving participants;<br>Cholesterol: not available in 47.6% among surviving participants aged <75;<br>Glucose: not available in 37.6% among surviving participants.<br>Therefore, these outcomes cannot be further analyzed. | no |
| March 16, 2013 | 5.2. | Secondary outcomes:<br>Self-perceived health status was recorded with five answer categories. All categories will be used for analysis, instead of using a dichotomous definition. | no |
| March 16, 2013 | 3.2., 6 | Eight-year follow-up analyses:<br>Detailed plan for survival analysis. | no |
| February 17, 2014 | 7 | Additional baseline variables:<br>Use of information from linkage with Swiss National Cohort (SNC) for definition of baseline variables. | no |

| | | | |
|---|---|---|---|
| February 17, 2014 | 3.2. | Sensitivity analysis: Repetition of Cox models for overall mortality with adjustment for baseline variables. | |
| February 17, 2014 | 3.1. | Imputation analyses for two-year follow-up:<br>For two-year follow-up analysis, due to missing data, use of complete case analysis is subject to bias. With the availability of methods for imputation, and the availability of additional base-line information from subjects (based on data linkage with the Swiss National Cohort), an imputation analysis based will be used as a main analysis. We will report imputed analyses as main analyses, and unadjusted complete case analyses as sensitivity analyses. | no |

# Analytic plan PRO-AGE Solothurn
# (March 29, 2003)
# *(updates according to change protocol, March 30, 2015)*

## 1. Main hypotheses

For two-year follow-up, we hypothesise that the intervention:

- results in a higher **uptake of preventive care** (colon cancer screening, breast cancer screening, influenza vaccination, pneumococcal vaccination, blood pressure measurement, glucose measurement, cholesterol measurement)

- results in favourable changes in **health behaviour** (higher level of physical activity, reduced fat intake, higher fruit/fibre intake, reduction of hazardous alcohol use, increase in seat belt use, reduction of smoking)

***Amendment, based on decision of steering committee Sept 16, 2011***

*For eight-year follow-up, we hypothesize that the intervention:*

- *will increase survival*

## 2. Main analyses: Comparison A follow-up with B follow-up

Comparison A follow-up (A_2) with B follow-up (B_2) unadjusted, taking into account the household as a cluster (because randomisation was by household) (definition of outcome variables: see table below).

### 2.1. Subgroup analysis: Stratified analyses according to Pra risk status

Study Subhypothesis:
The intervention effects will be more favourable among subjects at low risk (based on Pra)
The Pra instrument classifies persons into high risk (Pra: Probability of the Risk for Hospital Admission) (>0.28).

***Amendment, based on decision of steering committee March 16, 2013***
*Exact cut-off should be used for this subgroup analysis (according to Pacala et al.1997): Pra >0.2860.*

Analyses are unadjusted with household as cluster.
Variables:
PRA028 = 0 low risk for hospital admission
PRA028 = 1 high risk for hospital admission

### 2.2. Analysis for effect in subgroups of persons who received site specific reinforcement

Intervention effects in persons randomised to group A who acccepted the preventive home visits versus other persons in group A

***Amendment, based on decision of steering committee March 16, 2013***
*This subgroup analysis is dropped because results are subject to bias (persons who accepted the preventive home may differ from the remaining persons in the intervention group).*

# 3. Statistical methods

Statistical analyses will take into account any cluster effect from
a) randomisation by household and
b) GP practice

PRO-AGE data arise in a clustered way. In addition to person, sources of variation are physician practice and household.

Households need to be taken into account as clusters in any case. First, members of the same household tend to have more similar health behaviour than any two persons picked at random from the population. Second, household determines randomisation; in fact, households were the units randomised, not individual patients. Thus, conceptually, households are our unit of intervention/observation.

This can be done efficiently using the GEE-methodology (Generalized Estimating Equations), which allows to characterize patterns of subjects responses of a given cluster.

***Amendment, based on decision of steering committee Feb 17, 2014***

## *3.1. Two-year follow-up*

*For two-year follow-up analysis, due to missing data, an imputation analysis based on the following method will be used: all primary and secondary two-year outcomes will be analyzed by a multiple imputation approach using chained equations. The underlying population will consist of all surviving individuals living in the community. The imputation model will consist of baseline information (including SNC information), outcomes at two-year follow-up, and SNC-based survival information to make the missing at random assumption more plausible. Binary variables will be imputed by a logistic regression model. Continuous and ordered categorical variables will be imputed by predictive mean matching. We will use 25 complete datasets for reporting results.*

*We will report imputed analyses as main analyses, and unadjusted complete case analyses as sensitivity analyses.*

***Amendment for eight-year follow-up, based on decision of steering committee Feb 17, 2014***

## *3.2. Eight-year follow-up*

*Survival analysis will be unadjusted, investigating intervention versus control group.*
*Primary outcome: Eight year survival*
*Secondary outcome: Eight year survival by causes of death*
*Survival curves will be plotted using Kaplan-Meier estimates. We will explore the association of the intervention with all-cause mortality using a Cox proportional hazard model accounting for household membership, reporting hazard ratios. The underlying time scale will be time of randomization until Dec 31, 2008, or death. The proportional hazard assumption will be tested by Schoenfeld's test. Numbers needed to be treated (NNT) with confidence intervals will be calculated from absolute risk differences over the follow-up period. Exploratory analyses of cause of death will be conducted in case of survival difference, using the same methodology as in the main survival analyses.*
*Sensitivity analysis: Repetition of Cox models for primary outcome with adjustment for baseline variables*

# 4. Descriptive analyses

## 4.1. Descriptive analysis of intervention process

Determine the quantity of home visits that occurred during the two project years in SOLOTHURN.

## 4.2. Descriptive analysis of feed-back from physicians

Describe responses of physicians to questionnaire.

## 4.3. Descriptive analysis of feed-back from older persons

Describe feed-back of older person to questionnaire (based on base-line questionnaires, group A only).

# 5. Definition of outcome variables (two-year follow-up)

## 5.1. Primary outcomes

| | Variable Name | Variable Label | Value Label |
|---|---|---|---|
| **Primary outcomes** | | | |
| Preventive care use | | | |
| | _BLUT01 | blood pressure measurement in previous year | 1 = no 2 = yes |
| | _CHOLE01LE75 | cholesterol measurement in previous 5years (n = persons < 75 years) | 1 = no 2 = yes |
| | _GLUC01 | fasting blood glucose measurement in previous 12 months | 1 = no 2 = yes |
| | _HAEMO01_LE80 | colon cancer screen in previous year (n = persons < 80 years) | 1 = no 2 = yes |
| | _GRIPP01 | influenza immunisation in previous year | 1 = no 2 = yes |
| | _PNEUM01 | pneumococcal immunisation (ever) | 1 = no 2 = yes |
| Health behaviour | | | |
| Physical activity | YKRPAKTIV | >= 30 minutes/day moderate/strenuous activity | 0 = no 1 = yes |
| Nutrition (fat) | YFETTREICH | low fat consumption (daily or several times per week) | 0 = no 1 = yes |
| Nutrition (fibre, fruit) | YFRUITFIBER | high fibre / fruit consumption (daily or several times per week) | 0 = no 1 = yes |
| Alcohol use | YALKOHOHL | At most 1 alcoholic drink per day | 0 = no 1 = yes |
| Tobacco use | YRAUCHEN | No current tobacco use | 0 = no 1 = yes |
| Injury prevention | YSICHERHE | Use of safety belts | 0 = no 1 = yes |

***Amendment, based on decision of steering committee March 16, 2013***
*Use of composite variables is dropped from initial version of above list.*

## 5.2. Secondary outcomes

| | Variable Name | Variable Label | Value Label |
|---|---|---|---|
| **Secondary outcomes** | | | |
| Health-related problems | | | |
| Self-perceived health status | YGESUNDHE | *Five categories* | *1 = poor*<br>*2= fair*<br>*3= good*<br>*4= very good*<br>*5= excellent* |
| Functional status | YHILFEATL | No difficulty or need of assistance in >=1 ADL | 0 = no 1 = yes |
| Survival and nursing home admission | | | |
| | MORT1224 | Survival at 2-year follow-up | 0 = no 1 = yes |
| | NRSH1224 | Nursing home admission at 2-year follow-up | 0 = no 1 = yes |

***Amendment, based on decision of steering committee March 16, 2013***
*Definition of self-report health based on all five categories.*
*Use of measurement values (blood pressure, cholesterol, glucose) is dropped from initial version of above list*

***<u>Amendment, based on decision of steering committee Mar 16, 2013</u>***

# *6. Definition of outcome variables (eight-year follow-up)*

## *6.1. Primary outcomes*

*Survival (number of days survived until end of eight-year follow-up or until Dec 31, 2008*

## *6.2. Secondary outcomes*

***Cause of death***
***According to ICD 10 classification:***
***Circulatory system (category I)***
- *Ischemic heart disease (I20-I25)*
- *Hypertensive diseases (I10-I15)*
- *Stroke (I64)*

***Neoplasm (category C)***
- *Respiratory (C30-C39)*
- *Digestive (C15-C26)*
- *Gynecological (C50-C58)*

***Other and unknown***

***<u>Amendment, based on decision of steering committee Feb 17, 2014</u>***

## *7. Derivation of socio-demographic information from database*

*Sociodemographic information was derived from the Swiss National Cohort (SNC) database [Bopp 2008, Spörri 2010], including information on education, marital status, religious affiliation, living arrangement, and socio-economic position (Swiss-SEP), an index ranging from 0-100 [Panczak, 2012]. The Swiss-SEP was developed based on the median rent per square meter, the proportion of households headed by a person with primary education or less, the proportion headed by a person in manual or unskilled occupation and the mean number of persons per room. The Swiss-SEP is strongly associated with household income and some causes of death [Panczak, 2012]. It is an index ranging from 0-100 derived from the median rent per square meter, the proportion of households headed by a person with primary education or less, the proportion headed by a person in manual or unskilled occupation and the mean number of persons per room. Higher values denote higher socio-economic position.*
