# Supplementary material for: Effect of Health Risk Assessment and Counselling on Health Behaviour and Survival in Older People: A Pragmatic Randomised Trial
Source: PLoS Med. 2015 Oct 19;12(10):e1001889. doi: 10.1371/journal.pmed.1001889 (PMC4610679; doi:10.1371/journal.pmed.1001889)
Supplement: S4 Text — (PDF) [file pmed.1001889.s016.pdf]

Supporting Information S4 Text

Health Risk Appraisal for Older Persons (HRA-O) questionnaire (German language version)

for: A Collaborative Care Model of Health Risk Assessment and Counselling in Older Persons: A Randomised Clinical Trial

Gesundheits-Profil
Fragebogen

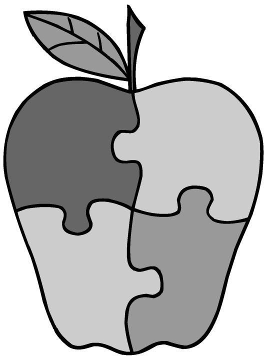

Bitte kontrollieren Sie Ihre persönlichen Angaben auf den aufgeklebten Etiketten. Die nachfolgenden Zeilen dienen dazu, fehlerhafte oder veraltete Angaben zu berichtigen.

____

____

____

Füllen Sie bitte Ihren Fragebogen zum Gesundheits-Profil aus, damit Sie Ihren vertraulich behandelten und persönlichen Gesundheits-Bericht erhalten. Ihr Bericht wird Sie über Ihr gesundheitliches Befinden hinsichtlich Ihrer Ernährung, Ihrer Risiken, der Verwendung von Medikamenten und der Gesundheitsvorsorge informieren.

Denken Sie daran ...
- *Lesen Sie jede Frage aufmerksam durch und befolgen Sie alle Anweisungen.*
- *Kreuzen Sie Kästchen folgendermassen an:* [X]
- *Tragen Sie Ihre Antworten folgendermassen in Druckschrift ein:* [H][A][N][S]
- *Schreiben Sie bitte keine Bemerkungen in die Zwischenräume und an die Ränder – sie können nicht berücksichtigt werden.*
- *Beantworten Sie die Fragen zu allen Abschnitten und Einzelpunkten, auch wenn das angesprochene Thema Sie nicht direkt betrifft.*
- *Lassen Sie keine Fragen aus, auch wenn Ihre Antwort "Nein" lautet.*
- *Achten Sie darauf, dass Sie jede Seite ausfüllen.*

© 2000 Dieser Fragebogen ist urheberrechtlich geschützt.

Medizinische Vorgeschichte

Es folgt eine Liste von ausgewählten Krankheitszuständen, die sich gewöhnlich über längere Zeit auswirken.

**Hat Ihnen jemals ein Arzt / eine Ärztin gesagt, Sie hätten ...**<br>***Bitte beantworten Sie jede Frage mit Nein oder Ja***		**Nein**	**Ja**
1.	... hohen Blutdruck?	☐1	☐2
2.	... eine koronare Herzkrankheit (Angina pectoris oder Herzinfarkt)?	☐1	☐2
3.	... eine Herzschwäche (Herzinsuffizienz)?	☐1	☐2
4.	... einen unregelmässigen Puls (Arrhythmien)?	☐1	☐2
5.	... einen Schlaganfall (Gehirnschlag)?	☐1	☐2
6.	... eine chronische Bronchitis?	☐1	☐2
7.	... Asthma?	☐1	☐2
8.	... eine Gelenkarthrose oder sonstige rheumatische Erkrankungen?	☐1	☐2
9.	... Osteoporose?	☐1	☐2
10.	... eine Zuckerkrankheit (Diabetes mellitus)?	☐1	☐2
11.	... eine Depression?	☐1	☐2
12.	... eine andere seelische Erkrankung, ausgenommen Depression?	☐1	☐2
13.	... grünen Star (Glaukom, Star wegen hohem Augendruck)?	☐1	☐2
14.	... eine Netzhauterkrankung des Auges?	☐1	☐2
15.	... grauen Star (Katarakt, Star wegen Linsentrübung)?	☐1	☐2

© 2000 Dieser Fragebogen ist urheberrechtlich geschützt.

Masse und gesundheitliche Daten

1. Ihre ungefähre Körpergrösse in Meter (m) und Zentimeter (cm) ohne Schuhe?

   ☐ m ☐☐ cm

2. Ihr ungefähres Körpergewicht in Kilogramm (kg), in leichter Kleidung ohne Schuhe?

   ☐☐☐ kg

3. Haben Sie in den letzten sechs Monaten unbeabsichtigt 5 kg oder mehr abgenommen?
   - ☐ 1 Nein
   - ☐ 2 Ja

4. Haben Sie in den letzten sechs Monaten unbeabsichtigt 5 kg oder mehr zugenommen?
   - ☐ 1 Nein
   - ☐ 2 Ja

5. Wie hoch ist Ihr Blutdruck?

   ☐☐☐ (Systolisch) oberer Wert ☐☐☐ (Diastolisch) unterer Wert

   - ☐ 1 Ich weiss es nicht / ich bin nicht sicher

6. Wie hoch ist Ihr Gesamtcholesterinspiegel? ☐☐.☐
   - ☐ 1 Ich weiss es nicht / ich bin nicht sicher

© 2000 Dieser Fragebogen ist urheberrechtlich geschützt.

Medikamente

	Geben Sie an, ob Sie <u>zur Zeit</u> folgende Medikamente verwenden:	Nein	Ja
1.	Medikamente gegen Herzkrankheiten	☐ 1	☐ 2
2.	Medikamente gegen hohen Blutdruck	☐ 1	☐ 2
3.	Medikamente gegen hohe Cholesterinwerte	☐ 1	☐ 2
4.	Spritzen oder Tabletten gegen Zuckerkrankheit (Diabetes mellitus)	☐ 1	☐ 2
5.	Schlafmittel (3 mal oder mehr pro Woche)	☐ 1	☐ 2
6.	Medikamente gegen Angst (schlechte Nerven oder Nervosität)	☐ 1	☐ 2
7.	Medikamente gegen depressive Zustände oder Traurigkeit	☐ 1	☐ 2
8.	Medikamente zum Ersatz von weiblichen Hormonen (Nur für Frauen)	☐ 1	☐ 2
9.	Medikamente gegen Arthrose oder andere Gelenksbeschwerden (3 mal oder mehr pro Woche)	☐ 1	☐ 2
10.	Regelmässig Alcacyl, Aspirin, oder Tiatral zur Vorbeugung gegen Durchblutungsstörungen	☐ 1	☐ 2

	Haben Sie <u>in den letzten 4 Wochen</u>, einmal oder mehrmals, eines der folgenden Medikamente eingenommen?	Nein	Ja
11.	Dalmadorm, Demetrin, Lexotanil, Rohypnol, Tranxilium oder Valium?	☐ 1	☐ 2
12.	Librax, Limbitrol oder Psychopax?	☐ 1	☐ 2
13.	Saroten oder Tryptizol?	☐ 1	☐ 2

14. Wie viele <u>verschiedene</u> Medikamente *(Tabletten, Tropfen, Pflaster, Salben, etc.)* verwenden Sie <u>zur Zeit</u>? *Einschliesslich derjenigen, bei denen Sie oben mit JA geantwortet haben.* ***(Tragen Sie 0 ein, wenn Sie keine Medikamente verwenden.)***

☐☐ Anzahl rezeptpflichtige Medikamente

☐☐ Anzahl nicht rezeptpflichtige Medikamente

☐☐ Anzahl pflanzliche Heilmittel

15. Haben Sie festgestellt, dass bei Ihnen Nebenwirkungen durch Ihre Medikamente auftreten?

☐ 1 Nein
☐ 2 Ja

© 2000 Dieser Fragebogen ist urheberrechtlich geschützt.

16. Es ist oft nicht einfach, Medikamente stets genau gemäss Verordnung zu verwenden. Was für Schwierigkeiten hatten Sie schon dabei?

Mehrere Antworten möglich. ***Kreuzen Sie bitte alle zutreffenden Antworten an.***

a. ☐1 Diese Frage trifft auf mich nicht zu, mir wurden keine Medikamente verordnet
b. ☐1 Ich nehme die Medikamente genau so, wie sie verordnet sind
c. ☐1 Ich vergesse es manchmal oder denke nicht daran
d. ☐1 Ich finde es nicht so wichtig
e. ☐1 Ich habe keine Zeit dazu
f. ☐1 Kosten
g. ☐1 Nebenwirkungen
h. ☐1 Schwierigkeiten mit der Handhabung/Verpackung

© 2000 Dieser Fragebogen ist urheberrechtlich geschützt.

Anzeichen und Symptome

Bitte berücksichtigen Sie bei Ihren Angaben nur Anzeichen und Symptome, die Sie während des letzten Monats verspürt haben.

**Geben Sie an, wie häufig Sie während des letzten Monats *(ungeachtet der Ursache)* durch folgende Symptome beeinträchtigt wurden:**		**Nie**	**Ab und zu**	**Oft/ immer**
1.	Fieber	☐1	☐3	☐5
2.	Unerklärliche Erschöpfung/Schwäche	☐1	☐3	☐5
3.	Unfähigkeit klar zu denken	☐1	☐3	☐5
4.	Alpträume	☐1	☐3	☐5
5.	Depression/Niedergeschlagenheit	☐1	☐3	☐5
6.	Angst/Erregungszustand	☐1	☐3	☐5
7.	Verschwommenes Sehen *(trotz Brille oder Kontaktlinsen)*	☐1	☐3	☐5
8.	Verstopfte Nase	☐1	☐3	☐5
9.	Ungewohnter Mundgeschmack	☐1	☐3	☐5
10.	Mundtrockenheit	☐1	☐3	☐5
11.	Unerklärliche Schläfrigkeit/Müdigkeit	☐1	☐3	☐5
12.	Nächtliche Kurzatmigkeit	☐1	☐3	☐5
13.	Kurzatmigkeit bei geringer Anstrengung	☐1	☐3	☐5
14.	Geschwollene Beine	☐1	☐3	☐5
15.	Herzklopfen oder unregelmässiger Puls	☐1	☐3	☐5
16.	Schwindel beim Aufstehen	☐1	☐3	☐5
17.	Ohnmachtsanfälle	☐1	☐3	☐5
18.	Schmerzen in der Brust	☐1	☐3	☐5
19.	Husten	☐1	☐3	☐5
20.	Verstopfung	☐1	☐3	☐5
21.	Durchfall (Diarrhö)	☐1	☐3	☐5

© 2000 Dieser Fragebogen ist urheberrechtlich geschützt.

	Geben Sie an, wie häufig Sie während des letzten Monats *(ungeachtet der Ursache)* durch folgende Symptome beeinträchtigt wurden:	Nie	Ab und zu	Oft/ immer
22.	Übelkeit	☐1	☐3	☐5
23.	Bauchschmerzen	☐1	☐3	☐5
24.	Sodbrennen *(saures Aufstossen)*	☐1	☐3	☐5
25.	Schwierigkeiten bei der Blasenentleerung	☐1	☐3	☐5
26.	Mehr als zweimalige Blasenentleerung in der Nacht	☐1	☐3	☐5
27.	Häufiger Harndrang	☐1	☐3	☐5
28.	Hautausschlag	☐1	☐3	☐5
29.	Juckreiz	☐1	☐3	☐5
30.	Äusserliche Druckempfindlichkeit der Brust	☐1	☐3	☐5
31.	Beinschmerzen bei Anstrengung	☐1	☐3	☐5
32.	Unerklärliche Muskelschmerzen	☐1	☐3	☐5
33.	Sexuelle Probleme	☐1	☐3	☐5
34.	Kopfschmerzen	☐1	☐3	☐5
35.	Kalte oder schmerzende Hände oder Füsse	☐1	☐3	☐5

Beherrschung der Blase

Schwierigkeiten mit der Beherrschung der Blase sind im Alter ein häufiges Problem. Es kann z.B. vorkommen, dass beim Husten oder Niesen kleinere Urinmengen abgehen. In vielen Fällen kann aber durch geeignete Massnahmen eine Verbesserung erzielt werden.

36a. Ist Ihnen in den letzten 12 Monaten jemals unkontrolliert etwas Urin abgegangen?

☐1 Nein → *Fahren Sie auf Seite 8 (Schmerzen) fort*

☐2 Ja ↓

36b. Ist Ihnen das in den letzten 12 Monaten an mindestens sechs verschiedenen Tagen passiert?

☐1 Nein

☐2 Ja

© 2000 Dieser Fragebogen ist urheberrechtlich geschützt.

Schmerzen

1. Hatten Sie **in den letzten 4 Wochen** Schmerzen?
   - ☐1 Nein → *Fahren Sie auf Seite 10 (Zahn- und Mundhygiene) fort*
   - ☐2 Ja

In den nächsten zwei Fragen wird eine Skala verwendet, um die Stärke Ihrer Schmerzen zu erfassen. ***0*** *bedeutet kein Schmerz,* ***10*** *der schlimmste vorstellbare Schmerz.*
Kreuzen Sie die Zahl an, die Ihrer Schmerzstärke entspricht.

2. Wie stark sind Ihre Schmerzen **heute**?

**0**	**1**	**2**	**3**	**4**	**5**	**6**	**7**	**8**	**9**	**10**
☐0	☐1	☐2	☐3	☐4	☐5	☐6	☐7	☐8	☐9	☐10

kein Schmerz … schlimmster Schmerz

3. Wie stark waren Ihre Schmerzen **durchschnittlich in der letzten Woche**?

**0**	**1**	**2**	**3**	**4**	**5**	**6**	**7**	**8**	**9**	**10**
☐0	☐1	☐2	☐3	☐4	☐5	☐6	☐7	☐8	☐9	☐10

kein Schmerz … schlimmster Schmerz

Lesen Sie die folgenden Fragen zum Thema Schmerz aufmerksam durch und wählen Sie jeweils die Antwort, welche für Sie am ehesten zutrifft.

		**Nein**	**Ja**
4.	Verspüren Sie jeden Tag Schmerzen?	☐1	☐2
5.	Haben Sie mehrmals pro Woche Schmerzen?	☐1	☐2
6.	Haben Sie Schmerzen, die nie ganz verschwinden?	☐1	☐2
7.	Nehmen Sie Schmerzmittel ein (3 mal oder mehr pro Woche)?	☐1	☐2
8.	Waren Sie in der letzten Woche wegen Ihrer Schmerzen traurig oder niedergeschlagen?	☐1	☐2

© 2000 Dieser Fragebogen ist urheberrechtlich geschützt.

**Bereiten Ihnen die folgenden Tätigkeiten Schmerzen?** *Wenn Sie die Tätigkeit zur Zeit nicht ausüben, beantworten Sie die Frage als würden Sie die Tätigkeit ausüben*		**Nein**	**Ja**
9.	An- und Auskleiden, ein Bad nehmen	☐1	☐2
10.	Volle Einkaufstaschen tragen	☐1	☐2
11.	Weniger als 200m zu Fuss gehen	☐1	☐2
12.	Mehr als 200m zu Fuss gehen	☐1	☐2
13.	Einige Treppenstufen steigen	☐1	☐2
14.	Mehr als ein Stockwerk Treppen steigen	☐1	☐2
15.	Körperlich mittelschwer belastende Tätigkeiten, *wie Hausarbeiten oder leichter Sport*	☐1	☐2
16.	Körperlich stark belastende Tätigkeiten, *wie Heben schwerer Gegenstände, Rennen oder intensiver Sport*	☐1	☐2

**Hindert Sie Schmerz daran,…**		**Nein**	**Ja**
17.	... Verkehrsmittel (Auto, Bahn, Bus) zu benutzen?	☐1	☐2
18.	... an gemeinschaftlichen Aktivitäten *(z.B. Theater, Konzert, Gottesdienst)* teilzunehmen?	☐1	☐2
19.	... gemeinsam mit Angehörigen oder Freunden etwas zu unternehmen?	☐1	☐2

**Sind Schmerzen die Ursache dafür, dass …**		**Nein**	**Ja**
20.	... Sie eine Arbeit bzw. andere Tätigkeit weniger oft oder weniger lange ausführen können?	☐1	☐2
21.	... Sie weniger leistungsfähig sind, als Sie es sich wünschten?	☐1	☐2
22.	... Sie Arbeiten oder andere Tätigkeiten aufgeben mussten?	☐1	☐2
23.	... Sie Arbeiten oder andere Tätigkeiten mehr Anstrengung kosten?	☐1	☐2
24.	... Sie Schlafprobleme haben?	☐1	☐2
25.	... Sie sich müde und erschöpft fühlen?	☐1	☐2
26.	... Sie Hilfe von Familienangehörigen und Freunden benötigen?	☐1	☐2

© 2000 Dieser Fragebogen ist urheberrechtlich geschützt.

Zahn- und Mundhygiene

1. Tragen Sie abnehmbare Zahnprothesen (Teil- oder Totalprothesen)?
   - ☐1 Nein
   - ☐2 Ja

In den <u>vergangenen 3 Monaten</u>, wie oft..		Nie	Selten	Manch-mal	Häufig	Immer
2.	… haben Sie sich aufgrund von Zahnproblemen in der Art oder Menge Ihrer Ernährung eingeschränkt?	☐1	☐2	☐3	☐4	☐5
3.	… hatten Sie Schwierigkeiten, Lebensmittel zu beissen oder zu kauen, *wie z.B. festes Fleisch oder Äpfel?*	☐1	☐2	☐3	☐4	☐5
4.	*Bitte beachten Sie die Fragestellung* … konnten Sie Speisen und Getränke **<u>gut</u>** schlucken?	☐1	☐2	☐3	☐4	☐5
5.	… wurden Sie durch Ihre Zähne (bzw. Ihre Zahnprothese) am normalen Sprechen behindert?	☐1	☐2	☐3	☐4	☐5
6.	*Bitte beachten Sie die Fragestellung* … konnten Sie **<u>alles</u>** essen, ohne dabei Beschwerden zu haben?	☐1	☐2	☐3	☐4	☐5
7.	… mieden Sie den Kontakt mit anderen Menschen wegen des Zustandes Ihrer Zähne (oder Ihrer Zahnprothese)?	☐1	☐2	☐3	☐4	☐5
8.	*Bitte beachten Sie die Fragestellung* … waren Sie **<u>glücklich oder zufrieden</u>** mit dem Aussehen Ihrer Zähne und Ihres Zahnfleisches (bzw. Ihrer Zahnprothese)?	☐1	☐2	☐3	☐4	☐5
9.	… nahmen Sie Medikamente, die Schmerzen oder Beschwerden im Mundbereich lindern sollten?	☐1	☐2	☐3	☐4	☐5
10.	… waren Sie wegen Ihrer Zähne, Ihres Zahnfleisches (oder Ihrer Zahnprothese) in Sorge?	☐1	☐2	☐3	☐4	☐5
11.	… fühlten Sie sich aufgrund von Problemen mit Ihren Zähnen, Ihrem Zahnfleisch (oder Ihrer Zahnprothese) nervös oder befangen?	☐1	☐2	☐3	☐4	☐5
12.	… war es Ihnen unangenehm, wegen Problemen mit Ihren Zähnen (oder Ihrer Zahnprothese) im Beisein anderer Menschen zu essen?	☐1	☐2	☐3	☐	☐5
13.	… reagierten Ihre Zähne oder Ihr Zahnfleisch empfindlich auf "heiss", "kalt" oder "süss"?	☐1	☐2	☐3	☐4	☐5

© 2000 Dieser Fragebogen ist urheberrechtlich geschützt.

Sehen

Im Folgenden möchten wir Sie bitten, Ihr Sehvermögen zu beschreiben. Hinweis für Brillen- oder Kontaktlinsenträger: Beantworten Sie die Fragen bitte so, wie Sie mit der Sehhilfe sehen. Wählen Sie zu jeder Frage die Antwort, die Ihre Situation am besten beschreibt.

1. Wie beurteilen Sie zur Zeit Ihr Sehvermögen mit beiden Augen (*gegebenenfalls mit Brille oder Kontaktlinsen*)?
   - ☐1 Ausgezeichnet → *Fahren Sie auf Seite 14 (Hören) fort*
   - ☐2 Gut
   - ☐3 Einigermassen
   - ☐4 Schlecht
   - ☐5 Sehr schlecht
   - ☐6 Befragte Person ist blind *(Fragebogen wird durch Bezugsperson ausgefüllt)*

2. Wie oft machen Sie sich Sorgen um Ihr Augenlicht?
   - ☐1 Nie
   - ☐2 Selten
   - ☐3 Manchmal
   - ☐4 Häufig
   - ☐5 Immer

3. Haben Sie in letzter Zeit eine Verschlechterung Ihres Sehvermögens festgestellt?
   - ☐1 Nein
   - ☐2 Ja

Die folgenden Punkte fragen nach den Schwierigkeiten, die es Ihnen bereitet, bestimmte Tätigkeiten auszuüben (gegebenenfalls mit Brille oder Kontaktlinsen).

4. Wie schwer fällt es Ihnen, normale Schrift in den Zeitungen zu lesen?
   - ☐1 Überhaupt keine Schwierigkeiten
   - ☐2 Leichte Schwierigkeiten
   - ☐3 Ziemliche Schwierigkeiten
   - ☐4 Äusserste Schwierigkeiten
   - ☐5 Aufgehört wegen eingeschränktem Sehvermögen
   - ☐6 Aufgehört aus anderen Gründen

5. Wie schwer fällt es Ihnen, Arbeiten oder Hobbys auszuüben, bei denen Sie auf kurze Entfernung gut sehen müssen, *wie etwa Schreiben, Kreuzworträtsel lösen, Karten- und Gesellschaftsspiele, Nähen oder die Verwendung von Werkzeugen?*
   - ☐1 Überhaupt keine Schwierigkeiten
   - ☐2 Leichte Schwierigkeiten
   - ☐3 Ziemliche Schwierigkeiten
   - ☐4 Äusserste Schwierigkeiten
   - ☐5 Aufgehört wegen eingeschränktem Sehvermögen
   - ☐6 Aufgehört aus anderen Gründen

© 2000 Dieser Fragebogen ist urheberrechtlich geschützt.

6. Wie schwer fällt es Ihnen, bei schwachem Licht oder nachts Stufen oder Treppen hinunterzugehen?
   - ☐1 Überhaupt keine Schwierigkeiten
   - ☐2 Leichte Schwierigkeiten
   - ☐3 Ziemliche Schwierigkeiten
   - ☐4 Äusserste Schwierigkeiten
   - ☐5 Aufgehört wegen eingeschränktem Sehvermögen
   - ☐6 Aufgehört aus anderen Gründen

7. Wie schwer fällt es Ihnen, beim Vorwärtsgehen auch Dinge rechts und links wahrzunehmen (unerwartete Hindernisse, Gefahren)?
   - ☐1 Überhaupt keine Schwierigkeiten
   - ☐2 Leichte Schwierigkeiten
   - ☐3 Ziemliche Schwierigkeiten
   - ☐4 Äusserste Schwierigkeiten
   - ☐5 Aufgehört wegen eingeschränktem Sehvermögen
   - ☐6 Aufgehört aus anderen Gründen

8. Wie schwer fällt es Ihnen, aufgrund von Sehproblemen etwas in einem vollen Regal zu finden?
   - ☐1 Überhaupt keine Schwierigkeiten
   - ☐2 Leichte Schwierigkeiten
   - ☐3 Ziemliche Schwierigkeiten
   - ☐4 Äusserste Schwierigkeiten
   - ☐5 Aufgehört wegen eingeschränktem Sehvermögen
   - ☐6 Aufgehört aus anderen Gründen

9. Müssen Sie aufgrund von Sehproblemen Ihre Arbeit oder sonstigen Tätigkeiten vorzeitig abbrechen?
   - ☐1 Nie
   - ☐2 Selten
   - ☐3 Manchmal
   - ☐4 Häufig
   - ☐5 Immer

© 2000 Dieser Fragebogen ist urheberrechtlich geschützt.

Jetzt stellen wir Ihnen ein paar Fragen zum Thema Autofahren.

10a. Fahren Sie selber Auto, sei dies regelmässig oder nur hin und wieder?

- ☐1 Ja → *Fahren Sie bei Frage 11 auf dieser Seite fort*
- ☐2 Nein

10b. Sind Sie nie selber Auto gefahren oder haben Sie das Autofahren aufgegeben?

- ☐1 Ich bin nie selbst Auto gefahren → *Fahren Sie auf Seite 14 (Hören) fort*
- ☐2 Ich habe das Autofahren aufgegeben ↓

10c. Warum haben Sie das Autofahren aufgegeben?

- ☐1 Hauptsächlich wegen meiner Sehfähigkeit
- ☐2 Hauptsächlich aus anderen Gründen
- ☐3 Aus beiden obengenannten Gründen

→ *Fahren Sie auf Seite 14 (Hören) fort*

Diese Frage richtet sich nur an Personen, welche selber Auto fahren.

11. Wie schwer fällt Ihnen das Autofahren bei Tag in bekannter Umgebung?

- ☐1 Keinerlei Schwierigkeiten
- ☐2 Wenig Schwierigkeiten
- ☐3 Ziemliche Schwierigkeiten
- ☐4 Grösste Schwierigkeiten

© 2000 Dieser Fragebogen ist urheberrechtlich geschützt.

Hören

Die folgenden Fragen ermitteln, ob Sie aufgrund von Hörproblemen Schwierigkeiten haben. Hinweis für Personen mit Hörgerät: beantworten Sie die Fragen bitte so, wie Sie mit Hörgerät hören.

1. Wie würden Sie Ihr Hörvermögen einstufen (*gegebenenfalls mit Ihrem Hörgerät*)?
   - ☐1 Ausgezeichnet → *Fahren Sie auf Seite 15 (Psyche und Wohlbefinden) fort*
   - ☐2 Gut
   - ☐3 Mittelmässig
   - ☐4 Schlecht
   - ☐5 Sehr schlecht
   - ☐6 Gehörlos

	**Verursachen eventuelle Hörschwierigkeiten, dass ...**	**Nein**	**Gele-gentlich**	**Ja**
2.	... Sie verlegen sind, wenn Sie Leute kennenlernen?	☐1	☐2	☐3
3.	... Sie sich hilflos fühlen, wenn Sie sich mit Ihren Familienangehörigen unterhalten?	☐1	☐2	☐3
4.	... Sie Schwierigkeiten haben, wenn Sie Freunde, Verwandte oder Nachbarn besuchen?	☐1	☐2	☐3
5.	... Sie seltener in den Gottesdienst gehen oder einen Theaterbesuch machen, als Sie dies eigentlich gern tun würden?	☐1	☐2	☐3
6.	... mit Familienangehörigen Missverständnisse auftreten?	☐1	☐2	☐3
7.	... Sie Schwierigkeiten beim Radiohören oder Fernsehen haben?	☐1	☐2	☐3
8.	... Sie Schwierigkeiten haben, wenn Sie mit Verwandten oder Freunden im Restaurant sind?	☐1	☐2	☐3
9.	... Sie Verständnisprobleme haben, wenn jemand flüstert?	☐1	☐2	☐3
10.	... Sie sich dadurch behindert fühlen?	☐1	☐2	☐3
11.	... Ihr persönlicher oder gesellschaftlicher Umgang eingeschränkt oder erschwert wird?	☐1	☐2	☐3

12a. Tragen Sie ein Hörgerät?
   - ☐1 Nein → *Fahren Sie auf Seite 15 (Psyche und Wohlbefinden) fort*
   - ☐2 Ja

12b. Wie oft wechseln Sie durchschnittlich die Batterien Ihres Hörgeräts?
   - ☐1 Alle 1-2 Wochen oder öfter
   - ☐2 Alle 3-4 Wochen
   - ☐3 Einmal im Monat oder seltener

© 2000 Dieser Fragebogen ist urheberrechtlich geschützt.

Psyche und Wohlbefinden

Bei Frage 1 bis 5 geht es darum, wie Sie sich während des letzten Monats gefühlt haben.

**Wie lange haben Sie sich <u>während des letzten Monats ...</u>**		**Nie**	**Selten**	**Manch-mal**	**Oft**	**Fast immer**	**Immer**
1.	... ausgeglichen und zufrieden gefühlt?	☐1	☐2	☐3	☐4	☐5	☐6
2.	... unruhig oder nervös gefühlt?	☐1	☐2	☐3	☐4	☐5	☐6
3.	... so deprimiert gefühlt, dass Sie nichts mehr aufheitern konnte?	☐1	☐2	☐3	☐4	☐5	☐6
4.	... niedergeschlagen und traurig gefühlt?	☐1	☐2	☐3	☐4	☐5	☐6
5.	... glücklich gefühlt?	☐1	☐2	☐3	☐4	☐5	☐6

6. Falls Sie sich deprimiert oder niedergeschlagen fühlen, gibt es für Sie zur Zeit einen besonderen Grund dazu, *beispielsweise dass Sie kürzlich eine geliebte Person verloren haben oder dass Sie kürzlich umziehen mussten?*

☐1 Nein ☐3 Diese Frage trifft für mich nicht zu
☐2 Ja

Wählen Sie bitte die <u>zutreffendste</u> Antwort auf jede der folgenden Fragen zu Ihrem Gedächtnis:		**Fast nie**	**Manch-mal**	**Oft**	**Fast immer**
7.	Können Sie sich erinnern, welche Kleidung Sie gestern anhatten?	☐1	☐2	☐3	☐4
8.	Erinnern Sie sich gewöhnlich in einem Kaufhaus, welchen Eingang Sie benutzt haben?	☐1	☐2	☐3	☐4
9.	Können Sie sich erinnern, wo Sie sich verabredet haben oder wo Sie Ihr Auto geparkt haben?	☐1	☐2	☐3	☐4
10.	Können Sie im Dunkeln Ihren Autoschlüssel oder Briefkastenschlüssel von Ihrem Hausschlüssel unterscheiden?	☐1	☐2	☐3	☐4
11.	Erinnern Sie sich, wo Sie Ihre Brille oder Ihre Schlüssel gelassen haben?	☐1	☐2	☐3	☐4
12.	Wenn Sie jemand anruft, können Sie ihm oder ihr den Weg zu Ihrem Haus beschreiben?	☐1	☐2	☐3	☐4
13.	Wenn Sie eine Fernsehserie oder eine Radioserie verfolgen, können Sie sich erinnern, was in der letzten Folge geschah?	☐1	☐2	☐3	☐4
14.	Können Sie beschreiben, was Sie am letzten Sonntagnachmittag gemacht haben?	☐1	☐2	☐3	☐4
15.	Können Sie eine Melodie nachsummen, wenn Sie sie einige Male gehört haben?	☐1	☐2	☐3	☐4
16.	Können Sie Geräte, die Sie zum Reinigen auseinandergenommen haben, wieder zusammensetzen (*z. B. Kaffeemaschine, Rasierer*)?	☐1	☐2	☐3	☐4

© 2000 Dieser Fragebogen ist urheberrechtlich geschützt.

Soziales Umfeld

Unser soziales Umfeld bietet uns Gesellschaft und Freizeitkontakte, aber auch Hilfe und Unterstützung in ernsten Situationen.

*Zunächst fragen wir nach Kontakten zu **Familienangehörigen** und **Verwandten** (Ehepartner, Kinder, Enkelkinder, Verschwägerte und andere Verwandte).*

1. Mit wie vielen Familienangehörigen treffen Sie sich mindestens einmal im Monat oder haben Sie mindestens einmal im Monat sonstigen Kontakt (z.B. telefonisch)?

   - ☐ 1 9 oder mehr Personen
   - ☐ 2 5 bis 8 Personen
   - ☐ 3 3 oder 4 Personen
   - ☐ 4 2 Personen
   - ☐ 5 1 Person
   - ☐ 6 Keine

2. Mit wie vielen Familienangehörigen sind Sie so vertraut, dass Sie sie um Unterstützung bitten können?

   - ☐ 1 9 oder mehr Personen
   - ☐ 2 5 bis 8 Personen
   - ☐ 3 3 oder 4 Personen
   - ☐ 4 2 Personen
   - ☐ 5 1 Person
   - ☐ 6 Keine

3. Mit wie vielen Familienangehörigen sind Sie so vertraut, dass Sie private Angelegenheiten mit ihnen besprechen können?

   - ☐ 1 9 oder mehr Personen
   - ☐ 2 5 bis 8 Personen
   - ☐ 3 3 oder 4 Personen
   - ☐ 4 2 Personen
   - ☐ 5 1 Person
   - ☐ 6 Keine

*Jetzt fragen wir nach Kontakten zu **Freunden** und **Nachbarn**.*

4. Mit wie vielen Freunden oder Nachbarn treffen Sie sich mindestens einmal im Monat oder haben Sie mindestens einmal im Monat sonstigen Kontakt (z.B. telefonisch)?

   - ☐ 1 9 oder mehr Personen
   - ☐ 2 5 bis 8 Personen
   - ☐ 3 3 oder 4 Personen
   - ☐ 4 2 Personen
   - ☐ 5 1 Person
   - ☐ 6 Keine

5. Mit wie vielen Freunden oder Nachbarn sind Sie so vertraut, dass Sie sie um Hilfe bitten können?

   - ☐ 1 9 oder mehr Personen
   - ☐ 2 5 bis 8 Personen
   - ☐ 3 3 oder 4 Personen
   - ☐ 4 2 Personen
   - ☐ 5 1 Person
   - ☐ 6 Keine

6. Mit wie vielen Freunden oder Nachbarn sind Sie so vertraut, dass Sie private Angelegenheiten mit ihnen besprechen können?

   - ☐ 1 9 oder mehr Personen
   - ☐ 2 5 bis 8 Personen
   - ☐ 3 3 oder 4 Personen
   - ☐ 4 2 Personen
   - ☐ 5 1 Person
   - ☐ 6 Keine

© 2000 Dieser Fragebogen ist urheberrechtlich geschützt.

7. Wie oft ist jemand (Partner, Angehörige, Freunde etc) da, welcher Ihnen Freundschaft und Zuneigung zeigt, wenn Sie es brauchen?

☐1 Immer	☐4 Manchmal
☐2 Meistens	☐5 Selten
☐3 Oft	☐6 Nie

8. Wie oft ist jemand (Partner, Angehörige, Freunde etc) da, welcher mit Ihnen Ihre intimsten Sorgen und Ängste teilt, wenn Sie es brauchen?

☐1 Immer	☐4 Manchmal
☐2 Meistens	☐5 Selten
☐3 Oft	☐6 Nie

9. Wie oft ist jemand (Partner, Angehörige, Freunde etc) da, welcher Sie spüren lässt, dass Sie gebraucht werden und den Sie lieben?

☐1 Immer	☐4 Manchmal
☐2 Meistens	☐5 Selten
☐3 Oft	☐6 Nie

10. Wie oft pro Monat nehmen Sie an organisierten Veranstaltungen teil *(z.B. Hobby- und Freizeitgruppen, Vereine, kirchliche oder politische Anlässe)*?

☐1 Neunmal oder öfter	☐4 Zweimal
☐2 Fünf- bis achtmal	☐5 Einmal
☐3 Drei- oder viermal	☐6 Nie

Allgemeiner Gesundheitszustand

1. Wie würden Sie im Grossen und Ganzen Ihren gesundheitlichen Allgemeinzustand beschreiben? Würden Sie sagen, er ist ...

☐1 ... ausgezeichnet
☐2 ... gut
☐3 ... mässig
☐4 ... schlecht

© 2000 Dieser Fragebogen ist urheberrechtlich geschützt.

Selbständigkeit

Hier finden Sie einige Fragen zu verschiedenen Tätigkeiten. Unterscheiden Sie dabei bitte, ob Sie völlig selbständig sind oder ob Sie Schwierigkeiten haben, ein Hilfsmittel oder eine Hilfsperson benötigen

**Geben Sie bitte an, ob Sie in der Lage sind, die folgenden Tätigkeiten auszuüben.**		**Selb-ständig ohne Schwie-rigkeiten**	**Selb-ständig aber mit Schwie-rigkeiten**	**Möglich aber nur mit Hilfs-mittel**	**Möglich aber nur mit Hilfs-person**	**Nein**
1.	Essen (Nahrungsaufnahme)?	☐1	☐2	☐3	☐4	☐5
2.	Vom Bett aufstehen und sich zu einem Stuhl begeben?	☐1	☐2	☐3	☐4	☐5
3.	Aufs WC gehen?	☐1	☐2	☐3	☐4	☐5
4.	Sich anziehen?	☐1	☐2	☐3	☐4	☐5
5.	Baden oder Duschen?	☐1	☐2	☐3	☐4	☐5
6.	Telefonieren?	☐1	☐2	☐3	☐4	☐5
7.	Sich von zu Hause fortbegeben, beispielsweise mit dem Auto oder öffentlichem Verkehrsmittel?	☐1	☐2	☐3	☐4	☐5
8.	Einkaufen?	☐1	☐2	☐3	☐4	☐5
9.	Mahlzeiten zubereiten?	☐1	☐2	☐3	☐4	☐5
10.	Arbeiten im Haushalt verrichten?	☐1	☐2	☐3	☐4	☐5
11.	Handwerkliche Arbeiten in Haus und Garten?	☐1	☐2	☐3	☐4	☐5
12.	Wäsche waschen?	☐1	☐2	☐3	☐4	☐5
13.	Medikamente einnehmen?	☐1	☐2	☐3	☐4	☐5
14.	Mit Geld umgehen?	☐1	☐2	☐3	☐4	☐5
15.	Etwa 500 Meter zu Fuss gehen?	☐1	☐2	☐3	☐4	☐5
16.	Mindestens dreimal wöchentlich zwei Kilometer zu Fuss gehen?	☐1	☐2	☐3	☐4	☐5
17.	Mindestens dreimal wöchentlich so intensiv Sport zu treiben, dass Sie ins Schwitzen oder ausser Atem geraten?	☐1	☐2	☐3		☐5

© 2000 Dieser Fragebogen ist urheberrechtlich geschützt.

Bei den folgenden Tätigkeiten wollen wir wissen, ob Sie aus gesundheitlichen oder körperlichen Gründen regelmässig oder ständig die ***Art und Weise*** *(Frage 18-20) oder die* ***Häufigkeit*** *(Frage 21-23) ihrer Verrichtung geändert haben.*

**Haben Sie in den letzten 12 Monaten aus gesundheitlichen oder körperlichen Gründen die Art und Weise geändert, mit der Sie ...**		**Nein**	**Ja**	**Diese Tätigkeit nicht aus-geführt**
18.	**... einen Kilometer zu Fuss gehen?** *z. B. Sie gehen seit dem letzten Jahr langsamer oder vorsichtiger, haben Ihre Haltung oder Ihren Schritt verändert, verwenden seit dem letzten Jahr neu einen Stock oder andere Gehhilfen oder legen häufiger Ruhepausen ein als vorher.*	☐ 1	☐ 2	☐ 3
19.	**... 10 Treppenstufen steigen?** *z. B. Sie steigen seit dem letzten Jahr langsamer oder vorsichtiger, haben Ihren Schritt oder Ihre Gehweise verändert, legen häufiger Ruhepausen ein oder benutzen in den letzten 12 Monaten häufiger das Geländer.*	☐ 1	☐ 2	☐ 3
20.	**... in ein Auto, in einen Bus oder in einen Zug ein- oder aussteigen?** *z. B. Sie stützen sich in den letzten 12 Monaten vermehrt mit den Händen ab oder ziehen sich mit den Armen hoch, Sie nehmen sich mehr Zeit oder lassen sich neu seit dem letzten Jahr von anderen helfen.*	☐ 1	☐ 2	☐ 3

**Sind Sie aus gesundheitlichen oder körperlichen Gründen in den letzten 12 Monaten weniger oft ...**		**Nein**	**Ja**	**Diese Tätigkeit nicht aus-geführt**
21.	**... einen Kilometer zu Fuss gegangen?** *z. B. Sie gehen seltener zu Fuss als noch vor einem Jahr, nicht mehr jeden Tag, sondern nur noch 3-4 mal pro Woche, beispielsweise weil Sie schneller ermüden.*	☐ 1	☐ 2	☐ 3
22.	**... 10 Treppenstufen gestiegen?** *z. B. Sie steigen seltener Treppenstufen als noch vor einem Jahr, wenn möglich, nehmen Sie häufiger den Fahrstuhl, vielleicht weil Sie Gelenkbeschwerden haben.*	☐ 1	☐ 2	☐ 3
23.	**... in ein Auto, in einen Bus oder in einen Zug eingestiegen?** *z. B. Sie benutzen das Auto oder den Bus seltener als noch vor einem Jahr, weil Sie möglicherweise Mühe beim Aufstehen aus den Sitzen haben.*	☐ 1	☐ 2	☐ 3

© 2000 Dieser Fragebogen ist urheberrechtlich geschützt.

Rauchen

Frage 1 und 2 richten sich an ALLE Befragten:

1. Welche Aussage trifft für Sie am ehesten zu?
   Kreuzen Sie das für Sie Zutreffende an.
   - ☐1 Ich bin zur Zeit Zigarettenraucher
   - ☐2 Ich habe früher Zigaretten geraucht
   - ☐3 Ich habe nie Zigaretten geraucht

2. Konsumieren Sie Tabak in einer anderen Form *(z.B. Zigarre oder Pfeife)*?
   - ☐1 Nein
   - ☐2 Ja

Waren Sie früher Zigarettenraucher, ***dann fahren Sie auf Seite 21 (Frage 8) fort.***
Wenn Sie nie Zigaretten geraucht haben, ***dann fahren Sie auf Seite 21 (Alkohol) fort.***

Fragen 3 bis 7 richten sich nur an JETZIGE Zigarettenraucher.

3. Wie viele Zigaretten rauchen Sie normalerweise pro Tag?
   - ☐1 1 pro Tag oder weniger
   - ☐2 Mehr als 1 pro Tag ☐☐☐ Zigaretten pro Tag

4. Wie alt waren Sie, als Sie anfingen, mehr oder weniger regelmässig Zigaretten zu rauchen?
   ☐☐ Jahre alt

5. Haben Sie ernsthaft daran gedacht, im Laufe der nächsten 6 Monate mit dem Rauchen aufzuhören?
   - ☐1 Nein → *Fahren Sie bei Frage 7 auf dieser Seite fort*
   - ☐2 Ja

6. Haben Sie vor, in den nächsten 30 Tagen mit dem Rauchen aufzuhören?
   - ☐1 Nein
   - ☐2 Ja

7. Welche der folgenden Gründe halten Sie davon ab, mit dem Rauchen aufzuhören?
   Hier sind mehrere Antworten möglich. ***Kreuzen Sie bitte alle zutreffenden Antworten an und fahren anschliessend auf Seite 21 (Alkohol) fort***

   a. ☐1 Ich will nicht aufhören
   b. ☐1 Ich glaube, ich kann das nicht
   c. ☐1 Stress
   d. ☐1 Gewichtszunahme
   e. ☐1 Andere Gründe

© 2000 Dieser Fragebogen ist urheberrechtlich geschützt.

Frage 8 und 9 richten sich nur an EHEMALIGE Zigarettenraucher:

8. Wie alt waren Sie, als Sie <u>anfingen</u>, mehr oder weniger regelmässig Zigaretten zu rauchen?

   ☐☐ Jahre alt

9. Wie alt waren Sie, als Sie <u>aufhörten</u>, mehr oder weniger regelmässig Zigaretten zu rauchen?

   ☐☐ Jahre alt

Alkohol

Im Folgenden fragen wir Sie nach Ihrem Alkoholkonsum.
Als ein alkoholisches Getränk zählt eines der folgenden Beispiele:

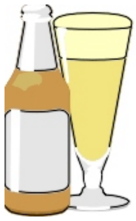

1 Dose Bier
1 Flasche Bier
0,3 l (3 dl)

oder

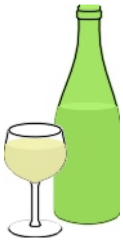

1 Glas Wein
1 Glas Sekt
0,1 l (1 dl)

oder

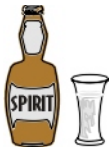

1 Gläschen
Schnaps, Magen-bitter usw. 2 cl

oder

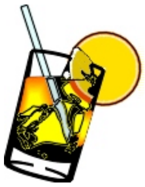

1 Gläschen
Likör oder
Mischgetränk

1. Wie oft trinken Sie alkoholische Getränke?
   - ☐1 Nie → *Fahren Sie auf Seite 23 (Körperliche Aktivität) fort*
   - ☐2 Einmal im Monat oder seltener
   - ☐3 Zwei- bis viermal im Monat
   - ☐4 Zwei- bis dreimal in der Woche
   - ☐5 Viermal in der Woche oder öfter

Bitte fahren Sie nun fort mit den folgenden Fragen 2 bis 10, auch wenn Sie nur selten Alkohol konsumieren

2. Wie viele alkoholische Getränke (Zählung siehe oben) konsumieren Sie etwa an einem Tag, an dem Sie Alkohol trinken?
   - ☐1 1 oder 2
   - ☐2 3 oder 4
   - ☐3 5 oder 6
   - ☐4 7 bis 9
   - ☐5 10 oder mehr

3. Wie oft kommt es vor, dass Sie bei einer Gelegenheit **vier** oder mehr alkoholische Getränke konsumieren?
   - ☐1 Nie
   - ☐2 Weniger als einmal im Monat
   - ☐3 Einmal im Monat
   - ☐4 Jede Woche einmal
   - ☐5 Täglich oder fast täglich

© 2000 Dieser Fragebogen ist urheberrechtlich geschützt.

4. Ist es in den letzten 12 Monaten vorgekommen, dass Sie nicht mit dem Trinken aufhören konnten, wenn Sie einmal angefangen hatten?

- ☐ 1 Nie
- ☐ 2 Weniger als einmal im Monat
- ☐ 3 Einmal im Monat
- ☐ 4 Jede Woche einmal
- ☐ 5 Täglich oder fast täglich

5. Ist es in den letzten 12 Monaten vorgekommen, dass Sie Ihre Arbeiten nicht wie geplant erledigen konnten, weil Sie getrunken haben?

- ☐ 1 Nie
- ☐ 2 Weniger als einmal im Monat
- ☐ 3 Einmal im Monat
- ☐ 4 Jede Woche einmal
- ☐ 5 Täglich oder fast täglich

6. Ist es in den letzten 12 Monaten vorgekommen, dass Sie einen morgendlichen Kater hatten?

- ☐ 1 Nie
- ☐ 2 Weniger als einmal im Monat
- ☐ 3 Einmal im Monat
- ☐ 4 Jede Woche einmal
- ☐ 5 Täglich oder fast täglich

7. Ist es in den letzten 12 Monaten vorgekommen, dass Sie Schuld- oder Reuegefühle hatten, nachdem Sie getrunken haben?

- ☐ 1 Nie
- ☐ 2 Weniger als einmal im Monat
- ☐ 3 Einmal im Monat
- ☐ 4 Jede Woche einmal
- ☐ 5 Täglich oder fast täglich

8. Ist es in den letzten 12 Monaten vorgekommen, dass Sie sich am nächsten Morgen nicht mehr erinnern konnten, was am Abend vorher geschehen war, weil Sie getrunken haben?

- ☐ 1 Nie
- ☐ 2 Weniger als einmal im Monat
- ☐ 3 Einmal im Monat
- ☐ 4 Jede Woche einmal
- ☐ 5 Täglich oder fast täglich

9. Haben Sie sich im Zusammenhang mit Alkoholgenuss schon einmal verletzt?

- ☐ 1 Nein
- ☐ 2 Ja, aber nicht in den letzten 12 Monaten
- ☐ 3 Ja, während der letzten 12 Monate

10 Hat sich ein Verwandter, ein Freund, ein Arzt oder eine andere Bezugsperson besorgt über Ihr Trinken gezeigt oder Ihnen geraten, das Trinken einzuschränken?

- ☐ 1 Nein
- ☐ 2 Ja, aber nicht in den letzten 12 Monaten
- ☐ 3 Ja, während der letzten 12 Monate

© 2000 Dieser Fragebogen ist urheberrechtlich geschützt.

Körperliche Aktivität

Bei den folgenden Fragen geht es um Ihre körperliche Betätigung während der vergangenen sieben Tage. Wenn die letzte Woche nicht Ihrer normalen Lebensweise entsprach, antworten Sie für eine für Sie typische Woche. Beantworten Sie alle einzelnen Fragen so genau wie möglich. Denken Sie bitte daran, beide Teile der Frage zu beantworten.

1a. An wie vielen Tagen der letzten Woche haben Sie sich **ausserhalb Ihres Bettes sitzend beschäftigt** mit Tätigkeiten *wie z.B. Lesen, Fernsehen, Telefonieren oder Handarbeiten*?

- ☐1 Nie → *Fahren Sie bei Frage 2a auf dieser Seite fort*
- ☐2 1-2 Tage pro Woche
- ☐3 3-4 Tage pro Woche
- ☐4 5-7 Tage pro Woche

1b. Wie viele Stunden etwa haben Sie sich an diesen Tagen sitzend beschäftigt?

- ☐1 Weniger als 1 Stunde pro Tag
- ☐2 1-2 Stunden pro Tag
- ☐3 2-4 Stunden pro Tag
- ☐4 Mehr als 4 Stunden pro Tag

2a. An wie vielen Tagen der letzten Woche waren Sie aus irgendeinem Grund **zu Fuss ausserhalb Ihrer Wohnung unterwegs** *wie z.B. zum Spazieren, Einkaufen, für Besuche oder andere Tätigkeiten*?

- ☐1 Nie → *Fahren Sie bei Frage 3a auf dieser Seite fort*
- ☐2 1-2 Tage pro Woche
- ☐3 3-4 Tage pro Woche
- ☐4 5-7 Tage pro Woche

2b. Wie viele Stunden etwa gingen Sie an diesen Tagen zu Fuss?

- ☐1 Weniger als 1 Stunde pro Tag
- ☐2 1-2 Stunden pro Tag
- ☐3 2-4 Stunden pro Tag
- ☐4 Mehr als 4 Stunden pro Tag

3a. An wie vielen Tagen der letzten Woche haben Sie **leichten Sport oder Freizeitaktivitäten** *wie z.B. leichte Lockerungsübungen, Kegeln, Angeln oder ähnliches* getrieben?

- ☐1 Nie → *Fahren Sie auf Seite 24 (Frage 4a) fort*
- ☐2 1-2 Tage pro Woche
- ☐3 3-4 Tage pro Woche
- ☐4 5-7 Tage pro Woche

3b. Wie viele Stunden etwa haben Sie an diesen Tagen solche leichten Sportarten oder Freizeitaktivitäten getrieben?

- ☐1 Weniger als 1 Stunde pro Tag
- ☐2 1-2 Stunden pro Tag
- ☐3 2-4 Stunden pro Tag
- ☐4 Mehr als 4 Stunden pro Tag

© 2000 Dieser Fragebogen ist urheberrechtlich geschützt.
© 1991 New England Research Institutes, Inc, 9, Galen Street, Watertown, MA 02472 U.S.A.

4a. An wie vielen Tagen der letzten Woche haben Sie **mässig anstrengenden Sport** getrieben *wie z.B. Gymnastik (auch Wassergymnastik), Freizeitschwimmen,Tanzen, Wandern, Radfahren zum Einkaufen oder zum Vergnügen oder ähnliches*?

- ☐1 Nie → *Fahren Sie bei Frage 5a auf dieser Seite fort*
- ☐2 1-2 Tage pro Woche
- ☐3 3-4 Tage pro Woche
- ☐4 5-7 Tage pro Woche

4b. Wie viele Stunden etwa haben Sie an diesen Tagen solche mässig anstrengenden Sportarten getrieben?

- ☐1 Weniger als 1 Stunde pro Tag
- ☐2 1-2 Stunden pro Tag
- ☐3 2-4 Stunden pro Tag
- ☐4 Mehr als 4 Stunden pro Tag

5a. An wie vielen Tagen der letzten Woche haben Sie **stark anstrengenden Sport** getrieben *wie z.B. Joggen, Sportschwimmen, Radfahren (als Sport oder auf Hometrainer), Tennis, Aerobic, Ballsport, Skifahren (Alpin und Langlauf) oder ähnliches*?

- ☐1 Nie → *Fahren Sie bei Frage 6a auf dieser Seite fort*
- ☐2 1-2 Tage pro Woche
- ☐3 3-4 Tage pro Woche
- ☐4 5-7 Tage pro Woche

5b. Wie viele Stunden etwa haben Sie an diesen Tagen solche stark anstrengenden Sportarten getrieben?

- ☐1 Weniger als 1 Stunde pro Tag
- ☐2 1-2 Stunden pro Tag
- ☐3 2-4 Stunden pro Tag
- ☐4 Mehr als 4 Stunden pro Tag

6a. *Eine spezielle Form der körperlichen Aktivität sind Übungen zur Erhöhung der Muskelkraft oder -ausdauer.*
An wie vielen Tagen der letzten Woche haben Sie **spezielles Muskeltraining durchgeführt** *wie z.B. Hanteltraining, Gewichtheben, Liegestütze, Verwendung von Krafttrainingsgeräten oder ähnliches*?

- ☐1 Nie → *Fahren Sie auf Seite 25 (Frage 7) fort*
- ☐2 1-2 Tage pro Woche
- ☐3 3-4 Tage pro Woche
- ☐4 5-7 Tage pro Woche

6b. Wie viele Stunden etwa haben Sie an diesen Tagen spezielles Muskeltraining durchgeführt?

- ☐1 Weniger als 1 Stunde pro Tag
- ☐2 1-2 Stunden pro Tag
- ☐3 2-4 Stunden pro Tag
- ☐4 Mehr als 4 Stunden pro Tag

© 2000 Dieser Fragebogen ist urheberrechtlich geschützt.
© 1991 New England Research Institutes, Inc, 9, Galen Street, Watertown, MA 02472 U.S.A.

7.	**Haben Sie in <u>der letzten Woche</u> Arbeiten <u>im eigenen Haushalt</u> verrichtet wie:**	**Nein**	**Ja**
a.	Leichtere Hausarbeit *wie Staubwischen, Wäsche waschen, Bügeln oder Geschirrspülen*?	☐1	☐2
b.	Schwerere Hausarbeit *wie Staubsaugen, Fussböden reinigen oder Fensterputzen*?	☐1	☐2
c.	Reparaturarbeiten zu Hause *wie Malen, Tapezieren, Installationen*?	☐1	☐2
d.	Schwerere Garten- oder Umgebungsarbeiten *wie Rasenmähen, Umgraben, Holzhacken oder Schneeschaufeln*?	☐1	☐2
e.	Leichtere Gartenarbeit *wie Laubrechen, Beet- oder Grabpflege*?	☐1	☐2
f.	Anstrengende pflegerische Verrichtungen *wie Kinderbetreuung, Pflege des hilfsbedürftigen Partners oder eines anderen Erwachsenen*?	☐1	☐2

8a. **Haben Sie in der letzten Woche <u>ausserhalb Ihres Haushaltes</u> gearbeitet? (ehrenamtlich, unentgeltlich oder gegen Bezahlung)**

☐1 Nein → *Fahren Sie bei Frage 9 auf dieser Seite fort*

☐2 Ja ↓

8b. Wie viele <u>Stunden pro Woche</u> haben Sie gegen Bezahlung und/oder unentgeltlich/ehrenamtlich gearbeitet?

☐☐ Anzahl Stunden pro Woche

8c. Welche der folgenden Beschreibungen trifft am besten auf **das Ausmass an körperlicher Tätigkeit** bei der Arbeit ausserhalb Ihres Haushaltes zu?

☐1 Hauptsächlich sitzende Tätigkeit mit leichten Armbewegungen

☐2 Tätigkeit im Sitzen oder Stehen mit einigen Gängen

☐3 Tätigkeit im Umhergehen mit Handhabung von Materialien, die weniger als 20 kg wiegen

☐4 Tätigkeit im Umhergehen mit schwerer körperlicher Arbeit, bei der oft Materialien, die über 20 kg wiegen, gehandhabt werden

Sie haben auf den Seiten 23 bis 25 Fragen zur körperlichen Aktivität beantwortet.

9. <u>Seit wann</u> sind Sie in der von Ihnen beschriebenen Form aktiv?

☐1 Seit über 6 Monaten

☐2 Seit weniger als 6 Monaten

☐3 Ich will nächsten Monat damit anfangen

☐4 Ich will in den nächsten 6 Monaten damit anfangen

☐5 Ich bin körperlich nicht aktiv und habe diesbezüglich keine Pläne

© 2000 Dieser Fragebogen ist urheberrechtlich geschützt.
© 1991 New England Research Institutes, Inc, 9, Galen Street, Watertown, MA 02472 U.S.A.

10. Welche der folgenden Gründe halten Sie davon ab, sich mehr körperlich zu betätigen?
Hier sind mehrere Antworten möglich. ***Kreuzen Sie bitte alle zutreffenden Antworten an.***

a. ☐1 Ich bin bereits regelmässig körperlich aktiv
b. ☐1 Ich habe niemanden, der mitmacht
c. ☐1 Ich kenne keine Angebote, die für meine Altersgruppe geeignet sind
d. ☐1 Ich habe keine Zeit
e. ☐1 Kosten
f. ☐1 Wetter
g. ☐1 Ich habe eine Krankheit, die meine Belastbarkeit einschränkt
h. ☐1 Ich habe eine körperliche Behinderung
i. ☐1 Ich habe vermehrt Schmerzen, wenn ich mich körperlich betätige
j. ☐1 Andere Gründe

Ernährung

Denken Sie an Ihre Essgewohnheiten während des letzten Monats. Wie oft ungefähr haben Sie von den folgenden Lebensmittelgruppen konsumiert?
Kreuzen Sie eine Antwort für jede Lebensmittelgruppe an.

Wie oft etwa essen Sie von diesen Lebensmitteln?		Nie oder seltener als 1mal pro MONAT	1-3 mal pro MONAT	1-2mal pro WOCHE	3-4mal pro WOCHE	5mal oder häufiger pro WOCHE
1.	Fettreiche, durchzogene Fleischstücke (*z.B. Bruststück vom Rind, Braten oder Schnitzel vom Schweinehals, Kalbsbrustschnitten*)	☐1	☐2	☐3	☐4	☐5
2.	Gebratenes Poulet mit Haut, panierte Schnitzel, Cordon bleu, panierten und frittierten Fisch	☐1	☐2	☐3	☐4	☐5
3.	Hackfleisch, Hackbraten, Hamburger	☐1	☐2	☐3	☐4	☐5
4.	Cervelat, Wienerli, Bratwurst, Schüblig, Fleischkäse	☐1	☐2	☐3	☐4	☐5
5.	Aufschnitt, Leberpastete	☐1	☐2	☐3	☐4	☐5
6.	Speck, Salami, Mortadella, Coppa, Streichwurst	☐1	☐2	☐3	☐4	☐5
7.	Vollfetter Käse/ Schmelzkäse, Fondue, Raclette, Doppelrahmkäse	☐1	☐2	☐3	☐4	☐5
8.	Eier	☐1	☐2	☐3	☐4	☐5
9.	Vollmilch, Kaffeerahm, Rahm, Sauerrahm, Creme fraiche	☐1	☐2	☐3	☐4	☐5
10.	Normale Margarine, Butter, Kräuter-butter, Mayonnaise, Rahmsauce, Béchamelsauce	☐1	☐2	☐3	☐4	☐5

© 2000 Dieser Fragebogen ist urheberrechtlich geschützt.

Wie oft etwa essen Sie von diesen Lebensmitteln?		Nie oder seltener als 1mal pro MONAT	1-3 mal pro MONAT	1-2mal pro WOCHE	3-4mal pro WOCHE	5mal oder häufiger pro WOCHE
11.	Salatsaucen auf Rahm- oder Mayonnaisebasis (*z.B. French Dressing*)	☐1	☐2	☐3	☐4	☐5
12.	Pommes frites, Kroketten, Bratkartoffeln, Rösti	☐1	☐2	☐3	☐4	☐5
13.	Pommes Chips, Knabbergebäck, Nüsse, Erdnüsse, Butterstängeli	☐1	☐2	☐3	☐4	☐5
14.	Schokolade, Pralinés, Guetzli	☐1	☐2	☐3	☐4	☐5
15.	Rahmglace, Patisserie, Kuchen, Blätterteiggebäck, Berliner, Schenkeli	☐1	☐2	☐3	☐4	☐5

Noch einmal: Denken Sie an Ihre Essgewohnheiten während des letzten Monats. Wie oft ungefähr haben Sie von den folgenden Lebensmittelgruppen konsumiert?
Kreuzen Sie eine Antwort für jede Lebensmittelgruppe an.

Wie oft etwa essen Sie von diesen Lebensmitteln?		Nie oder Seltener als 1mal pro WOCHE	1mal pro WOCHE	2-3 mal pro WOCHE	4-6 mal pro WOCHE	Jeden Tag
16.	Frische Früchte, Dörrfrüchte, Kompott	☐1	☐2	☐3	☐4	☐5
17.	Naturreine Fruchtsäfte (*z.B. Orangen- oder Apfelsaft*)	☐1	☐2	☐3	☐4	☐5
18.	Blattsalate	☐1	☐2	☐3	☐4	☐5
19.	Rohe oder gekochte Gemüse	☐1	☐2	☐3	☐4	☐5
20.	Hülsenfrüchte (*z.B. Linsen, weisse oder rote Bohnen, Kichererbsen*)	☐1	☐2	☐3	☐4	☐5
21.	Kartoffeln	☐1	☐2	☐3	☐4	☐5
22.	Vollreis, Vollkornteigwaren	☐1	☐2	☐3	☐4	☐5
23.	Getreideflocken, Kleie	☐1	☐2	☐3	☐4	☐5
24.	Dunkles Brot (*z.B. Vollkorn- oder Roggenbrot*)	☐1	☐2	☐3	☐4	☐5

© 2000 Dieser Fragebogen ist urheberrechtlich geschützt.

25. Seit wann unternehmen Sie etwas, um den Konsum fetthaltiger Nahrung einzuschränken?

- ☐1 Seit über 6 Monaten
- ☐2 Seit weniger als 6 Monaten
- ☐3 Ich will nächsten Monat damit anfangen
- ☐4 Ich will in den nächsten 6 Monaten damit anfangen
- ☐5 Ich habe diesbezüglich keine Pläne

26. Mit welchen Schwierigkeiten haben Sie zu kämpfen, um weniger fett zu essen?
Hier sind mehrere Antworten möglich. ***Kreuzen Sie bitte alle zutreffenden Antworten an.***

- a. ☐1 Ich habe meinen Fettkonsum bereits eingeschränkt
- b. ☐1 Ich glaube nicht, dass es wichtig ist, weniger fett zu essen
- c. ☐1 Ich mag den Geschmack von stark fetthaltigen Speisen
- d. ☐1 Meine Freunde/Familienmitglieder möchten den Konsum von stark fetthaltigen Speisen nicht einschränken
- e. ☐1 Ich finde es kompliziert, fettarme Kost einzukaufen und zu kochen
- f. ☐1 Fettreiche Speisen sind leicht zu haben
- g. ☐1 Fettarme Speisen sind teurer
- h. ☐1 Andere Gründe

27. Seit wann unternehmen Sie etwas, um Obst, Gemüse und Ballaststoffe in Ihren Speisezettel aufzunehmen?

- ☐1 Seit über 6 Monaten
- ☐2 Seit weniger als 6 Monaten
- ☐3 Ich will nächsten Monat damit anfangen
- ☐4 Ich will in den nächsten 6 Monaten damit anfangen
- ☐5 Ich habe diesbezüglich keine Pläne

28. Was hält Sie davon ab, mehr Obst, Gemüse und Ballaststoffe zu essen?
Hier sind mehrere Antworten möglich. ***Kreuzen Sie bitte alle zutreffenden Antworten an.***

- a. ☐1 Ich esse bereits viel Obst, Gemüse und Ballaststoffe
- b. ☐1 Ich glaube nicht, dass es wichtig ist, diese Nahrungsmittel meiner Kost hinzuzufügen
- c. ☐1 Ich mag den Geschmack nicht
- d. ☐1 Meine Freunde/Familienmitglieder möchten nicht, dass ich mehr davon verwende
- e. ☐1 Ich finde es kompliziert, Obst, Gemüse und ballaststoffreiche Nahrungsmittel einzukaufen und zuzubereiten
- f. ☐1 Gute Produkte sind nicht leicht zu haben
- g. ☐1 Gute Produkte sind teuer
- h. ☐1 Andere Gründe

© 2000 Dieser Fragebogen ist urheberrechtlich geschützt.

Vorbeugung gegen Verletzungen

Sicherheit beim Autofahren

1. Wie oft verwenden Sie die Sicherheitsgurte, wenn Sie ein Auto fahren oder mitfahren?
   - ☐1 Immer
   - ☐2 Fast immer
   - ☐3 Manchmal
   - ☐4 Selten
   - ☐5 Nie

2. Wie oft fahren Sie mit einem Auto oder Motorrad, wenn der Fahrer, Sie eingeschlossen, Alkohol getrunken hat?
   - ☐1 Nie
   - ☐2 Weniger als einmal im Jahr
   - ☐3 Weniger als einmal im Monat, aber mindestens einmal im Jahr
   - ☐4 Weniger als einmal in der Woche, aber mindestens einmal im Monat
   - ☐5 Ein- oder zweimal die Woche
   - ☐6 Dreimal die Woche oder öfter
   - ☐7 Täglich

Gleichgewicht

3. Schränken Sie gewisse Tätigkeiten ein, weil Sie Angst haben, hinzufallen?
   - ☐1 Nein
   - ☐2 Ja

4. Sind Sie in den letzten Jahren jemals hingefallen und haben nicht mehr aufstehen können?
   - ☐1 Nein
   - ☐2 Ja

5a. Sind Sie im Laufe der letzten 12 Monate jemals hingefallen?
   - ☐1 Nein → *Fahren Sie auf Seite 30 (Gesundheitsvorsorge) fort*
   - ☐2 Ja ↓

5b. Wie oft?
   - ☐1 Einmal
   - ☐2 Mehr als einmal

5c. Ereignete/ereigneten sich der Sturz/die Stürze...
   - ☐1 Nur innerhalb der Wohnung / zu Hause
   - ☐2 Nur ausserhalb der Wohnung
   - ☐3 Innerhalb und ausserhalb der Wohnung

© 2000 Dieser Fragebogen ist urheberrechtlich geschützt.

Gesundheitsvorsorge

Wir möchten gerne wissen, ob die folgenden Vorsorgemassnahmen bei Ihnen durchgeführt wurden.

**Sind folgende Massnahmen bei Ihnen vorgenommen worden?** **Bitte beantworten Sie jede Frage mit Nein oder Ja**		**Nein**	**Ja**
1.	Blutdruckmessung innerhalb der vergangenen 12 Monate?	☐1	☐2
2.	Augenkontrolle bei einem Augenarzt / einer Augenärztin oder einem Optiker / einer Optikerin innerhalb der vergangenen 12 Monate?	☐1	☐2
3.	Gehörkontrolle / einfache Hörprüfung bei einem Arzt / einer Ärztin oder bei einer Fachstelle für Hörberatung innerhalb der vergangenen 12 Monate?	☐1	☐2
4.	Zahnkontrolle innerhalb der vergangenen 12 Monate?	☐1	☐2
5.	Cholesterinmessung innerhalb der letzten 5 Jahre?	☐1	☐2
6.	Bestimmung des Blutzuckers innerhalb der vergangenen 3 Jahre?	☐1	☐2
7.	Untersuchung auf Blut im Stuhl innerhalb der vergangenen 12 Monate?	☐1	☐2
8.	Impfung gegen Grippe innerhalb der vergangenen 12 Monate?	☐1	☐2
9.	Impfung gegen Lungenentzündung (Pneumovax) in den vergangenen 10 Jahren?	☐1	☐2
Nur für Frauen:			
10.	Mammographie (Röntgen der Brust) innerhalb der letzten 2 Jahre?	☐1	☐2
11.	Vaginal-Abstrich zur Krebsvorsorge innerhalb der letzten 3 Jahre?	☐1	☐2

Bei der folgenden Frage möchten wir wissen, weshalb einige der obengenannten Vorsorgemassnahmen bei Ihnen nicht durchgeführt worden sind.

12. Was hat Sie gegebenenfalls von den oben aufgelisteten Vorsorgemassnahmen abgehalten?
Hier sind mehrere Antworten möglich. ***Kreuzen Sie bitte alle zutreffenden Antworten an.***

a. ☐1 Diese Frage trifft auf mich nicht zu, bei mir wurden bereits alle Vorsorgemassnahmen durchgeführt
b. ☐1 Bei mir nicht nötig, ich habe keine gesundheitlichen Probleme
c. ☐1 Ich habe bisher nie daran gedacht
d. ☐1 Finanzielle Gründe/Kosten, Versicherung
e. ☐1 Aus meiner Sicht sind diese Massnahmen nicht wichtig
f. ☐1 Wurde vom Arzt/von der Ärztin oder anderen Fachpersonen nie empfohlen
g. ☐1 Ich habe keine Zeit dazu
h. ☐1 Andere Gründe

© 2000 Dieser Fragebogen ist urheberrechtlich geschützt.

Beruf und Ruhestand

1a. Welche berufliche Tätigkeit haben Sie in Ihrem Leben vorwiegend ausgeübt?

1b. Welche Beschreibung trifft für diese berufliche Tätigkeit zu?
Kreuzen Sie bitte nur eine Antwort an.

- ☐1 Angestellt mit Leitungsfunktion
- ☐2 Angestellt ohne Leitungsfunktion
- ☐3 Selbständig (Landwirtschaft)
- ☐4 Selbständig (Industrie, Gewerbe, Dienstleistung)
- ☐5 Hausfrau

2. Sind Sie zur Zeit im Ruhestand?
- ☐1 Nein → *Fahren Sie auf Seite 32 (Persönliche Angaben) fort*
- ☐2 Ja ↓

3. Sind Sie aufgrund eines körperlichen oder gesundheitlichen Problems vorzeitig in Ruhestand gegangen?
- ☐1 Nein
- ☐2 Ja

4. Arbeiten Sie zur Zeit gegen Bezahlung?
- ☐1 Nein
- ☐2 Ja, Teilzeit
- ☐3 Ja, Vollzeit

5. Leisten Sie zur Zeit ehrenamtliche Arbeit?
- ☐1 Nein
- ☐2 Ja, Teilzeit
- ☐3 Ja, Vollzeit

© 2000 Dieser Fragebogen ist urheberrechtlich geschützt.

Persönliche Angaben

1. Wo wurden Sie geboren?
   - ☐1 In der Schweiz:

     Welcher Ort? ☐☐☐☐☐☐☐☐☐☐☐☐☐☐☐☐☐☐ Welcher Kanton? ☐☐

   - ☐2 In einem anderen Land:

     In welchem Land? ☐☐☐☐☐☐☐☐☐☐☐☐☐☐☐☐☐☐

2. Welches ist Ihre Muttersprache?
   - ☐1 Deutsch
   - ☐2 Französisch
   - ☐3 Italienisch
   - ☐4 Andere Sprache ____________________

3. Welches ist Ihre Wohnsituation? Leben Sie ...
   - ☐1 Alleine
   - ☐2 Mit festem Partner (Ehepartner, Lebenspartner) in einem Haushalt
   - ☐3 Mit einer anderen Person/anderen Personen *(z.B. Familienangehörige)* in einem Haushalt

4. Welches ist Ihre gegenwärtige Wohnform? Leben Sie gegenwärtig ...
   - ☐1 In einer Wohnung / in einem Haus
   - ☐2 In einer betreuten Wohnform *(z.B. Alterssiedlung)*
   - ☐3 In einem Alters-/Pflegeheim

5. Welches ist die höchste Ausbildungsstufe, die Sie abgeschlossen haben?
   - ☐1 Universität, Hochschule
   - ☐2 Höhere Berufsausbildung *(z.B. Meisterdiplom, Fachhochschule, Seminar)*
   - ☐3 Berufslehre, Berufsschule
   - ☐4 Ausbildung nach der Schule, aber ohne Abschluss
   - ☐5 Keine Ausbildung nach der Schule

© 2000 Dieser Fragebogen ist urheberrechtlich geschützt.

Ihre Meinung zum Fragebogen

Diese abschliessende Beurteilung ermittelt Ihre Erfahrungen mit diesem ausgefüllten Fragebogen. Ihre ehrlichen Einschätzungen helfen uns, diesen Fragebogen für den späteren Einsatz bei anderen Personen zu verbessern.

1. Wie schwer war es für Sie, die Fragen und Anweisungen in diesem Fragebogen zu verstehen?
   - ☐1 Sehr schwer
   - ☐2 Schwer
   - ☐3 Einfach
   - ☐4 Sehr einfach

Wenn *Sehr schwer* oder *Schwer*: Welche Bereiche, Fragen oder Anweisungen waren für Sie schwierig zu verstehen?

____

2. Wie schwer war es für Sie, den Fragebogen auszufüllen?
   - ☐1 Sehr schwer
   - ☐2 Schwer
   - ☐3 Einfach
   - ☐4 Sehr einfach

Wenn *Sehr schwer* oder *Schwer*: Welche Bereiche oder Fragen waren für Sie schwierig zu beantworten?

____

3. Wie viel Hilfe haben Sie benötigt, um diesen Fragebogen auszufüllen?
   - ☐1 Eine andere Person hat den Fragebogen für mich ausgefüllt.
   - ☐2 Ich habe den Fragebogen mit Hilfe einer anderen Person ausgefüllt.
   - ☐3 Ich habe den Fragebogen ohne Hilfe ausgefüllt, hätte aber gerne Hilfe gehabt.
   - ☐4 Ich habe den Fragebogen selbständig ausgefüllt und hätte dafür auch keine Hilfe benötigt.

4. Wie würden Sie die Länge des Fragebogens beurteilen?
   - ☐1 Zu lang
   - ☐2 Gerade richtig
   - ☐3 Zu kurz

5. Wie viel Zeit haben Sie benötigt, um den Fragebogen auszufüllen?

   ☐☐☐ Minuten

© 2000 Dieser Fragebogen ist urheberrechtlich geschützt.

6. Haben Ihnen einzelne Bereiche oder Fragen in dem Bogen <u>missfallen</u>?

☐ 1 Nein
☐ 2 Ja

Wenn *Ja*: Welche Bereiche oder Fragen haben Ihnen missfallen?

---

7. Haben Sie spezielle Themenbereiche in diesem Fragebogen <u>vermisst</u>, die Ihrer Meinung nach über diesen Fragebogen abgedeckt sein sollten?

☐ 1 Nein
☐ 2 Ja

Wenn *Ja*: Um welche Themenbereiche handelt es sich hierbei?

Nehmen Sie sich bitte noch etwas Zeit, um zurück zu blättern und sich zu vergewissern, dass Sie keine Seiten oder Fragen ausgelassen haben.

Datum der Beantwortung des Fragebogens: Tag ☐☐ Monat ☐☐ Jahr ☐☐☐☐

Vielen Dank für das Ausfüllen dieses Fragebogens!

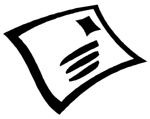

Wir bitten Sie, diesen Fragebogen innert 20 Tagen mit beiliegendem Rückantwortcouvert zurück zu senden.

Version 2.5: Dieser Fragebogen wurde gestützt auf ein von den Regents der University of California, Los Angeles, erteiltes Nutzungsrecht von der Kerngruppe Altersforschung (Spitalverband Bern, Forschungsfonds Geriatrie am Spital Bern-Ziegler, Morillonstrasse 75, CH-3001 Bern) in Zusammenarbeit mit anderen Organisationen entwickelt. Der Fragebogen ist urheberrechtlich geschützt und darf weder als Ganzes noch teilweise ohne vorgängige schriftliche Einwilligung der Urheberrechtsberechtigten verwendet werden.
Version 2.5: © 2000 University of California, Los Angeles
Abschnitt "Körperliche Aktivität": © 1991 New England Research Institutes, Inc, 9 Galen Street, Watertown, MA 02472 U.S.A.

© 2000 Dieser Fragebogen ist urheberrechtlich geschützt.
