# Supplementary material for: Effect of Health Risk Assessment and Counselling on Health Behaviour and Survival in Older People: A Pragmatic Randomised Trial
Source: PLoS Med. 2015 Oct 19;12(10):e1001889. doi: 10.1371/journal.pmed.1001889 (PMC4610679; doi:10.1371/journal.pmed.1001889)
Supplement: S5 Text — (PDF) [file pmed.1001889.s017.pdf]

Supporting Information S5 Text

PRO-AGE Intervention Material

for: A Collaborative Care Model of Health Risk Assessment and Counselling in Older Persons: A Randomised Clinical Trial

PRO-AGE: Intervention Manual

(PRevention in Older persons- Assessment in Generalists' practices)

GUIDANCE NOTES FOR GENERAL PRACTITIONERS PARTICIPATING IN THE INTERVENTION

Note: This is a confidential document for use in the practices participating in the intervention.

C.G. Swift, D. Harari, S. Iliffe, N. Lübke, T. Münzer, A.E. Stuck

Acknowledgement: This project is supported by the European Union (CTR_QLK6-CT-1999-02205). The authors would like to thank J.C. Beck, MD for comments to this manual.

TABLE OF CONTENTS

1. THE HRA-O BASED APPROACH 4

A. THE HRA-O QUESTIONNAIRE (Appendix i) 4

B. THE OLDER PERSONS HEALTH PROFILE REPORT (Appendix ii) 5

C. THE PROVIDER SUMMARY REPORT (Appendix iii) 6

2. REINFORCEMENT AND INTERVENTION BY THE GENERAL PRACTICE 7

A. PRINCIPLES 7

B. ROLE OF THE GENERAL PRACTITIONER 8

C. ROLE OF THE HEALTH EDUCATOR 8

3. EVIDENCE-BASED GUIDELINES: PREVENTIVE HEALTH IN OLDER PEOPLE 9

A. CARDIOVASCULAR RISK PREVENTION 10
Hypertension 10
Hyperlipidaemia 11
Diabetes mellitus 12

B. IMMUNISATIONS 14
Influenza immunisation 14
Pneumonia vaccination 15

C. CANCER SCREENING 16
Colon cancer 16
Breast cancer 17
Cervical cancer 18
Prostate cancer 18

D. HEALTH MAINTENANCE 19
Dental Care

Eyesight testing 19
Hearing assessment 20
Exercise 21
Nutrition 22
Physical function and independence 23

E. SPECIFIC MEDICAL HEALTH ISSUES 24
Pain 24
Medication use 25
Injury prevention and falls 26
Urinary incontinence 27

F. PSYCHOSOCIAL HEALTH AND BEHAVIOUR 29
Screening for depression and dementia 29
Smoking 30
Alcohol use 31

G. APPENDIX

(i) The HRA-O Questionnaire

(ii) The Health Profile Report

(iii) The Provider Summary Report

(iv) Regional information for intervention

(v) Guidelines for Management of Hypertension: Report of the Third Working Party of the British Hypertension Society, 1999 (J Human Hypertension 1999;13:569-592)

(vi) Joint British Societies Coronary Risk Prediction Chart

(vii) Scottish Intercollegiate Guidelines Network (SIGN) Lipids and the primary prevention of coronary heart disease 1999

(viii) Visual Function Qusetionnaire 25

(ix) Mini-mental Status Examination

(x) 15-item Geriatric Depression Scale

1. The HRA-O based approach

A. THE HRA-O QUESTIONNAIRE (Appendix i)

The Health Risk Appraisal for Older Persons (HRA-O) is a computer-based approach based on a self-administered questionnaire. A software program generates a personal report to the older person (the Older Persons Health Profile Report) and a summary report for use by the primary care physician and the health educator.

The HRA-O questionnaire covers domains that identify potential health and disability risk factors in older persons in order to pursue the intervention aims of reducing risk of functional decline and improving quality of life. The chosen domains share the following characteristics:

1. Identify factors with potential impact on physical functionning, quality of life and psychosocial functionning

2. Identify factors that are potentially modifiable with a view to reducing health and disability risks
3. Use generalisable methods of assessment that are feasible with respect to costs, and acceptability by the target population
4. Use standardised measurement instruments that provide quality information in terms of validity, reliability, and sensitivity

Based on these criteria, the following domains and instruments were selected:

**Domain**	**Description**
Medical History	Self-reported chronic conditions
Your Health Measurements	Self-reported height, weight, blood pressure, and cholesterol
Medication	Self-reported medication use, inappropriate medication use
Signs and Symptoms	Monitoring in Elderly of Drug-related Symptoms Questionnaire
Pain	Multidimensional Pain Assessment Instrument
Oral Health	Geriatric Oral Health Index
Eyesight	Visual Functioning Questionnaire
Hearing	Hearing Handicap Inventory for the Elderly
Psychosocial Health and Well-Being	5-item Mental Health Inventory Screening Test, Memory Self Report
Social Network	Lubben Social Network Scale
Self Perceptions About Health	Self-perceived general health status
Functioning	Basic, instrumental and advanced Activities of Daily Living
Tobacco Use	Partners in Prevention Tobacco Use Questionnaire
Alcohol Use	The WHO Alcohol Use Disorders Identification Test
Physical Activity	Physical Activity Scale for the Elderly

Nutrition	Cholesterol Reduction in Seniors Program Fat and Plant Food Screening Questionnaire
Injury Prevention	Study of Osteoporotic Fractures Research Group Survey
Preventive Care	Self-reported use of preventive care

Older persons allocated to the intervention will receive this questionnaire at the project start (and if the project is successful, in yearly intervals thereafter) by mail. Participants will be asked to complete the questionnaire and return it to their general practice. The PRO-AGE project team will then support the general practice by entering the questionnaire into a computer database and by producing the Older Persons Health Profile Report and the Physician Summary Report.

B. THE OLDER PERSONS HEALTH PROFILE REPORT (Appendix ii)

The computer-based programme generates a personal health profile report based on a composite analysis of the questionnaire data. It provides feedback to the patient in the form of recommendations supported by an explanation of benefits.

<u>Categories of Recommendation</u>

Direct information and instruction on health matters

- Advice on health related behaviours which the patients themselves may adopt to improve their health status and reduce risk (e.g. diet, smoking, exercise)
- Information on how to access wider sources of support and information (e.g. national organisations such as the National Osteoporosis Society)
- Prompts for the patients to seek advice from their General Practitioner or Practice Nurse where relevant

The feedback is provided under the following headings:

**Section**	**Information about**
Preventive Care	Personalised preventive services checklist
Medication Use	Tips for tracking medication use
Pain	Pain management
Oral health	Importance of oral care
Eyesight	Eyesight and ageing
Hearing	Hearing and ageing
Emotional and Social Health	Mood and Memory
Emotional and Social Health	Social Network
Functioning	Functional status
Special Concerns	Health matters of particular importance
Tobacco Use	Tobacco use and cessation
Alcohol Use	How effects of alcohol change with age

Physical Activity	Personal physical activity score
Nutrition	Ways to improve diet
Injury Prevention	Reducing risk of injury
Working With Your Doctor	How to get the most from their doctor

C. THE PROVIDER SUMMARY REPORT (Appendix iii)

The provider summary report lists the main findings of the HRA-O questionnaire on two pages. All reported relevant symptoms or findings are indicated on this summary report. This report can be used as a basis for further action, such as further diagnostic work-up, management or referral.

It is re-emphasised that all information listed in the summary report is based on self-reported information provided by the older person. Therefore, its reliability needs to be checked before a clinical decision is made.

The primary care physician and the health educator will use the provider summary report as a basis for preventive and health promotion care.

2. REINFORCEMENT AND INTERVENTION BY THE GENERAL PRACTICE

A. PRINCIPLES

The computerised HRA-O system is the basis for the intervention by the general practice. The written report system alone is not however expected to lead to significant health risk behaviour changes among the target population. Personal reinforcement and follow-up by the general practitioner and a health educator or a health educator team is key for a successful programme.

The method of how the HRA-O will be integrated into the primary health care system and regional factors strongly influence how prevention and health promotion are implemented (see Appendix iv for regional information on intervention). The following are some general principles of how the intervention process is organised:

1. The intervention aims to achieve favourable health-related behaviour changes in older people through patient education by using:
- multiple modalities i.e. written report, personal counselling, general practitioner and health educator involvement
- repeated follow-up
- methods for promoting self-efficacy
- a motivational approach (based on the transtheoretical model of stages of change)

2. An overall aim of the study is to achieve a greater involvement by general practices in preventive care and health promotion amongst the older population. The intervention process therefore incorporates physician training by providing a physician training manual, group training sessions, written and personal feed-back to individual physicians, and interaction with academic geriatricians. A further thrust of the intervention is to effect changes in practice organisation in order to facilitate preventive health care delivery (e.g inserting a prompt and reminder system, developing a supportive role by health educator).

3. Each region has developed a regional version of the intervention process, based on the regional resources (see appendix iv). The priorities are to ensure that (i) the full intervention can be offered to the maximum number of participating patients, trained physicians, and health educators; (ii) the project is optimally integrated into the primary care practice system, (iii) the intervention personnel is working with optimal efficiency, (iv) there is a defined and ongoing interaction between project geriatrician and practice teams, between practice teams, and between individual members of practice team, (v) key aspects of the intervention process are documented for scientific and for quality assurance and documentation purposes, and (vi) that this system undergoes ongoing quality improvement.

B. ROLE OF THE GENERAL PRACTITIONER

- To contribute to the implementation of the intervention by reinforcing recommendations received by patients (in their personal report and from the health educator) on both an opportunistic and systematic basis
- To provide advice, diagnostic assessment, appropriate therapeutic interventions and referrals to other agencies or for specialist care where this is indicated (see specific recommendations in Section 4). This may occur in collaboration with the research health educator where appropriate. The following summarises the role of the GP:
    - Endorsement and reinforcement of recommendations provided in the Health Profile Report.
    - Provision of literature relevant to health risk reduction.
    - Contacts to relevant organisations (e.g. voluntary bodies, local facilities)
    - Professional diagnostic and therapeutic advice
    - Interventions reflecting national or international guidelines (e.g. statutory annual 75+ health check)
    - Interventions and advice based on clinical judgement
    - Referral to specialist care
    - Although reinforcement may have been provided by the practice nurse, the ultimate initiative to seek advice will rest with the individual patient. It is hoped, however, that HRA-O may enhance the take up of a range of prevention recommendations (e.g. influenza immunisation).

Whilst section 4 provides guideline recommendations for preventive health and health risk management, it will, of course, be a matter of individual judgement by the GP regarding the appropriate response in each case. Furthermore published guidelines within this area of health care do change over time, and in general the policy adopted by the GP should be to refer and revert to currently accepted standards where these are available. The educational sessions conducted by the study geriatricians will target any areas where recently published information has led to a revision of the guideline recommendations contained in this document.

C. ROLE OF THE HEALTH EDUCATOR

The health educator will optimise health promotion and preventive care by:

- having a system of regular contact with older persons within the practice to discuss personal health promotion and preventive care (based on patients' questionnaire feed-back)
- motivating older persons for health promotion and disease prevention
- referring older persons to the general practitioner or to other health professionals if needed
- taking a comprehensive view of each older individual to assess their combined risks, problems and resources and to to help them prioritise
- contributing to empowerment of older persons, supporting them in problem-coping and problem-solving
- collaborating with the general practitioner and other members of the team.

3. EVIDENCE-BASED GUIDELINES: PREVENTIVE HEALTH IN OLDER PEOPLE

The guidelines are presented in the following format:

Basic principles This summarises the basic rationale and content of the recommendations that are given to older people in the participant report, and to health practitioners in the guideline document.

Recommendations Summary of guildeline recommendations for action by the physician, in response to health risk issues identified by the HRA-O questionnaire.

Regional information Where relevant, this outlines any special aspect(s) to be taken into account in the European region for which the manual is used.

Background evidence Gives relevant background evidence to the guideline recommendations, including one or more citations of key papers and/or selected articles and any brief quantitative information on benefit and risk where this is available.

Where the *grading* of any supporting data has been systematically assessed or is clear this is indicated (scale ***A – D*** in based on established guidelines *BMJ 1998;316:1303-1309* A-evidence from meta-analysis of randomised controlled trials (RCT's), or from at least one RCT; B-evidence from at least one controlled trial without randomisation or quasi-experimental study; C-evidence from descriptive studies; and D-evidence from expert committee reports or opinions).

The *Appendix* includes copies of recommended assessment tools, and main articles cited in the background or regional section of the manual. It may also include copies of brochures or other material relevant for the physician.

A. CARDIOVASCULAR RISK PREVENTION

Hypertension

Basic principles

Control of hypertension has been widely shown to reduce its complications in older hypertensive patients. This should be in accordance with the level of control recommended by national (e.g. British Hypertension Society) or international (e.g. European Hypertension Society) guidelines on hypertension management in elderly people. These guidelines emphasise the importance of treating isolated systolic hypertension. There is evidence however, that current guidelines are not being implemented, and the detection and management of hypertension remains sub-optimal (***B***).
As a rule of thumb, a minimum annual check is appropriate for older individuals with no history of hypertension and a quarterly check for those controlled on medication. For those with poor control, measures to achieve control (including specialist referral where necessary) and more frequent monitoring are appropriate. While the evidence is less robust for those over 80, the weighting is in favour of achieving control where there is no contraindicating comorbidity.

Recommendations

- Screening: annual measurement of blood pressure
- If BP 140-159/90-99 mmHg: observe monthly for a minimum of 4 months (and annually thereafter) if the levels do not rise above 160 mmHg SBP or 100mmHg DBP. Treat at this level in the presence of established diabetes, cardiovascular complications, or target organ damage, or if 10-year coronary heart disease risk is >= 15% (***B***) (see Appendix for Coronary Risk Prediction Chart).
- If sustained SBP >=160 or DBP >=100 mmHg: drug therapy is indicated, with titration to control (***A***)
- Optimal blood pressure treatment targets are systolic blood pressure =<140 mm Hg and diastolic blood pressure =<85 mm Hg (***A***). For patients with diabetes, a lower target of =<140/80 is recommended (***A***).
- In addition, use non-pharmacological measures in all hypertensive and borderline hypertensive individuals. The following are effective in lowering blood pressure: sodium restriction (***A***), reduced fat intake (***A***), increased fruit and vegetable intake (***A***), regular aerobic exercise (***A***), and alcohol restriction (***A***).
- In the absence of contraindications (including diabetes), low dose thiazides are the first line treatment for elderly hypertensive individuals (***A***).
- For primary prevention to reduce cardiovascular risk, 75mg of aspirin is recommended for hypertensive individuals aged 50 years and older who have either target organ damage or diabetes or a 10 year coronary heart disease risk >= 15 (***A***).

Regional information

No specific regional issues.

Background evidence

Hypertension, including isolated systolic hypertension (=>160/<90 mm Hg), is found in more than half of all people aged over 60. Absolute benefit from treatment of diastolic hypertension and isolated systolic hypertension is greater in older people than younger age groups, particularly with respect to cardiovascular complications (including heart failure), and vascular dementia (***A***).
Antihypertensive treatment is beneficial until at least age 80 (***A***), and regular screening of blood pressure should continue until this age. Once treatment is started, it should be

continued after the age of 80. There is no evidence base in current literature on which to recommend firm guidelines on screening for hypertension, or on treating newly diagnosed hypertension in patients aged over 80. Consensus opinion regarding this age group however, is that treatment of hypertension (with the same optimum levels as younger patients) is appropriate in the context of hypertensive complications or target organ damage. Antihypertensive treatment (even of borderline systolic hypertension) should also be considered in hypertensive octagenarians and beyond who are without complications, and who are generally fit with reasonable life expectancy (***D***).
Low dose thiazides (e.g bendrofluazide 2.5mg) are the accepted first line treatment for elderly people, and are well-tolerated (***A***). Beta-blockers are less effective than thiazides as first line treatment, and meta-analysis suggests that they decrease stroke but no other cardiovascular events in this age group (***A***). Long-acting dihydropyridine calcium antagonists (e.g. amlodipine 5mg) are suitable alternatives for elderly patients when thiazides are ineffective, contraindicated, or not tolerated (***A***). While ACE inhibitors may be useful in treating hypertension coexisting with heart failure or left ventricular dysfunction, they should be used with great caution in older hypertensives who have peripheral vascular disease (because of the association with renovascular disease), or established renal impairment.

References

Guidelines for Management of Hypertension: Report of the Third Working Party of the British Hypertension Society, 1999 (J Human Hypertension 1999;13:569-592) *(see appendix (iv))* or www.hyp.ac.uk/bhs.

Hyperlipidaemia

Basic principles

All older adults should have their total cholesterol levels tested every 5 years, in combination with an assessment for other cardiovascular risk factors. In addition to primary prevention, lowering cholesterol also delays progression of atherosclerotic cardiovascular disease (secondary prevention).

Recommendations

- Measurement of total cholesterol every 5 years, at least until age 75 and above 75 in “biologically fit” older individuals
- Use a coronary Risk prediction chart for guidance on management (cholesterol/HDL ratio is optimal for risk calculation) – see *Appendix (v)* for Joint British Societies Coronary Risk Prediction Chart. If the estimated coronary heart disease event risk at 10 years is <30% then apply lifestyle measures, and reassess at 5 years.
- Appropriate lifestyle measures are reducing dietary fat, sugar and salt (***A***) and increasing starchy carbohydrate, fruit and vegetables (***A***), exercise (***B***), weight reduction if obese (***B***), alcohol limitation if excessive (***B***), and smoking cessation (***B***).
- If the 10 year risk of a coronary event is >= 30 % and the serum cholesterol is <5.0mmol/L, reassess after 1 year of lifestyle measures (***C***).
- If the 10 year risk of a coronary event is >= 30 % and the serum cholesterol is >=5.0mmol/L, reassess after 3-6 months of lifestyle measures. If the fasting total serum cholesterol remains elevated >=5.0 mmol/L, statin therapy is indicated (e.g. pravastatin (***A***) or simvastatin (***B***)) in addition to non-pharmacological measures (***A***). Treatment target is total cholesterol <5.0 mmol/L (***B***).

In view of lack of evidence for individuals with hyperlipidaemia aged 75 years and above, current consensus agreement is that existing statin treatment should be continued in these patients, and de novo treatment should be considered on an individual patient basis (***D***). Non-pharmacological measures should however be appropriately applied in all older patients.

Regional information
No specific regional issues

Background evidence
Hypercholesterolemia,and low HDL (=<0.9 mmol/L) are risk factors for cardiovascular events at least until age 75 years.
Statin therapy for primary and secondary prevention significantly reduces major coronary events (by 30%), all-cause mortality, and stroke risk in patients with coronary heart disease (***A***).

References
The recommendations are based on the Scottish Intercollegiate Guidelines Network (SIGN) Lipids and the primary prevention of coronary heart disease 1999 *(see appendix (vi))* or www.show.scot.nhs.uk/sign/clinical.htm).

Diabetes mellitus

Basic principles
The estimated prevalence of unrecognised diabetes in older people (5%), and the frequently asymptomatic or nonspecific clinical presentation in this population provides the basis for consensus guidelines recommending screening every 3 years.

Recommendations

- Consensus opinion recommends screening for diabetes using a fasting blood glucose (FBG) test every 3 years in those over 45 years of age (***D***).
- Annual screening is recommended in older patients with first-degree family history, obesity, low HDL (=<0.9 mmol/L), high fasting triglycerides (>2.8 mmol/L), or coronary heart disease (***D***).
- The FBG level diagnostic of diabetes is >=7.0mmol/L (***A***).
- In treating diabetes, the same glucose targets (both FBG and HbA1c) apply to healthy older people as to younger people in order to lower the risk of complications (***A***). In elderly patients with comorbidities however, the goal of treatment should be to avoid symptoms of hyperglycaemia, and prevent hypoglycaemia (***D***).
- Sulphonylureas should be used cautiously in older people - start with low initial doses and dosage increments should be gradual (***D***). Gliclazide is preferred, as it has been associated with fewer hypoglycaemic episodes than glyburide (***A***).
- The use of premixed insulins instead of mixing insluins minimises dosage errors in older patients (***B***).
- A multidisciplinary team approach to diabetes management, emphasising patient education by nurses and dieticians has been shown to improve blood glucose control in older patients (***B***).

Regional Information
No specific regional information

Background

The recommendations are based on the 1998 clinical practice guidelines for the management of diabetes in Canada (Canadian Med Assoc J 1998;159 (8Suppl):S1-S28

B. IMMUNISATIONS

Influenza immunisation

Basic principles
Each year in Europe, there are thousands of hospital admissions and deaths due to influenza infection, most in people aged over 65. Systematic reviews from the UK, the US and Canada have concluded that vaccinating elderly people against influenza is an effective, safe, and cost effective way of reducing influenza related deaths and illness.

Recommendations
- Influenza vaccination should be given to all those 65 and over (***A***) and to those below that age, with additional risk factors (adults with chronic respiratory or cardiovascular disease, diabetes mellitus, renal impairment, immunosuppression and residents of long-term care facilities).
- Contraindications are known hypersensitivity or anaphylaxis to eggs, and febrile illness, until symptoms disappear.

Regional information
England: The Department of Health has agreeed arrangements for vaccinating those over the age of 65 against influenza next winter. The agreement is for one year in the first instance. There will be no target payments and there will be an item of service payment of £6.45 for each patient vaccinated. (*BMJ* 2000;320:1734 (24 June)

Background evidence
United States Preventive Services Task Force (USPTF) recommends vaccination for all people age >=65 (***B***) and other risk groups (***B***), annually (***B***). The evidence from systematic reviews of research studies across Europe and N.America clearly shows that annual influenza vaccination of elderly people prevents respiratory illness, pneumonia, hospital admission, and death (***A***). Vaccination of elderly people in long-term care reduces the risk of each of these outcomes by about half (***B***). Trials of influenza vaccine in elderly people have also established its safety, reporting mild local side effects (such as soreness) in less than 20% of subjects and no adverse systemic effects.

References

Diguiseppi C. Why everyone over 65 deserves influenza vaccine *BMJ* 1996;313:1162

Gross PA, Hermogenes AW, Sacks HS, Lau J, Levandowski RA. The efficacy of influenza vaccine in elderly people: a meta-analysis and review of the literature. *Ann Intern Med* 1995;123:519-27

NHS Centre for Reviews and Dissemination. Influenza vaccination and older people. Effectiveness Matters 1996:2(1)

Govaert TM, Dinant GJ, Aretz K, Masurel N, Sprenger M, Knottnerus JA. Adverse reactions to influenza vaccine in elderly people: randomised double blind placebo controlled trial. *BMJ* 1993;307:988-90

Pneumonia vaccination

Pneumonia vaccination

Basic principles

The Pneumococcal vaccination has been shown to be effective at providing immunity against pneumococcus and at reducing morbidity and mortality in older people, long-term care residents, and those with chronic disease.

Recommendations

Pneumovax for all patients aged 65 and older,
and for younger patients with comorbidity (adults with alcoholism or cirrhosis, plus same indications as for influenza vaccination)

Revaccinate after 10 years to boost immunity

Regional information

No specific regional issues

Background evidence

A systemic review of research evaluating the clinical effectiveness of vaccination with pneumococcal polysaccharide vaccine showed that it can be expected to reduce the risk of systemic infection due to pneumococcal types included in the vaccine by 83% and systemic infection due to all pneumococci by 73% (***A***). No evidence was found that the vaccine was less efficacious for the elderly, institutionalised people, or those with chronic disease. In a retrospective study from the US, pneumococcal vaccination of elderly people with chronic lung disease was associated (over a period of 2 years) with fewer hospitalisations for pneumonia, fewer deaths, and direct medical care cost savings (***B***).
A newly-published meta-analysis (*Moore AR et al*) concluded that there was no evidence from randomised trials that pneumococcal vaccine is beneficial in older people. However, this review had two potentially serious flaws. Firstly trials assessing 6-12 valent vaccines were included (low valent vaccines are not effective), and secondly older pople were combined with immunocompromised persons in the analysis. While the effectiveness of pneumococcal vaccine remains under scrutiny, our recommendations are based on the soundest evidence presently available.

Fifty percent of people will have mild local adverse effects of pain and erythema; 1% will have fever and myalgia. Anaphylactic reactions are rare, approximately 5 per million doses.

References

Hutchison BG et al. Clinical effectiveness of pneumococcal vaccine. Meta-analysis. Can Fam Physician 1999;45:2381-93
Nichol KL et al. The health and economic benefits associated with pneumococcal vaccination of elderly people with chronic lung disease. Arch Intern Med 1999;159:2437-42
Moore RA et al. Are the pneumococcal polysaccharide vaccine effective? Meta-analysis of the prospective trials. BMC Family Practice 2000;1:1

C. CANCER SCREENING

Colon cancer

Basic principles

Patients are encouraged to discuss the possible benefits of faecal occult blood testing (FOBT) with their doctor on the basis of information that early detection of colon cancer increases the likelihood of cure.

Recommendations

- The decision to offer FOBT will be a matter of judgement and depends on regional rules, but it is appropriate to discuss the advantages of early detection of colon cancer with individuals fit enough potentially to undergo surgery. It may be appropriate to offer this test to such individuals requesting advice, even if they have no relevant symptoms, but the physician would need to be reasonably certain that the necessary gastroenterology and/or surgical follow-up could be provided locally.
- More serious consideration should be given to this and other investigation (including specialist referring) in individuals at high risk (first degree family history, colitis) or in those with relevant symptoms (e.g. weight loss, anaemia)

Regional information

UK: "Neither colonoscopy nor testing for stool occult blood is yet part of routine screening in the United Kingdom, although there are strong pressures to move in the direction of routine occult blood testing. Although many British gerontologists and gastroenterologists support the view that yearly faecal occult blood testing would be beneficial, neither this nor the necessary follow-up investigation is routinely available within the UK health care system. When individuals at low risk of bowel cancer request screening, they should be informed about the limitations and possible risks of the tests. Occult blood testing should only be offered when there are agreed protocols between primary and secondary care that are backed by the necessary resources for further investigation of individuals with a positive tests, Mass screening for normal risk individuals should await the results of ongoing trials, particularly the NHS pilot study of occult blood testing and the MRC/NHS trials of flexible sigmoidoscopy"
(British Society of Gastroenterology, Royal College of Physicians and the Association of Coloproctology of Great Britain and Ireland (Gut 2000;46:746-8).

Background evidence

USPTF recommendations are that all people age >50 should be screened annually with FOBT (***B***). For every 10 people who test positive for faecal occult blood 1 will be found to have a cancer, 3 an adenoma >1 cm, 1 a small adenoma, and 5 a negative examination. Three randomised trials have shown that screening by faecal occult blood testing every two years has the potential to reduce mortality by up to 20% in patients between 50 and 80 years of age.
Although 74% of bowel cancers found in patients testing positive on FOBT are distally located and therefore reachable by sigmoidoscopy, a distal lesion is associated with a greater than 10% chance of having another significant lesion proximally. Total colonoscopy is therefore regarded as the investigation of choice in patients with positive FOBT (***B***). There is insufficient evidence to determine whether the combination of FOBT and sigmoidoscopy produces greater benefits than either test alone.

References

AMWF-Leitlinie:Kolorektales Karzinom Prävention und Früherkennung in der asymptomatischen Bevölkerung – Vorsorge bei Risikogruppen – Endoskopische

Diagnostik und Therapie von Polypen und Karzinomen. Ergebnisse einer interdisziplinären Konsensuskonferenz Januar 1999. www.uni-duesseldorf.de/WWW/AWMF/awmfleit.htm.
Atkin W. Implementing screening for colorectal cancer BMJ 1999;319:1212-1213
Colon cancer screening (USPSTF Recommendation). J Am Geriatr Soc 2000;48:333-5

Breast cancer

Basic principles

While mammography is recommended every 1 to 2 years in women aged 50-69, the benefits of such a screening strategy on breast cancer mortality in women aged 70 and older are unclear.

Recommendations

- A screening mammogram every 2 years between age 50 and 69, where feasible within the National health screening programme.
- An annual clinical breast examination by an experienced practitioner (physician or nurse).
- All those over 65 should be offered instruction by the Practice Nurse in simple breast awareness programmes.

Regional information

In the United Kingdom the age limit for routine mammography programmes has recently been raised to 65.

Background evidence

USPTF and Canadian Task Force on the Periodic Health Examination recommendations in women 50-69 are mammography every 1-2 years along with clinical breast examination (CBE) (***A***). No clinical trials have evaluated screening (with either mammography or CBE) in women over 70 years of age.
There is sound evidence that among women aged 50-69 screening with mammography every 2 years reduces mortality due to breast cancer by 20-50%. In contrast, a recent analysis of the outcome of mammography screening in Sweden over the past 10 years, showed no significant reduction in mortality from breast cancer, and a substantial rate of false positive diagnoses. The consensus remains at present however that mammography screening programmes are beneficial.
Although the sensitivity of mammography does not decline in older women, a decision analysis of the utility of screening women aged 70 and over has shown that lives saved decreases with advancing age and comorbidity.
Annual clinical breast examination alone presently receives a ***C*** recommendation - sensitivity of CBE varies depending on the experience of the examiner, ranging from 65% for registered nurses to 87% for primary care physicians for lumps 1 cm in diameter.
Teaching breast self examination also receives a ***C*** recommendation - the sensitivity of breast self-examination is in the range of 26% to 40%, with decreasing values seen with increasing patient age.

References

Fletcher SW et al. Physicians' ability to detect lumps in silicon breast models. JAMA 1985;253:2224-2228
Nystrom L et al. Breast cancer screening with mammography; overview of Swedish randomised trials. Lancet 1993;341:973-978
Sjonell G et al. Mammographic screening does not reduce breast cancer. Lakartidningen 1999;96(8):904-5,908-13

Cervical cancer

Basic principles

Benefits of cervical smear screening every 5 years are shown in women aged 30-70, with a reduction in cervical cancer mortality. The impact of screening on risk is however greater in young versus older women.

Recommendations

- Women aged 70 and over who have had 3 previous negative tests and are asymptomatic can be largely reassured, with no further cervical smears required.
- In those over 65 who have never had cervical cytology, or where there has been a lapse of 5 years or more in testing up to the age of 65, consideration should be given to carrying out this test in the practice.

Background evidence

USPTF guidelines state that all women with a cervix and who are or have been sexually active (***A***) should be screened at least every 3 years (***B***). There is little evidence for better outcome of annual screening. USPTF recommendations are to stop after age 65 if smears were consistently normal in regular previous screening (***C***).

The relative risk for mortality from cervical cancer is lowest in women aged over 70 years, as compared with younger age groups. The national cervical screening programme for women aged 30-74 in the UK resulted in an overall falls in incidence of invasive disease, but the falls in mortality in older women (aged over 55) were largely unrelated to screening.

References

Sasieni P.Effect of screening on cervical cancer mortality in England and Wales: analysis of trends with an age period cohort model. BMJ 1999;318:1244-1245
Quinn M et al. Effect of screening on incidence of and mortality from cancer of cervix in England: evaluation based on routinely collected statistics BMJ 1999;318:904

Prostate cancer

(Note: prostate cancer screening is not covered in the HRA-O)
Early detection of prostate cancer by means of the prostate specific antigen PSA) test is gaining ground, but not yet the basis of an agreed evidence-based screening strategy. Consideration should be given to undertaking this test in men presenting with symptoms. A request for general advice on preventive care would normally trigger implementation of any nationally based protocol (e.g. existing 75 + screen in the United Kingdom) and/ or coverage of items in the HRA-O.

D. HEALTH MAINTENANCE

Dental Care

Basic principles

Dental problems are a source of morbidity and suboptimal nutrition in late life. Numerous studies have demonstrated that many older adults have problems chewing, pain, difficulties in eating, and problems in social relationships because of oral disorders.

Recommendations

- Every effort should be made to encourage each older patient to undergo an annual dental check.
- Encourage patients to seek early dental care where problems are identified through the HRA-O

Regional information

United Kingdom: NHS dentistry is increasingly hard to locate and it is recognised that there are financial disincentives. Less than half of the community population in the UK are "regular dental attenders", with non-attendance being associated with older age, fewer self-reported number of teeth possessed, edentulous status, and low income and other socio-demographic factors.

Background evidence

Periodontal diseases and recurrent caries are the predominant causes of tooth loss in older people; both problems are preventable. Oral mucosal lesions of all types are increasingly prevalent with age, affecting up to 30% of the elderly population. Individuals at greater risk of poor oral health are diabetics, smokers, and people taking medications with the side effect of reducing salivary flow (e.g. antidepressants, antihypertensives, diuretics)

References

McGrath C et al. Factors influencing older people's self reported use of dental services in the UK. Gerodontology 1999 Dec;16(2):97-102

Mandel ID. Preventive dental services for the elderly. Dental Clin N America 1989;33:81-90

Locker D et al. Self-perceived oral health status, psychological well-being, and life satisfaction in an older adult population. J Dent Res 2000 Apr;79(4):970-5

Eyesight testing

Basic principles

There is no evidence based indication for routine ocular examination by physicians in all older people. However, the HRA-O includes a vision screen (VFQ25 - *see appendix (vii)*).

Recommendations

- If the VFQ25 is positive, then an ocular assessment including refraction, intraocular pressure, and examination of the fundi is justified. This will normally involve referral, although a preliminary fundoscopic examination may be carried out by the physician.
- Every effort should be made to encourage each older patient to undergo a yearly (or at least two-yearly) eye test, including a check on intraocular pressures. Conditions such as diabetes and regular steroid intake increase the importance of regular eye tests and should therefore also trigger examination.

- Ocular symptoms of potentially medical significance (including any sudden deterioration in vision) will require the appropriate medical examination and consideration of referral for specialist assessment.

Regional information
In the UK, refraction is normally carried out by opticians, who will also measure intraocular pressures and undertake fundoscopy if requested, and will in addition advise on the need for ophthalmological referral. Thirty percent of a sample of a community population in the UK aged 65 or older were visually impaired in both eyes. More than 72% of the bilateral visual impairment was potentially remediable by surgery or glasses, nearly one in three people had visually impairing cataract, and 88% of these people were not receiving specialist attention.

Background evidence
Evidence for effectiveness of visual screening in older people who do not report visual impairment is lacking, but a small beneficial effect cannot be excluded.
There is evidence that visual impairment is prevalent in older people who fall.

References
Reidy A et al. Prevalence of serious eye disease and visual impairment in a north London population: population based, cross sectional study BMJ 1998;316:1643-1646

Smeeth L, Iliffe S Effectiveness of screening older people for impaired vision in community setting: systematic review of evidence from randomised controlled trials BMJ 1998;316:660-663

Hearing assessment

Basic principles
The commonest cause of hearing impairment in later life is presbyacusis (sensorineural high tone deafness). Hearing impairments are reported by 23% of people aged 65-74, 33% between age 75-84, and 48% by those aged 85 and over. Many older people are also not aware of having hearing loss.

Recommendations
- Screening for hearing loss in people aged 65 and over every 1-2 years by whisper test (whisper 4 numbers to be repeated by the patient 6-12 " away from the ear, whilst blocking the other ear with your finger) (***D***)
- Routine use of the otoscope where hearing loss is present, to exclude treatable otitis and to remove impacted wax
- Referral for audiological assessment with pure-tone threshold audiometry if hearing loss is having an impact on communication, socialisation, function, mood, or quality of life
- Patients should be discouraged from spending large amounts of money on commercial hearing aids until they have had a formal assessment by an audiologist.

Regional information
- No particular regional issues

Background evidence
- Although many older people with hearing loss may not report this symptom to their GP, screening for deafness in primary care (by whisper and vibrating fork tests) is suboptimal even within structured preventive health programmes.

Only 8% of hearing impaired people over 65 use a hearing aid, and one-fifth of users over 85 report impaired hearing despite adequate amplification. Hearing loss in later life can compromise ability to perform Instrumental Activities of Daily Living (e.g. shopping, using telephone, driving), and may lead to social withdrawal and depression. There is some evidence to suggest that improvement of hearing may contribute to improvement in cognition in older people with cognitive impairment.

References

- Freedman A et al. Preventive care for the elderly. Do family physicians comply with recommendations of the Canadian Task Force on Preventive Health Care? Can Fam Physician 2000;46:350-7
- Bess FH et al. Hearing impairment as a determinant of function in the elderly. J Am Geriatr Soc 1989;37:123-8
- Gennis V et al. Hearing and cognition in the elderly: new findings and a review of the literature. Arch Intern Med 1991;151:2259-2264

Exercise

Basic principles

Physical exercise in older people is beneficial both for primary and secondary prevention of cardiovascular morbidity and mortality, and prevention of falls and disability in frailer individuals.

Recommendations

- For patients who are physically inactive but otherwise well the advice and recommendations on levels of activity provided in the HRA-O should be reinforced.
- Where there are no contraindications, aerobic exercise should be promoted to enhance cardiopulmonary fitness (***A***) and general stamina, while resistance training should be advised to improve balance and muscular strength (***A***)
- Regular exercise programmes for older people can particularly benefit pain and disability related to knee osteoarthritis (***B***), weight loss in obese individuals (***A***), and control of hyperlipidaemia (***A***) and hypertension (***A***).
- Patients who are physically inactive receive written advice on exercise, but are also advised to discuss this with their GP, particularly if they intend to make a significant increase in physical activity. They will also have received reinforcement and advice from the health educator.
- Patients undertaking a new exercise programme should be advised to seek medical advice if they have new or worsening exercise-related symptoms such as breathlessness or joint pains.
-

Regional Information

No specific regional issues

Background evidence

There is substantial evidence that physical exercise in older people is beneficial for both primary and secondary prevention of cardiac morbidity and mortality, and the efficacy of exercise in preventing coronary artery disease in men is given a USPSTF Class ***A*** recommendation.

Both aerobic exercise (activity that results in increased heart rate for an extended period of time), and resistance training (the resistance against which a muscle generates force is gradually increased in order to enhance muscle strength), have been shown to improve longevity and reduce debility in older people. The former however is superior in improving

cardiopulmonary fitness, while the latter is more beneficial with regards to improving balance and muscle strength.
In the community, a programme to improve strength and balance in women aged 80 years and older consisting of four home visits from a physiotherapist, reduced falls and moderate injuries over the subsequent year, with benefit being most evident in frequent fallers.

References

Christmas C, Andersen RA. Exercise and older patients: Guidelines for the Clinician. J Am Geriatr Soc 2000;48:318-324

Nutrition

Basic principles

Poor nutrition in older people may result in obesity (affecting 16% of the adult population in the US), or in protein-calorie malnutrition (commonly multifactorial in aetiology).

Recommendations

- The physician and health educator should endeavour to reinforce the detailed recommendations in the HRA-O.
- Basic dietary advice should include eating 5 or more servings of fruit and fibre a day, and taking less than 30% of daily calories as fat by limiting egg yolks to 2 a week, substituting fish and poultry for other meat or cheese, drinking low fat milk, and choosing margarine instead of butter.
- Individuals may require referral for specialist dietary advice at the discretion of their GP, (e.g. for hyperlipidaemia, diabetes, obesity).

Regional Information

Overall, people aged 65 and over living in the community in the UK meet dietary recommendations for total fat intakes, vitamins and minerals, but not for saturated fat or fibre.

Background evidence

A healthy diet, as measured by an indicator based on WHO recommendations, has been associated with a reduction of 13% after 20 years in all cause mortality for Western European men aged 50-70. Dietary intervention has been shown to be effective in management of hyperlipidemia, hypertension, and cardiac disease (A). There is evidence however that while primary care practitioners recognise the value of dietary counselling in older people, multiple barriers to implementation exist, including lack of time, patient non-compliance, inadequate teaching materials, and lack of specialised knowledge.
Acute illness, especially when resulting in hospitalisation, accelerates nitrogen wasting and protein malnutrition in older people. Other modifiable causes of protein-calorie malnutrition in the elderly are depression, medications, congestive heart failure, malabsorption syndromes, chronic stool impaction, and social factors (immobility or isolation resulting in limited access to food, poverty).

References

National Diet and Nutrition Survey of people aged 65 and over. Department of Health Food Safety Information Bulletin 1998;103 (Dec):1-2
Hushner RF. Barriers to providing nutrition counselling by physicians: a survey of primary care practitioners. Prev Med 1995 Nov;24(6):546-52
Huijbregts P et al. Dietary pattern and 20 year mortality in elderly men in Finland, Italy, and the Netherlands: longitudinal cohort study. BMJ 1997;315:13-17

Physical function and independence

Basic Principles

Early multidisciplinary intervention in managing functional impairment in older people improves both functional and psychosocial outcomes (***B***). Recent work has focused on prevention by assessing early predictors of physical disability with ageing (so-called preclinical mobility disability), and the HRA-O aims to Identify these markers.

Recommendations

- The HRA-O report encourages patients to seek medical advice if they report functional disability, particularly if this is new. Practical advice is provided to the patients in matters of mobility, domestic activity and social function. This will require reinforcement and advice. Medical assessment is appropriate where the decline in function is sudden or unexplained.
- The HRA-O will identify patients with preclinical markers of disability who do not report reduced mobility, but do report needing to modify methods of doing tasks such as getting into or out of a car, climbing steps etc. These patients merit evaluation for chronic medical problems that may have a worsening impact on function.
- Comprehensive multidisciplinary geriatric assessment (medical, nursing, physiotherapy, occupational therapy)(CGA)(see UK note below) is indicated for patients newly reporting significant functional problems such as needing help with more than one basic activity of daily living.
- For the longer term disabled, access to remedial and support services will require information and/or referral to relevant agencies (both statutory and voluntary) concerned with disability and social support.

Regional Information

CGA in the UK will normally be provided via outpatient referral to hospital- based Departments of Geriatric Medicine.

Background evidence

A Canadian community based study of Screening for multidimensional functional capacity in patients aged 65 years and over, followed by appropriate referrals (e.g. to physiotherapy) showed an improvement in daily activities, mental health scores, and social functions in people aged over 75 years who were living alone or lonely. The intervention was less effective in improving total functional capacity of individuals without these sociodemographic factors.
A prospective study of women aged 70-80 without mobility difficulty identified self-report of method of doing a task as a strong predictor of mobility disability 18 months later. These indicators are included in the HRA-O, and provide a potential basis for screening for disability risk, and for targetting interventions to prevent mobility disability.

References

Hay WI et al. Prospective care of elderly patients in family practice. Is screening effective? Can Fam Physician 1998;44:2677-87
Fried LA et al. Preclinical mobility disability predicts incident mobility disability in older women. J Gerontology 2000;55A(1):M43-M52

E. SPECIFIC MEDICAL HEALTH ISSUES

Pain

Basic principles

Chronic pain is a major problem among older people living in the community, and is associated with a high level of primary care physician visits. Chronic pain in elderly people may lead to a downward spiral of depression, social isolation, sleep disturbance, and reduced mobility. Failure to evaluate pain in this age group is a critical factor leading to under treatment. Conversely, suboptimal assessment may also lead to inappropriate prescribing of analgesic medications such as nonsteroidal anti-inflammatory drugs (NSAID's) and opiates.

Recommendations

- Routine inquiry about pain should be part of every new health care professional encounter with older patients.
- Any pain complaint that has an impact on physical or psychosocial function needs to be addressed as a significant problem, often involving early use of pharmacological treatment.
- Pain in older people should invariably trigger a careful diagnostic search, including a careful history, physical examination and any indicated investigations (e.g. for osteoporosis). Its control is most effective when the underlying cause of pain can be identified and treated and its consequences addressed through a careful multidisciplinary assessment. Where the cause remains in doubt, appropriate specialist referral should occur.
- Simple patient self-assessment pain scales should be used to quantify pain (e.g. verbal or visual analogue scale: "On a scale of zero to ten, with zero being no pain at all, and 10 being pain as bad as you could possibly imagine, how much pain are you having now?") (***D***)
- Regular review of patients taking analgesic medications more than 3 times a week (as per HRA-O) is recommended. (***D***)
- Paracetamol (up to 1000 mg four times a day) is the drug of choice for most older people with mild to moderate pain, especially when the cause is identified as being osteoarthritic (***A***) or musculosketal (***D***)
- Use of NSAID's in older people should be limited to treating active inflammatory conditions or bone pain where there are no contraindications (renal impairment (***A***), upper gastrointestinal disease (***A***), congestive heart failure), and where alternative analgesic drug strategies (e.g. low-dose steroids or opiates) have failed (***D***). NSAID's should be used for short periods of time only in the elderly.
- Improved gastrointestinal tolerance with selective inhibitors of cyclo-oxygenase 2 inhibitors is unproven in the elderly, so a cautious approach to their use similar to NSAID's, is recommended in this population.
- Constipation is a major problem for older people on any type of opiate analgesia (***C***), and a bowel programme should be started early on in a treatment course.

Regional Information

No particular regional issues

Background evidence

Chronic pain is a major problem in the community, reported by 46.5% of a primary care population; a significant relationship is seen between advancing age and self-report of pain. Back pain and arthritis account for one third of all complaints. Despite this high

prevalence, people aged 65 and over have been systematically excluded from clinical trials of pain treatment, so current guidelines for the elderly population are largely based on consensus opinion.
Use of the patient-administered Pain Assessment instruments in primary care patients has been shown to lead to greater patient satisfaction with physician-patient communication regarding pain complains, regardless of the specific nature or cause of their pain.

References

Elliot AM et al. The epidemiology of chronic pain in the community.Lancet 1999;354:1248-52
Radecki SE et al. Randomised clinical trial of diagnostic instrument for pain complaints Fam Med 1999;31:713-721

Medication use

Basic principles

Written advice within the HRA-O encourages patients to discuss all decisions about medication with their GP, particularly if they regard their medication as unimportant, if cost is an issue or if they are concerned about side effects or interactions. To assist with compliance with medication regimens, patients on 3 or more medications are encouraged to keep a medication log. This should facilitate any review consultation and assist in any advice from GP or health educator on compliance.
The HRA-O collates information on medical symptoms with prescribed medication. Where possible drug side effects are flagged, patients are encouraged to discuss this possibility with their doctor.
Similarly, possible omissions based on well-recognised standard indications (e.g. thromboprophylaxis in atrial fibrillation) may also be flagged.

Recommendations

- Patients will have already received appropriate reinforcement and advice from the research health educator, but may request a review of medication with their GP.
- Patients should be advised always to bring their complete drug supplies to any and every medical consultation.

Where there is concern about compliance, measures would normally include:

- any possible steps to reduce the number and/or complexity of drug regimens (including discontinuation of drugs no longer indicated)
- to discuss and explain clearly the reasoning behind any treatment,
- to consider the use of compliance aids
- to enlist the help of a third party where necessary.
- Where possible medication side effects or possible omissions are flagged by the HRA-O, a drug review should be carried out.

Regional information

No specific regional issues

Background

A recent article evaluating potentially inappropriate medication use in older people rated the following medications as being very high risk for causing adverse events in this population: amitriptyline (because of it's strong anticholinergic and sedating properties, it should not be antidepressant of choice in elderly), and long-acting benzodiazepines chlordiazepoxide, flurazepam, and diazepam (because of prolonged sedation and increased risk of falls and injury).

References

Beers MH. Explicit criteria for determining potentially inappropriate medication use by the elderly. Arch Intern Med 1997;157:1531-1536

Injury prevention and falls

Basic principles

One third of people over the age of 65 fall each year. Falls are the major cause of accidental death, particularly in this age group. 5-10% of falls result in a fracture and 2.2% of injurious falls are fatal. Those not causing fractures are still associated with substantial morbidity and have considerable psychological consequences. Falls in older people are often a signal of underlying health-related problems or risk factors and are commonly due to a combination of these with environmental hazards. Early and systematic assessment of risk with appropriate intervention can reduce the incidence of falls (***A***)

In addition to a history one or more falls, accepted known risk factors in older people include:

- Readily detectable impairment of balance, gait or mobility
- Polypharmacy, in particular centrally sedating or blood-pressure lowering medications
- Visual impairment
- Impaired cognition or depression
- Present or past history of stroke, Parkinson's disease or degenerative lower limb joint disease
- Postural hypotension

Measures to reduce the risk of falls in late life are an area of growing sophistication, mainly in terms of multidimensional assessment and specialist syncope clinics. Individuals with known risk factors for recurrent falling, in particular a history of falls, are encouraged in their HRA-O report to seek medical advice.

Recommendations

- Physicians should elicit any evidence of the above and take the corresponding appropriate action where possible.
- Individuals with a history of recurrent falls or with two or more of the above risk factors should be referred for specialist comprehensive geriatric assessment (CGA) if not already undertaken and if locally available.
- Known high risk factors for osteoporosis are: radiographic evidence of osteopenia, loss of height associated with osteopenic vertebral deformity, previous fragility fracture, prolonged corticosteroid therapy, chronic disorders associated with osteoporosis, history of premature menopause; history of maternal hip fracture, and low body mass index. These constitute accepted indications for assessment (bone densitometry in certain cases to evaluate fracture risk and specialist referral where appropriate). Treatment options are non-pharmacological measures including increasing dietary intake of calcium, avoidance of smoking, excess alcohol and caffeine, proceeding to pharmacological treatment with calcium and vitamin D, Didronel or hormone replacement therapy, depending on the individual's risk profile and severity of disease.
- The physician and/or health educator should advise on falls prevention (e.g. improved lighting at home, removal of loose carpeting, non-skid mats in bathtub, grab rails where needed).

- The health educator should also advise concerning non-falls-related injury prevention [e.g. safe cooking, not smoking in bed, awareness of emergency exits in case of fire, seat-belt use, cautious driving (during day, familiar routes only, avoid right hand turns at busy intersections)].

Regional information

United Kingdom:

Falls prevention is a major element of the current NHS National Framework for the Care of Older People and most districts are developing specialist falls and syncope assessment clinics for referral as well as primary care guidelines.
High risk factors for osteoporosis are defined in guidelines from the National Osteoporosis Society and the Royal Colleges of Physicians

Background evidence

Assessment of older people for multiple falls risk factors with appropriate intervention has been clearly shown to reduce the subsequent incidence of falling in robust randomised controlled clinical trials (***A***). Using a postal questionnaire screen with follow-up assessment of those so identified as at high risk resulted in a 30-40% reduction. Opportunistic specialist assessment of those presenting in accident and emergency within two weeks of the index fall resulted in a 60-70% reduction in the number of falls in the subsequent year within the intervention group compared with controls.

References

Tinetti ME, Speechley M, Ginter SF (1988). Risk factors for falls among elderly people living in the community. N Engl J Med; 319:1701-1707.
Tinetti ME, Baker DI, McAvay G, et al. (1994). A multifactorial intervention to reduce the risk of falling among elderly people living in the community. N Engl J Med; 331:821-827.
Close J, Ellis M, Hooper R, Glucksman E, Jackson S, Swift C. Prevention of Falls in the Elderly Trial (PROFET). A Randomised Controlled Trial. Lancet 1999;353: 93-97
Campbell JA. Randomised controlled trial of a general practice programme of home based exercise to prevent falls in elderly women BMJ 1997;315:1065-1069

Urinary incontinence

Basic principles

The prevalence of urinary incontinence increases with age affecting up to 44% of people aged 75 years and over living in the community. Primary care physicians tend to have a low awareness of incontinence in older people. Overall the assessment and management of continence problems in older people continues to be inadequate in the majority of sufferers.

Recommendations

- Patients reporting recurrent loss of bladder control on the HRA-O should be evaluated.
- Assessment should start with a mid-stream urine culture. If incontinence persists in the setting of sterile urine, proceed to a more detailed history to seek a diagnosis of detrusor instability or drug-related incontinence.

Women should further be evaluated by pelvic examination (e.g. for clinically significant prolapse) and bladder palpation (for urinary retention in the absence of faecal impaction). Referral for specialist geriatric medical, gynaecological or urological assessment (including urodynamic studies) should be seriously considered (see UK note below). Established stress incontinence where surgery is not indicated may be treated with pelvic muscle exercises, local oestrogen therapy, or low-dose imipramine). Detrusor instability may (if the diagnosis is certain) be treated with bladder retraining, tolterodine or both.

- Further evaluation for men is aimed at identifying overflow incontinence due to benign or malignant prostatic enlargement (digital rectal examination and bladder palpation), as well as excluding bladder malignancy. Specialist urological referral should be seriously considered in view of the importance of early diagnosis in each case.

Background evidence

The high prevalence of urinary incontinence in older people is due largely to age-related changes (such as decreased bladder capacity, increased disinhibited bladder contractions [detrusor instability]), increased nocturnal sodium and fluid excretion, decreased urethral resistance in post-menopausal women, increased urethral resistance in men with prostatic enlargment, and weakness of the pelvic floor in women) combined with predisposing factors such as comorbidity, medications, and immobility.
Assessment should aim to identify the type of incontinence - functional, drug-related, stress, urge or overflow, and to apply appropriate treatment plans. Such assessment commonly requires specialised techniques.

Regional Information

UK: Specialist assessment of older people with continence problems in the NHS is widely available but variably provided by Departments of Geriatric Medicine, Urogynaecology, Gynaecology or Urology. GP's will need to ascertain the best line of referral in their locality.

References

Prosser et al. Case-finding incontinence in the over-75's. Br J Gen Pract 1997;47:498-500
Ouslander JG, Johnson TM. Incontinence. Ch 126 pp1595-1614. In: Principles of Geriatric Medicine and Gerontology: Hazzard W et al. 4th edition 1998.

F. PSYCHOSOCIAL HEALTH AND BEHAVIOUR

Screening for depression and dementia

Basic principles

The HRA-O questionnaire and report profiles are configured in such a way as to assist with the early identification of both depression and dementia. In both cases, detailed advice is provided (for patients as well as carers) to encourage early presentation for diagnosis, treatment and support.

Recommendations

- HRA-O response indicative of self-reported memory loss should prompt evaluation by Mini-mental state examination *(see Appendix (viii))*. A score below 25 is indicative of probable cognitive impairment, and the patient should be further evaluated for reversible (e.g. B12 deficiency, hypothyroidism) and irreversible (e.g. vascular, Alzheimer's type) dementia (see UK note below). Specialist referral should be considered.
- HRA-O response indicative of low mood should prompt completion of the Abbreviated Geriatric Depression Scale *(see Appendix (ix))*. This 15-item questionnaire takes 2 minutes to administer, and covers a broad range of questions about mood. It is used to screen for undetected depression, establish a baseline description, and monitor progress. A score of 11 or greater is consistent with the diagnosis of depression.
- Direct intervention will be at the clinical judgement of the GP, including initiation or review of medication, referral to local support services or referral for specialist assessment.

Regional information

UK: Detailed assessment of older people with impaired cognition is undertaken in the NHS in Departments of Psychiatry of Old Age or Geriatric Medicine, either of whom may provide a "Memory Clinic "for early diagnosis and assessment. GP’s should ascertain the best line of referral in their locality. Departments of Psychiatry of Old Age will also provide specialist advice and support in the diagnosis and management of depression.

Background *evidence*

The use by non-medical staff of a brief screening instrument (the mini-mental state examination) to detect cognitive impairment among older people in primary care was described in 1170 patients aged 75 years and over in a UK primary care practice. The prevalence of cognitive impairment (score below 25 on the mini-mental state examination) was 12.8%. Six percent of patients scored below 19, yet less than a third of this group had a diagnosis of dementia in their medical records. There was no association between low MMSE scores and gender or social class, but a relationship was observed with age - the proportion with dementia rose from 2.5% in those aged 75-79 years to 29.0% among those aged 90 years and over. Similar prevalence of cognitive impairment was observed in a larger US survey of screening primary care patients aged over 60 years. Moderate to severe impairment was associated with increased use of health services and increased mortality.

Despite positive attitudes about their skills for detecting and treating depression in the elderly, only one quarter of primary care physicians routinely used a screening tool in practice in a US study. A UK survey of screening for depression in patients aged 75 and over found that 22% had some evidence of depression previously undiagnosed by the primary care physician.

References

Iliffe S et al. Screening for cognitive impairment in the elderly using the mini-mental state examination. Br J Gen Pract 1990;40:277-9
Callahan CM et al, Documentation and evaluation of cognitive impairment in elderly primary care patients. Ann Intern Med 1995;122:422-9
Banazak DA. Late-life depression in primary care. How well are we doing? J Gen Intern Med 1996;11:163-7
Iliffe S et al. Assessment of elderly people in general practice. 1. Social circumstances and mental state. Br J Gen Pract 1991 Jan;41(342):9-12

Smoking

Basic Principles

Up to 30% of people over 65 years of age are current smokers. Respiratory problems, primarily due to smoking, are the second commonest cause of disability in old age. Smoking cessation strategies in young elderly have been shown to be efficacious and cost-effective in terms of cost per life year gained, yet recent studies have shown that doctors are less likely to giving advice on smoking cessation to older patients as compared with younger ones.

Recommendations

- Substantial information and written advice are provided to older smokers in the HRA-O. This smoking cessation advice should be reinforced by the health educator and physician, with emphasis placed on demonstrated benefits of quitting on respiratory and cardiovascular systems.
- The physician should work with the older patient to agree realistic targets (as an incentive) and where there are no contraindications (and if health-economically feasible) should consider the prescribing of nicotine patches, gum, or inhalers.
- Advice on any local stop smoking programmes should be made available.

Regional Information
UK: Nicotine replacement therapy is now available on NHS prescription in Health Action Zones.

Background Evidence

As for younger people, the most important predictor of successful smoking cessation (i.e. for at least 1 year) in older individuals is motivation. Counselling programs can achieve a cessation rate of 15% in well-motivated older people, increasing to 20% when combined with nicotine replacement therapy (***A***). Concommitant exercise programmes have also been shown enhance the impact of cognitive-behavioural programmes on long-term smoking cessation in women (***A***).
Where data is available, the benefits of smoking cessation appear to be comparable in young and in old. The beneficial impact of cessation on accelerated decline of lung function with smoking has been demonstrated up to the age of 80, especially in women. The decline in risk of myocardial infarction after stopping smoking (to virtually pre-smoking levels after 2-3 years) is also unaffected by ageing.
Data from the US suggests that older smokers are more likely to reject evidence that smoking is bad for the health, but whether these attitudes are prevalent in older smokers in Western Europe is unclear.

References

Connoly JM. Smoking cessation in old age: closing the stable door? Age and Ageing 2000;29:193-5

Vetter NJ, Ford D. Smoking prevention among elderly people aged 60 and over: a randomised controlled trial. Age and Ageing 1990;19:164-8

Alcohol use

Basic principles

The scale of alcohol problems amongst older people is widely underestimated. The continuation of a steady level of drinking may become problematic with ageing because of age-related changes in physiology and pharmacodynamics that increase sensitivity to the deleterious effects of alcohol.

Recommendations

- Reinforcement of advice in the HRA-O report may be necessary.
- Relevant points in the medical history in excessive drinkers are recurrent falling, insomnia, malnutrition, incontinence, social isolation, memory loss, anxiety and depression.
- Physical examination should include assessment for evidence of trauma, cardiomyopathy (including atrial fibrillation), peripheral neuropathy, and features of chronic hepatic disease.
- Elevated levels of gamma GT and AST indicative of hepatocellular damage are the most sensitive laboratory tests for chronic alcohol abuse. The MCV is commonly raised. Anaemia in alcohol users is often multifactorial and may be associated with thrombocytopaenia.
- Be aware of potential drug/alcohol interactions e.g antihypertensives, opiate analgesics, warfarin, antidepressants, anticonvulsants
- Intervention would be in accordance with the GP's clinical judgement. Short-term interventions may include treatment for underlying depression, treatment of malnutrition and coexisting metabolic disorders, and enlisting support from family members to help overcome patient denial. Long-term interventions may include referral to an appropriate alcohol detoxification outpatient or inpatient programme, and awareness of self-help groups.

Regional information

No specific regional issues

Background evidence

Between 5% and 12% of men and 1-2% of women in their 60s are problem drinkers. The rates are substantially higher among hospital outpatients and people attending clinics. A study of 1070 elderly men and women selected from general practice lists showed that nearly one fifth of both sexes who were regular drinkers exceeded the recommended limits.

Both the quantity of alcohol drunk and the frequency of drinking by elderly men - and so the frequency of problems related to alcohol - are higher than those in elderly women. On average elderly people drink less than younger people, but ageing does not always modify drinking behaviour, and excessive alcohol use may simply be carried into old age. The trend for elderly people to reduce alcohol consumption seems to be less noticeable in women.

Elderly people may be less tolerant of the adverse effects of alcohol. The mechanisms are not clear, but may include an increase in distribution volume (due to an increase in the ratio of body fat to water), a reduction in hepatic first-pass metabolism and/or an increase in CNS sensitivity to alcohol.

The current US National Institute on Alcohol Abuse and Alcoholism recommendations for safe limits of alcohol consumption have been adjusted downwards for people over 65 to no more than one drink a day, because of their particular vulnerability to its toxic effects.

References

Dunne FJ. Misuse of alcohol or drugs by elderly people. BMJ 1994;308:608-609
Dufour MC, Archer L, Gordis E. Alcohol and the elderly. Clin Geriatr Med 1992;8:127-141

HEALTH RISK ASSESSMENT, PREVENTION AND HEALTH MAINTENANCE GUIDELINES FOR PERSONS AGED 65 AND OVER

Use of these guidelines - *All* patients aged 65 and over should receive primary preventive health care. Reinforced health education by the GP and/or Practice Nurse should be provided to patients in whom health risks are identified through completion of HRA-O questionnaire or through routine enquiry

**Domains**	**Primary Prevention**	**Risk Assessment and Health Maintenance**
Hypertension	Yearly screen	SBP ≥ 160 or DBP ≥ 100 - treat to achieve goal of BP ≤ 140/85 with thiazides first-line, aspirin if high cardiac risk plus in all cases diet, exercise, limit salt intake
		BP 140-159/90-99 - treat if high cardiac risk, diabetes, end-organ damage; otherwise observe only
Hyperlipidaemia	5 yearly screen / Coronary Risk Prediction chart	Total cholesterol ≥ 5.0 + high cardiac risk - if cholesterol still ≥ 5 after 3-6 months of lifestyle measures (diet, exercise, weight reduction, smoking cessation) - treat with statins
Diabetes mellitus	3 yearly screen with fasting blood glucose (FBG)	FBG ≥ 7.0 mmol/L - treat with diet, glicazide is preferred sulphonylurea, multidisciplinary approach to promote compliance, modify individual treatment goals according to comorbidity
Influenza Vaccine	Administer yearly to all patients	Influenza and pneumococcal immunisation is an effective health maintenance intervention in both low and high risk patients.
Pneumococcal Vaccine	Administer 10 yearly to all patients	
Colon cancer	Annual screening by FOBT	…but only if back-up with specialist investigations (e.g. colonoscopy) is available
Breast cancer	Biannual screening mammogram to age 69	…if available, plus annual clinical breast exam by experienced practitioner
Cervical cancer	5 yearly screening (cervical smear)	Discontinue screening at age 70 if 3 previous smears negative
Dental care	Recommend annual dental check	To all patients
Eyesight testing	Recommend yearly eye test	To all patients, specialist referral where undiagnosed problems identified through HRA-O
Hearing assessment	Annual whisper test	Auroscopy and audiometry referral to evaluate hearing loss
Exercise	Recommend regular exercise ± limits	Limits should not be age-dependent, encourage goal-setting based on prior activity level, physical functionning, patient motivation and preferences regarding exercise
Nutrition	Basic dietary advice	≥5 servings fruit and fibre daily, reduce saturated fats. Identify causes of malnutrition
Physical function	Periodic functional assessment for patients with comorbidity	Evaluate where preclinical markers of disability identified through HRA-O, multidisciplinary assessment for those needing help with more than one basic ADL
Pain	Routine enquiry each patient encounter	Thorough assessment to avoid both under-treatment, and over-treatment (e.g. with NSAID's)
Medication use	Monthly review for ≥3 medications	Prescribing appropriateness, compliance aids, avoid long-acting benzo's and amitryptiline
Injury prevention/falls	Annual falls risk assessment	Education re: falls and injury prevention, comprehensive assessment in fallers
Urinary incontinence	Routine enquiry	Assess for UTI, functional, drug-related UI - refer for diagnosis of overflow, stress, urge UI
Depression/dementia	Screen patients ≥ 75 years	Further evaluation for dementia if MMSE <25 and for depression if GDS >11
Smoking	Cessation counselling for all smokers	Cognitive-behavioural approach ± nicotine replacement therapy
Alcohol use	Routine in-depth enquiry	Safe limit 1 drink/day (less on neurotropic drugs), HRA-O may underestimate hazardous drinking

S/DBP=systolic/diastolic blood pressure LTC=long-term care FOBT=faecal occult blood test ADL=activities of daily living UTI=urinary tract infection UI=urinary incontinence MMSE=minimental status exam GDS=geriatric depression scale.
