# Supplementary material for: Effect of Health Risk Assessment and Counselling on Health Behaviour and Survival in Older People: A Pragmatic Randomised Trial
Source: PLoS Med. 2015 Oct 19;12(10):e1001889. doi: 10.1371/journal.pmed.1001889 (PMC4610679; doi:10.1371/journal.pmed.1001889)
Supplement: S6 Text — (PDF) [file pmed.1001889.s018.pdf]

Supporting Information S6 Text

PRO-AGE Solothurn Intervention Material (German language version)

for: A Collaborative Care Model of Health Risk Assessment and Counselling in Older Persons: A Randomised Clinical Trial

PRO-AGE: Interventionsmanual

(PRevention in Older persons- Assessment in GEneralists' practices)

UNTERLAGEN FÜR DIE AN DER INTERVENTION BETEILIGTEN HAUSÄRZTINNEN UND HAUSÄRZTE

Hinweis: Dies ist ein vertrauliches Dokument, das nur zur Verwendung in den beteiligten Praxen bestimmt ist.

Autoren: C.G. Swift, D. Harari, S. Iliffe, N. Lübke, T. Münzer, A.E. Stuck

Danksagung: Dieses Projekt wird finanziell unterstützt von der Europäischen Union (CTR_QLK6-CT-1999-02205). Die Autoren danken Herrn Prof. J.C. Beck, MD für seine Kommentare zu einer früheren Version.

Dieses Manual wurde aus dem Englischen übersetzt und auf regionale Aspekte der Schweiz angepasst. Die Autoren danken Herrn J. Weiz für seine Mitwirkung bei der Übersetzung. Im Interesse der Einfachheit des Textes wird auf eine Schreibweise verzichtet, die immer beide Geschlechter berücksichtigt.

© Dieses Dokument ist als Ganzes, oder auch in Teilen urheberrechtlich geschützt.

Inhaltsverzeichnis

1. DAS HRA-O (HEALTH RISK APPRAISAL FOR OLDER PERSONS)........................... 3

A. DER GESUNDHEITSPROFIL-FRAGEBOGEN (ANHANG II) 3
B. DER PERSÖNLICHE GESUNDHEITSBERICHT (ANHANG III).. 4
C. DER ZUSAMMENFASSENDE BERICHT AN DEN HAUSARZT (ANHANG IV) 5

2. BERATUNG UND INTERVENTION IN DER ALLGEMEINPRAXIS 5

A. GRUNDLAGEN 5
B. DIE ROLLE DES HAUSARZTES... 7
C. DIE ROLLE DER GESUNDHEITSBERATUNG 7

3. EVIDENZ-BASIERTE EMPFEHLUNGEN ZUR PRÄVENTION UND GESUNDHEITSFÖRDERUNG IM ALTER.. 8

A. VORBEUGUNG GEGEN HERZ-KREISLAUF RISIKEN 9
Hypertonie 9
Hyperlipidämie... 11
Diabetes mellitus 12
B. IMPFUNGEN 14
Grippeimpfung 14
Impfung gegen Pneumonie 15
C. KREBSVORSORGE 17
Kolonkarzinom.. 17
Mammakarzinom.. 18
Zervixkarzinom 19
Prostatakarzinom 20
D. AUFRECHTERHALTUNG DER GESUNDHEIT... 21
Zahnpflege.. 21
Augenuntersuchungen 22
Prüfung des Hörvermögens 23
Körperliche Bewegung 24
Ernährung... 25
Funktionelle Fähigkeiten und Unabhängigkeit... 27
E. BESONDERE MEDIZINISCHE GESUNDHEITSPROBLEME.. 28
Schmerzen 28
Verwendung von Medikamenten 30
Vorbeugung gegen Stürze und Verletzungen... 31
Urininkontinenz.. 33
F. PSYCHOSOZIALE GESUNDHEIT UND VERHALTEN 35
Screening auf Depression und Demenz... 35
Rauchen 36
Alkoholkonsum 38

4. ANHANG (ORDNER) 40

I Tabellarische Zusammenfassung der Empfehlungen
II Der Gesundheits-Profil Fragebogen
III Der Gesundheitsbericht
IV Der zusammenfassende Bericht für den Hausarzt
V Ausgewählte Assessment-Instrumente
VI Ausgewählte Literatur
VII Diverse Projektinformationen

1. Das HRA-O (Health Risk Appraisal for Older Persons)

A. Der Gesundheitsprofil-Fragebogen (Anhang II)

Der Gesundheitsprofil-Fragebogen basiert auf einem validierten Fragebogen nach dem Selbstausfüllerprinzip.

Aufgrund der Antworten im Fragebogen generiert ein Computerprogramm einen individuellen Bericht für die teilnehmende, ältere Person und einen zusammenfassenden Bericht an den behandelnden Hausarzt.

Der Fragebogen deckt Domänen ab, die entweder das Risiko einer funktionellen Einschränkung erfassen, oder ein potentielles Gesundheitsrisiko für die ältere Person darstellen. Durch eine spezifische Intervention sollen folgende Ziele erreicht werden:

- Abnahme des Risikos einer funktionellen Einschränkung
- Verbesserung der Lebensqualität

Die gewählten Domänen haben folgende Gemeinsamkeit:

1. Identifikation von Faktoren mit potentiell negativem Einfluss auf Körperfunktion, Lebensqualität und psychosoziales Netzwerk
2. Erfassung von Faktoren, die durch gezielte Intervention modifiziert werden können
3. Einsatz valider Instrumente, die ausreichend hohe Sensitivität, Spezifität und Reliabilität bieten
4. Einsatz von Strategien, die im Hinblick auf Kosten und Akzeptanz bei der Zielbevölkerung durchführbar sind

Aufgrund dieser Überlegungen wurden folgende Domänen und Instrumente gewählt:

Domäne	Instrument
Medizinische Vorgeschichte	Eigene Angaben über chronische Krankheiten
Masse und gesundheitliche Daten	Eigene Angaben über Grösse, Gewicht, Blutdruck und Cholesterin
Medikamente	Monitoring in the Elderly old Drug Related Symptoms Questionnaire
Anzeichen und Symptome	Fragebogen zur Erfassung medizinischer Symptome und medikamenten-induzierter Nebenwirkungen
Schmerzen	Multidimensional Pain Assessment Instrument
Zahn-und Mundhygiene	Geriatric Oral Health Assessment Index
Sehen	Visual Functioning Questionnaire
Hören	Hearing Handicap Inventory for the Elderly
Psyche und Wohlbefinden	5-item Mental Health Inventory Screening Test, Memory Self Report
Soziales Umfeld	Lubben Social Network Scale
Selbsteinschätzung der Gesundheit	Eigene Angaben zur Selbsteinschätzung Gesundheit
Funktion	Aktivitäten des täglichen Lebens (ADL), instrumentelle und erweiterte ADL
Rauchen	Partners in Prevention Tobacco Use Questionnaire
Alkoholkonsum	The WHO Alcohol Use Disorders Identification Test
Körperliche Aktivität	Physical Activity Scale for the Elderly
Ernährung	Cholesterol Reduction in Seniors Program Fat and Plant Food Screening Questionnaire
Vorbeugung gegen Verletzungen	Study of Osteoporotic Fractures Research Group Survey
Gesundheitsvorsorge	Eigene Angaben über durchgeführte Vorsorgemassnahmen

Ältere Personen, die an der Intervention teilnehmen, erhalten je nach Gesamtdauer des Projektes den Fragebogen in jährlichen Abständen. Das Interventionsteam unterstützt die Hausärzte bei der Erfassung der Studienteilnehmer und erstellt EDV-gestützt den Gesundheitsbericht und den zusammenfassenden Bericht für den Hausarzt. In einzelnen Interventionsgebieten werden die Hausärzte bei der Umsetzung von Empfehlungen durch Mitglieder durch ein Beratungsteam unterstützt.

B. Der persönliche Gesundheitsbericht (Anhang III)

Das HRA-O Computerprogramm generiert aufgrund der Analyse der Antworten aus dem Fragebogen einen persönlichen Gesundheitsbericht. Dieser enthält individuelle und allgemeine Empfehlungen und ergänzende Erläuterungen.

Die direkten Informationen und Hinweise zum Gesundheitsbericht umfassen:

- Beratung zu persönlichem Gesundheitsverhalten in Bereichen, in denen die Patienten selbst ihr Verhalten verändern können (z.B. Rauchen, körperliche Aktivität, Ernährung)
- Hinweise auf weitere Informationsquellen zu einem Thema (z.B. Adresse einer Organisation, eine Fachbroschüre, Angabe einer Internetadresse)
- Hinweise an den Patienten, ein bestehendes Problem mit dem Arzt zu besprechen

Das folgende Inhaltsverzeichnis zeigt die Abschnitte und Zielsetzungen des persönlichen Gesundheitsberichtes.

**Abschnitt**	**Erfahren Sie mehr über**
Gesundheitsvorsorge	Ihre persönliche Checkliste
Medikamentengebrauch	Medikamententipps und Medikamentenplan
Schmerzen	Möglichkeiten der Schmerzbehandlung
Zahn und Mundhygiene	Zahnpflege im Alter
Sehvermögen	Altersbedingte Sehstörungen
Hören	Hörstörungen im Alter
Psyche und Wohlbefinden	Ihre Stimmung und Ihr Gedächtnis
Psyche und Wohlbefinden	Ihr soziales Netzwerk
Funktion	Ihren funktionellen Zustand
Besondere Bemerkungen	Einige besonders wichtige Punkte
Rauchen	Möglichkeiten zur Aufgabe des Rauchens
Alkoholgenuss	Alkohol im Alter
Körperliche Betätigung	Ihren Trainingszustand
Ernährung	Ihre Essgewohnheiten
Verhütung von Verletzungen	Sicherheit zu Hause und unterwegs
Ihr Hausarzt/Ihre Hausärztin	Themen zur Besprechung

C. Der zusammenfassende Bericht an den Hausarzt (Anhang IV)

Im zusammenfassenden Bericht an den Hausarzt sind alle klinisch relevanten Ergebnisse aus dem Gesundheitsprofil-Fragebogen auf zwei Seiten zusammengestellt. Alle relevanten Symptome oder Befunde sind darin aufgeführt und wenn auffällig, entsprechend markiert. Diese Zusammenfassung kann als Grundlage für weitere Massnahmen, z.B. diagnostische Abklärungen, Behandlung oder Überweisung verwendet werden. In diesem Zusammenhang muss allerdings noch einmal erwähnt werden, dass alle im zusammenfassenden Bericht aufgeführten Befunde auf der von der älteren Person selbst ausgefüllten schriftlichen Befragung basieren. Deshalb sollte jede Angabe aus dem Fragebogen vor einer klinischen Entscheidung auf Korrektheit überprüft werden.

Der Hausarzt und der Gesundheitsberater verwenden den zusammenfassenden Bericht als Grundlage für die individuelle Beratung bezüglich Prävention und Gesundheitsförderung.

2. BERATUNG UND INTERVENTION IN DER ALLGEMEINPRAXIS

A. Grundlagen

Der computergestütze Gesundheitsbericht ist die Grundlage der Intervention in der ärztlichen Praxis. Vom ausgedruckten Bericht allein ist allerdings nicht zu erwarten, dass er bei älteren Personen bereits zu einer Verhaltensänderung führt. Daher sind die individuelle Bekräftigung und das wiederholte Aufgreifen der Empfehlungen durch den Hausarzt oder die Gesundheitsberatung der Schlüssel zum Erfolg.

Regionale Faktoren und die Art und Weise, wie das HRA-O in das Gesundheitssytem integriert wird, beeinflussen die Durchführung der Prävention und Gesundheitsförderung wesentlich. Im folgenden sind einige allgemeine Grundprinzipien der Intervention zusammengefasst.

1. Die Intervention versucht, wünschenswerte Verhaltensänderungen bei älteren Personen zu bewirken. Hierbei basiert sie auf:

- dem Einsatz unterschiedlicher Methoden (persönlicher Gesundheitsbericht, persönliche Beratung, Einbezug von Hausarzt und Gesundheitsberatung)
- wiederholter Verlaufsbeobachtung
- Erziehung der älteren Person zur Eigenverantwortung
- der Theorie, dass eine Verhaltensänderung induziert werden kann, wenn die individuelle Motivationsstufe in der Beratungssituation berücksichtigt wird

2. Die Intervention versucht, das Engagement der Hausarztpraxen im Bereich Gesundheitsvorsorge und Gesundheitserziehung zu erhöhen. Dies soll mittels gezielter Unterstützung der beteiligten Ärzte mit folgenden unterschiedlichen Methoden erreicht werden:

  - dem Interventionsmanual
  - Weiterbildungsangeboten
  - schriftlicher oder persönlicher Rückmeldung an die beteiligten Ärzte
  - Beschaffung evidenzbasierter Fachliteratur

3. Jede Region erstellt, basierend auf lokalen Ressourcen, eine regionale Version des Interventionsprozesses. Folgende Faktoren sind hierbei zu berücksichtigen:

(I) Die gesamte Intervention sollte einer maximalen Zahl von Patienten und Ärzten zukommen

(II) Das Projekt ist möglichst optimal in das bestehende System der Grundversorgung zu integrieren

(III) Das an der Intervention beteiligte Personal soll so effizient wie möglich eingesetzt werden

(IV) Es ist ein definierter kontinuierlicher Kontakt zwischen am Projekt beteiligten Geriatern, Praxen, und dem Team der Gesundheitsberater sowie zwischen den beteiligten Praxen und ggf. zwischen einzelnen Mitgliedern der Praxen sicherzustellen

(V) Wesentliche Aspekte des Interventionsprozesses sind zur Qualitätssicherung und für wissenschaftliche Zwecke zu dokumentieren

(VI) Das System unterzieht sich regelmässigen Qualitätskontrollen

Zusatzinformatin Schweiz

Nach dem transtheoretischen Modell, das der Intervention zugrunde liegt, sind zu Beginn der Beratung folgende Stufen möglich:

1. Absichtslosigkeit
2. Absichtsbildung
3. Vorbereitung
4. Handlung
5. Aufrechterhaltung

Im Beratungsverlauf kann es zu einem Stuf<enwechsel in beide Richtungen kommen.

Weiterführende Literatur siehe Anhang: Zimmerman GL, Olsen CG, Bosworth MF. A 'stages of change' approach to helping patients change behavior. Am Fam Phys. 2000,1;61,1409-16

B. Die Rolle des Hausarztes

Der Hausarzt trägt zur Umsetzung der Intervention bei, indem er:

- im Rahmen der ohnehin stattfindenden Patientenkontakte systematisch zu einer positiven Verstärkung der Empfehlungen im Gesundheitsbericht und der ergänzenden Gesundheitsberatung beiträgt
- medizinischen Rat gibt, diagnostische Massnahmen ergreift und angemessene therapeutische Schritte ergreift Patienten bei entsprechender Indikation zu Spezialisten überweist

Die hausärztlichen Tätigkeiten im Rahmen des Projektes betreffen (ggf. in Zusammenarbeit mit dem medizinischen Studienkoordinator):

- Motivation zur Umsetzung der Empfehlungen, aus dem persönlichen Gesundheitsbericht
- Abgabe von Informationsmaterial/Literatur zur Reduktion des Gesundheitsrisikos
- Erleichterung von Kontakten zu projektrelevanten Organisationen (lokale Beratungsstellen, Selbsthilfeorganisationen, Freiwilligenverbände)
- Abgabe medizinischer und therapeutischer Empfehlungen nach dem aktuellen Stand wissenschaftlicher Erkenntnisse
- Berücksichtigung nationaler und internationaler Richtlinien bei Beratung und Therapie
- Therapie und Beratung unter Berücksichtigung der klinischen Situation
- Überweisung zum Spezialisten soweit erforderlich
- Wenngleich Hausarzt und Gesundheitsberater bereits wesentlich die Motivation der Patienten zur Umsetzung der Empfehlungen fördern sollen, muss die Initiative hierzu beim Patienten selbst liegen. Dennoch gehen wir davon aus, dass das HRA-O die Zahl verschiedener präventiver Interventionen erhöhen wird.

Trotz der in diesem Manual enthaltenen Empfehlungen bleibt es dem klinischen Ermessen des Hausarztes überlassen, welche Vorgehensweise am geeignetsten erscheint. Publizierte Leitlinien können im Laufe der Zeit überarbeitet oder ungültig werden. Das dem vorliegenden Manual zugrunde liegende Konzept besteht darin, derzeit anerkannte Standards umzusetzen, wo immer sie anwendbar sind. Im Rahemen des Fortbildungsangebot durch die Projektgeriate werden Bereiche angesprochen, bei denen neuere Erkenntnise zu Anpassungen von Empfehlungen führen.

C. Die Rolle der Gesundheitsberatung

Das Team der Gesundheitsberater unterstützt die Gesundheitserziehung und Gesundheitsvorsorge durch:

- enge Zusammenarbeit mit den beteiligten Ärzten und anderen Mitgliedern des Praxisteams
- regelmässigen, aktiven Kontakt mit den Teilnehmern, um die Empfehlungen des Gesundheitsberichts zu unterstützen und präventivmedizinische Massnahmen zu unterstützen und zu konkretisieren und damit die Aktivitäten der individuellen Gesundheitsförderung zu steigern

- Motivation der älteren Person in den Bereichen Gesundheitsverhalten und Prävention auf Basis des transtheoretischen Modells (s.o)
- ggf. rechtzeitige Kontaktaufnahme zum Hausarzt, wenn indiziert
- Förderung der Selbstbestimmung und Selbstverantwortung durch Begleitung bei der Lösung und Bewältigung von Problemen
- Hilfestellung beim Erarbeiten von Prioritäten und gezielter Besprechung der Risiken, Probleme und Ressourcen

3. EVIDENZ-BASIERTE EMPFEHLUNGEN ZUR PRÄVENTION UND GESUNDHEITSFÖRDERUNG IM ALTER

Die Empfehlungen zu jedem Themenbereich sind auf folgende Weise gegliedert:

1. **Einführung:** In diesem Teil werden die grundlegenden Überlegungen zum Inhalt der Empfehlungen des Gesundheitsberichts erörtert.
2. **Empfehlungen:** Kurze Empfehlungen für das weitere Vorgehen unter Bezug auf den zusammenfassenden Bericht an den Hausarzt.
3. **Zusatzinformation Schweiz:** Falls besondere Aspekte berücksichtigt werden müssen, sind diese regionalen Informationen in einem Abschnitt „Zusatzinformation Schweiz“ ausgeführt.
4. **Hintergrundinformation:** Dieser Abschnitt nennt den Empfehlungen zugrunde liegende relevante Publikationen und Studienergebnisse und gibt kurze, wenn möglich quantitative Informationen zu Nutzen und Risiken der Empfehlungen. Dieser Abschnitt listet die den Empfehlungen zugrunde liegenden relevanten Informationen (bspw. wichtige Publikationen und Quellen) und gibt kurze, wenn möglich quantitative Informationen zu Nutzen und Risiken der Empfehlungen. In den Fällen, in denen der Evidenzgrad für die Empfehlung klar ersichtlich ist, ist der entsprechende
5. **Evidenzgrad** Der Evidenzgrad ist mit A bis D (gemäss Klassifikation von evidenzbasierten Richtlinien, BMJ 1998;316:1303-1309) angegeben.

Evidenzgrad:

A: basiert auf einer Meta-Analyse von randomisierten kontrollierten Studien oder auf mindestens einer randomisierten kontrollierten Studie.
B: basiert auf mindestens einer nicht-randomisierten, kontrollierten Studie.
C: basiert auf einer deskriptiver Studie.
D: basiert auf Expertenkonsensus oder Expertenmeinung.

Referenzen: Dieser Abschnitt listet Guidelines, Studien und sonstige thematisch relevante Publikationen.

Anhang: Der Anhang beinhaltet beinhaltet Kopien von verwendeten Instrumenten und Kopien von bedinders wichtigen Artikeln.

A. Vorbeugung gegen Herz-Kreislauf Risiken

Hypertonie

Einführung

Es ist gut belegt, dass die Einstellung des Blutdrucks bei älteren Hypertonikern zu verminderten kardiovaskulären Komplikationen führt. Kontrolle und Einstellung sollten entsprechend den Richtlinien nationaler und internationaler (zum Beispiel der Europäischen Hypertonie Gesellschaft) Fachgesellschaften erfolgen. Diese Richtlinien unterstreichen ebenfalls die Wichtigkeit der Behandlung der isolierten systolischen Hypertonie. Allerdings gibt es Anhaltspunkte dafür, dass diese bestehenden Leitlinien in der Praxis nicht in ausreichendem Mass umgesetzt werden und folglich Diagnose und Behandlung der Hypertonie nicht optimal sind **(B)**.

Als Grundregel mag gelten: Mindestens eine Blutdruckmessung im Jahr für ältere Personen, die in der Anamnese keine Hypertonie aufweisen, und vierteljährliche Kontrollen bei Patienten mit medikamentös eingestellter Hypertonie. Bei ungenügend eingestellten Hypertonikern sollten Massnahmen ergriffen werden (nötigenfalls auch die Überweisung an einen Spezialisten), um eine bessere Einstellung zu erzielen, und die Blutdruckkontrollen sollten häufiger durchgeführt werden. Zwar ist die Evidenzlage für den Benefit einer Blutdruckeinstellung bei über Achtzigjährigen schwächer, doch sprechen die vorliegenden, wissenschaftlichen Daten für eine weitere gute Einstellung des Blutdrucks, auch bei hochbetagten Patienten, wenn keine Kontraindikationen aufgrund von Begleiterkrankungen vorliegen.

Empfehlungen

- Als Screening jährliche Blutdruckmessung
- Bei Blutdruck von 140-159 / 90-99 mm Hg: Monatliche Kontrolle über mindestens 4 Monate (danach einmal jährlich) wenn die Werte nicht über 160 mm Hg systolisch beziehungsweise 100 mm Hg diastolisch steigen. Bereits bei diesen Werten sollte aber schon behandelt werden, wenn ein manifester Diabetes besteht, kardiovaskuläre Komplikationen oder Organmanifestationen nachgewiesen sind oder das Risiko einer koronaren Herzkrankheit innerhalb der nächsten 10 Jahre >= 15% beträgt **(B)** (siehe Anhang: Coronary Risk Prediction Chart).
- Bei persistierendem syst. BD >=160 beziehungsweise diast. BD >= 100 mm Hg ist eine medikamentöse Behandlung indiziert **(A)**.
- Die optimalen Behandlungsziele für den Blutdruck sind ein systolischer Blutdruck von =<140 mm Hg und ein diastolischer Blutdruck von =< 85 mm Hg **(A)**. Für Patienten mit Diabetes wird ein niedrigeres Behandlungsziel von <140/80 empfohlen **(A)**.
- Zusätzlich sollten bei allen Personen mit Bluthochdruck oder mit einer Grenzwerthypertonie (>=130/85 mm Hg) nichtpharmakologische Massnahmen ergriffen werden. Folgende Massnahmen haben sich zur Blutdrucksenkung als wirksam erwiesen: Einschränkung der Natrium-Zufuhr **(A)**, reduzierte Fettzufuhr **(A)**, erhöhter Verzehr von Obst und Gemüse **(A)**, regelmässiges Ausdauertraining **(A)** und Reduktion des Alkoholkonsums **(A)**.
- Wenn keine Kontraindikationen (einschliesslich Diabetes) vorliegen, sind niedrigdosierte Thiazide als Kombinationspräparate(z.B. Comilorid®, Moduretic®) für ältere Leute mit erhöhtem Blutdruck das Mittel der ersten Wahl **(A)**.
- Als Primärprävention zur Herabsetzung des kardiovaskulären Risikos werden bei 50-jährigen und älteren hypertensiven Personen, bei denen bereits ein Endorganschaden,

ein Diabetes mellitus oder ein Risiko für eine koronare Herzkrankheit innerhalb der nächsten 10 Jahre von >=15% vorliegt, 75 bis 100 mg Aspirin pro Tag empfohlen **(A)**.

Zusatzinformation Schweiz

Folgende Empfehlungen zur Blutdruckmessung werden von der Schweizerischen Vereinigung gegen den hohen Blutdruck herausgegeben. Der Blutdruck sollte mit einer Armmanschette nach 3 Minuten Ruhe im Sitzen gemessen werden, idealerweise wird nach 3 Messungen der Mittelwert aus der 2. und 3. Messung festgehalten. Zusätzlich wird für ältere Patienten zum Ausschluss der orthostatischen Hypotonie eine BD Messung im Stehen empfohlen.

Hintergrundinformation

Hypertonie, einschliesslich der isolierten systolischen Hypertonie (>=160/<90 mm Hg), wird bei mehr als der Hälfte der Bevölkerung über 60 Jahre festgestellt. Der absolute Nutzen, den die Patienten aus der Behandlung der diastolischen und der isolierten systolischen Hypertonie ziehen, ist bei alten Menschen grösser als bei jüngeren Altersgruppen, insbesondere was kardiovaskuläre Komplikationen (einschliesslich Herzinsuffizienz) und vaskuläre Demenz angeht **(A)**.

Eine antihypertensive Behandlung ist mindestens bis zum Alter von 80 Jahren vorteilhaft **(A)**, und regelmässige Blutdruckkontrollen sollten bis zu diesem Alter fortgesetzt werden. Wenn mit einer Behandlung einmal begonnen wurde, sollte sie auch über das Alter von 80 Jahren hinaus weitergeführt werden. Aus der derzeitigen Fachliteratur lassen sich keine gesicherten Richtlinien ableiten, nach denen ein Hypertonie-Screening oder die Behandlung einer neu diagnostizierten Hypertonie bei Personen über 80 erfolgen sollte. Es besteht allerdings der allgemeine Konsens, dass auch für diese Altersgruppe im Hinblick auf hypertensive Komplikationen und Endorganschäden die Behandlung der Hypertonie (mit denselben optimalen Werten wie für jüngere Patienten) angebracht ist. Eine antihypertensive Behandlung (selbst bei systolischer Grenzwerthypertonie) sollte daher auch bei hypertonen Achtzigjährigen und älteren in Betracht gezogen werden, die keine Komplikationen aufweisen und sich eines guten Allgemeinzustands ohne wesentliche Einschränkung ihrer Lebenserwartung erfreuen **(D)**.

Niedrig dosierte Thiazide in Kombinationspräparaten(z.B. Comilorid®, Moduretic®) sind als Medikamente der ersten Wahl für ältere Menschen empfohlen und werden gut vertragen **(A)**. Betablocker sind als Medikamente erster Ordnung weniger wirksam als Thiazide, und Meta-Analysen lassen vermuten, dass sie in dieser Altersgruppe zwar die Zahl der Schlaganfälle, nicht aber anderer kardiovaskulärer Ereignisse verringern **(A)**. Dihydropyridin-Calciumantagonisten mit langer Wirkungsdauer (beispielsweise 5 mg Amlodipin) sind für ältere Patienten geeignete Alternativen, wenn Thiazide unwirksam, kontraindiziert oder unverträglich sind **(A)**. ACE-Hemmer können zwar bei der Behandlung einer mit Herzinsuffizienz oder linksventrikulärer Funktionseinschränkung einhergehenden Hypertonie nützlich sein, sie sollten aber bei älteren Hypertonikern mit peripheren Gefässerkrankungen (wegen potentieller renovaskulärer Mitbeteiligung) oder manifester Niereninsuffizienz mit Vorsicht verwendet werden.

Referenzen

Guidelines for Management of Hypertension: Report of the Third Working Party of the British Hypertension Society, 1999 (J Human Hypertension 1999;13:569-592 oder www.hyp.ac.uk/bhs)

Schweizerischen Vereinigung gegen den hohen Blutdruck, Arterielle Hypertonie, Empfehlungen für Ärzte, 1998

Hyperlipidämie

Einführung

Ältere Menschen sollten sich regelmässig alle 5 Jahre einer Kontrolle des Cholesterinspiegels unterziehen und sich gleichzeitig auf andere kardiovaskuläre Risikofaktoren untersuchen lassen. Zusätzlich zur Primärprävention verzögert die Verminderung des Cholesterins das Fortschreiten einer atherosklerotischen Herz-Kreislauf-Erkrankung (Sekundärprävention).

Empfehlungen

- Messung des Gesamtcholesterins alle 5 Jahre, mindestens bis zum 75. Lebensjahr, bei biologisch rüstigen Patienten auch darüber hinaus.
- Verwendung der Coronary-Risk-Prediction-Chart oder der Richtlinie der Arbeitsgruppe Lipide und Atherosklerose (siehe Anhang).
- Als Richtlinie für die Behandlung: Wenn das geschätzte Risiko eines kardiovaskulären Ereignisses innerhalb der nächsten 10 Jahre <30% (s. Coronary Risk Prediction Chart) ist, genügen Massnahmen zur Veränderung des Lebensstils und Kontrollen nach 5 Jahren.
- Geeignete Massnahmen zur Veränderung des Lebensstils sind: Reduktion der täglichen Zufuhr von Fett, Glucose und Salz **(A)** sowie erhöhte Zufuhr von verzweigten Kohlenhydraten, Obst und Gemüse **(A)**, körperliche Bewegung **(B)**, Gewichtsabnahme bei Adipösen **(B)**, Reduktion eines exzessiven Alkoholkonsums **(B)** und Aufgabe des Rauchens **(B)**.
- Wenn das Risiko für ein koronares Ereignis innerhalb der nächsten 10 Jahre >=30% beträgt und der Cholesterinspiegel im Serum unter 5 mmol/l liegt, sind Massnahmen zur Veränderung des Lebensstils zu ergreifen und deren Erfolg nach einem Jahr erneut zu überprüfen **(C)**.
- Wenn das Risiko für ein koronares Ereignis innerhalb der nächsten 10 Jahre >=30% beträgt und der Cholesterinspiegel im Serum bei >= 5 mmol/l liegt, erneute Kontrolle 3-6 Monate nach Massnahmen zur Veränderung des Lebensstils. Wenn das Gesamtcholesterin im Nüchternserum >= 5 mmol/l bleibt, ist zusätzlich zu nichtpharmakologischen Massnahmen **(A)** eine Statin-Behandlung indiziert [zum Beispiel Pravastatin (Selipran®, Mevalotin®) **(A)** oder Simvastatin (Zocor®) **(B)**]. Behandlungsziel: Gesamtcholesterin < 5 mmol/l **(B)**.
- Angesichts des Mangels an Daten für Personen mit einer Hyperlipidämie ab 75 Jahren wird heute mehrheitlich die Auffassung vertreten, dass eine bestehende Statin-Behandlung bei diesen Patienten fortgesetzt und eine Neubehandlung vom Zustand des einzelnen Patienten abhängig gemacht werden sollte **(D)**. Nichtpharmakologische Massnahmen sollten in angemessener Form jedoch bei allen älteren Patienten ergriffen werden.

Zusatzinformation Schweiz

Das im Projekt eingesetzte Cardiac Risk Prediction Chart weicht in seinen Empfehlungen nicht von denen der Arbeitsgruppe Lipide und Atherosklerose (AGLA) der Schweizerischen Gesellschaft für Kardiologie ab. Verglichen mit der Tabelle der AGLA verzichtet die englische Version auf die Bestimmung des LDL-Cholesterin und der Triglyceride, teilt Patienten detaillierter in Gruppen mit erhöhtem cardiovaskuläre Risiko ein und lässt eine genauere Risikoabschätzung in % zu.

Hintergrundinformation
Hypercholesterinämie und niedrige HDL-Werte (0.9 mmol/l) sind mindestens bis zum Alter von 75 Jahren Risikofaktoren für kardiovaskuläre Ereignisse.

Eine Statin-Behandlung zur Primär- und Sekundärprävention vermindert signifikant die Zahl gravierender koronarer Ereignisse (um 30%), die Gesamtmortalität sowie das Schlaganfall-Risiko bei Patienten mit koronarer Herzkrankheit **(A)**.

Referenzen

Scottish Intercollegiate Guidelines Network (SIGN). Lipids and the primary prevention of coronary heart disease 1999 (oder www.show.scot.nhs.uk/sign/guidelines/published/index.html).

Empfehlungen 1999 zur Behandlungsindikation des Risikofaktors Cholesterin, Schweizerische Aerztezeitung. 2000, 81:38, 2139-43

Diabetes mellitus

Einführung
Die geschätzte Prävalenz eines unerkannten Diabetes bei älteren Leuten von 5% und das häufig asymptomatische oder unspezifische Krankheitsbild in dieser Population sind Ausgangspunkt für Konsensus-Richtlinien, in denen ein Blutzucker-Screening alle drei Jahre empfohlen wird.

Empfehlungen
- Konsensus-Empfehlungen fordern bei über 45-Jährigen alle 3 Jahre ein Diabetes-Screening mittels Nüchtern-Blutzuckerbestimmung (NBZ) **(D)**.
- Jährliche Abklärungen werden bei älteren Patienten empfohlen, in deren Familienanamnese bei Verwandten ersten Grades Diabetes bekannt ist, die übergewichtig sind oder einen niedrigen HDL-Wert (0.9 mmol/l), hohe Nüchtern-Triglyceridwerte (> 2.8mmol/l) oder eine koronare Herzkrankheit aufweisen **(D)**.
- Für die NBZ-Bestimmung sollte der Patient mindestens 8 h nichts gegessen und lediglich Wasser getrunken haben. Ein Nüchtern-Blutzucker-Spiegel von >=7.0 mmol/l bei einer Bestimmung aus venösem Blut ist diagnostisch für das Vorliegen eines Typ II Diabetes. Bei einer kapillären Probe gilt ein Nüchtern-Blutzuckerspiegel von >=6.1 mmol/l als diagnostisch, er sollte aber mittels venöser Probe überprüft werden. Bei der Behandlung eines Diabetes gelten für gesunde Alte prinzipiell dieselben Blutzucker-Zielwerte (sowohl NBZ als auch HbA1c) wie für jüngere Menschen, um das Komplikationsrisiko herabzusetzen **(A)**. Bei älteren Patienten mit Begleiterkrankungen sollte jedoch das Behandlungsziel darin bestehen, Symptomen einer Hyperglykämie vorzubeugen und eine Hypoglykämie zu vermeiden **(D)**.
- Sulfonylharnstoffe sollten bei älteren Leuten mit Vorsicht verwendet werden; mit niedrigen Initialdosen beginnen und die Dosierung schrittweise erhöhen **(D)**. Glimepirid (Amaryl®) ist vorzuziehen, da es mit weniger Hypoglykämie-Episoden in Zusammenhang gebracht wurde als Glibenclamid (Daonil®, Euglucon®) **(A)**.
- Die Verwendung von fertigen Mischinsulinen reduziert Dosierungsfehler bei älteren Patienten auf ein Minimum **(B)**.

- Ein interdisziplinärer Teamansatz in der Behandlung des Diabetes mit spezieller Patientenschulung und Diätberatung durch Spezialisten führt nachweislich zu einer Verbesserung der Blutzucker-Einstellung älterer Patienten **(B)**.

Zusatzinformation Schweiz

Obwohl ältere Personen gelegentlich normale Nüchtern-Blutzuckerwerte aufweisen und deren HbA1C pathologisch erhöht ist, wird die alleinige Bestimmung des HbA1c zur Diagnose des Diabetes derzeit nicht empfohlen. Das HbA1C (Kosten derzeit ca, sFr. 30.-) dient zur Verlaufsbeurteilung. Die BZ Einstellung erfolgt nach der Leitlinie „Moderne Einstellung des Diabetes“, der Arbeitsgruppe der Schweizerischen Diabetesgesellschaft und der Schweizerischen Gesellschaft für Endokrinologie und Diabetologie. Das Ziel HbA1C sollte auch bei einer älteren Person zwischen 6% und 7% liegen.

Neuere orale Antidiabetika wie Thiazotolidindione (z.B. Rosiglitazon, Avandia®) oder Aminosäurederivate wie Nateglinid (Starlix ®) bieten Diabetikern über 65 Jahre interessante Therapie-Optionen. Die Langzeiteffekte solcher Substanzen sind für diese Patientenpopulation jedoch nicht ausreichend beschrieben und diese Substanzen wurden bisher noch nicht in den Therapieempfehlungen für ältere Diabetiker erwähnt.

Referenzen

Clinical Practice Guidelines für die Behandlung des Diabetes in Kanada (Canadian Med Assoc J 1998;159 (8Suppl): S1-S28

Position Paper Screening for Type II Diabetes, American Diabetes Association, Clinical Practice Recommendations 2000, Diabetes Care, Vol 24 Supplement 1, http://journal.diabetes.org/CareSup1Jan00.htm

Moderne Einstellung des Diabetes, Arbeitsgruppe der Schweizerischen Diabetesgesellschaft und der Schweizerischen Gesellschaft für Endokrinologie und Diabetologie, Schweizerische Aerztezeitung, 2000, 81,25.1403-1404

B. Impfungen

Grippeimpfung

Einführung
Jedes Jahr gibt es in Europa Tausende von Krankenhauseinweisungen und Todesfällen wegen Grippeinfektionen, überwiegend bei Menschen im Alter von über 65 Jahren. Systematische Übersichtsstudien aus Grossbritannien, den Vereinigten Staaten und Kanada sind zu dem Schluss gelangt, dass die Impfung bei älteren Menschen eine wirksame, sichere und kostengünstige Methode darstellt, um grippebedingte Todes- und Krankheitsfälle zu reduzieren.

Empfehlungen

- Eine Grippeimpfung sollte jährlich bei allen, die 65 Jahre und älter sind **(B)**, erfolgen sowie bei jüngeren Personen, die zusätzliche Risikofaktoren aufweisen (Erwachsene mit chronischen Atemwegs- oder Herz-Kreislauf-Erkrankungen, Diabetes mellitus, Niereninsuffizienz, Immunsuppression und bei Bewohnern von Langzeitpflegeeinrichtungen).
- Kontraindikationen sind bekannte Überempfindlichkeit oder Anaphylaxie gegenüber Hühnereiweiss sowie febrile Erkrankungen, bis die Symptome abgeklungen sind.

Zusatzinformation Schweiz
Aktuell gelten die Empfehlungen zur Grippeprävention, Stand August 2000, des BAG in Zusammenarbeit mit der Arbeitsgruppe Influenza und der Schweizerischen Kommission für Impffragen (BAG, Supplementum XIII, Empfehlungen zur Grippeprävention, 2000: 1-18). Bisher liegen noch keine epidemiologisch gesicherten Daten über die Effizienz der nasalen Influenzavaccine vor. Die nasale Applikation ist zudem mit höheren Kosten verbunden.

Hintergrundinformation
Die United States Preventive Services Taskforce (USPTF) empfiehlt eine jährliche Impfung **(B)** für alle Personen im Alter von >=65 **(B)** und andere Risikogruppen **(B)**. Die Ergebnisse von systematischen Übersichtsstudien in Europa und Nordamerika zeigen deutlich, dass eine jährliche Grippeimpfung bei älteren Leuten Atemwegserkrankungen, Pneumonien, Krankenhauseinweisungen und Todesfällen vorbeugt **(A)**. Die Impfung älterer Leute in Pflegeeinrichtungen reduziert jedes der genannten Risiken etwa um die Hälfte **(B)**. Klinische Prüfungen von Grippeimpfstoffen bei älteren Menschen haben ihre Unbedenklichkeit erwiesen, wobei blande lokale Nebenwirkungen (wie leichte Entzündungsreaktionen) bei weniger als 20% der Geimpften beobachtet wurden und keine unerwünschten systemischen Wirkungen auftraten.

Referenzen

Diguiseppi, C. BMJ 1996;313:1162 (Editorial) Why everyone over 65 deserves influenza vaccine

Gross PA, Hermogenes AW, Sacks HS, Lau J, Levandowski RA. The efficacy of influenza vaccine in elderly people: a meta-analysis and review of the literature. Ann Intern Med 1995;123:518-27

NHS Centre for Reviews and Dissemination. Influenza vaccination and older people. Effectiveness Matters 1996:2(1)

Govaert TM, Dinant GJ, Aretz K, Masurel N, Sprenger M, Knottnerus JA. Adverse reactions to influenza vaccine in elderly people: randomised double blind placebo controlled trial. BMJ 1993;307:988-90

Impfung gegen Pneumonie

Einführung
Die Pneumokokkenimpfung stellt eine wirksame Immunisierung gegen Streptococcus pneumoniae dar, mit dem Ziel die Morbidität und Mortalität bei alten Menschen, Pflegeheimbewohnern und chronisch Kranken zu reduzieren.

Empfehlungen
- Pneumovax Impfung für alle Patienten, über 65.
- Auffrischimpfung nach 10 Jahren.

Zusatzinformation Schweiz
In der Schweiz gibt es derzeit keine BAG-Empfehlung zur Pneumokokkenimpfung bei älteren Personen. Die Impfung wird für Patienten über 65 Jahre nicht von den Krankenkassen übernommen

Hintergrundinformation
Eine 1999 veröffentlichte Meta-Analyse über die klinische Effektivität der Impfung mit Pneumokokken Polysacharid Vaccine (Hutchinson et al.) zeigte, dass das Risiko an einer systemischen Pneumokokkeninfektion zu erkranken um 83% gesenkt werden kann, wenn die Vaccine den entsprechenden Serotyp beinhaltete. Das Risiko an einer systemischen Pneumokokkeninfektion überhaupt zu erkranken wurde durch die Impfung um 73% reduziert **(A)**. Die Impfung war bei älteren, institutionalisierten Menschen oder solchen mit chronischen Erkrankungen ebenfalls effektiv. In einer retrospektiven Studie bei Personen mit chronisch obstruktiver Pneumopathie war die Pneumokokkenimpfung (über einen Beobachtungszeitraum von 2 Jahren) mit weniger Hospitalisationen wegen Pneumonie, weniger Todesfällen und Kosteneinsparungen assoziiert **(B)**.

Die kürzlich im Internet veröffentlichte Meta-Analyse von Moore et al. stellt die obigen Empfehlungen in Frage. Eine kritische Durchsicht dieser Arbeit zeigt jedoch potentielle Schwachstellen: 1. alte Menschen wurden gemeinsam mit anderweitig immunsupprimierten Personen analysiert, 2. Studien, die nieder und höhervalente Vaccine einsetzten, wurden gepoolt. Diese beiden methodischen Fehler lassen Zweifel an der Schlussfolgerung der Autoren aufkommen. Während sich der Schutz der Pneumokokkenimpfung bei Personen über 65 Jahren weiterhin der kritischen Beurteilung stellen muss, basieren unsere Empfehlungen auf den besten derzeit verfügbaren, Daten.

Eine neuere, prospektive Studie aus Skandinavien, hat die die Effekte einer 23-valenten Pneumokokenvaccine und der Influenza Impfung auf eine grosse Kohorte (N=100242) Personen über 65 Jahre untersucht. Die Autoren kamen zu dem Schluss, dass die Inzidenz einer Hospitalistation für die Diagnosen: Pneumokokken Pneumonien (-36%), und invasive Pneumokokkenerkrankung (-57%) reduziert werden konnte. Die Gesamt-Mortalität war bei den Geimpften um 57% niedriger, als in der Kontrollgruppe.

Referenzen

Hutchison BG et al. Clinical effectiveness of pneumococcal vaccine. Meta-analysis. Can Fam Physician 1999, 45:2381-93

Nichol KL et al. The health and economic benefits associated with pneumococcal vaccination of elderly people with chronic lung disease. Arch Intern Med 1999,8;159:2437-42

Moore RA et al. Are the pneumococcal polysaccharide vaccines effective? Meta-analysis of the prospective trials. BMC Family Practice 2000;1:1,http://www.biomedcentral.com/1471-2458

Christenson B et al. Effects of a large scale intervention with influenza and 23-valent pneumococcal vaccined in adults aged 65 years or older: a prospective study. Lancet, 2001; 357:1008-11

C. Krebsvorsorge

Kolonkarzinom

Einführung
Die Aufforderung der Patienten, eventuelle Vorteile eines fäkalen okkulten Bluttests (FOBT) mit ihrem Arzt zu erörtern basiert auf der Evidenz, dass die frühzeitige Entdeckung eines Kolonkarzinoms die Wahrscheinlichkeit einer Heilung erhöht.

Empfehlungen

- Die Entscheidung, ob man einen FOBT vorschlagen soll, ist eine Ermessensfrage und hängt von den regionalen Krebsvorsorgeprogrammen ab. Es ist aber angebracht, die Vorteile der frühzeitigen Diagnose eines Kolonkarzinoms mit den Patienten zu besprechen, deren Allgemeinzustand einem eventuellen chirurgischen Eingriff nicht entgegenstände. Es ist angemessen, diesen Test Personen vorzuschlagen, die um Beratung bitten, selbst wenn sie keine entsprechenden Symptome aufweisen. In der Regel sollten eine Probe an drei aufeinander folgenden Tagen entnommen werden. Bei jeder positiven Probe sollte eine Kolonoskopie in Erwägung gezogen werden.
- Der FOBT sowie weitergehende Untersuchungen (einschliesslich Überweisung an einen Facharzt) sollten in jedem Fall bei Personen mit hohem Risiko (Kolonkarzinom bei Verwandten ersten Grades, Kolitis) oder bei symptomatischen Patienten (zum Beispiel Gewichtsverlust, Anämie) erwogen werden.

Hintergrundinformation
Die United States Preventive Services Taskforce (USPTF) empfiehlt, dass alle Personen ab 50 Jahren jährlich einem Screening mit dem FOBT unterzogen werden sollen **(B)**. Auf zehn Personen, deren Test auf okkultes Blut im Stuhl positiv ausfällt, wird bei einer ein Karzinom, bei drei ein Adenom >1 cm, bei einer ein kleineres Adenom festgestellt und bei fünf ein negativer Befund erhoben. In drei randomisierten Studien konnte gezeigt werden, dass ein alle zwei Jahre durchgeführtes Screening auf okkultes Blut im Stuhl in der Lage ist, die Mortalität bei Patienten im Alter zwischen 50 und 80 Jahren um bis zu 20% zu senken. Zwar sind 74% der Dickdarmkarzinome, die bei Patienten mit positivem FOBT festgestellt werden, distal lokalisiert und daher durch Rektosigmoidoskopie erreichbar, doch geht ein distaler Befund mit mehr als 10%igen Wahrscheinlichkeit mit einer weiteren signifikanten, proximal gelegenen Veränderung einher. Die totale Koloskopie wird daher als die Untersuchungsmethode der Wahl bei Patienten mit positivem FOBT angesehen **(B)**. Die vorliegenden Daten reichen nicht aus, um zu bestimmen, ob die Kombination des FOBT mit der Rektosigmoidoskopie als Screeningverfahren grössere Vorteile bietet als jede Untersuchungsmethode für sich.

Referenzen

Atkin W. Implementing screening for colorectal cancer BMJ 1999;319:1212-1213

Colon cancer screening (USPSTF Recommendation). J Am Geriatr Soc 2000;48:333-5

Leitlinie der Dt Gesellschaft für Verdauungs- und Stoffwechselkrankheiten / Arbeitsgemeinschaft f. Gastroenterologische Onkologie: Kolorektales Karzinom. AWMF-Leitlinien-Register Nr. 021/007, Erstellung 1/1999. http://www.uni-duesseldorf.de/WWW/AWMF/ll/iverd007.htm

Mammakarzinom

Einführung

Internationale Leitlininen empfehlen zwar bei Frauen im Alter von 50-69 Jahren alle ein bis zwei Jahre eine Mammographie, der Benefit einer solchen Screening-Strategie auf die Reduktion der Brustkrebsmortalität bei Frauen ab 70 Jahren ist jedoch offen.

Empfehlungen

- Ein Mammographie-Screening sollte bei Frauen im Alter von 50-69 Jahren alle 2 Jahre erfolgen, soweit dies im Rahmen der nationalen Gesundheitsvorsorgeprogramme durchführbar ist.
- Jährlich sollte eine klinische Untersuchung der Brust erfolgen.
- Alle Frauen sollten in einfachen Methoden zur Selbstuntersuchung der Brust angeleitet werden.

Zusatzinformation Schweiz

Seit Juni 1999 werden in der Schweiz Screeningprogramme von der obligatorischen Krankenversicherung übernommen, wenn die angewendete Methode den Empfehlungen der Kommission der Europäischen Union entspricht. Bisher haben die Kantone Wadt, Wallis und Genf Screeningkampagnen, die diese Kriterien erfüllen, eingeführt. Im Kanton Solothurn besteht damit noch kein Angebot, und Mammographien werden nur aufgrund klinischer Indikation (z.B. Abklärung eines Palpationsbefunds) von der Krankenkasse finanziert.

Zudem erwähnenswert ist auch die in der Aerztezeitung publizierte Kontroverse um den Benefit der Mammographie als Screeningverfahren . Eine kürzlich veröffentliche Meta-Analyse (Gøtzsche P et al), die den Nutzen der Mammographie erneut in Frage stellte, löste auch in der Schweiz eine Diskussion aus. Das Ergebnis der Diskussion muss zur Zeit noch abgewartet werden. Damit halten wir daran fest, dass eine Screeninguntersuchung alle 2 Jahre im Alter von 50 bis 69 Jahren angezeigt ist.

Hintergrundinformation

Die United States Preventive Services Taskforce (USPTF) und die Canadian Task Force on the Periodic Health Examination empfehlen für Frauen zwischen 50 und 69 alle 1-2 Jahre eine Mammographie in Kombination mit einer klinischen Brustuntersuchung (KBU) **(A)**. Es liegen keine klinischen Studien vor, in denen ein Screening (entweder durch Mammographie oder durch KBU) bei Frauen über 70 ausgewertet wurde. Es gibt Studien dafür, dass ein alle 2 Jahre durchgeführtes Mammographie-Screening bei Frauen zwischen 50 und 69 Jahren die Brustkrebsmortalität um 20-50% herabsetzt. Dagegen zeigte eine kürzlich durchgeführte Analyse der Ergebnisse des Mammographie-Screenings in Schweden über die letzten zehn Jahre keine wesentliche Verminderung der Mortalität im Zusammenhang mit Mammakarzinomen und eine erhebliche Quote falsch positiver Diagnosen. Mehrheitlich wird derzeit jedoch weiterhin die Meinung vertreten, dass Mammographie Screening-Programme Vorteile bieten.

Die Sensitivität der Mammographie nimmt zwar bei älteren Frauen nicht ab, doch eine Entscheidungsanalyse über den Nutzen des Screenings von Frauen im Alter ab 70 Jahren hat gezeigt, dass der Überlebensgewinn mit der Zunahme des Alters und der Begleiterkrankungen zurückgeht.

Die jährliche klinische Brustuntersuchung allein erhält zur Zeit eine **C**-Empfehlung – die Sensitivität der KBU variiert je nach der Erfahrung der untersuchenden Person und bewegt

sich bezüglich Knoten von 1 cm Durchmesser zwischen 65% für diplomierte Krankenschwestern und 87% für Allgemeinpraktiker.

Die Unterweisung zur Selbstuntersuchung der Brust erhält ebenfalls eine **C**-Empfehlung – die Sensitivität bei der Selbstuntersuchung der Brust liegt zwischen 26% und 40% und nimmt mit zunehmendem Alter der Patientinnen ab.

Referenzen

Nystrom L et al. Breast cancer screening with mammography; overview of Swedish randomised trials. Lancet 1993;341:973-978

Sjonell G et al. Mammographic screening does not reduce breast cancer. Lakartidningen 1999.24;96 :904-5, 908-13

Fletcher SW et al. Physicians' ability to detect lumps in silicon breast models. JAMA 1985;253:2224-2228

Gøtzsche P et al. Is screening for breast cancer with mammography justifiable? Lancet 2000; 355: 129-34

Zervixkarzinom

Einführung

Ein Zervixabstrich alle 3 Jahre als Screeninguntersuchung bei Frauen zwischen 30 und 70 reduziert nachgewiesenermaßen die Mortalität bei Zervixkarzinomen. Die Risikoreduktion durch das Screening ist bei jungen Frauen jedoch deutlicher als bei älteren.

Empfehlungen

- Bei Frauen im Alter ab 70 Jahren, bei denen drei vorangegangene Tests negativ ausgefallen sind und keine Symptome vorliegen, kann auf weitere Zervixabstriche verzichtet werden.
- Frauen über 65, die sich nie einer zytologischen Untersuchung der Zervix unterzogen haben oder bei denen der letzte Abstrich 3 Jahre oder länger zurückliegt, sollte diese Vorsorgeuntersuchung empfohlen werden.

Zusatzinformation Schweiz

Der Zervixabstrich ist in den Krebs-Früherkennungs-Richtlinien der Verordnungen über Leistungen der obligatorischen Krankenversicherung alle 3 Jahre eingeschlossen

Hintergrundinformation

Die Richtlinien der United States Preventive Services Taskforce (USPTF) sehen vor, dass alle Frauen, bei denen eine Zervix vorhanden ist und die sexuell aktiv sind oder waren, eine Screeninguntersuchung durchführen lassen sollten **(A).** Das Screeningintervall sollte bei 3 Jahren liegen **(B)**. Es gibt wenig Anhaltspunkte dafür, dass ein jährliches Screening zu besseren Ergebnissen führen würde. Die USPTF empfiehlt, die Abstriche nach Erreichen des 65. Lebensjahres einzustellen, wenn sie bei regelmässigen vorausgegangenen Screenings normal ausgefallen waren **(C)**.

Das relative Mortalitätsrisiko des Zervixkarzinoms ist für Frauen über 70 Jahre im Vergleich zu anderen Altersgruppen sehr gering. Im UK National Cervix Cancer Screening Programm für Frauen im Alter von 30-74 Jahren sank gesamthaft die Inzidenz des

invasiven Karzinomes, aber die Reduktion der Mortalität bei älteren Frauen (55 Jahre und älter) war weitgehend nicht durch das Screening beeinflusst.

Referenzen

Sasieni P. Effect of screening on cervical cancer mortality in England and Wales: analysis of trends with an age period cohort model. BMJ 1999;318:1244-1245

Quinn M et al. Effect of screening on incidence of and mortality from cancer of cervix in England: evaluation based on routinely collected statistics BMJ 1999;318:904

Prostatakarzinom

Hinweis: Im Gesundheitsprofil wird das Screening auf Prostatakarzinom wegen mangelndem Konsensus über die Notwendigkeit eines solchen Screenings nicht erwähnt. Die nachfolgende Stellungnahme dient als Grundlage für die Beratung, falls Männer danach fragen.

Die Früherkennung eines Prostatakarzinoms mittels Bestimmung des Prostata-spezifischen Antigens (PSA) gewinnt zunehmend Befürworter, kann aber noch nicht als evidenzbasierte Screeningstrategie eingestuft werden. Die Durchführung dieses Tests sollte bei Männern mit entsprechender Symptomatik in Betracht gezogen werden. Darüber hinaus sollten die Untersuchungen der jeweiligen nationalen Vorsorgeprogramme wahrgenommen werden.

D. Aufrechterhaltung der Gesundheit

Zahnpflege

Einführung

Zahnprobleme sind eine Ursache für Morbidität und Mangelernährung im fortgeschrittenen Alter. In zahlreichen Studien wurde nachgewiesen, dass viele ältere Menschen wegen Erkrankungen im Mundbereich Probleme beim Kauen, Schmerzen und Schwierigkeiten beim Essen sowie Probleme im gesellschaftlichen Kontakt haben.

Empfehlungen

- Es sollte möglichst jeder ältere Patient davon überzeugt werden, sich einer jährlichen zahnärztlichen Untersuchung zu unterziehen.
- Die Patienten sollten zügig den Zahnarzt aufzusuchen, wenn aufgrund des Gesundheitsfragebogens entsprechende Probleme identifiziert wurden.

Zusatzinformation Schweiz

Im Rahmen der computergestützen Auswertung des Fragebogens zum Gesundheitsprofil wird aufgrund der eigenen Angaben der Patienten der GOHAI (Geriatric Oral Health Assessment Index) Score, berechnet (Atkinson, Dolan) Dieser Score gibt weist auf modifizierbare Risikofaktoren in drei Bereichen der oralen Gesundheit hin , die einen direkten Einfluss auf die Funktion haben:

1. physische Funktion wie: essen sprechen, schlucken,
2. psychosoziale Funktion wie Unzufriedenheit, Missgunst, Sorgen, Aesthetik
3. Schmerzen, Unbehagen

Eine frühzeitge Erkennung solcher Probleme und die Unterstützung der Patienten bei der regelmässigen zahnmedizinischen Kontrolle vermeiden funktionelle Einbussen.

Hintergrundinformation

Parodontose und rezidivierende Karies sind bei älteren Leuten die vorherrschenden Ursachen für den Verlust von Zähnen; beiden Problemen kann vorgebeugt werden. Veränderungen der Mundschleimhaut aller Art treten im Alter mit bis zu 30% immer häufiger auf. Personen, die ein erhöhtes Risiko für Erkrankungen im Mundbereich aufweisen, sind Diabetiker, Raucher und Patienten, die Medikamente einnehmen, die als Nebenwirkung zu einer Verminderung des Speichelflusses führen (zum Beispiel bestimmte Antidepressiva, Antihypertensiva und Diuretika).

Referenzen

McGrath C et al. Factors influencing older people's self reported use of dental services in the UK. Gerodontology 1999 Dec;16(2):97-102

Mandel ID. Preventive dental services for the elderly. Dental Clin N America 1989;33:81-90

Locker D et al. Self-perceived oral health status, psychological well-being, and life satisfaction in an older adult population. J Dent Res 2000 Apr;79(4):970-5

Nitschke I. Zahnmedizinische Grundlagen zur geriatrischen Rehabilitation – eine Einführung in die Alternszahnmedizin. Z Gerontol Geriat 2000; 33: Suppl. 1: I/45-I/49

Atkinson KA, Dolan T. Development of the Geriatric Oral Health Assessment Index. J Dent Educ 1990;54:680-7

Augenuntersuchungen

Einführung

Es gibt keine evidenzbasierten Indikationen für routinemässige ärztliche Augenuntersuchungen bei allen älteren Menschen. Dennoch ist im Gesundheitsfragebogen ein Screening Test des Sehvermögens enthalten (adaptierte Fragen aus dem VFQ25).

Empfehlungen

- Es sollte jedem älteren Patienten empfohlen werden, sich jährlich einer Augenuntersuchung inklusive Messung des Augeninnendrucks zu unterziehen. Da auch bei normalem Augendruck ein Glaukom vorliegen kann, sollte zumindest die erste Augendruckmessung im Rahmen einer ophthalmologischen Kontrolle erfolgen (mit Inspektion des Fundus). Die alleinige Messung des Augendruckes ist nicht ausreichend zur Beurteilung des frühen primären chronischen Offenwinkelglaukoms.
- Gewisse Umstände, wie das Vorliegen eines Diabetes oder der regelmässige Einsatz von Kortikosteroiden (auch nicht-systemische), machen eine regelmässige Augenkontrolle noch wichtiger.
- Augensymptome von potentiell erheblicher klinischer Relevanz (insbesondere eine plötzliche Visusverschlechterung) erfordern eine entsprechende ärztliche Untersuchung und ggf. die Überweisung an den Facharzt.

Zusatzinformation Schweiz

Im zusammenfassenden Bericht wird darauf hingewiesen, wenn möglicherweise aufgrund der Screeningbefragung Probleme in bestimmten Sehfunktionen vorliegen. Solche Symptome können auf eine Visusbeeinträchtigung, einen Katarakt, und eine Gesichtsfeldeinschränkung hinweisen.

Wenn aus mangelnder Infrastruktur eine jährliche Kontrolle mit Messung des Augendrucks nicht durchführbar ist, kann die Kontrolle alle 2 Jahre z.B. im Rahmen der Fahrtauglichkeitsuntersuchung durchgeführt werden.

Hintergrundinformation

Es gibt keine gesicherte Evidenz über den Nutzen eines Sehscreenings bei älteren Menschen, die keine visuellen Störungen angeben; ein gewisser Vorteil einer solchen Massnahme ist allerdings wahrscheinlich.

Es gibt Belege dafür, dass Störungen des Sehvermögens häufig bei älteren Menschen vorliegen, die zu Stürzen neigen.

Referenzen

Reidy A et al. Prevalence of serious eye disease and visual impairment in a north London population: population based, cross sectional study BMJ 1998;316:1643-1646

Smeeth L, Iliffe S. Effectiveness of screening older people for impaired vision in community setting: systematic review of evidence from randomised controlled trials BMJ 1998;316:660-663

Rott C, Wahl HW, Tesch-Römer C. Die Rolle von Seh- und Höreinbußen für den Alternsprozess. 1996 in: Tesch-Römer C, Wahl HW. Seh- und Höreinbussen älterer Menschen: Herausforderungen in Medizin, Psychologie und Rehabilitation. Steinkopff. Darmstadt; 89-106

Spannus K, Ritzmann P. Medikamentöse Glaukombehandlung. pharma-kritik 2000; 22:57-60

Prüfung des Hörvermögens

Einführung
Die häufigste Ursache für eine Beeinträchtigung des Hörvermögens im fortgeschrittenen Alter ist die Altersschwerhörigkeit, eine Wahrnehmungsminderung für hohe Frequenzen. Hörstörungen werden bei 23% der Menschen im Alter von 65-74 Jahren, bei 33% derer zwischen 75 und 84 Jahren und bei 48% der 85-jährigen und Älteren festgestellt. Viele ältere Menschen sind sich Ihres Hörverlustes nicht bewusst.

Empfehlungen

- Screening des Hörvermögens bei Personen ab 65 Jahren durch den Flüstertest (in einer Entfernung von 15-30 cm vom Ohr drei Zahlen flüstern, die vom Patienten wiederholt werden sollen, während man das andere Ohr mit dem Finger zuhält. Das Testergebnis ist positiv, wenn eine Zahl nicht verstanden wird) **(D)**.
- Wenn eine Hörminderung vorliegt, otoskopische Kontrolle um eine behandelbare Otitis auszuschliessen oder Cerumen zu entfernen.
- Überweisung an den Facharzt zur Audiometrie, wenn der Hörverlust die Kommunikation, die Sozialisation, die Selbständigkeit, die Stimmung oder die Lebensqualität beeinträchtigt.
- Den Patienten sollte vom Kauf teurer Hörgeräte abgeraten werden, bevor eine fachärztliche Abklärung und ggf. ein kostenloser Versuch mit dem Gerät erfolgt ist.

Zusatzinformation Schweiz
In der Schweiz erfolgt die Hörgeräteanpassung von Hörgeräteakustikern. Die Kosten für die Anpassung und das Gerät werden auf Antrag von der AHV übernommen. Hierfür muss auf der Anmeldung für Hilfsmittel-Leistungen der AHV nachgwiesen werden, dass der Patient bei einem von der AHV anerkannten Spezialarzt zur Abklärung überwiesen wurde. Ein Hörgerät kann bis zur Dauer von einem Jahr zur Probe getragen werden, der Patient trägt lediglich die anfallenden Materialkosten. Die Kosten für Batterien können in manchen Fällen über Ergänzungsleitungen abgegolten werden.

Hintergrundinformation
Wenngleich viele ältere Menschen mit Hörminderungen dieses Symptom ihrem Hausarzt gar nicht mitteilen, bleibt auch der Effekt eines Screenings auf Hörstörungen in der medizinischen Primärversorgung hinter den Erwartungen zurück, selbst wenn dies im Rahmen strukturierter Vorsorgeprogramme vorgesehen ist. Nur 8% der Leute über 65 mit Hörstörungen verwenden ein Hörgerät, und ein Fünftel der Hörgeräteträger über 85 melden Hörstörungen trotz einer entsprechenden Verstärkung. Eine Hörminderung im fortgeschrittenen Alter kann die Selbsthilfefähigkeiten im Alltag (zum Beispiel Einkaufen, Telefonieren oder Autofahren) beeinträchtigen und zu gesellschaftlichem Rückzug und Depression führen. Es gibt einige Belege dafür, dass eine Verbesserung des Hörens bei

älteren Menschen mit kognitiven Störungen zu einer Verbesserung ihrer kognitiven Fähigkeiten führen kann.

Referenzen

Freedman A et al. Preventive care for the elderly. Do family physicians comply with recommendations of the Canadian Task Force on Preventive Health Care? Can Fam Physician 2000;46:350-7

Bess FH et al. Hearing impairment as a determinant of function in the elderly. J Am Geriatr Soc 1989;37:123-8

Gennis V et al. Hearing and cognition in the elderly: new findings and a review of the literature. Arch Intern Med 1991;151:2259-2264

Rott C, Wahl HW, Tesch-Römer C. Die Rolle von Seh- und Höreinbussen für den Alternsprozess. 1996 in: Tesch-Römer C, Wahl HW. Seh- und Höreinbussen älterer Menschen: Herausforderungen in Medizin, Psychologie und Rehabilitation. Steinkopff. Darmstadt; 89-106

Körperliche Bewegung

Einführung

Körperliche Aktivität ist für ältere Menschen vorteilhaft, und zwar sowohl in der Primär- als auch in der Sekundärprävention kardiovaskulärer Erkrankungen und Todesfälle sowie für die Verhütung von Stürzen und Invalidität bei gebrechlichen Personen.

Empfehlungen

- Bei Personen, die körperlich nicht aktiv aber sonst gesund sind, sollten die im Gesundheitsbericht abgegebenen Empfehlungen hinsichtlich körperlicher Aktivität unterstützt werden.
- Wenn keine Kontraindikationen bestehen, sollte ein regelmässiges Ausdauertraining empfohlen werden, um die kardiopulmonale Leistungsfähigkeit und die allgemeine Belastbarkeit zu erhöhen **(A)**, während ein Widerstands-/Krafttraining geraten erscheint, um das Gleichgewicht und die Muskelkraft zu verbessern und das Sturzrisiko zu reduzieren **(A)**.
- Regelmässige Übungsprogramme für ältere Personen können sich besonders vorteilhaft auf Schmerzen und Behinderungen bei Gonarthrose **(B)**, auf die Gewichtsreduktion bei Übergewichtigen **(A)** sowie die Einstellung einer Hyperlipidämie **(A)** und Hypertonie **(A)** auswirken.
- Patienten, die körperlich nicht aktiv sind, erhalten schriftliche Ratschläge für ihr Training, es wird ihnen aber auch empfohlen, diese mit ihrem Hausarzt zu besprechen, insbesondere wenn sie vorhaben, ihre körperliche Aktivität wesentlich zu steigern. Ausserdem werden sie durch das Beratungsteam hierin unterstützt und ergänzend beraten.
- Patienten, die mit einem neuen Trainingsprogramm beginnen, sollten sich beim Hausarzt vorstellen, wenn sich bei ihnen neue oder zunehmende trainingsbedingte Beschwerden einstellen wie z.B. Atemnot oder Gelenkschmerzen.

Hintergrundinformation
Es gibt sichere Belege dafür, dass sich körperliche Betätigung bei älteren Menschen sowohl in der Primär- als auch in der Sekundärprävention kardialer Erkrankungen und Todesfälle günstig auswirkt. Der Nutzen körperlichen Trainings für die Prävention der koronaren Herzkrankheit bei Männern hat zu einer Empfehlung der US Preventive Service Task Force der Kategorie **A** geführt.

Sowohl regelmässiges Ausdauertraining (eine Aktivität, die über längere Zeit zu einer erhöhten Herzfrequenz führt) als auch Widerstands-/Krafttraining (bei dem die Muskelkraft durch eine stufenweise Erhöhung des Widerstandes trainiert wird,) verbessern nachweislich die Lebenserwartung und mindern die allgemeine Schwäche im Alter.

Ein Trainingsprogramm zur Verbesserung von Kraft und Gleichgewicht, das von Physiotherapeuten, im Rahmen von vier Hausbesuchen Frauen ab 80 Jahren angeboten wurde, vermochte nach einer Studie im Folgejahr die Häufigkeit von Stürzen und mittelschweren Verletzungen zu verringern, wobei diejenigen Frauen, die häufig gestürzt waren, am meisten profitierten.

Referenzen
Christmas C, Andersen RA. Exercise and older patients: Guidelines for the Clinician. J Am Geriatr Soc 2000;48:318-324

Meusel H. Grundlagen der Bewegungstherapie in der geriatrischen Rehabilitation. Z Gerontol Geriat 2000; 33: Suppl.1:I/35-I/44

Ernährung

Einführung
Eine schlechte Ernährung kann bei älteren Menschen zu Übergewicht (hiervon sind 16% der Erwachsenen in den USA betroffen) oder zu Unterernährung bzw. Eiweissmangelernährung (häufig multifaktorieller Genese) führen. Zu bemerken ist, dass Adipositas eine Eiweissmangelernährung nicht ausschliesst.

Zur Erfassung einer Fehlernährung gehören unter anderem:

Anamnese, Gewichtsverlauf, Appetit, Nahrungsgewohnheiten, Schluckbeschwerden, Zahnstatus

Körperliche Untersuchung, Grösse, Gewicht, BMI

ggf. Laboruntersuchungen (Albumin, Eisen, Transferrin, Lymphocytenzahl)

Empfehlungen
- Der Arzt und die Gesundheitsberater sollten sich bemühen, die Umsetzung der im Gesundheitsbericht detailliert dargelegten Empfehlungen zu unterstützen.
- Zu den grundlegenden diätetischen Ratschlägen gehört es, fünf oder mehr Portionen Obst und Gemüse pro Tag zu verzehren und weniger als 30% der täglichen Kalorien in Form von Fett zu sich zu nehmen (z.B. indem man Fisch und Geflügel als Ersatz für anderes Fleisch und Käse einsetzt oder fettarme Milch trinkt und fettarme Margarine statt Butter verwendet). Zusätzlich wird die Einnahme von Ballaststoffen empfohlen.
- Beim Vorliegen einer Eiweissmangelernährung sollte neben der Suche nach der Ursache versucht werden, die Proteineinnahme zu erhöhen.

- Im Einzelfall bedarf es u.U. der Überweisung zu einer speziellen Ernährungsberatung (beispielsweise bei Hyperlipidämie, Diabetes oder Übergewicht).

Zusatzinformation Schweiz

Der der Ernährung zugrunde liegende Fragebogen und die Beispiele bzw. Alternativangebote im Gesundheitsbericht legen ihren Schwerpunkt vor allem auf die reduzierte Zufuhr fetthaltiger und die erhöhte Zufuhr ballaststoffreicher Nahrungsmittel (s. Anhang III). Im persönlichen Gesundheitsbericht werden zudem detaillierte Hinweise auf die ausreichende Zufuhr von Calcium (1000-1500 mg/d) und die Möglichkeit der Vitamin-D Substitution (600-800 U/d) gegeben.

Hintergrundinformation

Eine gesunde Kost entsprechend den Empfehlungen der WHO, über einen Zeitraum von 20 Jahren korrelierte bei Männern zwischen 50 und 70 Jahren in Westeuropa mit einer Herabsetzung der Gesamtmortalität um 13%. Diätetische Massnahmen haben sich als wirksam in den Behandlungskonzepten der Hyperlipidämie, der Hypertonie und kardialer Erkrankungen erwiesen **(A)**. Es gibt jedoch Belege dafür, dass Hausärzte zwar den Wert einer Ernährungsberatung bei älteren Leuten anerkennen, der praktischen Umsetzung allerdings vielfache Hindernisse (wie beispielsweise Zeitmangel, mangelnde Mitarbeit seitens der Patienten, unzulängliches Lehrmaterial und Mangel an spezifischem Wissen) entgegenstehen.

Eine akute Erkrankung, insbesondere wenn sie zur Hospitalisierung führt, beschleunigt den Stickstoffverlust und die Eiweissmangelernährung bei älteren Menschen. Weitere modifizierbare Ursachen für eine Unterernährung bzw. Eiweissmangelernährung bei älteren Menschen sind Depressionen, Medikationen, kardiopulmonale Kachexie, Malabsorptions-Syndrome, chronische Verstopfung und soziale Faktoren (Immobilität oder Isolierung, die zu einem eingeschränkten Zugang zu Nahrungsmitteln führen oder Armut).

Referenzen

National Diet and Nutrition Survey of people aged 65 and over. Department of Health Food Safety Information Bulletin 1998;103 (Dec)

Kushner RF. Barriers to providing nutrition counselling by physicians: a survey of primary care practitioners. Prev Med 1995;24:546-52

Huijbregts P et al. Dietary pattern and 20 year mortality in elderly men in Finland, Italy, and the Netherlands: longitudinal cohort study. BMJ 1997;315:13-17

Lauque S, Nourhashemi F, Vellas B. Testwerkzeuge für die Ernährungsanamnese bei älteren Personen. Z Gerontol Geriat 1999; 32:Suppl.1, I/45-I/54

Millen BE. Präventive Ernährungsdienste für ältere Populationen. Z Gerontol Geriat 1999; 32:Suppl.1, I/80-I/88

Funktionelle Fähigkeiten und Unabhängigkeit

Einführung

Dieser Bereich umfasst:

- Aktivitäten des täglichen Lebens (z.B. Transfer, Körperpflege, Ankleiden, Beherrschung der Blase und des Stuhlgangs, Mobilität, Essen)
- Instrumentelle Aktivitäten des täglichen Lebens (z.B. telefonieren, einkaufen, Wäsche waschen, Transport, einen Haushalt führen, der Umgang mit Geld und Medikamenten)
- Funktionseinschränkungen bestehen dann, wenn Teilnehmer in einem oder mehreren Bereichen hilfsbedürftig werden

Ein frühzeitiges interdisziplinäres Vorgehen bei der Therapie von Funktionseinschränkungen älterer Menschen verbessert sowohl die funktionellen als auch die psychosozialen Ergebnisse **(B)**. Die Forschung hat in der letzten Zeit ihr Interesse auf die Prävention funktioneller Einbussen gerichtet, und versucht, Prädiktoren der im Alter auftretenden Funktionsbeeinträchtigungen (insbesondere der sogenannten präklinischen Mobilitätsbehinderung) zu bestimmen. Der Gesundheitsfragebogen hat zum Ziel, solche Indikatoren zu identifizieren.

Empfehlungen

- Der Gesundheitsbericht fordert die Patienten auf, ärztlichen Rat zu suchen, wenn sie unter funktionellen Einschränkungen leiden, insbesondere wenn diese neu aufgetreten sind. Die Patienten erhalten praktische Ratschläge um ihre Mobilität sowie ihre häuslichen und sozialen Aktivitäten aufrecht zu erhalten. Diese bedürfen der Bekräftigung und Ergänzung.
- Eine ärztliche Abklärung ist angebracht, wenn sich ein Nachlassen bestimmter Funktionen plötzlich oder ohne erklärbare Ursache ergibt.
- Der Fragebogen erfasst Patienten mit präklinischen Hinweisen auf Funktionsbeeinträchtigungen, die bspw. nicht direkt über eine eingeschränkte Mobilität berichten, jedoch mitteilen, dass sie die Art und Weise ändern mussten, in der sie gewisse Aufgaben bewältigen (z.B. das Ein- und Aussteigen aus dem Auto, das Treppensteigen usw.). Diese Patienten sollten auf chronische Belastungen untersucht werden, die zu einem Fortschreiten der Funktionseinschränkungen beitragen könnten. Ein umfassendes, interdisziplinäres geriatrisches Assessment (medizinisch, pflegerisch, physiotherapeutisch und ergotherapeutisch) ist für Patienten indiziert, die über wesentliche, neu aufgetretene funktionelle Beeinträchtigungen berichten, z.B. Hilfsbedarf bei mehr als einer Basisaktivität des täglichen Lebens.
- Chronisch Behinderte sollten Zugang zu Gesundheits- und Beratungsdiensten erhalten entweder durch Information über und/oder durch direkte Weitervermittlung an die relevanten öffentlichen oder freien Einrichtungen, die sich mit den Problemen Behinderter und deren Beratung und Unterstützung befassen.

Hintergrundinformation

Eine kanadische Screening-Studie zur Aufdeckung verschiedener Funktionsbeeinträchtigungen bei 65-jährigen und älteren Menschen gefolgt von entsprechenden Vermittlungen (z.B. zur Physiotherapie), zeigte eine Verbesserung in den Alltagsaktivitäten, in den mentalen Scores und in den sozialen Kontakten vor allem bei über 75-jährigen, allein oder zurückgezogen lebenden Menschen. Für Personen, die diese

soziodemographischen Faktoren nicht aufwiesen, war die Intervention insgesamt weniger effektiv.

In einer prospektiven Studie mit Frauen zwischen 70 und 80 Jahren ohne Mobilitätseinschränkungen wurde deren eigene Darstellung, in welcher Art und Weise sie gewisse Aktivitäten verrichteten, als ein guter Indikator für eine 18 Monate später bestehende Mobilitätsbehinderung identifiziert. Diese Indikatoren sind im Gesundheitsfragebogen enthalten und bieten eine gesicherte Grundlage für das Screening auf ein Behinderungsrisiko und das Einleiten gezielter präventiver Massnahmen .

Referenzen

Hay WI et al. Prospective care of elderly patients in family practice. Is screening effective? Can Fam Physician 1998;44:2677-87

Fried LA et al. Preclinical mobility disability predicts incident mobility disability in older women. J Gerontology 2000;55A:M43-M52

E. Besondere medizinische Gesundheitsprobleme

Schmerzen

Einführung

Chronische Schmerzen sind eines der Hauptprobleme bei zuhause lebenden älteren Menschen und führen zu häufigen Besuchen beim Hausarzt. Chronische Schmerzen im Alter können zu einem Teufelskreis aus Depression, sozialer Isolierung, Schlafstörungen und verminderter Mobilität führen. Die unzureichende Schmerzbehandlung in dieser Altersgruppe ist ein wesentlicher Faktor für die Entstehung einer medizinischen Unterversorgung. Umgekehrt kann ein unzureichend erhobenes Schmerzassessment zur unangemessenen Verschreibung von Schmerzmitteln bspw. nichtsteroidalen Antirheumatika (NSAR) oder Opiaten führen.

Empfehlungen

- Eine routinemässige Schmerzanamnese sollte Bestandteil jedes neuen ärztlichen, pflegerischen oder therapeutischen Kontakts mit älteren Patienten sein.
- Jede Angabe von Schmerzen, die mit körperlichen oder psychosozialen Einschränkungen einhergehen, muss als ernsthaftes Problem eingestuft werden. Aus diesem Grund ist oft eine frühzeitige medikamentöse Behandlung der Schmerzsymptomatik erforderlich.
- Schmerzen bei älteren Menschen sollten ausnahmslos einer sorgfältigen diagnostischen Abklärung zugeführt werden, die eine eingehende Anamnese, die ärztliche Untersuchung sowie ggf. indizierte weitergehende Untersuchungen (beispielsweise auf Osteoporose) umfasst (siehe Kommentare unter Abschnitt Stürze). Am wirksamsten lassen sich Schmerzen unter Kontrolle bringen, wenn ihre zugrundeliegenden Ursachen erkannt und behandelt sowie ihre funktionellen Auswirkungen durch ein sorgfältiges interdisziplinäres Assessment identifiziert werden. Falls die Ursache zweifelhaft bleibt, sollte ggf. eine fachärztliche Überweisung erfolgen.
- Einfache Selbstbeurteilungsskalen sollten verwendet werden, um die Schmerzen zu quantifizieren (zum Beispiel eine verbale oder visuelle Analogskala: „Wo liegen auf einer Skala von null bis zehn, auf der Null gar keine Schmerzen bedeutet und 10 die schlimmsten Schmerzen, die Sie sich vorstellen können“) (**D**)

- Patienten, die laut zusammenfassendem Bericht öfter als dreimal wöchentlich Analgetika einnehmen, sollten regelmässig kontrolliert werden .
- Paracetamol ist für die meisten älteren Menschen mit leichten bis mittelstarken Schmerzen das Medikament der Wahl, insbesondere bei arthrotischer **(A)** oder muskuloskeletaler (**D**) Genese. Es sollte – wie jede Schmerzmedikation bei chronischen Schmerzen – in fester und regelmässiger Dosierung verordnet werden und kann vorübergehend täglich bis auf 4 x 1000 mg gesteigert werden.
- Die Verwendung von NSAR bei älteren Patienten sollte auf die Behandlung akuter Entzündungszustände oder Knochenschmerzen beschränkt werden, sofern diesbezüglich keine Kontraindikationen vorliegen (Niereninsuffizienz **(A)**, gastrointestinale Erkrankungen **(A)**, dekompensierte Herzinsuffizienz). Eine weitere Indikation für NSAR liegt vor, wenn andere Analgetika-Strategien fehlgeschlagen sind. NSAR sollten bei älteren Menschen nur über einen begrenzten Zeitraum verwendet werden.
- Eine mit selektiven Cyclooxygenase-2-Hemmern verbesserte gastrointestinale Verträglichkeit bei älteren Menschen ist nicht nachgewiesen, so dass bei dieser neuen Medikamentenklasse die gleiche Zurückhaltung angezeigt ist wie bei den herkömmlichen NSAR.
- Eines der Hauptprobleme älterer Patienten unter Opiat-Analgetika aller Art ist die Obstipation **(C).** Mit jeder Opiat-Behandlung sollten daher frühzeitig stuhlregulierende Massnahmen verbunden werden.

Hintergrundinformation

Chronische Schmerzen sind ein wichtiges Problem in der Bevölkerung, 46,5% der Patienten im hausärztlichen Bereich klagen über chronische Schmerzen. Es besteht eine signifikante Korrelation zwischen fortschreitendem Alter und der Angabe von Schmerzen durch die Patienten. Rückenschmerzen und Arthrosen sind für ein Drittel aller Klagen verantwortlich. Trotz dieser hohen Prävalenz wurden Menschen ab dem 65. Lebensjahr bisher systematisch von klinischen Studien zur Schmerzbehandlung ausgeschlossen, so dass die derzeitigen Richtlinien für ältere Menschen weitgehend auf einem Meinungskonsens beruhen. Die Verwendung von Fragebögen und Skalen für die hausärztliche Schmerzerhebung führt nachweislich zu einer grösseren Zufriedenheit in der Verständigung zwischen Arzt und Patient wenn es um beklagte Schmerzen geht, und zwar unabhängig von deren Art und Ursache.

Referenzen

Elliot AM et al. The epidemiology of chronic pain in the community. Lancet 1999;354:1248-52

Radecki SE et al. Randomised clinical trial of diagnostic instrument for pain complaints. Fam Med 1999;31:713-721

Interdisziplinärer Arbeitskreis Schmerz im Alter (Hrsg). Schmerz im Alter. Ein Kompendium für Hausärzte. 1999; Lukon; Puchheim

Verwendung von Medikamenten

Einführung

Im Gesundheitsbericht werden die Patienten aufgefordert, alle Fragen zu ihrer Medikation mit ihrem Hausarzt zu besprechen, insbesondere wenn sie ihre Medikamente möglicherweise als überflüssig erachten, Fragen zu Kosten bzw. Kostenübernahme haben oder wenn sie sich hinsichtlich Nebenwirkungen oder Wechselwirkungen Sorgen machen. Um die Compliance in der Medikamenteneinnahme zu fördern, erhalten Patienten, die drei oder mehr Medikamente einnehmen die Empfehlung, über ihre Medikamenteneinnahme Buch zu führen. Dies vereinfacht Überprüfungen und erleichtert die Beratung durch den Hausarzt oder weitere Beteiligte hinsichtlich der richtigen Medikamenteneinnahme.

In der automatisierten Auswertung des Gesundheitsfragebogens werden Informationen über medizinische Symptome mit der angegebenen Medikation verglichen. Wo möglicherweise Medikamentennebenwirkungen bestehen, werden die Patienten aufgefordert, dies mit ihrem Arzt zu besprechen.

Ebenso könnte auf mögliche Unterlassungen einer etablierten Standardmedikation bei bestimmten Indikationen (zum Beispiel Thromboembolieprophylaxe bei Vorhofflimmern) aufmerksam gemacht werden.

Zudem beinhaltet der Fragebogen einzelne Fragen zu im Alter ungeeigneten Medikamenten. Aufgrund der Konsensuskriterien von Beers und von Prävalenzdaten sind langwirksame Benzodiazepine und trizyklische Antidepressiva mit deutlichen anticholinergen Nebenwirkungen die am häufigsten verwendeten Kategorien, die im Alter (von wenigen Ausnahmen abgesehen) nicht mehr verwendet werden sollten, und für die es sichere Alternativen gibt.

Empfehlungen

- Die Medikation sollte in ihrer Komplexität und Anzahl soweit wie möglich reduziert werden. (Hierzu gehört auch das Absetzen von Medikamenten, die inzwischen nicht mehr indiziert sind)
- Jede Medikation sollte besprochen und in ihrem Zweck für den Patienten nachvollziehbar erklärt werden.
- Ggf. sollten Hilfsmittel (Dosetts, Medikamententagebuch etc.) zur Verbesserung der Compliance empfohlen werden.
- Gegebenenfalls sollte die Medikation unter den behandelnden Ärzten abgesprochen werden.
- Wenn im Gesundheitsbericht auf mögliche Nebenwirkungen oder eventuelle Unterlassungen hingewiesen wird, sollte eine Überprüfung der Medikation erfolgen.
- Langwirksame Benzodiazepine sollten wenn immer möglich ausgeschlichen werden da die Einnahme dieser Substanzen einen Risikofaktor für Stürze im Alter darstellt.
- Falls keine zwingende Indikation vorliegt, ist es im Hinblick auf das Nebenwirkungsprofil sinnvoll, sollten stark anticholinerg wirksame trizyklische Antidepressiva (Amitryptilin in Saroten Retard®.Limbitrol® und Tryptizol®) durch andere Antidepressive (z.B. selektive Serotonin Wiederaufnahmehemmer) ersetzt werden.

Zusatzinformation Schweiz

Patienten, die noch keinen Medikamentenplan haben, wird im Rahmen der Intervention vom Beratungsteam ein solcher angeboten. (s. Muster im Anhang). Dieser Medikamentenplan wurde aufgrund einer Fokusgruppenarbeit entwickelt und ermöglicht eine ausreichende Schriftgrösse und beinhaltet zudem ein Feld zur Angabe des Verordnungszwecks. Verschiedene Studien belegen, dass Patienten, die gut über die Indikation ihrer Medikamente informiert sind, eine bessere Compliance aufweisen. Zudem ist es von Vorteil, wenn Patienten, die mehrere Medikamente einnehmen, die unterschiedliche Indikation der einzelnen Präparate kennen oder nachschauen können.

Hintergrundinformation

In einem Artikel, der sich mit der Verwendung potentiell ungeeigneter Medikamente bei älteren Patienten befasste, wurden folgende Arzneimittel als besonders risikoreich für unerwünschte Nebenwirkungen bei dieser Personengruppe eingestuft: Amitriptylin (wegen seiner starken anticholinergen und sedierenden Eigenschaften im fortgeschrittenen Alter kein Antidepressivum der ersten Wahl), langwirksame Benzodiazepine (bspw. Flurazepam Dalmadorm oder Diazepam Valium) sowie Chlordiazepoxid Librax, Limbitrol (lang anhaltende Sedierung mit erhöhter Gefahr von Stürzen und Verletzungen).

Referenzen

Beers MH. Explicit criteria for determining potentially inappropriate medication use by the elderly. Arch Intern Med 1997;157:1531-1536

Von Rentelen-Kruse W. Medikamentencompliance. 2000. In: Nikolaus T. Klinische Geriatrie. Springer, Berlin Heidelberg New York; 218-228

Nikolaus T, Specht-Leible N, Bach M. Medikamentenverordnung und mehrfache Stürze bei älteren zu Hause lebenden, multimorbiden Patienten. Z Geront Geriat 1999; 32: 307-311

Vorbeugung gegen Stürze und Verletzungen

Einführung

Ein Drittel aller Menschen über 65 Jahre stürzen jedes Jahr. Stürze sind die häufigste Unfalltodesursache, insbesondere in dieser Altersgruppe. 5-10% der Stürze führen zu Frakturen und 2,2% der Stürze mit Verletzungsfolgen sind tödlich. Auch Stürze ohne Frakturen gehen mit einer erheblichen Morbidität einher und haben beträchtliche psychologische Auswirkungen. Stürze bei älteren Menschen sind oft Ausdruck zugrundeliegender Gesundheitsprobleme oder Risikofaktoren und sind gewöhnlich auf ein Zusammentreffen dieser Faktoren mit umweltbedingten Gefahrenmomenten zurückzuführen. Eine frühzeitige und systematische Risikoanalyse kann in Verbindung mit entsprechenden Interventionen die Häufigkeit von Stürzen senken **(A)**.

Zusätzlich zu einer Anamnese mit einem oder mehreren Stürzen gehören zu den allgemein anerkannten Risikofaktoren bei älteren Menschen:

- Leicht diagnostizierbare Störungen des Gleichgewichts, des Gangbildes oder der Beweglichkeit
- Polymedikation, insbesondere zentral sedierende oder blutdrucksenkende Medikamente

- Sehstörungen
- Kognitive Störungen oder Depression
- Schlaganfälle, Parkinson-Erkrankungen oder degenerative Gelenkerkrankungen der unteren Extremitäten in der aktuellen oder früheren Krankengeschichte
- Orthostatische Hypotonie

Die Massnahmen zur Reduktion des Sturzrisikos im fortgeschrittenen Alter werden immer differenzierter, v.a. was das multidimensionale Assessment und die spezifische Synkopenabklärung angeht. Personen mit bekannten Risikofaktoren für rezidivierende Stürze und besonders diejenigen, die anamnestisch bereits gestürzt sind, werden in ihrem Gesundheitsbericht aufgefordert, sich ärztlich beraten zu lassen.

Empfehlungen

- Ärzte sollten systematisch Risikofaktoren prüfen und nach Möglichkeit die entsprechend geeigneten Schritte einleiten
- Bei Personen mit rezidivierenden Stürzen in der Vorgeschichte oder die zwei oder mehr der obigen Risikofaktoren aufweisen, sollte die Durchführung eines umfassenden Assessment durch einen Geriater oder eine geriatrische Fachabteilung erwogen werden, sofern dies nicht bereits geschehen und ortsnah möglich ist.
- Als hochgradige Risikofaktoren für Osteoporose sind bekannt: röntgenologischer Nachweis einer Osteopenie, Abnahme der Körpergrösse durch osteoporotisch bedingte Wirbelkörperdeformitäten, vorangegangene Frakturen infolge erhöhter Knochenbrüchigkeit, längere Kortikosteroidbehandlung, vorzeitige Menopause in der Anamnese, Hüftfraktur der Mutter in der Familienanamnese und geringer Body-Mass-Index (unter 20 kg/m^2), Rauchen. Diese Faktoren stellen anerkannte Indikationen für eine Risikoabklärung dar (ggf. mittels Osteodensitometrie und/oder Überweisung an entsprechende Fachärzte). Das Behandlungsspektrum umfasst allgemeine und spezifische Verhaltensregeln (erhöhte Calciumzufuhr mit der Nahrung, Vermeidung des Rauchens sowie eines übermässigen Alkohol- und Koffeinkonsums, Widerstands-/Krafttraining) und die medikamentöse Behandlung (Calcium und Vitamin D, Biphosphonate oder Hormonsubstitution) je nach Risikoprofil und Präferenz der einzelnen Person und Schweregrad der Erkrankung.
- Ärztlicherseits oder i.R. einer Gesundheitsberatung sollten Ratschläge zur Vermeidung von Stürzen vermittelt werden (zum Beispiel Verbesserung der Beleuchtung im Hause, Entfernung von losen Teppichen, Einsatz rutschfester Matten in der Badewanne oder nötigenfalls die Installation von Handläufen oder Griffen).
- Im Rahmen einer Gesundheitsberatung sollten auch Ratschläge zur Vermeidung nicht sturzbedingter Verletzungen erteilt werden [zum Beispiel zum sicheren Kochen, kein Rauchen im Bett, Kenntnis der Notausgänge im Brandfall, Verwendung von Sicherheitsgurten, vorsichtiges Autofahren (tagsüber und nur vertraute Routen, Vermeidung von Linksabbiegen auf stark befahrenen Strecken) etc.].

Hintergrundinformation

Randomisierte und kontrollierte klinischen Studien haben gezeigt, dass die Untersuchung älterer Menschen auf verschiedene Sturzrisikofaktoren in Verbindung mit entsprechenden Interventionen geeignet sind, die Häufigkeit von Stürzen in der Folgezeit zu reduzieren **(A)**. Unter Verwendung eines postalisch versandten Fragebogens und anschliessender Intervention bei denjenigen Personen mit hohem Risiko, liessen sich Stürze um 30-40% reduzieren. Eine spezifische Abklärung bei Personen, die innerhalb von zwei Wochen

nach einem Sturz notfallmässig ins Krankenhaus eingeliefert wurden, führte im folgenden Jahr in der Interventionsgruppe, verglichen mit der Kontrollgruppe, zu 60-70% weniger Stürzen.

Zusatzinformation Schweiz

In der Schweiz gibt es derzeit noch keine allgemeingültigen Richtlinien zum Osteoporose-Screening. Bei bestehenden Risikofaktoren wird die Knochendensitometrie als diagnostische Massnahme empfohlen, da sie in der Beurteilung der Knochendichte dem konventionellen Röntgenbild (z.B. der Wirbelsäule) überlegen ist.

In einer kürzlich veröffentliche randomisierte Studie über den Einsatz von Hüft-Protektoren bei Patienten mit erhöhtem Risiko für eine Hüftfraktur konnte das Risiko einer sturz-assoziierten Fraktur um 60% gesenkt werden (Kannus P et al.). Aeltere Patienten, die sturzgefährdet sind und keiner aktiven präventiven Intervention zugeführt werden können, profitieren demnach von solchen Protektoren. Hüftprotektoren werden in der Schweiz unter anderem unter dem Handelsnamen Safe Hip® vertrieben.

Referenzen

Tinetti ME, Speechley M, Ginter SF 1988. Risk factors for falls among elderly people living in the community. N Engl J Med; 319:1701-1707.

Tinetti ME, Baker DI, McAvay G, et al. 1994. A multifactorial intervention to reduce the risk of falling among elderly people living in the community. N Engl J Med; 331:821-827.

Close J, Ellis M, Hooper R, Glucksman E, Jackson S, Swift C. Prevention of Falls in the Elderly Trial (PROFET). A Randomised Controlled Trial. Lancet 1999;353: 93-97

Campbell JA. Randomised controlled trial of a general practice programme of home based exercise to prevent falls in elderly women BMJ 1997;315:1065-1069

Lehmann R, Pfeifer M, Minne H, Alloilio B 2000. Sekundärprävention der Osteoporose und Identifikation von Hochrisikopatienten. ZaeFQ; 94: 445-451

Werle J, Zimber A. Sturzprophylaxe durch Bewegungssicherheit im Alter: Konzeption und Effektivitätsprüfung eines sensomotorischen Interventionsprogramms bei Osteoporose-Patientinnen. Z Geront Geriat 1999; 32: 348-357

Kraenzlin, ME, Rizzoli, R 1999. Abklärung der Osteoporose. Schweizerische Ärztezeitung 1999;80: 536-543.

Kannus P, Parkkari J, Niemi S, et al. Prevention of hip fracture in elderly people with use of a hip protector. N Engl J Med 2000;343:1506-13.

Urininkontinenz

Einführung

Die Prävalenz der Urininkontinenz nimmt mit dem Alter zu. Bis zu 44% der im eigenen Haushalt lebenden Personen im Alter von 75 Jahren und darüber sind davon betroffen. Die Hausärzte sind sich der Inkontinenz bei älteren Menschen oft nicht bewusst. Generell ist die Abklärung und Behandlung von Kontinenzproblemen älterer Menschen bei der Mehrzahl der Betroffenen immer noch unzureichend.

Empfehlungen

- Patienten, die im Gesundheitsfragebogen über wiederholten unfreiwilligen Harnabgang berichten, sollten abgeklärt werden.
- Die Abklärung sollte mit einer Mittelstrahl-Urinkultur beginnen. Sollte die Inkontinenz trotz sterilen Urins fortbestehen, sollte eine eingehende Anamnese nach Hinweisen auf eine Detrusor-Instabiltät oder eine Medikamentennebenwirkung erhoben werden.
- Frauen sollten ausserdem eine gynäkologische Untersuchung (bspw. auf einen klinisch relevanten Prolaps) mit Blasenpalpation bzw. Blasensonographie (zum Ausschluss eines Harnverhalts) erhalten. Ggf. ist eine Überweisung zur weitergehenden fachärztlichen Abklärung (z.B. gynäkologisch oder urologisch, ggf. einschliesslich Uroflowmetrie) sinnvoll. Eine nachgewiesene Stressinkontinenz, bei der ein chirurgischer Eingriff nicht indiziert ist, kann durch Beckenbodentraining, lokale oder systemische Oestrogentherapie oder niedrig dosiertes Imipramin behandelt werden. Eine Übererregbarkeit des M.detrusor i.S. einer instabilen Blase kann, wenn die Diagnose feststeht, durch Blasentraining und/oder Oxybutinin oder Trospiumchlorid behandelt werden.
- Weitere Abklärungen bei Männern zielen auf den Ausschluss einer Überlaufblase bei benigner oder maligner Prostatavergrösserung (rektale Untersuchung und Blasenpalpation/Blasensonographie) sowie den Ausschluss eines Blasenkarzinoms. Angesichts der Bedeutung einer frühzeitigen Diagnose sollte ggf. grosszügig an den Urologen überwiesen werden.

Zusatzinformation Schweiz

Die funktionelle Behandlung der Inkontinenz kann eventuell von Physiotherapeutinnen mit spezieller Weiterbildung angeboten werden. Zu einer guten Therapie gehören Behandlung der Inkontinenz mit gezielten Übungen (Tanzbergerkonzept) Tipps, Aufklärung, Umgang mit Inkontinenz (Verhalten beim Urinlösen) Umsetzung der Übungen im Alltag, d.h. Beckenbodenspannen während des Hustens und Hebens von Lasten. Spezialisierte Zentren bieten neben Kegelübungen zusätzlich apparative Therapie (Bio-Feedback, Elektrostimulation) an.

Hintergrundinformation

Die hohe Prävalenz der Inkontinenz bei älteren Leuten ist weitgehend auf die Kombination prädisponierender Faktoren wie Begleiterkrankungen, Medikation und Immobilität zurückzuführen. Hinzu kommen altersbedingte Veränderungen wie eine verminderte Blasenkapazität, Störungen der Detrusorfunktion, erhöhte nächtliche Natrium-und Flüssigkeitsausscheidung. Bei Frauen können eine durch postmenopausale Schleimhautatrophie oder Beckenbodenschwäche induzierter Urethraverschluss, bei Männern die Harnleiterobstruktion durch Prostatavergrösserung hinzukommen.

Referenzen

Prosser et al. Case-finding incontinence in the over-75's. Br J Gen Pract 1997;47:498-500

Ouslander JG, Johnson TM. Incontinence. Ch 126 pp1595-1614. In: Principles of Geriatric Medicine and Gerontology: Hazzard W et al. 4th edition 1998

Füsgen I, Melchior H. Inkontinenzmanual: Diagnostik – Therapie – Rehabilitation. 2.Aufl.1997. Springer; Berlin Heidelberg New York

Dubuis-Grieder C. Harninkontinenz in der Geriatie.1996, Ars Medici Dossier IV, 9-14

F. Psychosoziale Gesundheit und Verhalten

Screening auf Depression und Demenz

Einführung

Der Gesundheitsfragebogen und seine automatisierte Auswertung sind so konfiguriert, dass sie die frühzeitige Erkennung einer Depression unterstützen. Zusätzlich werden im Fragebogen Hinweise auf eine potentielle kognitive Beeinträchtigung erfasst. In beiden Fällen werden eingehende Empfehlungen an Patienten und Betreuungspersonen an die Hand gegeben, um die frühzeitige Vorstellung zur diagnostischen Abklärung, Behandlung und zur Einleitung weitergehender Unterstützungsmassnahmen sicherzustellen.

Empfehlungen

- Aufgrund der zunehmenden Prävalenz der Demenz im Alter ab 75 Jahren sollten alle Patienten in dieser Altersstufe eine jährliche Screening-Untersuchung mit einem Mini-Mental Status erhalten. Bei jüngeren Patienten besteht Handlungsbedarf, wenn der klinische Verdacht auf ein dementielles Syndrom vorliegt.
- Bei einem im Gesundheitsfragebogen erhobenen Hinweis auf eine Gedächtnisstörung sollte zunächst ein Mini-Mental-Status (MMST, siehe Anhang) erhoben werden. Eine Punktzahl unter 25 ist als Hinweis auf eine potentielle kognitive Störung, und der Patient sollte weitergehend auf einen reversiblen (zum Beispiel durch Vitamin B_{12}-Mangel oder eine Hypothyreose bedingten) oder irreversiblen dementiellen Prozess (zum Beispiel vom vaskulären oder vom Alzheimer-Typ) abgeklärt werden. Hierzu sollte die Überweisung an einen Spezialisten erwogen werden.
- Finden sich im Gesundheitsbericht Hinweise auf Niedergeschlagenheit sollte die Kurzfassung der Geriatrischen Depressionsskala (GDS, siehe Anhang) erhoben werden. Dieser Fragebogen mit 15 Items erfordert 2 Minuten zum Ausfüllen und deckt einen weiten Bereich von Fragen nach der Stimmungslage ab. Er wird als Screeningmethode für bislang unentdeckte Depressionen, zur standardisierten Erfassung eines Ausgangsstatus (vor Behandlungseinleitung) und zur Verlaufskontrolle verwendet. Eine Punktzahl über 5 ist als Hinweis auf eine depressive Störung zu werten.
- Ob unmittelbare Interventionen notwendig sind, liegt im klinischen Ermessen des Hausarztes. Diese kann u.a. die Einleitung oder Überprüfung einer medikamentösen Behandlung, die Überweisung an lokale sozialtherapeutische Einrichtungen (z.B. Tagesklinik) und Hilfsdienste oder zur Abklärung an entsprechende Spezialisten umfassen.

Zusatzinformation Schweiz

Die Antworten auf Fragen zur Erfassung eines möglichen dementiellen Syndromes beruhen auf der Selbsteinschätzung der älteren Person. Bei Patienten mit Demenz ist die Selbsteinschätzung nicht selten beeinträchtigt. Die Befragung im Gesundheitsprofil ersetzt daher die klinische Einschätzung, die genaue Anamnese inkl. Fremdanamnese und objektivere Methoden, wie den MMST nicht.

Hintergrundinformation

In Grossbritannien wurde der Einsatz eines kurzen Screeninginstruments (MMST) zur Feststellung einer kognitiven Störung durch nicht-ärztliches Personal bei 1170 älteren Menschen aus hausärztlichen Praxen im Alter von 75 Jahren und darüber beschrieben. Die Prävalenz einer kognitiven Störung (Punktzahl unter 25 im MMST) betrug 12,8%.

Sechs Prozent der Patienten hatten eine Punktzahl unter 19, doch weniger als ein Drittel dieser Gruppe wies in ihren medizinischen Unterlagen die Diagnose einer Demenz auf. Es gab keinen Zusammenhang zwischen niedrigen Punktzahlen im MMST und Geschlecht oder sozialem Status, es liess sich aber eine Korrelation zum Alter beobachten: der Anteil der Patienten mit Demenz stieg von 2,5% in der Altersgruppe zwischen 75-79 Jahren auf 29,0% bei den 90-jährigen und älteren. Ähnliche Prävalenzen kognitiver Störungen wurden in einer grösseren amerikanischen Studie beim Screening über 60-jähriger Patienten in Hausarztpraxen festgestellt. Mittlere bis schwere Störungen gingen mit einer vermehrten Inanspruchnahme von Gesundheitsdiensten und mit einer erhöhten Sterblichkeit einher.

Trotz positiver Einschätzung der eigenen Befähigung, Depressionen bei älteren Menschen zu erkennen und zu behandeln, verwendeten gemäss einer in den USA durchgeführten Studie nur ein Viertel der Allgemeinpraktiker routinemässig ein Screening-Instrument in ihrer Praxis. In einer Screeningstudie auf Depression in Grossbritannien bei Patienten ab 75 Jahren wurden bei 22% Anhaltspunkte für eine Depression gefunden, die der Hausarzt vorher nicht diagnostiziert hatte.

Referenzen

Iliffe S et al. Screening for cognitive impairment in the elderly using the mini-mental state examination. Br J Gen Pract 1990;40:277-9

Callahan CM et al. Documentation and evaluation of cognitive impairment in elderly primary care patients. Ann Intern Med 1995;122:422-9

Banazak DA. Late-life depression in primary care. How well are we doing? J Gen Intern Med 1996;11:163-7

Iliffe S et al. Assessment of elderly people in general practice. 1. Social circumstances and mental state. Br J Gen Pract 1991 Jan;41(342):9-1

Sandholzer H, Breull A, Fischer GC. Früherkennung und Frühbehandlung von kognitiven Funktionseinbussen: eine Studie über eine geriatrische Vorsorgeuntersuchung im unausgelesenen Patientengut der Allgemeinpraxis. Z Gerontol Geriat 1999;32:172-178

Rauchen

Einführung

Bis zu 30% der über 65 järigen Personen sind Raucher. Pulmonale Erkrankungen, in erster Linie als Folge des Rauchens, sind eine häufige Ursache für Invalidität im Alter. Strategien zur Aufgabe des Rauchens bei „jüngeren“ Alten haben sich als nützlich und kosteneffizient erwiesen, was die Kosten pro neuer Nichtraucher und gewonnenem Lebensjahr betrifft. Doch haben neuere Untersuchungen gezeigt, dass Ärzte weniger geneigt sind, ältere Menschen zur Aufgabe des Rauchens zu bewegen als jüngere.

Empfehlungen

- Den älteren Rauchern werden im Rahmen des Gesundheitsberichts wichtige Informationen und schriftliche Empfehlungen in die Hand gegeben. Diese Empfehlungen zur Aufgabe des Rauchens sollten im Rahmen der Gesundheitsberatung und vom Hausarzt unterstützt werden, wobei mit Nachdruck auf die erwiesenen Vorteile des Rauchverzichtes für das Atmungs- und das Herz-Kreislauf-System hingewiesen werden sollte.

- Der Arzt sollte mit dem älteren Patienten realistische Ziele (als Ansporn) erarbeiten und, wenn keine Kontraindikationen bestehen, die Verschreibung von Nikotin-Pflastern in Erwägung ziehen.
- Informationen über lokale Unterstützungsangebote (Programme, Selbsthilfegruppen) zur Aufgabe des Rauchens sollten den Patienten zugänglich gemacht werden.

Zusatzinformation Schweiz

Alle Raucherinnen und Raucher haben im Rahmen der Intervention die Möglichkeit am Programm „Ziel Nichtrauchen“ der Arbeitsgemeinschaft Tabakprävention teilzunehmen. Hierbei handelt es sich um ein Programm, das basierend auf dem transtheoretischen Modell computer-basiert und anhand der Antworten auf einen Fragebogen Beratungsbriefe entwirft und den Teilnehmern in 3 monatigen Abständen nach Hause schickt. Mit jedem Brief wird eine stufenspezifische Broschüre beigelegt. Anschrift: at: Arbeitsgemeinschaft Tabakprävention Schweiz, Effingerstrasse 40, 3001 Bern, Tel. 031 389 92 46, Fax 031 389 92 60, internet: www.at-schweiz.ch. Die Indikation zur Subsitutution mit Nikotinpflaster wird aber weiterhin durch den Hausarzt gestellt.

Hintergrundinformation

Wie bei jüngeren Menschen stellt auch bei älteren Personen die Motivation den wichtigsten prognostischen Faktor für eine erfolgreiche Aufgabe des Rauchens (>= 1 Jahr) dar. Beratungsprogramme können bei gut motivierten älteren Menschen Erfolgsquoten von 15% erreichen, die sich noch auf 20% steigern lassen, wenn sie mit einer Nikotinersatztherapie verbunden werden **(A)**. Auch gleichzeitige körperliche Trainingsprogramme konnten nachweislich die Wirksamkeit verhaltenstherapeutischer Programme zur langfristigen Raucherentwöhnung bei Frauen verbessern **(A)**.

Soweit Daten zur Verfügung stehen, scheint der Gewinn durch die Aufgabe des Rauchens bei jungen und alten Menschen vergleichbar zu sein. Der günstige Effekt einer Abstinenz auf die Erholung der durch das Rauchen zunehmend eingeschränkten Lungenfunktion konnte bis zum Alter von 80 Jahren, insbesondere bei Frauen, nachgewiesen werden. Die Reduktion des Risikos eines Herzinfarktes nach Aufgabe des Rauchens (Das Risiko liegt nach 2-3 Jahren praktisch auf gleichem Niveau wie vor dem Rauchen) ist ebenfalls unabhängig vom Alter.

Daten aus den USA lassen vermuten, dass ältere Raucher eher dazu neigen, Belege für die gesundheitsschädigende Wirkung des Rauchens zu negieren. Ob dies aber auch für ältere Raucher in Westeuropa gilt, ist offen.

Referenzen

Connoly JM. Smoking cessation in old age: closing the stable door? Age and Ageing 2000;29:193-5

Vetter NJ, Ford D. Smoking prevention among elderly people aged 60 and over: a randomised controlled trial. Age and Ageing 1990;19:164-8

Martin-Diener, E. Rauchentwöhnung per Computer, Prävention und Gesundheitsförderung im Kanton Zürich 2000, 8: 4-6.

Alkoholkonsum

Einführung
Das Ausmass der Alkoholprobleme unter älteren Menschen wird weit unterschätzt. Auch ein mengenmässig gleichbleibender Alkoholkonsum kann im Alter aufgrund altersbedingter physiologischer und pharmakodynamischer Veränderungen problematisch werden. Diese Veränderungen führen in der Folge zu einer erhöhte Empfindlichkeit gegenüber toxischen Wirkungen des Alkohols.

Empfehlungen
- Die im Gesundheitsbericht enthaltenen Empfehlungen sollten aufgegriffen und bekräftigt werden.
- Anamnestische Indikatoren für exzessives Trinken sind: wiederholte Stürze, Schlaflosigkeit, Mangelernährung, Inkontinenz, soziale Absonderung, Gedächtnisstörungen, Angst und Depression.
- Im Rahmen der ärztlichen Untersuchung sollte nach Hinweisen auf Traumata, Kardiomyopathie (einschliesslich Vorhofflimmern), eine periphere Neuropathie und Symptome der chronischen Leberschädigung gesucht werden.
- Erhöhte Gamma-GT- und GOT-Werte sind die empfindlichsten Laborparameter für chronischen Alkoholmissbrauch. Das mittlere Erythrozytenvolumen (MCV) ist häufig erhöht. Eine Anämie ist oft multifaktoriell bedingt und kann mit einer Thrombozytopenie kombiniert sein.
- Potentielle Interaktionen zwischen Medikamenten und Alkohol beispielsweise, mit Antihypertensiva, Opiat-Analgetika, Marcumar, Antidepressiva und Antikonvulsiva sind zu beachten.
- Die Interventionen hängen vom Ermessen des Hausarztes ab. Kurzfristige Massnahmen könnten bestehen in der Behandlung einer zugrunde liegenden Depression, einer Mangelernährung und damit einhergehenden Stoffwechselstörungen, oder im Einbeziehen von Familienangehörigen, um eine Verleugnungshaltung des Patienten zu überwinden. Langfristige Massnahmen könnten in der Vermittlung einer ambulanten oder stationären Entzugsbehandlung und Hinweisen auf Selbsthilfegruppen liegen.

Zusatzinformation Schweiz
Massvoller Alkoholkonsum kann auch günstige gesundheitliche Auswirkungen haben. Ergebnisse einer grossen Kohortenstudie haben z.B. gezeigt, dass Männer die regelmässig geringe Mengen Alkohol konsumieren ein geringeres Insultrisiko haben. Die Menge Alkohol pro Tag die unbedenklich konsumiert werden kann, sollte aber für jeden Patienten individuell festgelegt werden. Zu beachten ist insbesondere, dass bei Patienten in höherem Alter, Patienten mit Psychopharmaka, oder Patienten mit Sturzanamnese bereits geringe Mengen Alkohol (.z.B. ein Glas Wein) die Sturzgefährdung deutlich erhöhen können.

Hintergrundinformation
5%-12% der Männer und 1-2% der Frauen im Alter von 60 Jahren sind Problemtrinker. Die Prävalenz auf Notfallstationen ist erheblich höher. Eine Studie mit 1070 älteren Männern und Frauen aus Hausarztpraxen ergab, dass unter den Personen, die Alkohol zu sich nahmen, etwa ein Fünftel gewohnheitsmässig zu hohe Alkoholmengen konsumiert.

Sowohl die Alkoholmenge als auch die Häufigkeit des Trinkens – und damit die Häufigkeit von Alkoholproblemen – ist bei älteren Männern höher als bei älteren Frauen. Durchschnittlich trinken ältere Menschen weniger als jüngere, aber mit dem Alter ändert sich das Trinkverhalten nicht immer und übermässiger Alkoholkonsum wird möglicherweise im höheren Alter uneingeschränkt fortgeführt. Die Tendenz älterer Menschen, ihren Alkoholkonsum zu reduzieren, scheint bei Frauen weniger ausgeprägt zu sein.

Alte Menschen tolerieren die unerwünschten Wirkungen des Alkohols häufig schlechter. Die Mechanismen sind nicht geklärt. Mögliche Mechanismen sind bei einer lipophilen Substanz wie Alkohol: die Zunahme des Verteilungsvolumens (durch Erhöhung des Verhältnisses von Körperfett zu Körperwasser), ein geringerer First-Pass-Effekt in der Leber und/oder die erhöhte Empfindlichkeit des ZNS.

Die zur Zeit geltenden Empfehlungen für die Grenzen eines unbedenklichen Alkoholkonsums des Nationalen Instituts für Alkoholmissbrauch und Alkoholismus in den USA sind für Personen über 65 Jahren wegen ihrer besonderen Empfindlichkeit gegenüber den toxischen Effekten des Alkohols auf nicht mehr als ein alkoholisches Getränk pro Tag nach unten korrigiert worden.

Referenzen

Dunne, FJ. Editorial. Misuse of alcohol or drugs by elderly people. BMJ 1994;308:608-609

Dufour MC, Archer L, Gordis E. Alcohol and the elderly. Clin Geriatr Med 1992;8:127-141

4. Anhang

I Tabellarische Zusammenfassung der Empfehlungen
II Der Gesundheits-Profil Fragebogen
III Der Gesundheitsbericht
IV Der zusammenfassende Bericht für den Hausarzt
V Ausgewählte Assessment-Instrumente
VI Ausgewählte Literatur
VII Diverse Projektinformationen

Anhang (I): Zusammenfassung präventivmedizinischer Empfehlungen bei Personen über 65 Jahre

Allen Patienten, die 65 Jahre und älter sind sollten Maßnahmen der primären Prävention zuteil werden. Patienten, bei denen mittels des Gesundheitsfragebogens und/oder Anamnese bestehende Gesundheitsrisiken identifiziert wurden, sollten wiederholte Beratungen durch behandelnde Ärzte und ergänzende Gesundheitsberater erhalten.

Domäne	Empfehlung	Risikoanalyse und Gesundheitserhaltung
Hypertonie	Jährliche Blutdruckmessung	Bei allen: Diät, körperliche Bewegung, Natrium-Zufuhr↓. BD 140-159/90-99: medikamentöse Therapie bei kardialem Risiko/Diabetes/Endorganschaden, sonst beobachten. SBD >160 oder DBD >100: medikamentöse Therapie. Zielbereich BD < 140/85. Thiazide 1. Wahl. Bei kardiovaskulärem Risiko >=15% ASS
Hypercholesterinämie	Cholesterinbestimmung alle 5 Jahre / Coronary Risk Prediction Chart	Gesamtcholesterin >= 5 mmol/l und kardiovaskuläres Risiko >=30%: Wenn Chol. trotz 3-6 Monaten Diät/Nikotinkarenz/Gewichtsabnahme/körp. Aktivität >= 5 mmol/l: Statine
Diabetes mellitus	Nüchtern-BZ Messung alle 3 Jahre	Nü-BZ > 7.0 mmol/l: Diätbehandlung, Glimepirid oder anderer kurzwirksamer Sulfonylharnstoff. Interdisziplinärer Zugang zur Compliance-Förderung/Berücksichtigung der Komorbidität
Influenza Impfung	Jährlich bei allen Patienten	Influenza- und Pneumokokkenimpfung wirksam bei allen Patienten mit hohem Risiko (z.B. mit kardiopulmonaler Erkrankung oder Immunsuppression) ***und*** niedrigem Risiko.
Pneumovax	Alle 10 Jahre bei allen Patienten	
Kolonkarzinom	Jährlicher Test auf occultes Blut	Nur wenn ggf. weitergehende Untersuchungen wie Koloskopie bei positivem Test durchführbar
Mammakarzinom	Jährlich klinische Vorsorg, bei Verdacht oder bes. Risiko Mammographie	Wenn möglich, zusätzlich Anleitung zur Selbstuntersuchung
Zervixkarzinom	PAP-Abstriche i.R. d. Vorsorgeuntersuchung alle 3 Jahre	Bis zum Alter von 70 Jahren. Nach 70 Jahren nicht mehr, soweit vorher 3 Abstriche negativ waren
Zahnvorsorge	Jährlicher Zahnarztbesuch	alle Patienten
Augenkontrolle	Jährliche Kontrolle inkl. Druckmessung	alle Patienten, bei Sehstörungen laut Gesundheitsfragebogen fachärztliche Abklärung
Gehörprüfung	Jährlicher Flüstertest	Otoskopie und Überweisung zur Audiometrie bei Abnahme des Gehörs
Bewegung	Im Rahmen der Möglichkeiten regelmässige körperliche Bewegung	Alter alleine ist keine Kontraindikation, Zielsetzungen fördern
Ernährung	Allgemeine Ernährungsberatung	Empfohlen werden tägl. mindestens 5x Früchte oder Ballaststoffe. Zufuhr gesättigter Fette↓, Erfassung von Malnutrition
Funktionsprüfung	Periodische Funktionsprüfung bei Personen mit entsprechender Komorbidität	Untersuchung, wenn klinische Vorzeichen der Behinderung durch den Gesundheitsfragebogen identifiziert wurden. Interdisziplinäres Assessment bei Patienten, die in mehr als einer Tätigkeit des täglichen Lebens eingeschränkt sind
Schmerz	Regelmässige Schmerzananmese	Sorgfältige Evaluation um Über- und Unterdosierungen (z.B. mit NSAR) zu vermeiden
Medikation	Konzentration auf Wesentliches, Medikamententagebuch	Überprüfung der Indikationen, Hilfen zur Verbesserung der Compliance, Vermeiden von lang wirksamen Benzodiazepinen und Amitriptylin
Unfallverhütung/Sturz	Jährliche Überprüfung auf Risikofaktoren	Patientenaufklärung bez. Fallrisiken und Unfallverhütung, umfassendes Sturzassessment, ggf. Osteoporosebehandlung
Inkontinenz	Regelmässige Befragung nach Symptomen	Ausschluss Harnwegsinfekt, funktionelle oder medikamentös induzierte Inkontinenz, Restharn – zur weiteren Abklärung ggf. Überweisung zum Facharzt
Demenz/Depression	Screening für alle ≥ 75 Jahre	Weitere Demenzabklärung bei MMSE <25, weitere Depressionsabklärung bei GDS >5
Rauchen	Beratung in Richtung Nikotinkarenz	Verhaltenstherapeutisches Vorgehen mit oder ohne Nikotinsubstitution
Alkoholkonsum	Regelmässige Frage nach Ausmass des Konsums	Unbedenklich: 1 alkoholisches Getränk/Tag (weniger bei Psychopharmaka). Cave: Gesundheitsfragebogen kann gefährlichen Alkoholkonsum unterschätzen

S/D BD=systolischer/diastolischer Blutdruck, MMSE=Minimental Status Exam, GDS=Geriatric Depression Scale
