# Supplementary material for: Effect of Health Risk Assessment and Counselling on Health Behaviour and Survival in Older People: A Pragmatic Randomised Trial
Source: PLoS Med. 2015 Oct 19;12(10):e1001889. doi: 10.1371/journal.pmed.1001889 (PMC4610679; doi:10.1371/journal.pmed.1001889)
Supplement: S7 Text — (PDF) [file pmed.1001889.s019.pdf]

Supporting Information S7 Text

Sensitivity Analysis of Two-Year Outcome Results Accounting for Attrition

for: A Collaborative Care Model of Health Risk Assessment and Counselling in Older Persons: A Randomised Clinical Trial

Additional Sensitivity Analysis of Two-Year Outcome Results

For the multiple imputation approach we assumed a missing at random assumption [1]. Some individuals were lost-to-follow-up at 2 years, where a missing at random situation possibly does not hold. We conducted an additional sensitivity analysis to test the potential impact of an attrition bias [2]..

We had mainly three reasons for LTFU (Loss to Follow-Up): 1) practice withdrawn, 2) moving away, 3) other reason. Mainly the first two reasons gave indication for a missing-not-at-random situation. The proportion of individuals which were lost-to-follow-up (LTFU) for the two year outcomes is 6.2% (132 individuals out of 2,147 persons). We defined LTFU if all of the primary outcomes described in Table 2 in the manuscript were completely missing. Of the 132 individuals, 106 (80.3%) individuals were LTFU because of the practice which withdraw from the study, 21 (15.9%) individuals because they moved away, and 5 (3.8%) individuals because of other reasons. We assessed if LTFU was associated with baseline characteristics age, gender, items of the Pra questionnaire, and the neighbourhood index of socioeconomic position (Swiss-SEP), using logistic regression models, for three dropout situations 1) practice withdrawn, 2) moving away, 3) overall (practice withdrawn, moving away, other reason). We did not assess LTFU for “other reasons”, because only five individuals had this situation. We then used inverse-probability-of-attrition weighting (IPAW) to examine the influence of attrition bias on the group allocation and the 2 year outcomes [2]. We used only models for overall attrition. IPAW accounts for the fact that the chance of being not-lost-to-follow-up was not the same for all patients, and up-weights individuals with characteristics similar to individuals with missing follow-up information [2]. We calculated probabilities of being not-lost-to-follow-up using a logistic regression model with predictors which were associated with overall attrition (i.e. hospital admission and Swiss-SEP), and the group allocation. These weights were used in the primary 2 year outcome analysis.

Table A1 reports the Odds Ratios (OR) from the logistic regression models. We found that at least one hospital admissions was associated with a higher risk of attrition due to practice withdrawn (OR 2.08 95%CI (1.36, 3.18)). Further a socioeconomic neighbourhood index greater or equal than 61.1 (median value) was associated with a lower risk of attrition due to practice withdrawn (OR 0.23 95%CI (0.14, 0.38)). There was no evidence of an association of the baseline characteristics with attrition due to moving away. For overall attrition associations were slightly less pronounced than for practice withdrawn (at least one hospital admissions: OR 1.78 95%CI (1.20, 2.65)) and Swiss-SEP (Swiss-SEP greater or equal than 61.1, OR 0.40 95%CI (0.27, 0.58)). Table A2 shows the primary outcome sensitivity analysis using IPAW. Probability weights ranged from 1.03 to 1.14 (mean=1.07, median=1.08). Estimates were similar to complete case and multiple imputation results.

References:

[1] Spratt M, Carpenter J, Sterne JA, et al. Strategies for multiple imputation in longitudinal studies. American journal of epidemiology. Aug 15 2010;172(4):478-487.

[2] Weuve J, Tchetgen Tchetgen EJ, Glymour MM, Beck TL, Aggarwal NT, Wilson RS, Evans DA, Mendes de Leon CF. Accounting for bias due to selective attrition: the example of smoking and cognitive decline. Epidemiology 2012;23:119-128.

Table A1: Odds ratios (OR) of attrition for selected baseline characteristics, by attrition reasons

		Attrition due to practice withdrawn		Attrition due to moving away		Overall attrition	
		OR (95%CI)	p-value	OR (95%CI)	p-value	OR (95%CI)	p-value
Group allocation	Control	Reference	0.71	Reference	0.62	Reference	0.73
	Intervention	0.93 (0.62, 1.39)		0.80 (0.32, 1.98)		0.94 (0.65, 1.35)	
Age	65-69	Reference	0.65	Reference	0.20	Reference	0.43
	70-74	1.13 (0.71, 1.82)		0.42 (0.09, 1.87)		0.95 (0.61, 1.49)	
	75-79	1.14 (0.63, 2.08)		1.98 (0.69, 5.67)		1.29 (0.78, 2.16)	
	80+	1.65 (0.77, 3.55)		1.95 (0.43, 8.84)		1.63 (0.81, 3.25)	
Gender	Male	Reference	0.61	Reference	0.38	Reference	0.96
	Female	1.11 (0.74, 1.65)		0.68 (0.29, 1.61)		0.99 (0.69, 1.41)	
Self-perceived health	Excellent/Very good/Good	Reference	0.35	Reference	0.45	Reference	0.60
	Fair/Poor	0.79 (0.47, 1.31)		1.46 (0.57, 3.80)		0.89 (0.57, 1.38)	
Hospital use in past year	Zero admissions	Reference	0.001	Reference	0.60	Reference	0.01
	≥ 1 admissions	2.08 (1.36, 3.18)		0.73 (0.21, 2.49)		1.78 (1.20, 2.65)	
Doctor visits in past year	< 7 visits	Reference	0.11	Reference	0.98	Reference	0.15
	≥ 7 visits	1.43 (0.94, 2.20)		1.02 (0.37, 2.79)		1.34 (0.91, 1.98)	
Self-reported diabetes	No diabetes	Reference	0.999	Reference	0.67	Reference	0.77
	Reported diabetes	1.00 (0.54, 1.85)		1.31 (0.38, 4.48)		1.09 (0.63, 1.87)	
Self-reported coronary heart disease	No CHD	Reference	0.88	Reference	0.74	Reference	0.99
	Reported CHD	1.04 (0.65, 1.66)		0.83 (0.28, 2.48)		1.00 (0.65, 1.52)	
No informal caregiver available if needed	No informal caregiver available	Reference	0.91	Reference	0.86	Reference	0.74
	Informal caregiver available	0.96 (0.51, 1.83)		0.88 (0.20, 3.79)		0.91 (0.50, 1.63)	
Swiss-SEP	< 61.1	Reference	<0.001	Reference	0.28	Reference	<0.001
	≥ 61.1	0.23 (0.14, 0.38)		1.63 (0.67, 3.96)		0.40 (0.27, 0.58)	

Table A2. Primary Outcomes at 2-Year Follow-up: Sensitivity Analysis Based using Inverse-Probability-of-Attrition weights.[a]

Outcome	Intervention Group	Control Group	Odds Ratio (95% CI)[c]	*P* Value
	No./ Total (%)[b]			
**Health behaviours**				
Medium to high level of physical activity (≥ 30 minutes per day)[d]	519/719 (72.2)	731/1161 (63.0)	1.52 (1.22–1.89)	<0.001
Medium to high level of fruit/ vegetable/ fiber intake (≥ 2 portions per day)	346/727 (47.6)	456/1174 (38.8)	1.43 (1.16–1.76)	<0.001
Low level of fat intake (< 2 portions of high fat items per day)	225/734 (30.7)	297/1185 (25.1)	1.32 (1.06–1.65)	0.01
Use of seat belt (always use of seat belt)	650/729 (89.2)	1011/1194 (84.7)	1.48 (1.10–1.99)	0.009
No tobacco consumption	660/730 (90.4)	1066/1189 (89.7)	1.09 (0.79–1.50)	0.61
No or little alcohol use (≤ 1 alcoholic drink per day)	685/733 (93.5)	1072/1191 (90.0)	1.59 (1.11–2.27)	0.01
**Adherence with selected preventive care recommendations**[e]				
Blood pressure measurement in past y	705/766 (92.0)	1069/1210 (88.3)	1.51 (1.10–2.08)	0.01
Cholesterol measurement (persons aged <75 y) in past 5 y	400/444 (90.1)	624/722 (86.4)	1.43 (0.99–2.09)	0.06
Glucose measurement in past 3 y	622/766 (81.2)	925/1197 (77.3)	1.27 (1.01–1.60)	0.04
Influenza vaccination in past y	496/751 (66.1)	707/1194 (59.2)	1.34 (1.09–1.65)	0.006
Pneumococcal vaccination (ever)	225/732 (30.7)	221/1151 (19.2)	1.87 (1.48–2.36)	<0.001
Faecal occult blood test in past y (persons aged <80 y)	167/606 (27.6)	205/976 (21.0)	1.44 (1.13–1.83)	0.003

[a] CI denotes confidence interval.
[b] Total is the number of persons with available data per outcome. One reason for the variable denominators is the variable definition of the target participant group for cholesterol measurement and faecal occult blood test. For cholesterol measurement, the target group was persons aged <75 yr, and for fecal occult blood testing, the target group was persons aged <80 yr, respectively. The other reason for the variable denominators is different numbers of missing data per outcome. An example: The denominator for the physical activity outcome in the intervention group is 719. As indicated in the flow diagram (Figure 1 main manuscript), 779 of 827 surviving persons in the intervention group answered the 2-yr follow-up questionnaire. Among the 779 person, 60 did not respond to the physical activity question, leaving 719 persons with complete data on physical activity at the 2-yr follow-up.
[c] Control group is reference group.
[d] Based on participant self-reported answers to average daily duration of moderate or strenuous level of physical activity.
[e] Based on abstraction of primary care physicians' patient charts.
